# A reduced panel of eight genes (*ATM*, *SF3B1*, *NOTCH1*, *BIRC3*, *XPO1*, *MYD88*, *TNFAIP3*, and *TP53*) as an estimator of the tumor mutational burden in chronic lymphocytic leukemia

**DOI:** 10.1111/ijlh.13435

**Published:** 2020-12-16

**Authors:** Jasmine Chauzeix, Cédric Pastoret, Lucie Donaty, Nathalie Gachard, Thierry Fest, Jean Feuillard, David Rizzo

**Affiliations:** ^1^ Laboratoire d'Hématologie et UMR CNRS 7276/INSERM 1262 CRIBL Centre de Biologie et de Recherche en Santé CHU et Université de Limoges Limoges France; ^2^ Inserm MICMAC ‐ UMR_S 1236 CHU Rennes Université Rennes 1 Rennes France

**Keywords:** chronic lymphocytic leukemia, high‐throughput sequencing, prognosis, tumor mutational burden

## Abstract

**Introduction:**

Mutational complexity or tumor mutational burden (TMB) influences the course of chronic lymphocytic leukemia (CLL). However, this information is not routinely used because TMB is usually obtained from whole genome or exome, or from large gene panel high‐throughput sequencing.

**Methods:**

Here, we used the C‐Harrel concordance index to determine the minimum panel of genes for which mutations predict treatment‐free survival (TFS) as well as large resequencing panels.

**Results:**

An eight gene estimator was defined encompassing *ATM*, *SF3B1*, *NOTCH1*, *BIRC3*, *XPO1*, *MYD88*, *TNFAIP3*, and *TP53*. TMB estimated from either a large panel of genes or the eight gene estimator was increased in treated patients or in those with a short TFS (<2 years), unmutated *IGHV* gene or with an unfavorable karyotype. Being an independent prognostic parameter, any mutation in the eight gene estimator predicted a shorter TFS better than Binet stage and *IGHV* mutational status among patients with an apparently non‐progressive disease (TFS >6 months). Strikingly, the eight gene estimator was also highly informative for patients with Binet stage A CLL or with a good prognosis karyotype.

**Conclusion:**

These results suggest that the eight gene estimator, that is easily achievable by high‐throughput resequencing, brings robust and valuable information that predicts evolution of untreated patients at diagnosis better than any other parameter.

## 1 INTRODUCTION

Chronic lymphocytic leukemia (CLL) is one of the most frequent B‐cell lymphoproliferative disorder of the elderly and is characterized by a lymphocytosis exceeding 5 G/L, composed of small circulating monomorphic round B lymphocytes, with almost constant infiltration of bone marrow and secondary lymphoid organs.^1^, ^2^ CLL disease ranges from very indolent, with patients surviving decades without any treatment, to rapidly progressive forms, leading to death, and occasionally undergoing transformation to aggressive lymphoma known as Richter syndrome. From a clinical point of view, Binet and Rai classifications are the most reliable staging systems to predict overall survival.^3^, ^4^ However, evolution remains rather heterogeneous within each prognostic group. Among the numerous other published prognosis factors, only IGHV mutational status and the presence of *TP53* mutations or deletions, are consensually accepted as predictive for CLL progression and resistance to therapy, respectively.^5^, ^6^, ^7^ Unmutated IGHV (UM‐CLL) genes are strongly associated with poor overall survival, while Binet stage A patients with mutated *IGHV* (M‐CLL) genes have a very good prognosis, especially when serum protein electrophoresis is normal.^5^, ^6^, ^8^ Other prognostic cytogenetic markers include isolated del(13q), which is associated with good prognosis, del(11q), which is thought to correspond to *ATM* inactivation and trisomy 12, which defines a CLL group with an intermediate prognosis.^9^, ^10^

Description of the mutational landscape either by whole exome or whole genome sequencing identified new mutations in CLL such as those of *SF3B1*, *NOTCH1*, *BIRC3* that are associated with a poor prognosis as well as CLL driving mutations such as those of *ATM* or *MYD88*.^11^, ^12^, ^13^, ^14^ Puente et al^15^ and Burns et al^16^ showed that the number of mutations in driver genes is increased in UM‐CLL when compared to M‐CLL patients. Puente et al^15^ also reported that an increased number of driving mutations in CLL is associated with shorter treatment‐free survival (TFS) in CLL patients. The prognostic interest of the number of accumulated mutations has also been evaluated from large panels of resequenced genes.^17^, ^18^

With the aim to bring the maximum information while sequencing the minimum number of genes, we raised the question whether the prognostic information of the number of accumulated mutations at diagnosis could be estimated from a reduced panel of genes. We first evaluated the number of accumulated mutations or tumor mutational burden (TMB) from two resequencing panels of 70 and 65 genes in two completely independent series of 80 and 70 CLL patients, respectively. The C‐Harrel concordance index was used to search for the most informative genes and to define a reduced panel of genes to predict TFS. This TFS predictor was compared to Binet stage, *IGHV* mutational status, and cytogenetic abnormalities.

## 2 MATERIALS AND METHODS

### 2.1 Patients

We analyzed 150 samples from patients with typical CLL, diagnosed between 1980 and 2018, from two University Hospital Centers (series 1, n = 80 and series 2, n = 70). Their Matutes score was 4 or 5 by flow cytometry in all cases. Inclusion criteria were based on the availability of biological samples and cytogenetic results. Flowchart of patients is presented in Figure S1. TMB, the eight gene estimator (see Section 3), Binet stage, cytogenetic abnormalities and their *IGHV* mutational status were analyzed.

### 2.2 DNA extraction

Genomic DNA was extracted from peripheral blood mononuclear cells using the QIAamp DNA Blood Mini Kit (Qiagen) according to the manufacturer's instructions.

### 2.3 High‐throughput sequencing

Patients were assayed by high‐throughput sequencing (HTS). Sequences of primers are listed in Supplementary Information. Cases from series 1 were sequenced using a panel of targeted regions known to be involved in lymphoma (see Table S1). This panel spanned 221.6 kb and was designed on the AmpliSeq designer platform (www.ampliseq.com). Libraries were constructed using the Ion AmpliSeq Library kit 2.0 (Thermo Fischer Scientific) according to the manufacturer's instructions and sequenced on Proton (IonTorrent, Thermo Fisher). Cases from series 2 were sequenced using a custom panel of 122.2 kb targeting 65 genes known to be involved in hematological neoplasms (see Table S2). Libraries were generated in duplicate for each patient, using Advanta NGS library prep kit on 48.48 Access Array system (Fluidigm) and sequenced on NextSeq550 (Illumina).

### 2.4 Variant annotation

To overcome the fact that the germline counterpart was not available, variants were filtered to retain exonic and pathogenic acquired mutations based on a methodology previously described^19^ according to an in‐house pipeline (Supplementary material and methods). Briefly, we first restricted the analysis to variants with a sequencing depth ≥100× and supported by ≥5 mutated reads. Minimum variant allele frequency (VAF) was set to 2%. Highly recurrent hotspot mutations in CLL were identified based on bibliography and by systematic screening of databases for cancer mutations (COSMIC, IARC TP53 databases) for all variants. Validation of non–hotspot mutations needed either positive prediction of pathogenicity by the bioinformatic scores SIFT and/or CADD in order to control the false discovery rate of new variants. Variants with no available classification by SIFT or CADD scores were also evaluated but all tolerated variants were not considered. We then systematically screened databases for polymorphisms (dbSNP, GnomAD, 1000 genomes). All variants with a minor allele frequency (MAF) in the general population ≥0.01 were considered as polymorphisms. For other variants with an entry in dbSNP, GnomAD or 1000 genome databases but with MAF <0.01 or with absence of available annotations were specifically reviewed by two of us (JC, DR) to strictly retain highly plausible mutations only.

### 2.5 Immunoglobulin gene sequence analysis

Amplification of *IGH* gene rearrangements was performed using VH Leader or FR1 primers and JH or CH primers as described in ERIC guidelines.^20^ Analysis of VDJ sequences was assessed with IMGT/V‐QUEST (http://www.imgt.org/IMGT_vquest/analysis) or IGBLAST for complex rearrangements (https://www.ncbi.nlm.nih.gov/igblast).

### 2.6 Cytogenetics

Conventional cytogenetic and fluorescence in situ hybridization were performed after IL2 plus DSP30 stimulation, according to recommendations of the French Group for Hematological Cytogenetics.^21^, ^22^ Quantitative multiplex PCR of short fluorescent fragment (QMPSF) was performed as described elsewhere.^23^

### 2.7 Statistics

The chi‐square test was used to evaluate the difference between categorical covariates for TMB or the eight gene estimator subgroups when sufficient patient numbers were achieved. For small sample size, chi‐square test was replaced by Fisher's exact test. The Mann and Whitney test was used to compare lymphocyte blood counts between treatment naïve patients of both series. The effects of genetics (IGHV mutational status, individual *gene* mutations), cytogenetics (trisomy 12, del(11q), del(13q) and del(17p)), TMB (two subgroups with a threshold at two or more mutations), and the eight gene estimator (two subgroups: mutated vs unmutated) covariates on treatment‐free survival (TFS) were examined among previously untreated Binet stage A patients using the Cox proportional hazard model^24^ processed with the Survival R package (https://github.com/therneau/survival). Briefly, each covariate was first tested in univariate analysis. All significant covariates with a *P*‐value below 0.20 after univariate analysis were included simultaneously in the multivariate model. The significance of variables in the final model was tested by a backward stepwise process using the likelihood ratio to evaluate the effect of omitting variables. The stability of the final model was validated by performing 1000 bootstrap samples. To look at the individual relationship between a given parameter and the survival rate, we used the C‐Harrel concordance index, which is designed to estimate the concordance probability (hereafter the informativeness) by comparing the rankings of two independent pairs of survival times *Ti*,*Tj* for patients *i* and *j*, and the variable *xi*, *xj*.^25^ Roughly, a pair (*i*,*j*) is concordant if *xi* < *xj* and *Ti* < *Tj*. The c‐Harrel index corresponds to the proportion of concordant pairs. Here, *xi* and *xj* take the value 0 or 1 according to the criteria studied (mutational status of a given gene, mutational complexity, Binet stage A vs B…). The C‐Harrel concordance index can also be interpreted as a summary measure of the area(s) under the time dependent ROC curve(s).^26^ With each variable being coded as 1 (present) or 0 (absent), the C‐Harrel concordance index was calculated with the “rcorr.cens” command of the Hmisc package (https://github.com/harrelfe/Hmisc).

## 3 RESULTS

### 3.1 Population characteristics and distribution of accumulated mutations

The impact of TMB was studied in a series of 150 CLL patients issued from two hospital centers (n = 80 and 70 cases each). Clinical, biological, and cytogenetic characteristics are shown in Table S3. From the first series, 34 patients (43%) had been previously treated or were under therapy. Among the 46 treatment‐naive (untreated) patients of the first series 36 (80.0%), 7 (15.6%) and 2 (4.4%) were Binet stage A, B, and C, respectively. These percentages were comparable for the second series. The two series were similar in terms of age, sex ratio, hemoglobin levels, and platelet counts. Distribution of untreated patients with *IGHV* mutated or unmutated genes (M‐CLL vs UM‐CLL) was similar in both series with 52.3% and 52.9% M‐CLL and median treatment‐free survival (TFS) according to the *IGHV* mutational status being comparable (4.1 and 4.7 years for M‐CLL, and 1.1 and 2.0 years for UM‐CLL for each series, respectively). Distribution of accumulated mutations among patients with their frequencies is shown in Figures S2 and S3 for the first series and in Figures S4 and S5 for the second series. As expected, *ATM*, *SF3B1*, *NOTCH1* and *BIRC3* were the most frequently mutated genes in both series of patients. Median TMB was comparable in both series (1.74 and 1.75 for series 1 and 2, respectively).

### 3.2 Relationship between TMB and TFS in CLL patients

We then studied the TMB impact on TFS for the 110/116 (95%) Binet stage A and B untreated patients of the entire series. As shown in Figure S6, and consistent with previous reports,^15^, ^17^ TFS decreased with accumulation of mutations, with a similar poor prognosis for patients with two or more mutations. Patients were thus separated into two categories, those with a TMB below 2 and those with a TMB equal or greater than 2. TFS was significantly decreased among patients with TMB equal to or greater than 2 (Figure S7, mean TFS = 1.5 and 4.3 years for high and low TMB, respectively, logrank test *P* = .0001). This was also true when both series were analyzed separately (Figure S8). Thus, we confirm that TMB is an informative prognostic parameter in CLL.

### 3.3 Definition of the eight gene estimator

We next used the C‐Harrel concordance index to identify the most informative genes.^27^ The farthest from 0.5 is the C‐Harrel concordance index, the highest is the predictive survival informativeness of a given criteria. A C‐Harrel concordance index <0.5 indicates a negative impact on TFS, whereas a value >0.5 means a positive effect. C‐Harrel concordance index values are presented in Table S4. As expected, *P*‐values of the logrank test were statistically significant for the lowest values of the C‐Harrel concordance index. Next, we searched for the combination of genes that minimizes the C‐Harrel concordance index. This analysis identified a panel of eight genes: *SF3B1*, *ATM*, *NOTCH1*, *BIRC3*, *XPO1*, *TNFAIP3*, *MYD88* and *TP53*, a combination of which gave the maximum predictive information for TFS (Table S5). With a C‐Harrel concordance index of 0.408, prognostic informativeness of these eight genes was superior to that of any individual gene. Individually, *ATM*, *NOTCH1* and *SF3B1* were the most informative. C‐Harrel concordance index of these three genes together was 0.412, close to that of the eight genes. By contrast, C‐Harrel concordance index of *TP53* was weak, in accordance with the fact that *TP53* mutations mainly predict resistance to Fludarabine.

As shown in Figure S9, any mutation in genes of this panel deeply influenced the survival rate of Binet stage A or B patients, without effect of the number of accumulated mutations. By contrast, mutations in the remaining genes other than the eight selected had no impact on TFS (Figure S10). This panel of eight genes was then referred to as the eight gene estimator.

### 3.4 Relationship between TMB and the eight gene estimator with the CLL short‐term progressiveness

With a threshold of at least one mutation, we compared the C‐Harrel concordance index of the eight gene estimator to that of the main cytogenetic abnormalities and *IGHV* mutational status. Among poor prognosis criteria, Binet stage B and unmutated *IGHV* genes had the most informative C‐Harrel concordance index followed by the eight gene estimator (Table S5). However, the eight gene estimator had the best C‐Harrel concordance index for patients with a TFS of at least 6 months while the Binet stage was in eighth position (Table 1). In this analysis, the weight of Binet stage on TFS is artefactually overestimated since it is the main decision criteria to treat patients. Therefore, this result underlines the importance of genetics in predicting the prognosis of patients with an apparently non‐progressive disease.

**TABLE 1:** Impact of the main genetic markers on TFS among 80 Binet stage A and B patients with a TFS over 6 months: number of cases, C‐Harrel concordance index (C‐index), and logrank test *P*‐value are given for each parameter

Criteria	Number of events	C‐index	logrank test, *P*‐value
≥ 1 mutation in the eight gene estimator	46	0.353	6.10^−4^
Unmutated *IGHV*	35	0.362	2.10^−3^
*SF3B1* mutation	20	0.391	1.10^−3^
*ATM* mutation	21	0.418	0.014
*NOTCH1* mutation	14	0.450	0.007
Trisomy 12	9	0.454	0.056
Binet stage B	10	0.462	0.038
*BIRC3* mutation	11	0.465	0.10
Complex karyotype	10	0.471	0.11
*XPO1* mutation	3	0.478	0.065
*TNFAIP3* mutation	2	0.481	0.077
del(17p)	3	0.481	0.059
*MYD88* mutation	4	0.484	0.21
del(11q)	5	0.487	0.39
*TP53* mutation	9	0.496	0.52
Isolated del(13q)	22	0.524	0.18
Normal karyotype	29	0.541	0.38
Isolated del(13q) or normal karyotype	51	0.568	0.015

### 3.5 Relationship between TMB and the eight gene estimator with the CLL mid‐term progressiveness

**FIGURE 1:**
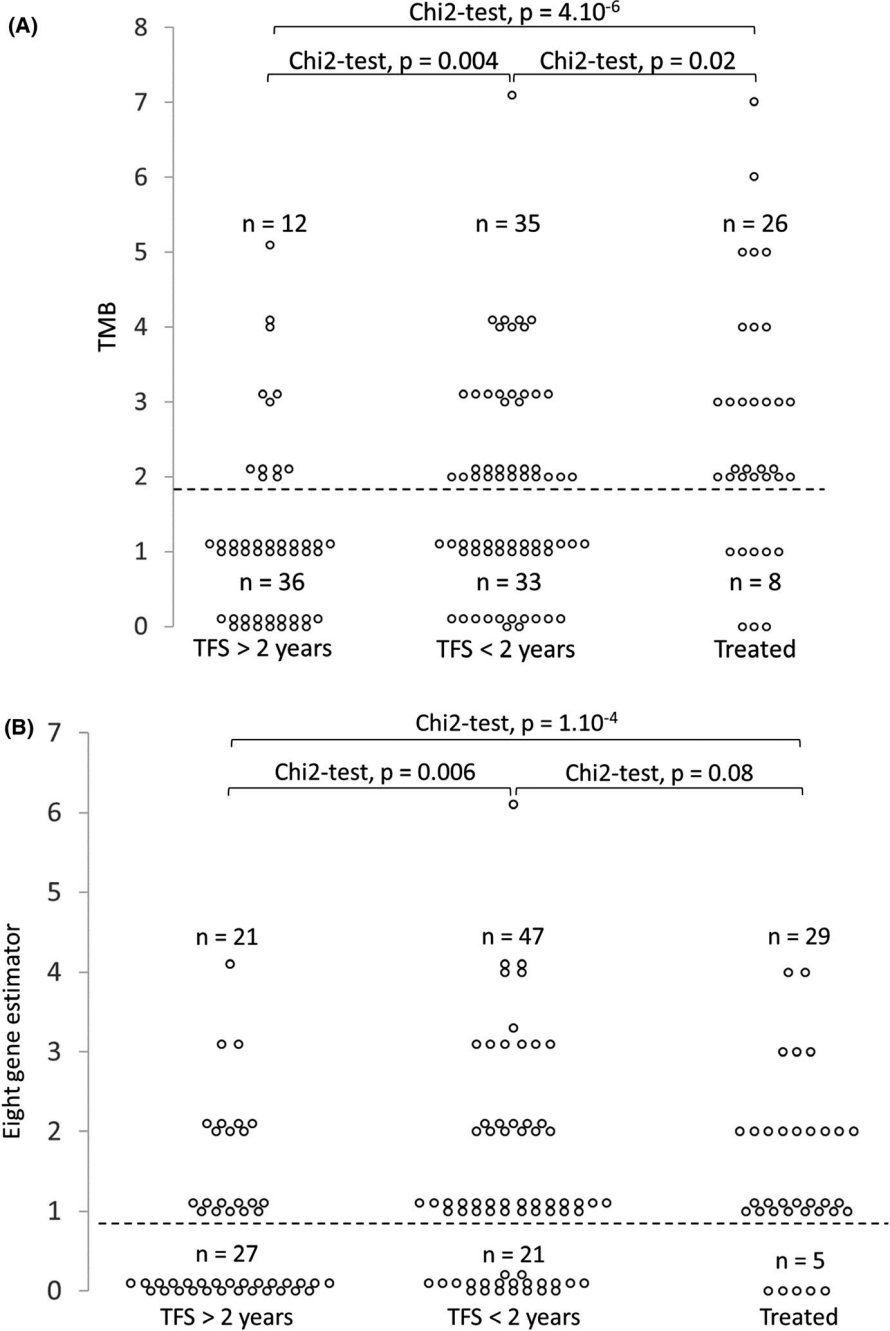
Relationship between TFS and TMB (A) or mutations among the eight gene estimator (B). Number of cases with a TMB < or ≥2 (A) or with mutations < or ≥1 among the eight gene estimator (B) are indicated. The chi‐square test *P*‐values are given

Even if nearly impossible to estimate, patients seen in specialized hospital centers are very heterogeneous in terms of the duration disease progression (or silent history of their cancer) at time of diagnosis and this should influence the TFS. Therefore, we separated the entire series of untreated patients into two categories: those that had a TFS <2 years and the others (short and long TFS, respectively). As shown in Figure 1A, TMB estimated from the whole panel of genes was significantly higher in both previously treated patients and untreated patients with a short TFS when compared to those with long TFS (Chi^2^‐test, *P* = 4.10^−6^ and 0.004, respectively). TMB was greater in previously treated patients than in untreated patients with a short TFS. As from the whole gene panel, TMB from the eight gene estimator was decreased in patients with long TFS and was higher in previously treated patients when compared to those with a short TFS (Figure 1B).

These results clearly indicate that, with Binet stage and *IGHV* mutational status, TMB estimated from either the whole panel or the eight gene estimator may identify patients at diagnosis who will need to be rapidly treated despite a clinically non‐progressive disease.

### 3.6 Relationship between TMB and the eight gene estimator with cytogenetics and IGHV mutational status

**FIGURE 2:**
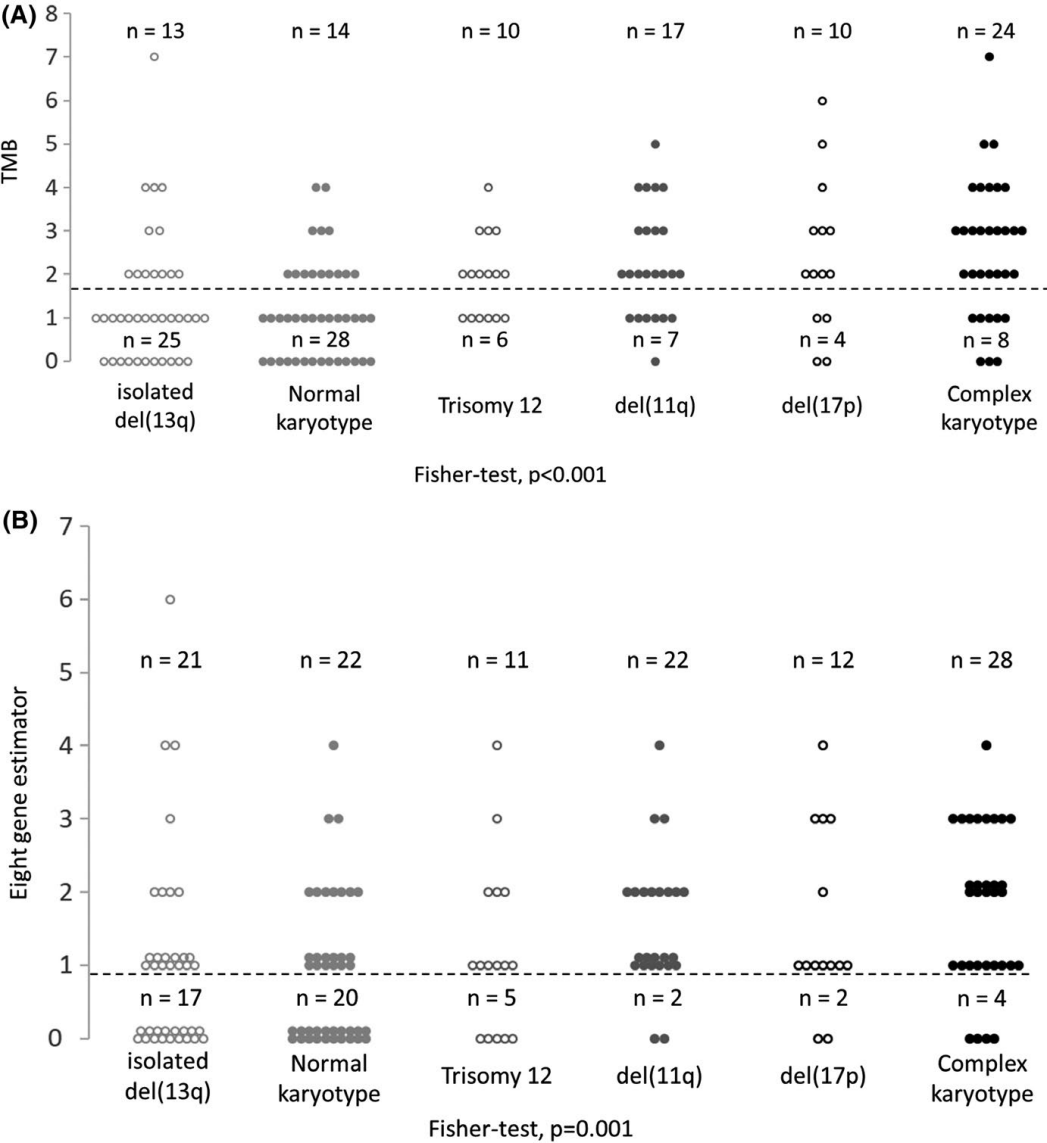
Relationship between the main cytogenetic categories and TMB from the entire gene panel (A) or from the eight gene estimator (B). Number of cases with a TMB < or ≥2 (A) or with mutations < or ≥1 among the eight gene estimator (B) are indicated. The Fisher's exact test *P*‐values are given

**FIGURE 3:**
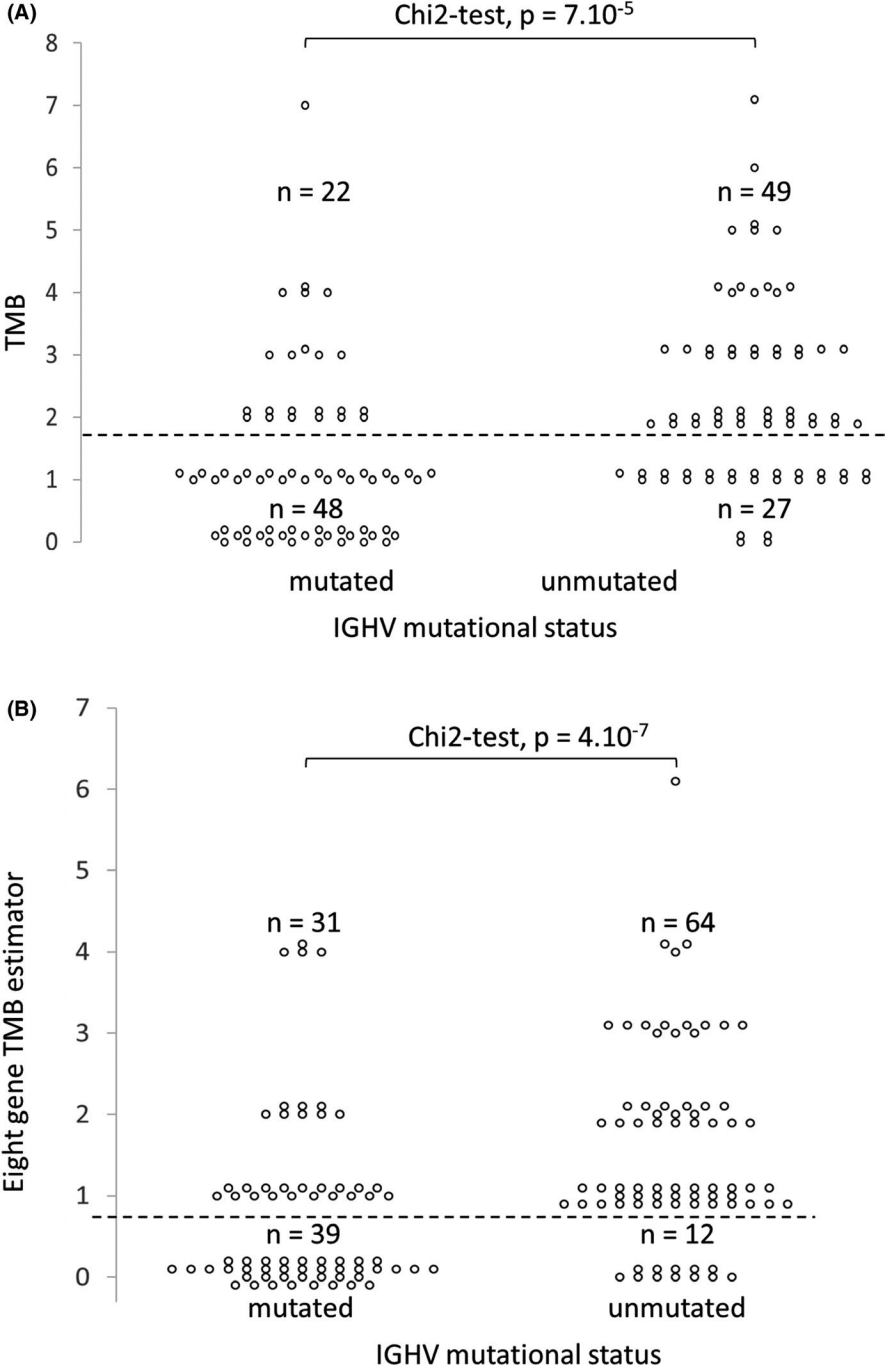
Relationship between *IGHV* mutational status and TMB from the entire gene panel (A) or from the eight genes estimator (B). Number of cases with a TMB < or ≥2 (A) or with mutations < or ≥1 among the eight gene estimator (B) are indicated. The chi‐square test *P*‐values are given

We also raised the question of the relationships between TMB and cytogenetics combined with *IGHV* mutational status. TMB evaluated from the whole panel or from the eight gene estimator was significantly higher in high risk (del(11q), del(17p) or complex karyotype) than in low risk (isolated del(13q) or normal karyotype) cytogenetic categories and was strongly associated with cytogenetic complexity (Figure 2). TMB from the entire panel was also higher among UM‐CLL (Figure 3A, Chi‐square test, *P* = 7.10^−5^). The same result was observed for the eight gene estimator (Figure 3B, Chi‐square test, *P* = 4.10^−7^).

This analysis shows that the whole gene panel and the eight gene estimator gave similar results, emphasizing a strong relationship between TMB and CLL progression, as well as with poor prognosis cytogenetic abnormalities or with unmutated *IGHV* genes.

### 3.7 The eight gene estimator harbors all the TFS informativeness of TMB for patients with isolated del(13q) or Binet stage A CLL

**FIGURE 4:**
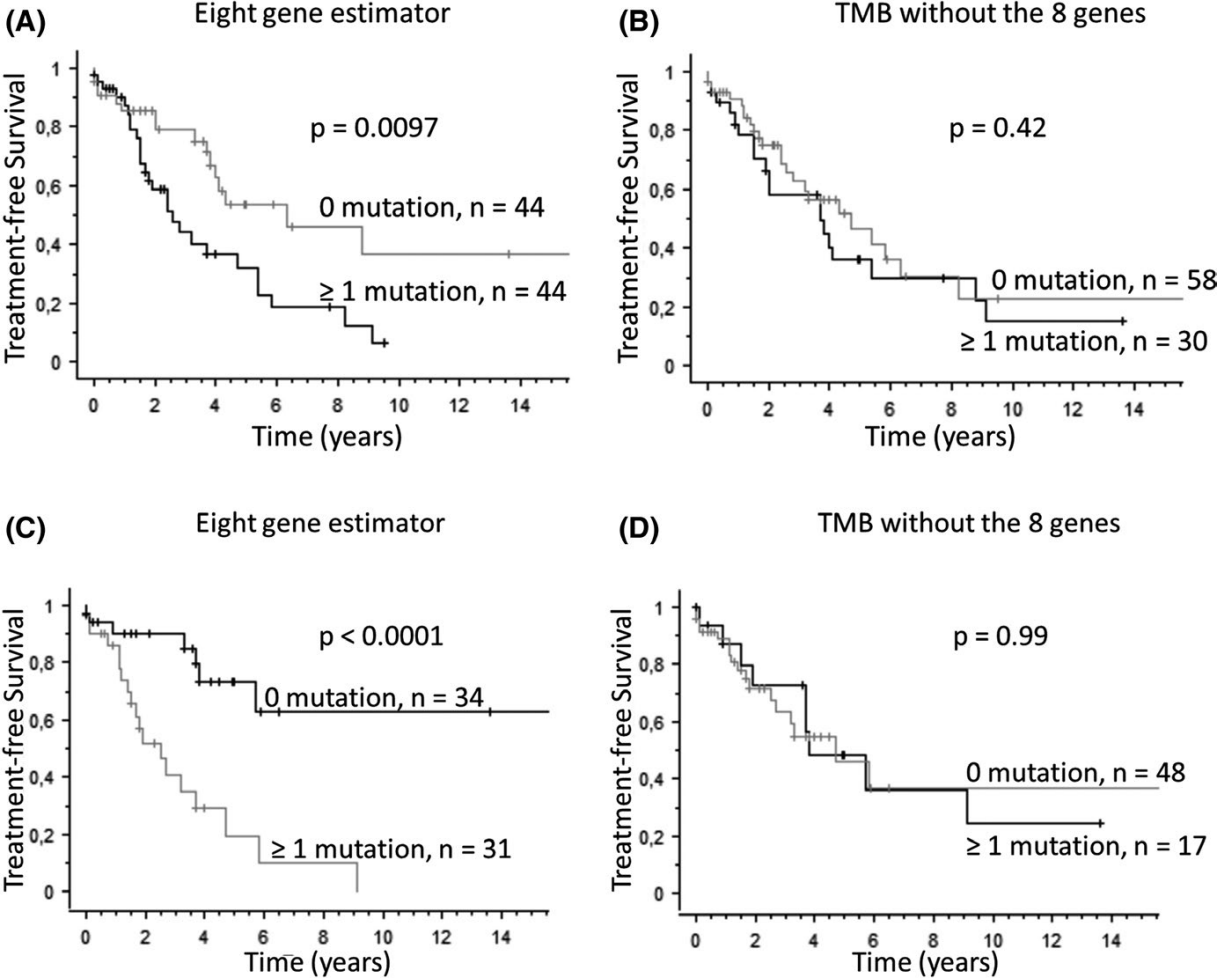
Treatment‐free survival (TFS) according to TMB from the eight gene estimator for Binet stage A patients (A) or patients with a normal karyotype or isolated del(13q) (B). Kaplan‐Meier survival curves for the eight gene estimator or for the whole gene panel minus the eight gene estimator are presented on the left and right panels, respectively. The mutation threshold was 1. Number of patients and *P*‐value of the logrank test are given

To further evaluate the interest of the eight gene estimator, the impact of TMB on patients with Binet stage A CLL or with a normal karyotype or isolated del(13q) was studied (Figure 4). TFS was significantly shorter for patients who were mutated for the eight gene estimator (Figure 4A, Binet stage A, logrank test, *P* = .0097; Figure 4B, isolated del(13q) or normal karyotype, logrank test, *P* < .0001). By contrast, mutations not included in the eight gene estimator had absolutely no effect on TFS. This indicates that TMB given by the eight gene estimator could help to predict evolution of patients in Binet stage A or with good prognosis cytogenetics.

### 3.8 Univariate and multivariate survival analysis of TMB and the eight gene estimator among Binet stage A patients

A Cox univariate model was constructed on untreated Binet stage A untreated patients with the whole TMB and the eight gene estimator together with the *IGHV* mutational status, mutations in each of the eight genes and the main cytogenetic abnormalities (Table S6). The most significant poor prognosis variable was the eight gene estimator (hazard ratio [HR] = 3.4), before TMB from the whole panel and IGHV mutational status (HR = 2.7 and 2.3, respectively). We constructed a multivariate model that included the eight gene estimator together with the *IGHV* mutational status, isolated del(13q), trisomy 12 and del(11q) (See methods for selection of parameters). Only the eight gene estimator was found to be independent according to this model (*P* = .001), showing that this estimator alone brought a new independent global information.

## 4 DISCUSSION

Whole exome and whole genome sequencing are not yet routinely available in clinical practice, due to high cost and time‐consuming analysis. However, it has been shown that accumulation of driver mutations in tumor cells predicts prognosis in CLL patients. Some authors proposed large resequencing panels to estimate this genomic complexity and to predict prognosis. For example, Nadeu et al^17^ sequenced a series of 28 driver genes and showed that accumulation of driver alterations (mutational complexity) is associated with a shorter TFS independently of the Binet Stage and *IGHV* mutational status. Kleinstern et al^18^ presented the results on a tumor mutational load that was estimated from resequencing a panel of 60 genes. However, resequencing numerous genes is costly, often requires complex bioinformatic analysis and is not well adapted to serial production and reporting results in the routine practice of patient care. Moreover, according to current techniques, it can be far more simple, quicker, and cheaper to resequence a “few well chosen” genes in depth rather than large panels.

Here, we show that an eight gene TMB estimator gave similar prognostic information when compared to the whole TMB evaluated from a large series of resequenced genes in CLL. Any mutation within the eight gene estimator was associated with shorter TFS. This was also true for Binet stage A patients or for patients with good prognosis karyotypes. Being an independent prognostic marker, the significance of the eight gene estimator was superior to any of the other genetic or cytogenetic parameters after multivariate analysis. Moreover, mutations in the eight gene estimator were strongly associated with UM‐CLLs and with poor prognosis cytogenetic abnormalities.

Apart from *TNFAIP3*, genes of the eight gene estimator are all well known to be recurrently mutated in CLL. As cited above, the mutational status of *TP53* is required to predict the response to Fludarabine.^28^ Mutations in *SF3B1* have been recognized as a marker associated with chemotherapy resistance in patients with wild type *TP53*.^29^ With the *SF3B1*‐K700E mutation, *ATM* deletion leads to a CLL‐like disease in mouse.^30^ *XPO1* mutations are correlated with those of *SF3B1*.^31^ Even if the relationship between *ATM* mutations and function is not strong, the residual *ATM* allele is frequently mutated in patients with *ATM* deletion and is associated with poorer response to chemotherapy.^32^, ^33^ Being part of the integrated mutational and cytogenetic score of Rossi et al,^10^ *NOTCH1* and *BIRC3* alterations are poor prognosis genetic markers. Clonal and subclonal *NOTCH1* mutations are associated with trisomy 12^34^ and predict shorter TFS.^35^ *MYD88* is one of the genes whose mutations drive clonal or subclonal evolution of CLL.^14^ Mutations of *BICR3* and *TNFAIP3* are associated with NF‐κB activation.^36^, ^37^, ^38^ Meanwhile, *BIRC3* mutations are specifically found in CLL and marginal zone lymphoma of the spleen.^38^, ^39^ Inactivating mutations of *TNFAIP3* have been reported in MALT and diffuse large B‐cell lymphomas^36^, ^37^ and are infrequent in CLL.^40^ Here, three untreated CLL patients (2.6%) harbored mutations of *TNFAIP3*. TFS was 0.1, 1.4, and 3.2 years. The two former cases also exhibited mutations of *SF3B1* and *ATM*. This confirms that *TNFAIP3* mutations are indeed infrequent in CLL, being in fact part of the genetic complexity.

Interestingly, seven genes of the eight gene estimator, *TP53*, *SF3B1*, *ATM*, *NOTCH1*, *XPO1*, *BIRC3*, and *MYD88* are part of the nine genes used by Guièze et al^41^ to show that a multihit mutational profile is associated with a much shorter progression‐free survival in relapsed/refractory CLL. This suggests that the eight gene estimator could be also useful to predict the response to chemotherapy.

Values of the C‐Harrel concordance index revealed that the main interest of the eight gene estimator was to predict the prognosis of patients with a TFS over 6 months, *id est* of patients who did not have a rapidly progressive or active CLL and were not immediately treated. Consistently, mutations within the eight gene estimator also predicted the TFS of Binet stage A and of patients with a good prognosis karyotype. The C‐Harrel concordance index also classifies genes according to their prognostic informativeness. In that view, *SF3B1*, *ATM*, and *NOTCH1* were the three most informative genes. The C‐Harrel concordance index of these three genes together was close to that of the eight gene estimator, even if higher. Nevertheless, the C‐Harrel concordance index analysis clearly indicates that the five other genes also contributed to prognostic information. By contrast, mutations in genes other than the eight gene estimator did not improve TFS prediction.

The main limitation of this study is the number of patients. Despite this limitation, our results show that the TMB (or mutational complexity) can be evaluated from an eight gene estimator that comprises *ATM*, *SF3B1*, *NOTCH1*, *BIRC3*, *XPO1*, *MYD88*, *TNFAIP3*, and *TP53*. Any mutation in this set of genes is associated with poor prognosis cytogenetics (eg, del(11q), del(17p) and complex karyotype) as well as with unmutated *IGHV* genes. Above all, it predicted shorter TFS even in Binet stage A patients or patients with a good prognosis karyotype. High‐throughput sequencing and analysis of the mutational status of this limited set of genes is easily achievable in hospital centers where molecular diagnosis and follow‐up of CLL patients is routinely done. Whether the eight gene estimator could predict the response to chemotherapy and/or the time to next treatment is an open question that needs future prospective studies.

## CONFLICT OF INTEREST

The authors have no conflict of interest to declare.

## AUTHOR'S CONTRIBUTIONS

JC and DR performed high‐throughput sequencing and data analysis for center 1. CP and TF performed high‐throughput sequencing and data analysis for center 2. LD performed a part of high‐throughput sequencing technique. NG analyzed *IGHV* mutational status. JF supervised the statistical analysis and performed the C‐Harrel analysis.

## Supporting information

Figure S1

Figure S2

Figure S3

Figure S4

Figure S5

Figure S6

Figure S7

Figure S8

Figure S9

Figure S10

Legends

Table S1

Table S2

Table S3

Table S4

Table S5

Table S6

Supplementary Material

Supplementary Methods

## Data Availability

The data that support the findings of this study are available on request from the corresponding author. The data are not publicly available due to privacy or ethical restrictions.
