# Supplementary figures and images for: A reduced panel of eight genes (ATM, SF3B1, NOTCH1, BIRC3, XPO1, MYD88, TNFAIP3, and TP53) as an estimator of the tumor mutational burden in chronic lymphocytic leukemia
Source: Int J Lab Hematol. 2020 Dec 16;43(4):683–92. doi: 10.1111/ijlh.13435 (PMC8451785; doi:10.1111/ijlh.13435)

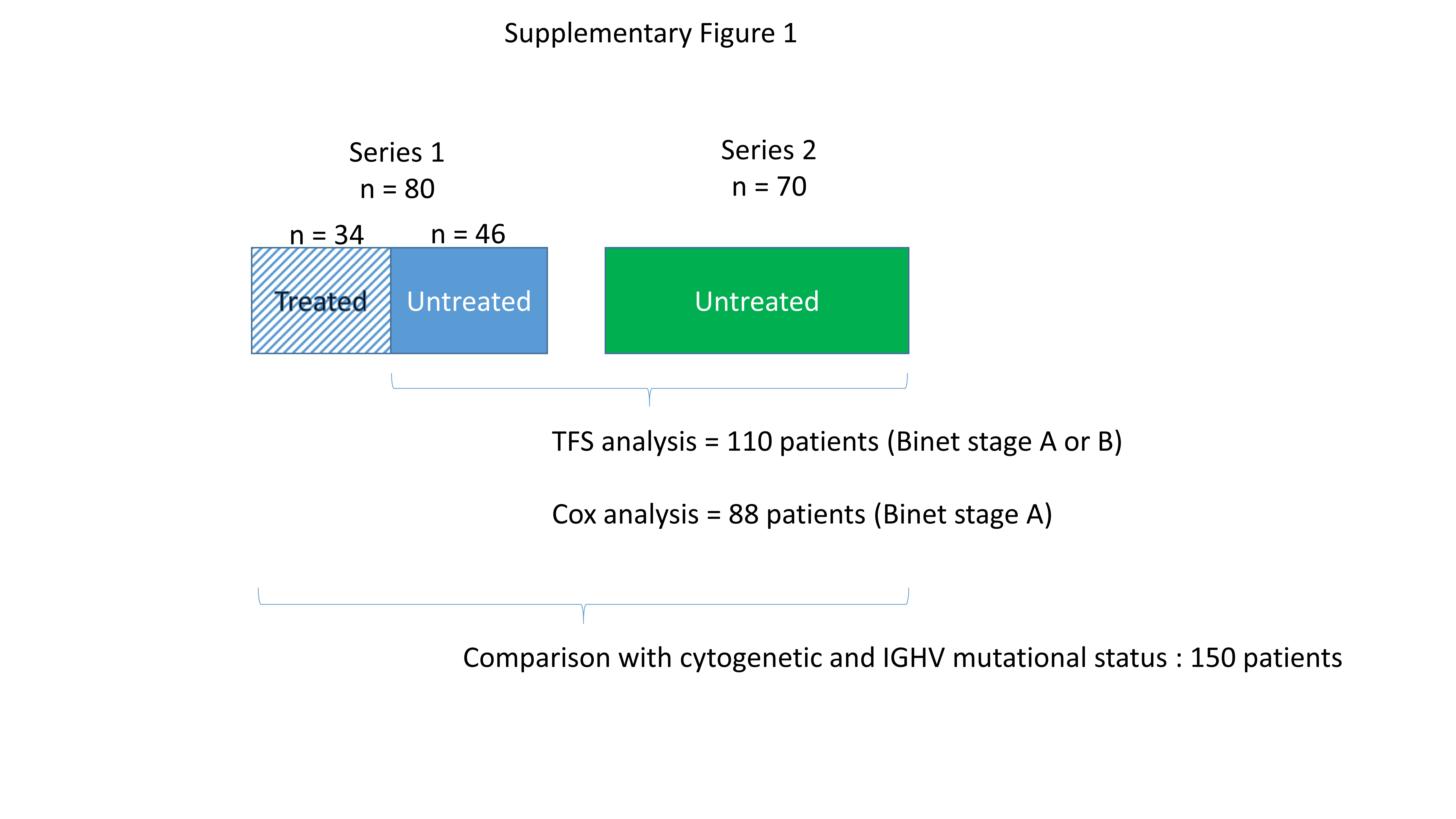

Supplement: Supplementary file 1 — Figure S1 [file IJLH-43-683-s014.JPG]

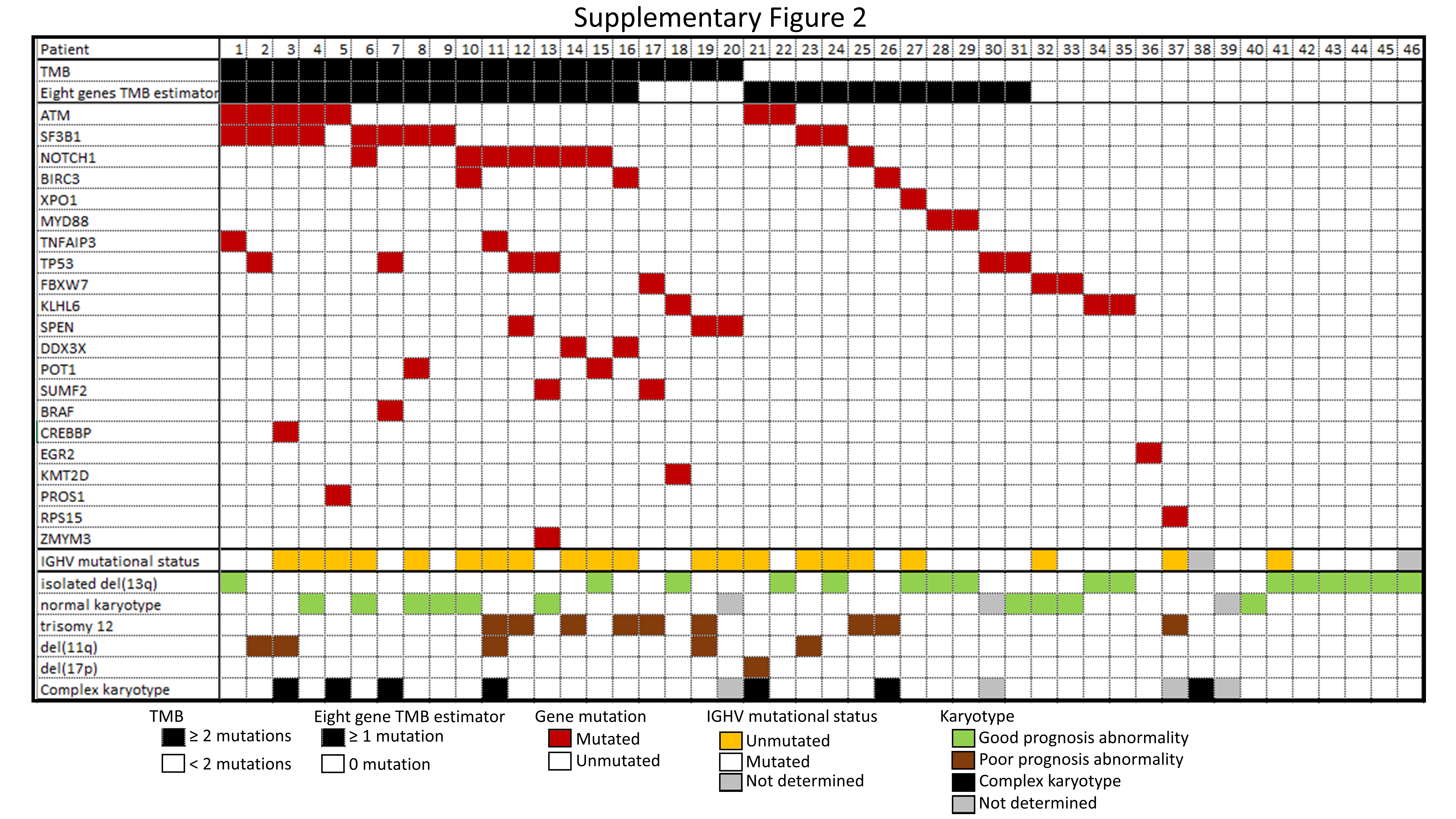

Supplement: Supplementary file 2 — Figure S2 [file IJLH-43-683-s010.JPG]

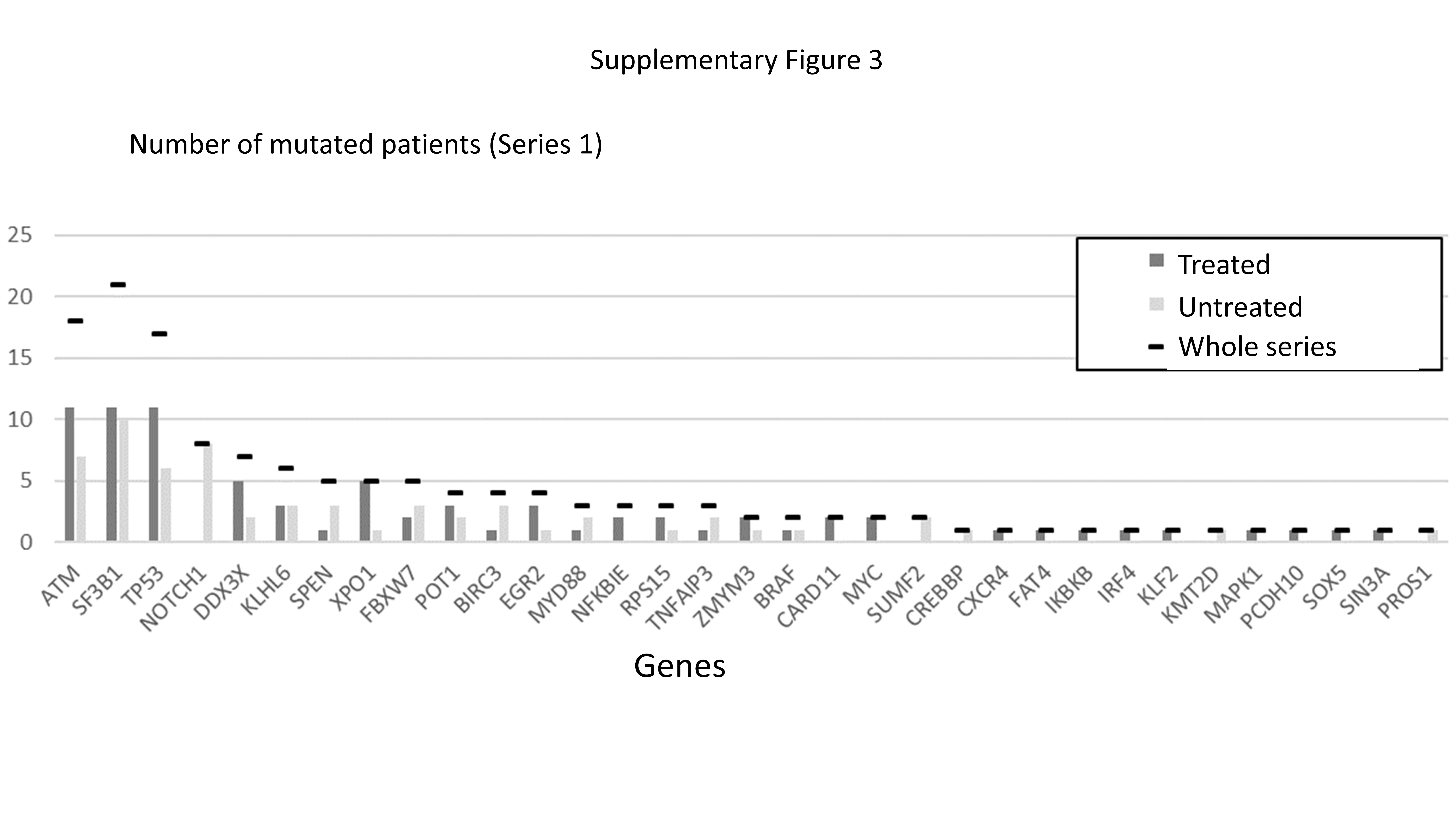

Supplement: Supplementary file 3 — Figure S3 [file IJLH-43-683-s005.JPG]

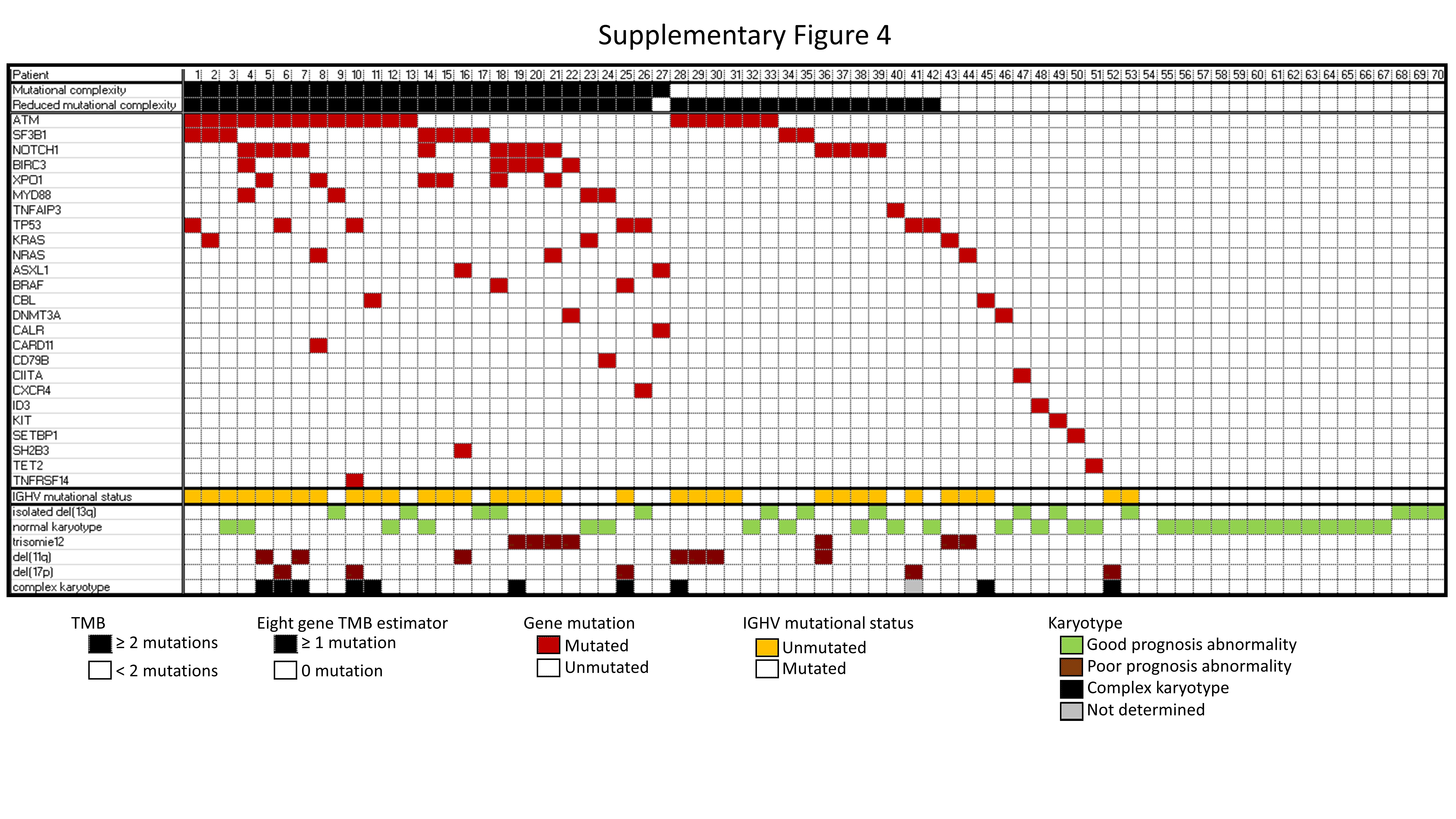

Supplement: Supplementary file 4 — Figure S4 [file IJLH-43-683-s019.JPG]

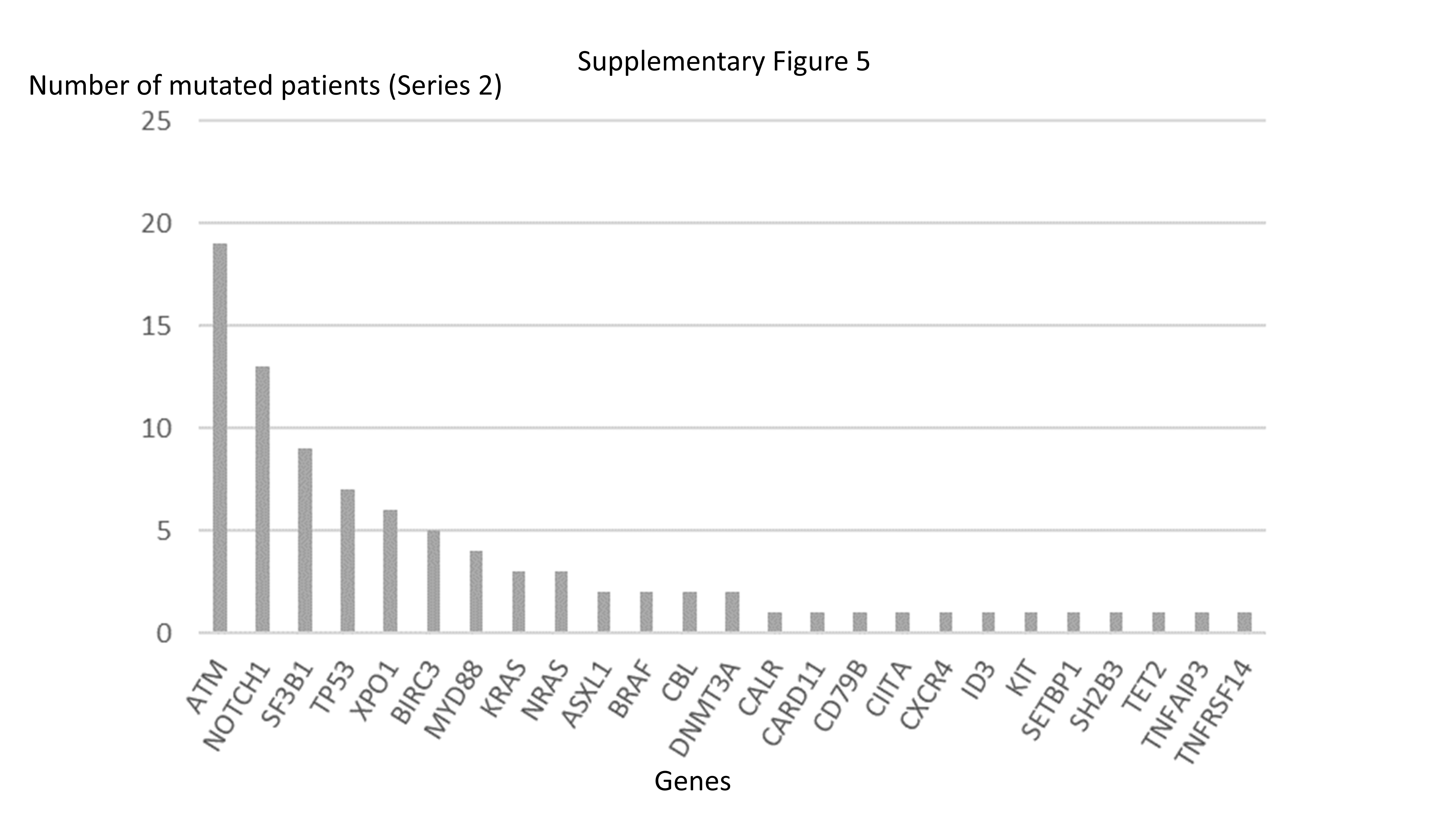

Supplement: Supplementary file 5 — Figure S5 [file IJLH-43-683-s006.JPG]

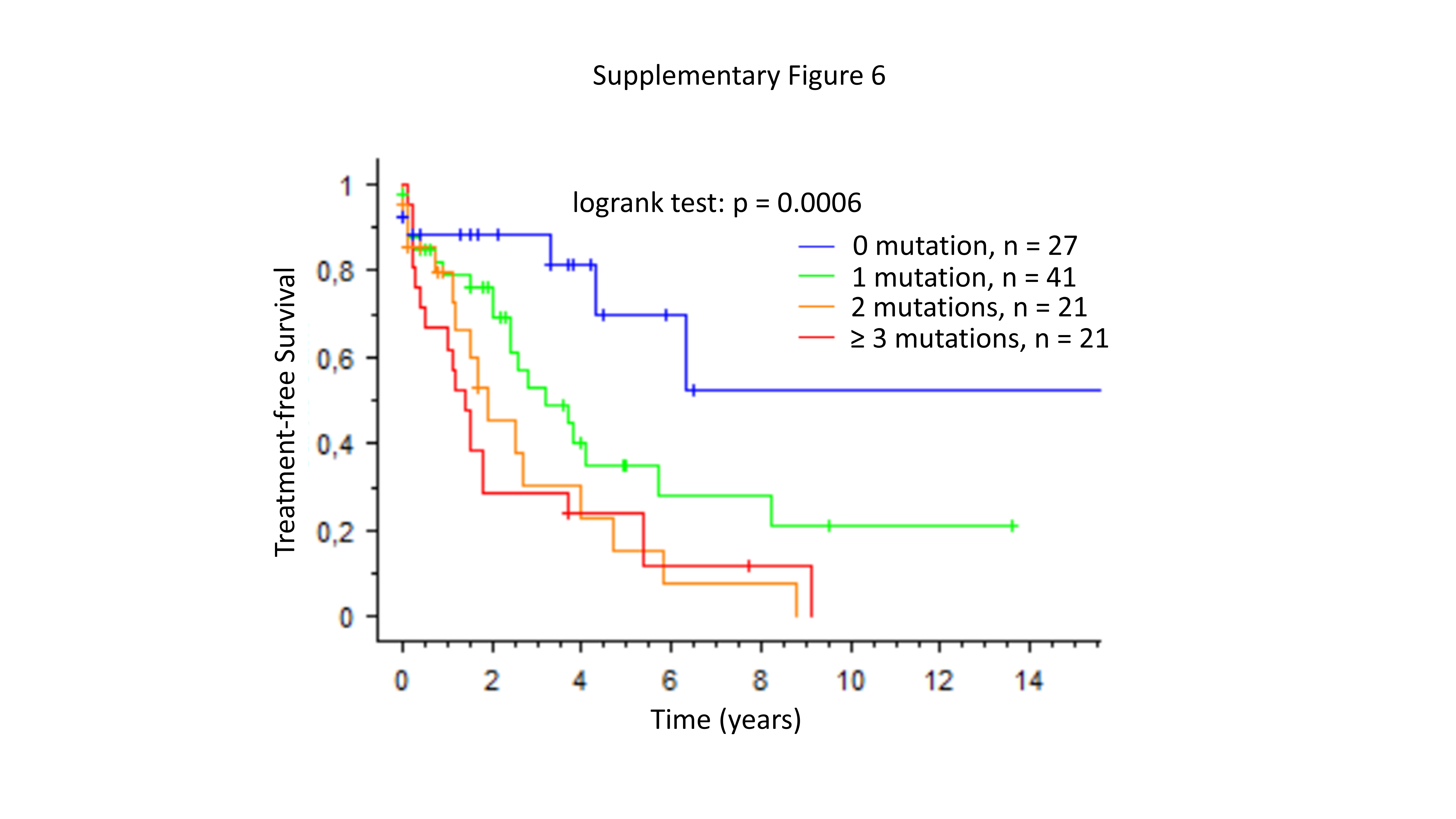

Supplement: Supplementary file 6 — Figure S6 [file IJLH-43-683-s007.JPG]

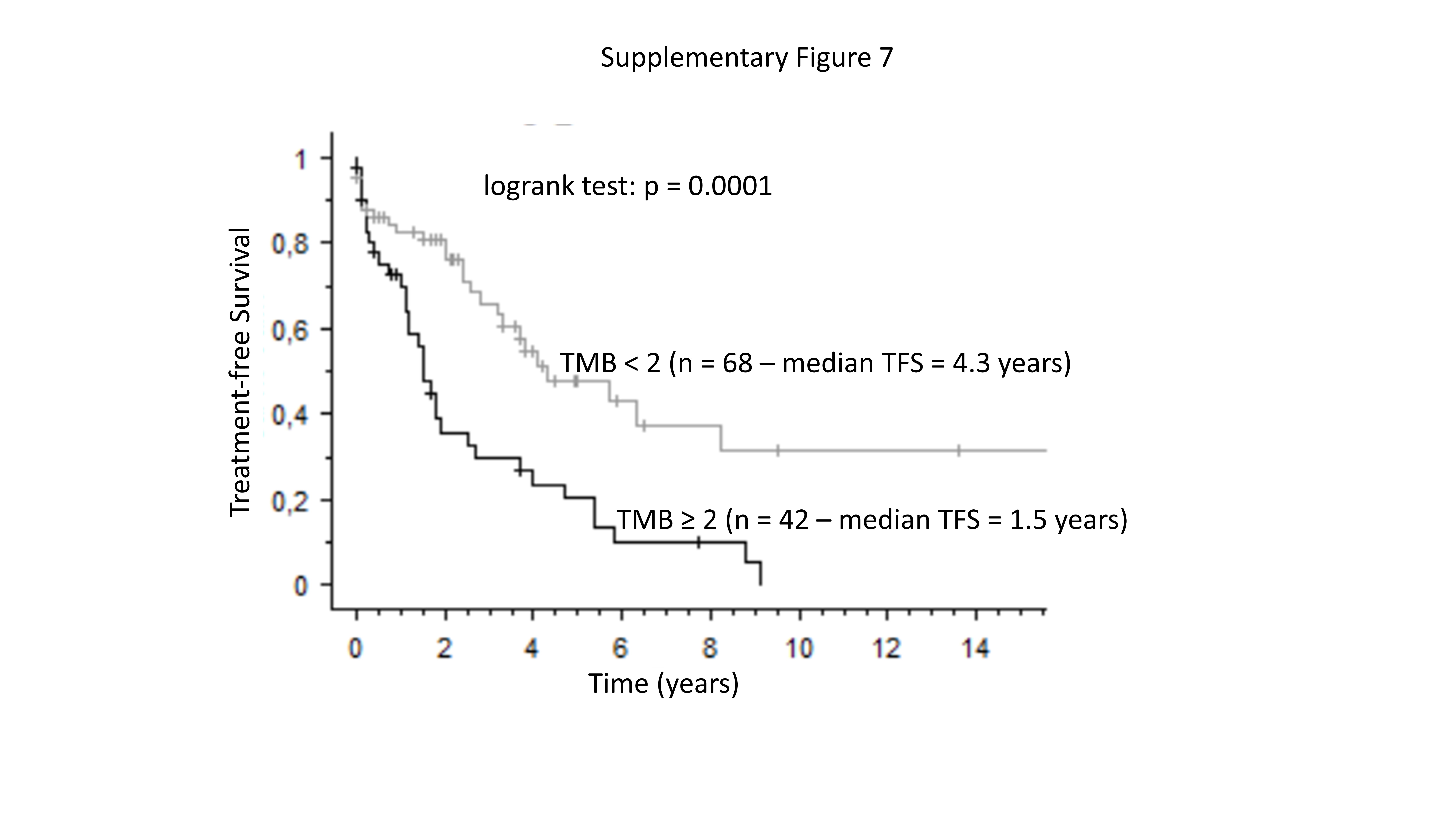

Supplement: Supplementary file 7 — Figure S7 [file IJLH-43-683-s016.JPG]

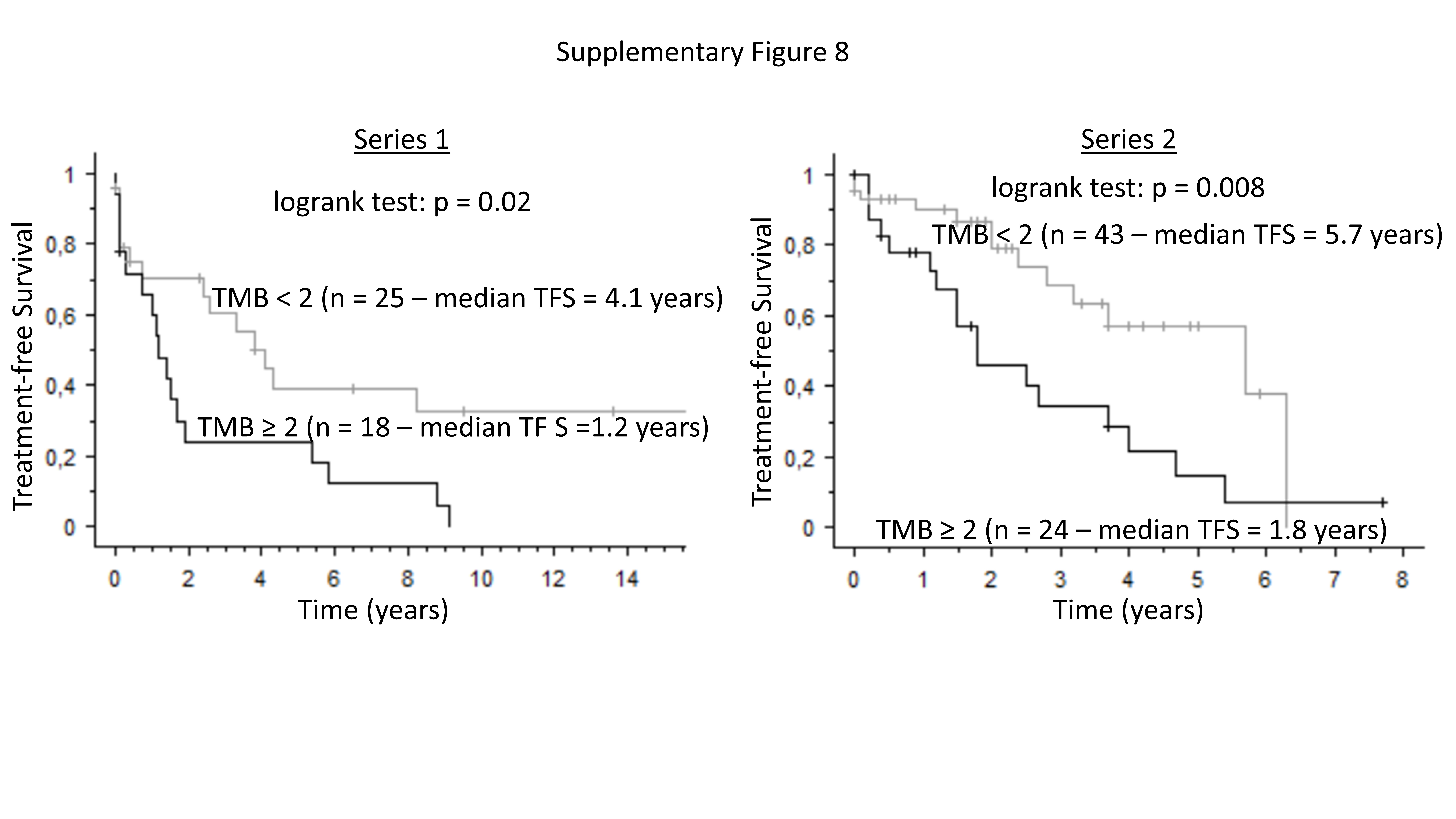

Supplement: Supplementary file 8 — Figure S8 [file IJLH-43-683-s015.JPG]

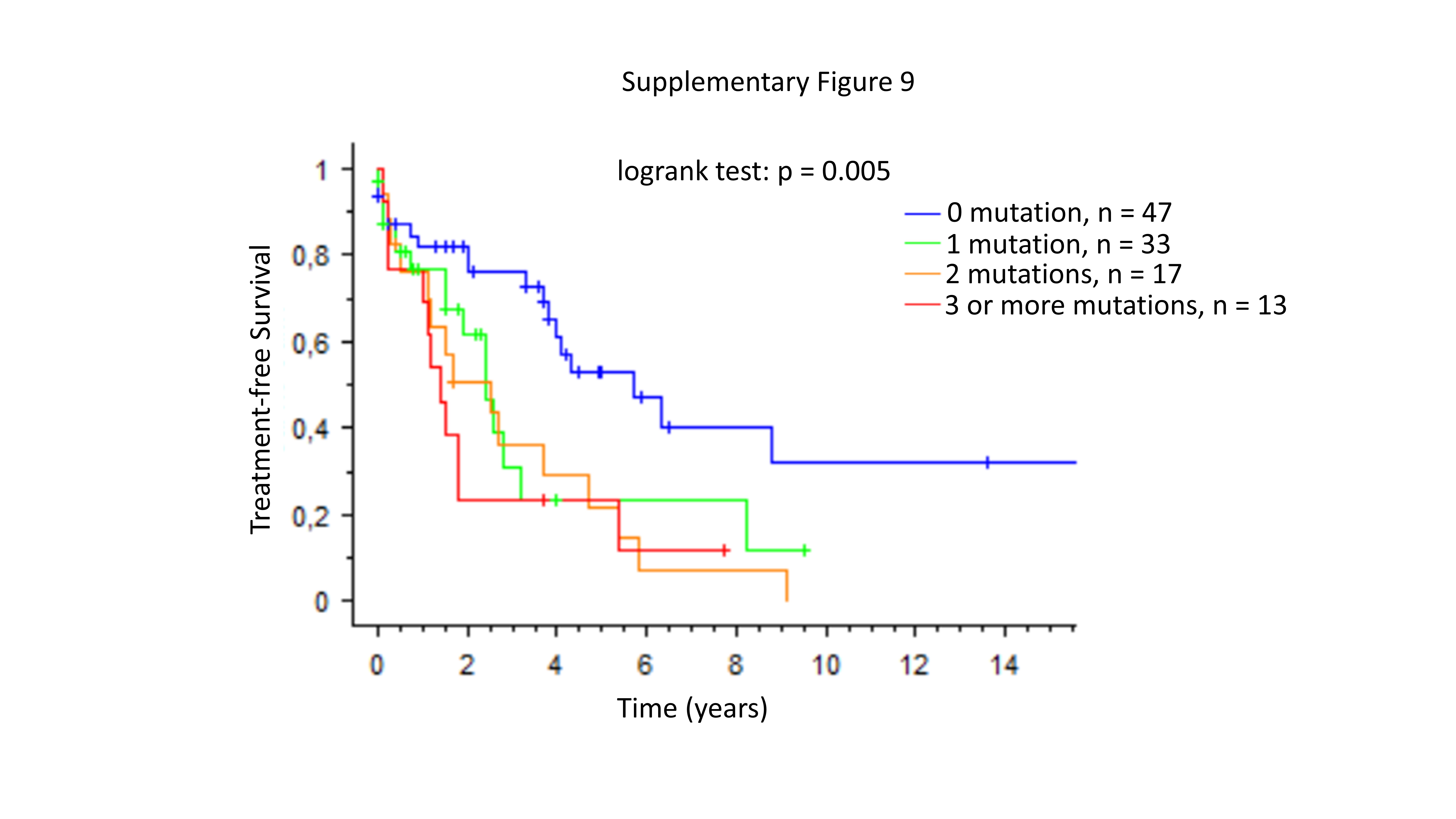

Supplement: Supplementary file 9 — Figure S9 [file IJLH-43-683-s012.JPG]

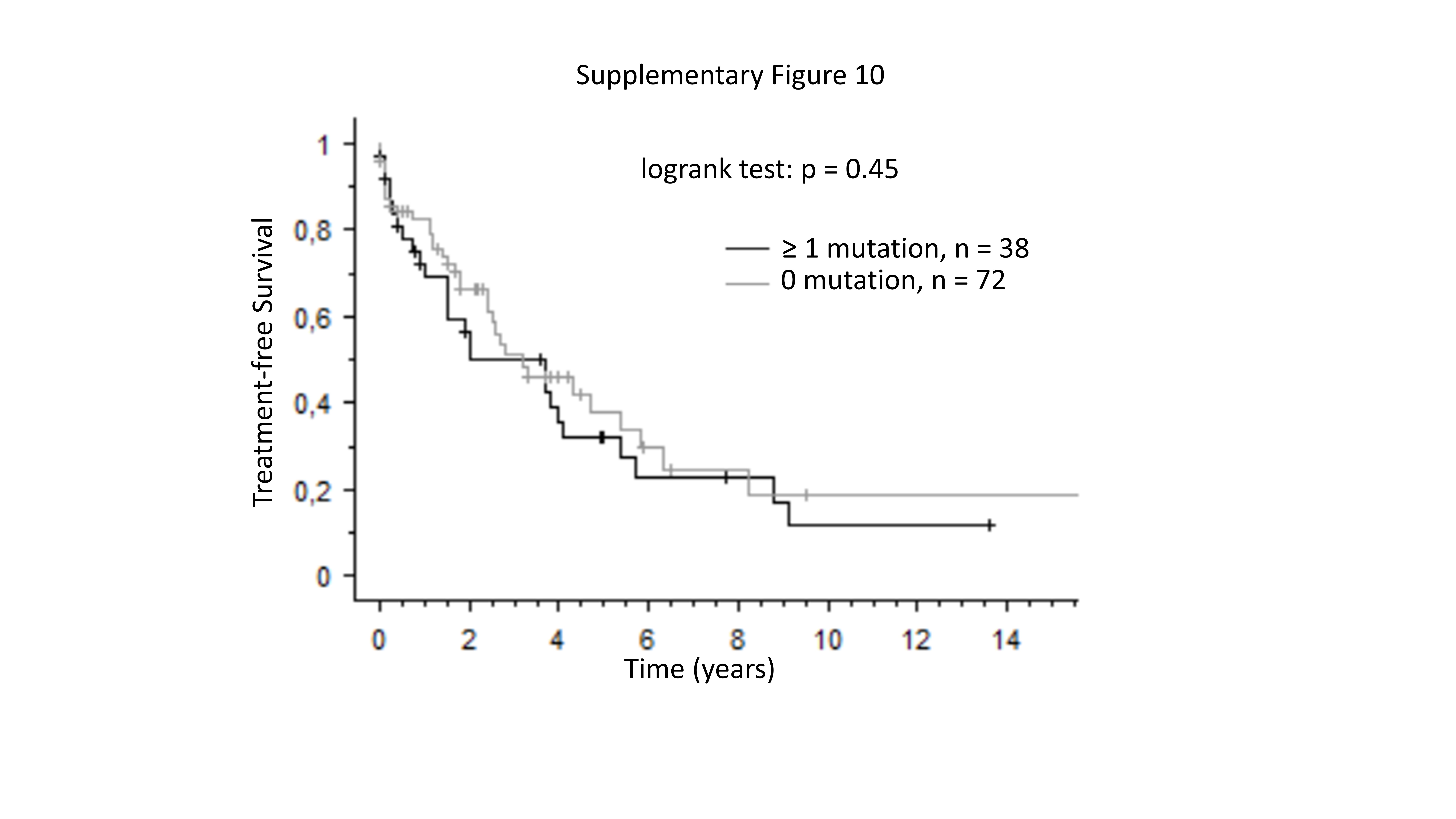

Supplement: Supplementary file 10 — Figure S10 [file IJLH-43-683-s004.JPG]
