# Supplementary material for: A reduced panel of eight genes (ATM, SF3B1, NOTCH1, BIRC3, XPO1, MYD88, TNFAIP3, and TP53) as an estimator of the tumor mutational burden in chronic lymphocytic leukemia
Source: Int J Lab Hematol. 2020 Dec 16;43(4):683–92. doi: 10.1111/ijlh.13435 (PMC8451785; doi:10.1111/ijlh.13435)
Supplement: Supplementary file 11 — Legends [file IJLH-43-683-s011.docx]

**Supplementary Figure 1: Flow chart of the study**

**Supplementary Figure 2: Mutational landscape and cytogenetic characteristics of untreated patients of series 1 at diagnosis**

**Supplementary Figure 3: Number of patients presenting a mutation per gene across series 1**

Dark gray bars indicate previously treated patients. Light gray bars indicate untreated patients. The black line indicates the whole series 1.

**Supplementary Figure 4: Mutational landscape and cytogenetic characteristics of patients of series 2 at diagnosis**

**Supplementary Figure 5: Number of patients presenting a mutation per gene across validation series**

**Supplementary Figure 6: Treatment-free survival (TFS) according to the number of mutations among Binet stage A and B CLL patients at diagnosis**

Number of patients and p-value of the logrank test are given.

**Supplementary Figure 7: Treatment-free survival (TFS) according to TMB at diagnosis among Binet stage A and B patients (n = 110)**

The TMB threshold was 2. Number of patients, median TFS and p-value of the logrank test are given

**Supplementary Figure 8: Treatment-free survival (TFS) according to TMB among Binet stage A and B CLL patients at diagnosis for series 1 (Supplementary Figure 7A) and series 2 (Supplementary Figure 7B)**

The TMB threshold was 2. Number of patients, median TFS and p-values of the logrank test are given.

**Supplementary Figure 9: Treatment-free survival (TFS) according to the number of mutations in the eight gene estimator among Binet stage A and B CLL patients at diagnosis**

Number of patients and p-value of the logrank test are given.

**Supplementary Figure 10: Treatment-free survival (TFS) according to TMB without mutations of the eight gene estimator among Binet stage A and B CLL patients at diagnosis**

The mutation threshold was 1. Number of patients and p-values of the logrank test are given.
