# Supplementary material for: A reduced panel of eight genes (ATM, SF3B1, NOTCH1, BIRC3, XPO1, MYD88, TNFAIP3, and TP53) as an estimator of the tumor mutational burden in chronic lymphocytic leukemia
Source: Int J Lab Hematol. 2020 Dec 16;43(4):683–92. doi: 10.1111/ijlh.13435 (PMC8451785; doi:10.1111/ijlh.13435)
Supplement: Supplementary file 12 — Table S1 [file IJLH-43-683-s003.docx]

| **Supplementary Table 1: Panel of genes used in series 1** | | |  |  |
| --- | --- | --- | --- | --- |
| Chromosome | Gene | Region | Reference | bold: genes in common between  the training and the validation center |
| Chr1 | SPEN | E11 | NM_015055 |  |
| **Chr1** | **ARID1A** | **from E1 to 20** | **NM_006015** |  |
| **Chr1** | **NOTCH2** | **from E26 to 28 + E34** | **NM_024408** |  |
| Chr2 | REL | from E1 to E10 (with UTR) | NM_001291746 |  |
| **Chr2** | **XPO1** | **E14, E15** | **NM_003400** |  |
| **Chr2** | **CXCR4** | **E1, E2** | **NM_003467** |  |
| **Chr2** | **SF3B1** | **from E1 to E25** | **NM_012433** |  |
| **Chr3** | **MYD88** | **from E3 to E5** | **NM_001172567** |  |
| Chr3 | EPHA3 | E1, E3, E5, E7, E9, E11, E13, E15, E17 | NM_005233 |  |
| Chr3 | PROS1 | E2, E4, E6, E8, E9, E10, E11, E13, E15 | NM_000313 |  |
| Chr3 | TBL1XR1 | E3, E6, E7, E8, E12 | NM_024665 |  |
| Chr3 | KLHL6 | E6, E7 | NM_130446 |  |
| Chr4 | ZAR1 | from E1 to E4 | NM_175619 |  |
| Chr4 | RASSF6 | from E1 to E11 | NM_201431 |  |
| Chr4 | PTPN13 | from E2 to E48 | NM_080685 |  |
| Chr4 | FAT4 | E1 | NM_024582 |  |
| Chr4 | PCDH10 | E1 | NM_032961 |  |
| Chr4 | FBXW7 | from E2 to E12 | NM_033632 |  |
| Chr5 | EGR1 | E1, E2 | NM_001964 |  |
| **Chr6** | **IRF4** | **from E2 to E9** | **NM_002460** |  |
| Chr6 | NFKBIE | from E1 to E6 | NM_004556 |  |
| **Chr6** | **PRDM1** | **from E1 to E7** | **NM_001198** |  |
| **Chr6** | **TNFAIP3** | **from E2 to E9** | **NM_006290** |  |
| **Chr7** | **CARD11** | **from E2 to E9** | **NM_032415** |  |
| Chr7 | SUMF2 | from E1 to E9 | NM_015411 |  |
| Chr7 | ASL | E1, E2, E4, E6, E8, E10, E12,E14, E15, E16, E17 | NM_000048 |  |
| Chr7 | POT1 | from E5 to E19 | NM_015450 |  |
| **Chr7** | **BRAF** | **E11, E15** | **NM_004333** |  |
| **Chr7** | **EZH2** | **E16, E18** | **NM_004456** |  |
| Chr8 | IKBKB | from E2 to E22 | NM_001556 |  |
| Chr8 | EIF3H | from E1 to E8 | NM_003756 |  |
| **Chr8** | **MYC** | **from E1 to E3** | **NM_002467** |  |
| Chr9 | JAK2 | E7 | NM_004972 |  |
| **Chr9** | **NOTCH1** | **from E25 to E28 + E34** | **NM_017617** |  |
| Chr10 | EGR2 | E3, E4 | NM_001136178 |  |
| Chr11 | SWAP70 | from E1 to E3 | NM_015055 |  |
| **Chr11** | **BIRC3** | **from E6 to E9** | **NM_001165** |  |
| **Chr11** | **ATM** | **from E1 to E62** | **NM_000051** |  |
| Chr12 | SOX5 | from E1 to E15 | NM_006940 |  |
| Chr12 | KMT2D | from E1 to E54 | NM_003482 |  |
| Chr12 | POU6F1 | from E2 to E6 | NM_002702 |  |
| Chr12 | MDM2 | E1, E3, E5, E7, E9, E11 | NM_002392 |  |
| Chr12 | DTX1 | E2 | NM_004416 |  |
| Chr13 | RB1 | E2, E4, E6, E8, E10, E12, E14, E16, E18, E20, E22, E24, E26 | NM_000321 |  |
| Chr13 | DLEU2 | E1, E2, E4, E5, E7, E9, E10 |  |  |
| Chr13 | Mir16-1 | E1 |  |  |
| Chr13 | Mir15A | E1 |  |  |
| Chr13 | DLEU1 | from E1 to E6 |  |  |
| Chr14 | TRAF3 | E3, E9, E10, E12 | NM_145725 |  |
| Chr15 | SIN3A | E4, E8 | NM_001145357 |  |
| **Chr16** | **CREBBP** | **from E1 to E31** | **NM_004380** |  |
| **Chr16** | **PLCG2** | **from E18 to E23** | **NM_002661** |  |
| Chr17 | ATP2A3 | E2, E5, E8, E11, E14, E17, E20, E23 | NM_174953 |  |
| **Chr17** | **TP53** | **from E2 to E11** | **NM_000546** |  |
| Chr17 | MAP3K14 | E12 | NM_003954 |  |
| **Chr17** | **CD79B** | **from E1 to E6** | **NM_001039933** |  |
| Chr18 | MYOM1 | E2, E6, E8, E10, E12, E14, E16, E20, E22, E24, E26, E28, E30, E32, E34, E36, E38 | NM_003803 |  |
| Chr18 | DCC | E1, E3, E5, E7, E9, E11, E13, E15, E17, E19, E21, E23, E25, E29 | NM_005215 |  |
| **Chr18** | **BCL2** | **E2, E3** | **NM_000633** |  |
| Chr18 | RPS15 | from E1 to E4 | NM_001018 |  |
| Chr19 | CACNA1A | E1, E4, E7, E10, E13, E16, E19, E41, E44, E47 | NM_000068 |  |
| Chr19 | KLF2 | from E1 to E3 | NM_016270 |  |
| Chr19 | CCNE1 | from E1 to E11 | NM_001238 |  |
| **Chr19** | **CD79A** | **from E1 to E5** | **NM_001783** |  |
| Chr22 | CECR1 | E2 | NM_001282228 |  |
| Chr22 | MAPK1 | from E1 to E8 | NM_002745 |  |
| Chr22 | XBP1 | from E1 to E5 | NM_005080 |  |
| ChrX | DDX3X | from E1 to E17 | NM_001356 |  |
| ChrX | ZMYM3 | from E2 to E25 | NM_001171162 |  |
| **ChrX** | **BTK** | **from E14 to E16** | **NM_000061** |  |
