# Supplementary material for: A reduced panel of eight genes (ATM, SF3B1, NOTCH1, BIRC3, XPO1, MYD88, TNFAIP3, and TP53) as an estimator of the tumor mutational burden in chronic lymphocytic leukemia
Source: Int J Lab Hematol. 2020 Dec 16;43(4):683–92. doi: 10.1111/ijlh.13435 (PMC8451785; doi:10.1111/ijlh.13435)
Supplement: Supplementary file 13 — Table S2 [file IJLH-43-683-s009.docx]

| **Supplementary Table 2: Panel of genes used in series 2** | | | |  |
| --- | --- | --- | --- | --- |
| Chromosome | Gene | Region | Reference | bold: genes in common between the training and the validation center |
| Chr1 | TNFRSF14 | from E1 to E8 | NM_003820.3 |  |
| Chr1 | ID3 | E1,E2 | NM_002167.4 |  |
| **Chr1** | **ARID1A** | **from E1 to E20** | **NM_006015.4** |  |
| Chr1 | CSF3R | E14 | NM_000760.3 |  |
| Chr1 | MPL | E10 | NM_005373.2 |  |
| Chr1 | NRAS | E2,E3 | NM_002524.3 |  |
| Chr1 | CD58 | from E1 to E6 | NM_001779.2 |  |
| **Chr1** | **NOTCH2** | **E26, E27,E28, E34** | **NM_024408.3** |  |
| Chr2 | DNMT3A | from E2 to 23 | NM_175629.2 |  |
| **Chr2** | **XPO1** | **E15** | **NM_003400.3** |  |
| **Chr2** | **CXCR4** | **E1,E2** | **NM_003467.2** |  |
| **Chr2** | **SF3B1** | **from E14 to E18** | **NM_012433.2** |  |
| Chr2 | IDH1 | E4 | NM_005896.3 |  |
| **Chr3** | **MYD88** | **from E1 to E5** | **NM_002468.4** |  |
| Chr3 | RHOA | E2 | NM_001664.2 |  |
| Chr3 | GATA2 | from E2 to E6 | NM_032638.4 |  |
| Chr3 | BCL6 | from E3 to E10 | NM_001706.4 |  |
| Chr4 | KIT | from E8 to E13,E17 | NM_000222.2 |  |
| Chr4 | TET2 | from E3 to E11 | NM_001127208.2 |  |
| Chr5 | NPM1 | E10 | NM_199185.3 |  |
| **Chr6** | **IRF4** | **from E2 to E9** | **NM_002460.3** |  |
| Chr6 | PIM1 | from E1 to E6 | NM_002648.3 |  |
| Chr6 | CCND3 | form E1 to E5 | NM_001760.4 |  |
| **Chr6** | **PRDM1** | **from E1 to E5** | **NM_001198.3** |  |
| **Chr6** | **TNFAIP3** | **from E2 to E9** | **NM_006290.3** |  |
| **Chr7** | **CARD11** | **from E4to E9** | **NM_032415.5** |  |
| **Chr7** | **BRAF** | **E15** | **NM_004333.4** |  |
| **Chr7** | **EZH2** | **from E2 to E20** | **NM_004456.4** |  |
| **Chr8** | **MYC** | **from E1 to E3** | **NM_002467.4** |  |
| Chr9 | JAK2 | E12,E14 | NM_004972.3 |  |
| Chr9 | CDKN2A | from E1 to E3 | NM_000077.4 |  |
| Chr9 | CDKN2B | E1 | NM_004936.3 |  |
| **Chr9** | **NOTCH1** | **E34** | **NM_017617.3** |  |
| **Chr11** | **BIRC3** | **from E6 to E9** | **NM_001165.4** |  |
| **Chr11** | **ATM** | **from E2 to E63** | **NM_000051.3** |  |
| Chr11 | CBL | from E1 to E16 | NM_005188.2 |  |
| Chr12 | KRAS | E2,E3 | NM_033360.2 |  |
| Chr12 | STAT6 | from E10 to E15 | NM_001178078.1 |  |
| Chr12 | SH2B3 | from E2 to E8 | NM_005475.2 |  |
| Chr13 | FLT3 | E13,E14,E15,E20 | NM_004119.2 |  |
| Chr13 | FOXO1 | E1,E2 | NM_002015.3 |  |
| Chr15 | B2M | from E1 to E3 | NM_004048.2 |  |
| Chr15 | IDH2 | E4 | NM_002168.3 |  |
| **Chr16** | **CREBBP** | **from E23 to E29** | **NM_004380.2** |  |
| Chr16 | CIITA | from E1 to 19 | NM_000246.3 |  |
| Chr16 | SOCS1 | E2 | NM_003745.1 |  |
| **Chr16** | **PLCG2** | **from E17 to E23** | **NM_002661.3** |  |
| **Chr17** | **TP53** | **from E2 to E11** | **NM_000546.4** |  |
| Chr17 | STAT5B | from E14 to E16 | NM_012448.3 |  |
| Chr17 | STAT3 | from E19 to E21 | NM_139276.2 |  |
| **Chr17** | **CD79B** | **E5,E6** | **NM_000626.3** |  |
| Chr17 | GNA13 | from E1 to E4 | NM_006572.5 |  |
| Chr17 | SRSF2 | E1 | NM_003016.4 |  |
| Chr18 | SETBP1 | E4 | NM_015559.2 |  |
| **Chr18** | **BCL2** | **E2, E3** | **NM_000633.2** |  |
| Chr19 | TCF3 | E18,E19 | NM_003200.4 |  |
| Chr19 | EPOR | E8 | NM_000121.3 |  |
| Chr19 | CALR | E9 | NM_004343.3 |  |
| Chr19 | MEF2B | from E2 to E9 | NM_001145785.1 |  |
| **Chr19** | **CD79A** | **E4,E5** | **NM_001783.3** |  |
| Chr20 | ASXL1 | E12 | NM_015338.5 |  |
| Chr21 | RUNX1 | from E2 to E9 | NM_001754.4 |  |
| Chr21 | U2AF1 | E2,E6 | NM_006758.2 |  |
| Chr22 | EP300 | from E24 to E30 | NM_001429.3 |  |
| **ChrX** | **BTK** | **E15** | **NM_000061.2** |  |
