# Supplementary material for: A reduced panel of eight genes (ATM, SF3B1, NOTCH1, BIRC3, XPO1, MYD88, TNFAIP3, and TP53) as an estimator of the tumor mutational burden in chronic lymphocytic leukemia
Source: Int J Lab Hematol. 2020 Dec 16;43(4):683–92. doi: 10.1111/ijlh.13435 (PMC8451785; doi:10.1111/ijlh.13435)
Supplement: Supplementary file 14 — Table S3 [file IJLH-43-683-s018.docx]

**Supplementary Table 3: Patient characteristics at diagnosis (mean +/- standard deviation are given for age, lymphocytosis, hemoglobin and platelet counts; *: p<0.05)**

|  | | | **Series 1**  **(n=80)** | | **Series 2**  **(n=70)** |
| --- | --- | --- | --- | --- | --- |
|  | | | **Whole series** | **Untreated patients**  **(n=46)** |  |
| Sex | | | Men: 58.8% (n=47)  Women: 41.3% (n=33) | Men: 60.9% (n=28)  Women: 39.1% (n=18) | Men: 55.7% (n=39)  Women: 44.3% (n=31) |
| Age (years) | | | 65.2 +/- 10.5 | 67.4 +/- 10.7 | 64.3 +/-10.4 |
| Binet | | Stage A  Stage B  Stage C | 76.3% (n=58)  15.8% (n=12)  7.9% (n=6) | 80.0% (n=36)  15.6% (n=7)  4.4% (n=2) | 74.3% (n=52)  21.4% (n=15)  4.3% (n=3) |
| Treatment-naive patients | | | 57.5% (n=46) | - | 100% (n=70) |
| *IGHV* gene mutation | M-CLL  UM-CLL | | 43.4% (n=33)  56.6% (n=43) | 52.3% (n=23)  47.7% (n=21) | 52.9% (n=37)  47.1% (n=33) |
| Lymphocytosis (G/L) | | | 30.9+/-39.8 | 30.1 +/-39.8 | 47.1+/-60.7 * |
| Hemoglobin (g/dL) | | | 13.8+/-1.7 | 13.7 +/- 1.8 | 13.0+/-2.0 |
| Platelets (G/L) | | | 203.1+/- 92.3 | 208.6 +/- 94.3 | 194.3+/-76.1 |
| Cytogenetic  (Karyotype + FISH) | | - isolated del(13q)  - normal karyotype  - trisomy 12  - del(11q)  - del(17p)  - complex karyotype | 32.5% (n=24)  17.5% (n=14)  11.3% (n=9)  21.3% (n=17)  11.3% (n=9)  27.5% (n=22) | 39.1% (n=16)  21.7% (n=10)  19.6% (n=9)  10.9% (n=5)  2.2% (n=1)  15.2% (n=7) | 21.4% (n=14)  40.0% (n=28) *  10.0% (n=7)  10.0% (n=7)  7.1% (n=5)  14.3% (n=10) |
| Mutations | | *NOTCH1*  *SF3B1*  *TP53*  *BIRC3*  *ATM*  *MYD88*  *XPO1*  *TNFAPI3* | 10.0 % (n = 8)  26.3 % (n = 21)  21.3 % (n = 17)  5.0 % (n = 4)  22.5% (n = 18)  3.8% (n=3)  7.5% (n=6)  3.8% (n=3) | 17.4% (n=8)  21.7% (n=10)  13.0% (n=6)  6.5% (n=3)  15.2% (n=7)  4.3% (n=2)  2.2% (n=1)  4.3% (n=2) | 18.6% (n=13)  12.9% (n=9)  10.0% (n=7)  7.1% (n=5)  27.1% (n=19)  5.7% (n=4)  8.6% (n=6)  1.4% (n=1) |
