# Supplementary material for: A reduced panel of eight genes (ATM, SF3B1, NOTCH1, BIRC3, XPO1, MYD88, TNFAIP3, and TP53) as an estimator of the tumor mutational burden in chronic lymphocytic leukemia
Source: Int J Lab Hematol. 2020 Dec 16;43(4):683–92. doi: 10.1111/ijlh.13435 (PMC8451785; doi:10.1111/ijlh.13435)
Supplement: Supplementary file 15 — Table S4 [file IJLH-43-683-s001.docx]

**Supplementary Table 4: Impact of different genetic markers on TFS among 110 Binet Stage A and B patients:** number of cases, C-Harrel concordance index (C-index) and log-rank test p-value are given for each parameter

| Criteria | Number of cases | C-index | log-rank test, p-value |
| --- | --- | --- | --- |
| ≥ 1 mutation in the eight gene estimator | 63 | 0.408 | 8.10^-4^ |
|  |  |  |  |
| ≥ 2 mutations in the whole panel | 63 | 0.408 | 8.10^-4^ |
| *ATM* mutation | 29 | 0.449 | 0.020 |
| *NOTCH1* mutation | 21 | 0.459 | 0.032 |
| *SF3B1* mutation | 23 | 0.461 | 0.019 |
| *XPO1* mutation | 6 | 0.462 | 0.005 |
| *TNFAIP3* mutation | 3 | 0.484 | 0.075 |
| *TP53* mutation | 14 | 0.487 | 0.25 |
| *MYD88* mutation | 6 | 0.489 | 0.32 |
| *CREBBP* mutation | 1 | 0.493 | 0.14 |
| *BIRC3* mutation | 11 | 0.501 | 0.44 |
| *CXCR4* mutation | 1 | 0.502 | 0.78 |
| *CD79B* mutation | 1 | 0.503 | 0.62 |
| *BRAF* mutation | 3 | 0.505 | 0.85 |
| *IRF4* mutation | 0 | NA | NA |
| *CARD11* mutation | 0 | NA | NA |
| *MYC* mutation | 0 | NA | NA |
