# Supplementary material for: A reduced panel of eight genes (ATM, SF3B1, NOTCH1, BIRC3, XPO1, MYD88, TNFAIP3, and TP53) as an estimator of the tumor mutational burden in chronic lymphocytic leukemia
Source: Int J Lab Hematol. 2020 Dec 16;43(4):683–92. doi: 10.1111/ijlh.13435 (PMC8451785; doi:10.1111/ijlh.13435)
Supplement: Supplementary file 16 — Table S5 [file IJLH-43-683-s008.docx]

**Supplementary Table 5: Impact of different genetic markers on TFS among 110 Binet Stage A and B patients at diagnosis:** number of cases, C-Harrel concordance index (C-index) and log-rank test p-value are given for each parameter.

| Criteria | Number of cases | C-index | log-rank test, p-value |
| --- | --- | --- | --- |
| Binet stage B | 22 | 0.369 | < 10^-4^ |
| Unmutated *IGHV* | 50 | 0.381 | 3.10^-4^ |
| ≥ 1 mutation in the eight gene estimator | 63 | 0.408 | 8.10^-4^ |
| Complex karyotype | 16 | 0.436 | 0.011 |
| del(11q) | 12 | 0.443 | 0.009 |
| Trisomy 12 | 15 | 0.446 | 0.011 |
| *ATM* mutation | 29 | 0.449 | 0.020 |
| *NOTCH1* mutation | 21 | 0.459 | 0.032 |
| *SF3B1* mutation | 23 | 0.461 | 0.019 |
| *XPO1* mutation | 6 | 0.462 | 0.005 |
| del(17p) | 5 | 0.474 | 0.020 |
| *TNFAIP3* mutation | 3 | 0.484 | 0.075 |
| *TP53* mutation | 14 | 0.487 | 0.25 |
| *MYD88* mutation | 6 | 0.489 | 0.32 |
| *BIRC3* mutation | 11 | 0.501 | 0.44 |
| Isolated del(13q) | 28 | 0.529 | 0.16 |
| Normal karyotype | 37 | 0.589 | 0.04 |
| Isolated del(13q) or normal karyotype | 65 | 0.606 | 0.0005 |
