# Supplementary material for: A reduced panel of eight genes (ATM, SF3B1, NOTCH1, BIRC3, XPO1, MYD88, TNFAIP3, and TP53) as an estimator of the tumor mutational burden in chronic lymphocytic leukemia
Source: Int J Lab Hematol. 2020 Dec 16;43(4):683–92. doi: 10.1111/ijlh.13435 (PMC8451785; doi:10.1111/ijlh.13435)
Supplement: Supplementary file 17 — Table S6 [file IJLH-43-683-s002.docx]

**Supplementary Table 6: Cox univariate analysis of TFS:** HR**:** Hazard ratio; LCI: Lower confidence interval; UCI: Upper confidence interval; p-value: probability that the hazard ratio = 1 (null hypothesis)

|  | HR | LCI | UCI | p-value |
| --- | --- | --- | --- | --- |
| Eight gene estimator |  |  |  |  |
| 0 mutation | **1.0** |  |  |  |
| 1 or more mutations | **3.4** | 1.6 | 6.9 | **0.0010** |
| TMB |  |  |  |  |
| 0 or 1 mutation | **1.0** |  |  |  |
| 2 or more mutations | **2.7** | 1.4 | 5.2 | **0.0037** |
| *SF3B1* mutation |  |  |  |  |
| absent | **1.0** |  |  |  |
| present | **2.8** | 1.4 | 5.9 | **0.0051** |
| Normal karyotype or isolated del(13q) |  |  |  |  |
| absent | **1.0** |  |  |  |
| present | **0.41** | 0.21 | 0.80 | **0.0093** |
| *IGHV* mutational status |  |  |  |  |
| mutated | **1.0** |  |  |  |
| unmutated | **2.3** | 1.2 | 4.5 | **0.012** |
| Trisomy 12 |  |  |  |  |
| absent | **1.0** |  |  |  |
| present | **2.5** | 1.2 | 5.3 | **0.014** |
| *NOTCH1* mutation |  |  |  |  |
| absent | **1.0** |  |  |  |
| present | **2.2** | 1.0 | 4.7 | **0.044** |
| Isolated del(13q) |  |  |  |  |
| absent | **1.0** |  |  |  |
| present | **0.49** | 0.21 | 1.1 | **0.091** |
| *TNFAIP3* mutation |  |  |  |  |
| absent | **1.0** |  |  |  |
| present | **3.3** | 0.76 | 14 | **0.11** |
| *XPO1* mutation |  |  |  |  |
| absent | **1.0** |  |  |  |
| present | **3.2** | 0.71 | 14 | **0.13** |
| del(11q) |  |  |  |  |
| absent | **1.0** |  |  |  |
| present | **2.0** | 0.77 | 5.2 | **0.15** |
| *TP53* disruption |  |  |  |  |
| absent | **1.0** |  |  |  |
| present | **1.8** | 0.74 | 4.3 | **0.19** |
| *ATM* disruption |  |  |  |  |
| absent | **1.0** |  |  |  |
| present | **1.6** | 0.74 | 3.4 | 0.24 |
| *TP53* mutation |  |  |  |  |
| absent | **1.0** |  |  |  |
| present | **1.7** | 0.64 | 4.3 | 0.30 |
| *ATM* mutation |  |  |  |  |
| absent | **1.0** |  |  |  |
| present | **1.6** | 0.67 | 3.6 | 0.30 |
| Complex karyotype |  |  |  |  |
| absent | **1.0** |  |  |  |
| present | **1.6** | 0.61 | 4.1 | 0.35 |
| Normal karyotype |  |  |  |  |
| absent | **1.0** |  |  |  |
| present | **0.71** | 0.34 | 1.5 | 0.36 |
| del(17p) |  |  |  |  |
| absent | **1.0** |  |  |  |
| present | **2.5** | 0.33 | 19 | 0.37 |
| *BIRC3* mutation |  |  |  |  |
| absent | **1.0** |  |  |  |
| present | **1.5** | 0.59 | 4.0 | 0.38 |
| *MYD88* mutation |  |  |  |  |
| absent | **1.0** |  |  |  |
| present | **1.1.10^-7^** | 0 | **-** | 1 |
