# Supplementary material for: A reduced panel of eight genes (ATM, SF3B1, NOTCH1, BIRC3, XPO1, MYD88, TNFAIP3, and TP53) as an estimator of the tumor mutational burden in chronic lymphocytic leukemia
Source: Int J Lab Hematol. 2020 Dec 16;43(4):683–92. doi: 10.1111/ijlh.13435 (PMC8451785; doi:10.1111/ijlh.13435)
Supplement: Supplementary file 18 — Supplementary Material [file IJLH-43-683-s017.docx]

Amplicons : Series 1

| Amplicon_ID | Forward_Primer | Reverse_Primer | Chromosome | Amplicon_Start | Amplicon_Stop |
| --- | --- | --- | --- | --- | --- |
| AMPL7152996658 | AAAGTGTATTTATTGTAGCCGAGTATCTAATT | ACAGGTGATATATTTTAAGAATTCTGTGCTTCT | chr11 | 108160263 | 108160425 |
| AMPL7153087129 | GGTGGAGCAACCTTCAATATCCT | GTAGAATGGGCTTATTCCTCTTCTCTTT | chr12 | 49433165 | 49433439 |
| AMPL7152996963 | GAGAATGGAATCCTATGGCTTTCCA | CCGTCATAAAGTCAAACAATTGTAACTTGA | chr17 | 7573853 | 7574124 |
| AMPL7153087155 | CTCAGTACATGTGCTTGAACGACT | CGGCCAAGTGTTACACAAATTCC | chr12 | 49422505 | 49422778 |
| AMPL7152996964 | GGGTTATAGGGAGGTCAAATAAGCA | GGCCTCTGATTCCTCACTGATTG | chr17 | 7578062 | 7578319 |
| AMPL7153087429 | GGGTGTTGGATGAAGACTGTTG | GGTCCTGCCCAGTTCATTGAG | chr12 | 49432289 | 49432522 |
| AMPL7152996969 | AAGGTGATAAAAGTGAATCTGAGGCAT | AATGGGACAGGTAGGACCTGAT | chr17 | 7576945 | 7577218 |
| AMPL7153087523 | AGTACACAACTTGTCAAGACAGAGAATG | AGGCCTGTTAACATGAAAACAACAAAAA | chr12 | 49446912 | 49447185 |
| AMPL7152996971 | CAGGAAGTCTGAAAGACAAGAGCA | CCAGGGTTGGAAGTGTCTCATG | chr17 | 7579714 | 7579988 |
| AMPL7153087701 | AAAGTTCAAAACCTGCAGCGTTT | GCAGCCTTCAATGGGCTTATTTG | chr12 | 49424571 | 49424845 |
| AMPL7152997068 | TTCCTTCTTCAATTTTTGTTGTTTCCATGT | ACACACACATAACTCCTTCATAAACAGAAT | chr11 | 108186514 | 108186701 |
| AMPL7153087758 | AGATCCCTGAGAAGATGGCCTT | CCTATCACAGATTGGTTTCCCTGA | chr12 | 49448095 | 49448369 |
| AMPL7152997069 | CAGTGGTATCTGCTGACTATTCCTG | TTGCCAATATTTAACCAATTTTGACCTACA | chr11 | 108204529 | 108204803 |
| AMPL7153087827 | TGGGTTTCTGCTAGGTTGTCTG | CCAAAGAGACCTTCACAATTACCTTCC | chr12 | 49425328 | 49425539 |
| AMPL7152997072 | ACCAAGTCAGTGGTCTTAATTGAAATTATG | GTAATATCTTGGTAGGCAAACAACATTCC | chr11 | 108217915 | 108218174 |
| AMPL7153087850 | AGGACTGGTCCTGTAGATAAGGC | CTGCGGCACTTAAGTCCTCA | chr12 | 49425583 | 49425857 |
| AMPL7152997074 | TTGTCCTTTGGTGAAGCTATTTATACATGT | GTTTCTCAAGTTTTCAGAAAAGAAGCCA | chr11 | 108190598 | 108190858 |
| AMPL7153088001 | ATGTTTAAAGGTGGTAGCTGTTGAGT | TCCTTTATAAGCCTGTAATTTGGGACATC | chr4 | 153258880 | 153259154 |
| AMPL7152997080 | GTACATGAAGGGCAGTTGGGTA | CTTGATGAAAAGATGAAGCATATTCATGCT | chr11 | 108198302 | 108198551 |
| AMPL7153088005 | TTTTTCCAGTTGCTACTTGCAATGA | GCCTTCATTTTTCTCTTCACCAGTATTTT | chr4 | 153250752 | 153251017 |
| AMPL7152997081 | GTTGCCATTCCAAGTGTCTTATTTTTGT | CATGAGGAAAAATTCACAAACAACAACC | chr11 | 108106329 | 108106601 |
| AMPL7153088012 | CACTGTCCTGTTTTGATATCCCAGA | CCCTTTCCTACTAGGATTAAGGTCAGTAAT | chr4 | 153245353 | 153245627 |
| AMPL7152997083 | GTTTTATTTCACAGGCTTAACCAATACGT | AAGAGCAAGACTTTGCAAAATATTCTTTCA | chr11 | 108165595 | 108165860 |
| AMPL7153088019 | GCCTAAAGTGATTCACTCGCCTT | GGAGGCTCTGATCCACTCCTT | chr12 | 49421467 | 49421741 |
| AMPL7153234370 | TACTTTGAATTTCATTCCAGGTTGTAGGT | AAAAACAAACCTGGAACATTCAGTATCTG | chr18 | 3119785 | 3120042 |
| AMPL7154368988 | CTTTGAGCTTAAGTTTATTTCCGATTGGTT | ACCATCTTCATTGTTAACAAGAAATTCACC | chr11 | 108213771 | 108214038 |
| AMPL7153234444 | CCTGGGAATTTAGCCTCCACTT | GCGTCTTTGGCTCTCTGAGTAT | chr18 | 3141860 | 3142133 |
| AMPL7154368990 | ACACCCAGCTGATATTTTGGGATTTTA | AGGACACCAGTCTTTATTTTCATAATCCAA | chr11 | 108187956 | 108188180 |
| AMPL7153234462 | GGAGAGTGGCTGTTAACTGCAG | GCAAGAACAAGAATTGTGTGTGTGT | chr18 | 3175960 | 3176215 |
| AMPL7154368992 | CGCACTCGGCCTTAAGGTTAAT | GCCTTGGTATAAGATGCATCTTTACTATGT | chr11 | 108170313 | 108170531 |
| AMPL7153234508 | GGAAACTCGAGATGGGAAACCT | CTCTGGAGATATTCATTAAGCCATACTTCT | chr18 | 3151738 | 3151956 |
| AMPL7154368994 | TACATATAAGGCAAAGCATTAGGTACTTGG | TGCTTTTCTATACCACATTCTCTCACAAT | chr11 | 108124480 | 108124663 |
| AMPL7153234523 | GGCTTCCTCAGTGTATTCTACAATGA | TTTTTCACTTGATGGAAAATGAGTACCAAT | chr18 | 3094109 | 3094382 |
| AMPL7154369001 | AGATACTGTGCCAGTTGAGTACATTTT | TGAAGATTTAGCCATTCCAAAACCAGATAA | chr11 | 108153260 | 108153534 |
| AMPL7153243399 | TAGAAATACTTGTGAGTGTCAAAATGAGCA | GGACTTTTCCTAGATGATATTTGTTTGCTT | chr15 | 75702129 | 75702400 |
| AMPL7154369004 | TGACAAGTAGTTAAGTCCTCAATGAATGG | GCTGGCATCCAACTTCTTGATC | chr11 | 108196670 | 108196943 |
| AMPL7153247998 | AATGTAACCTTGCTAAAGGAGTGATTTCT | CTTGGCAGCAGGATAGTCCTTC | chr8 | 128752597 | 128752853 |
| AMPL7154369006 | CAATTTTTAATGATGCTTTCTGGCTGGAT | CCCTTATTGAGACAATGCCAACATTAATTT | chr11 | 108180901 | 108181101 |
| AMPL7153254014 | AGGACAAGGAAGCTGTGTTTGA | GCAGTTTCTGTCCTTTCCCTGTT | chr7 | 65554600 | 65554850 |
| AMPL7154369008 | GGCAGTAGAAGTTGCTGGAAATTATG | ACTCACTCAGTTAACTGGTGAACATAAAAT | chr11 | 108199747 | 108200019 |
| AMPL7153254055 | TTCAGCGCCAGCACCTCTG | TGAGGGTCATCAGGAGCCCG | chr7 | 65554052 | 65554287 |
| AMPL7154369009 | CCTCTTATGTACCAATTGGCTGCTA | GCCTATGAGGAATTAGAGAGAGAAAATGAG | chr11 | 108202207 | 108202456 |
| AMPL7153254123 | GAGCCACTGGATTCTGAGGT | TTTTCTGTCCCACTCTGCACTT | chr7 | 65551632 | 65551905 |
| AMPL7154369010 | AAGAATTAGAGATGCTGAACAAAAGGACT | GAACCTCCACCTGCTCATACAC | chr11 | 108163243 | 108163512 |
| AMPL7153278496 | CAGTCCTCTCAGCCTCCATACT | GCTGGGAATAGGCAGTTTGCT | chr1 | 27057826 | 27058075 |
| AMPL7154387902 | GGGAATGAAAGCAGTGGTTTGT | TGGAAACTATGAAGCCCTTCCAATAATTTA | chr7 | 124510757 | 124511031 |
| AMPL7153320572 | ATTGGAGGAATTGGTTTATTTGTGGTTTAC | CCTGTCTTTACGAGCACAAGTTC | chr1 | 27099252 | 27099523 |
| AMPL7154387905 | TGTATTGTTCCTTGTATAAGAAATGGTGCT | GGACTGTACTTGTCTACATTTTATTTGCAC | chr7 | 124475342 | 124475529 |
| AMPL7152997091 | AGTGGGAGACAGACACATAAACAAG | GCAACACCTTCACCTAAAATAATCATGTT | chr11 | 108178538 | 108178810 |
| AMPL7153088035 | AAAAGAGGCCAAGGTACTTCACTTAAA | AGAGCTTTTAGTAATGTGCCTGTATTACAT | chr4 | 153303279 | 153303552 |
| AMPL7152997102 | CCATCTTGAACATCTTTGTTTCTCTTCC | GAGGCCTCTTATACTGCCAAATCAATATAA | chr11 | 108137864 | 108138138 |
| AMPL7153088043 | CCAAAGTAGGTCCAGTTTTCCCA | CCAACACCAAAGAGGAAAGTAGCTT | chr12 | 49442355 | 49442627 |
| AMPL7152997118 | ACTTTTTAAAGTAAATGATTTGTGGATAAACCT | GCTTAACAGAACACATCAGTTATTTTATAACTT | chr11 | 108141732 | 108141941 |
| AMPL7153088082 | CCACGGGATTCTTCATCTGCAA | GCAAGTTATTTGATGGGAAATGGACTG | chr12 | 49437172 | 49437357 |
| AMPL7152997141 | CCCTCCAATAGCTTGCTTTTCAC | AAAAACAGCAGCATGCTAATGAACT | chr11 | 108122518 | 108122791 |
| AMPL7153089499 | AAAGAAAGAGATTTACCATGCATGAATTCG | GCAAGGGATAGGTATGAATTAGAATTGCTA | chr2 | 61719444 | 61719697 |
| AMPL7152997163 | AAGGCCTTTAAACTGTTCACCTCA | TTCTAAAGGCTGAATGAAAGGGTAATTCA | chr11 | 108236001 | 108236275 |
| AMPL7153089781 | AAAATTATCTTCTAAGAGTCTTGGCCCAAA | CCATCAACTCCTGTGTCGTCTTC | chr16 | 3820517 | 3820789 |
| AMPL7152997300 | CCATCACACTTTGTTTATTATACTGGCCTT | GACTGAATATCACACTTCTAAAAGGTACGT | chr11 | 108202635 | 108202811 |
| AMPL7153089853 | GGACACTTAAGAGCCCTGGTCTA | CCTTGTCAGCAACAGCCTTTGTA | chr16 | 3789505 | 3789775 |
| AMPL7152997360 | CCCTTTCTTTCTAGTTTTTAGAAGTACCCA | CACAGCACTCTTTAGATAAACAGGTCATAA | chr11 | 108173566 | 108173821 |
| AMPL7153089882 | ACTCGTTGCAGGTGTAGACAAA | TCCCTTGTGTGGGACTAAAGC | chr16 | 3781237 | 3781511 |
| AMPL7152997390 | CTGGAATTTGCATTTTTCCTTCTATTCACA | ACAATGAGTTGTGACAATCCCACT | chr11 | 108128174 | 108128425 |
| AMPL7153090136 | GAGACCGCACCTGGTTACT | AGCGGATTCTGCAGCAACA | chr16 | 3778012 | 3778170 |
| AMPL7152997395 | ACAGAAACTAAAAGCTGGGTATCTTAGAC | GCTGAGTAATACGCAAATCCTTAAAAACA | chr11 | 108163889 | 108164163 |
| AMPL7153090162 | CCACTCCCTACCTACTCTCTGA | AGGTATAATTGAAAGTGTGACGATTTGGA | chr16 | 3841898 | 3842160 |
| AMPL7152997404 | TTCAGTGGAGGTTAACATTCATCAAGATT | CAGCAAACAGAACTGTTTTAGATATGCT | chr11 | 108172303 | 108172577 |
| AMPL7153090173 | CACATTCAGGCTCACGGG | CGTGAGCATGTCACCAGCT | chr16 | 3779042 | 3779309 |
| AMPL7152999913 | CTCGGATGTGGGCTCACA | ACCGTCCTGTCTTCCCTCTC | chr9 | 139400124 | 139400359 |
| AMPL7153090183 | CTCAGAACCATGTGTTGAGAGGA | GTGATTTAAACTGCCAACAAACTATCTGA | chr16 | 3794794 | 3795062 |
| AMPL7152999954 | CTGCATCTGTAAGTTTTGTGGCT | CAGTTTGAATGGTCAATGCGAGT | chr9 | 139391012 | 139391285 |
| AMPL7153090184 | CTGAGGTAGGAGAAGGCAGAC | ATGAAGCGGGTGGTGCAGC | chr16 | 3779386 | 3779656 |
| AMPL7153320574 | TAGTCATTCTCACTAGGGATTTCTTCAAGA | TTGTCACTCTCTTTTCTTTTTCCTTCCT | chr1 | 27092866 | 27093140 |
| AMPL7154387907 | GGAAAATACTCACAGCAAATGACATTTAGG | CAGGAGGATGCATGTCATAAATATGATTCT | chr7 | 124482848 | 124483081 |
| AMPL7153320576 | CTTCATGAGCCATTTCTAGCTCTGA | TTCATGGTCAAACAGCTCTCCAA | chr1 | 27087782 | 27088056 |
| AMPL7154387916 | TCACTACCACCCAATTTGAAAACGTA | GACAGCCAATCAGCATTCAGATG | chr7 | 124491712 | 124491984 |
| AMPL7153323032 | CACAAACTGTCTCTTGCCATAGAATAAA | TGTGCTCTCTAGGATTTGTTAGCTG | chr12 | 49446282 | 49446556 |
| AMPL7154387921 | AAGAAAGATTATTTTAAAAATTGCTCTAACCCA | CTGACAATAGACATTTTAGTCTACGATAACCA | chr7 | 124498797 | 124499067 |
| AMPL7153323053 | GCTCCACTGAAGATCCCAGTCT | ACCCTGAGAAGTTCCCATCTTCT | chr12 | 49440364 | 49440603 |
| AMPL7154387938 | CATTCATTTGAAAGCGGGAGAATACC | CGCTTACACCAAAATCGATTTCTATCATTT | chr7 | 124481044 | 124481280 |
| AMPL7153323081 | GGAGGCAAAGCATGAACTCAGA | TGGTCTGAAGACTGACATAACCAAG | chr12 | 49436269 | 49436507 |
| AMPL7154390016 | TGATTGGTTGGATCAGGATTCAGTTT | TCCACAAACCAATGTGTTAGTTTAAACAAA | chr12 | 69229641 | 69229911 |
| AMPL7153323122 | GTCAGGACCTGCAAAAGGGTAA | GTGGGAGTAGGAACTTGTGTCTT | chr12 | 49428102 | 49428352 |
| AMPL7154390023 | CTTAATGCCATTGAACCTTGTGTGA | AAATTTCAGGTTGTCTAAATTCCTAGGGTT | chr12 | 69233447 | 69233699 |
| AMPL7153335289 | GTTTACTTGAAGGCCTCCGGAAT | CCTCGCCTGTGGACAACAC | chr9 | 139390521 | 139390719 |
| AMPL7154390035 | TGTTCATGTAGCAGTGCATTTTCAC | CTCAGGAAGATGCCAAGCGGAAT | chr17 | 62007659 | 62007794 |
| AMPL7153348148 | GCACCCACTTCCAGTCTTCAAA | CCATCTTTCCCAATGCCGAACT | chr10 | 64575474 | 64575680 |
| AMPL7154390433 | GGTAGGAAACCTCTGGACCTCTA | GAAGCCAGTGAGTACCTAGAAAGG | chr1 | 27094425 | 27094561 |
| AMPL7153363298 | CCTGACTTTTTAATGATCTGCCTGTTCT | TGGCATTGCCAAAATGATCACAG | chr6 | 138202134 | 138202403 |
| AMPL7154392664 | CTGCGAAGGAGATGTTGACTGA | GCGGTAACATAAAGGTGTTTGATATTCAT | chr16 | 3817734 | 3817965 |
| AMPL7153381866 | ACTAGGTTTCGAATAAATTCACCCGTT | AATACTGAGGACATGGGTTTCTAAATATGT | chr4 | 153244175 | 153244449 |
| AMPL7154392792 | AGACAGTTCAGGGTGGAGTTCTA | GTGGTCAGGATAACGACTTATTAAACTGTT | chrX | 41200750 | 41200937 |
| AMPL7153397054 | ACTTAGACCTCAACCTGGAGACA | CATTAATGTCCAGCACCTCGATCT | chr4 | 134071483 | 134071644 |
| AMPL7154392793 | CCATCAAGTACAAGGCAATGAAGAAAG | ATGGACAGGCAGACAGATACACACAC | chr3 | 38182647 | 38182825 |
| AMPL7153415382 | CCCTCCCTCAAGTAAAACTTACTTGT | GTGGAGCTCGAGAAATAACACACATTA | chr19 | 30311556 | 30311809 |
| AMPL7154392795 | CCCTGAGGTACTCATCTTTCCTC | CTTCAGTCGATAGTTTGTCTGTTCCA | chr3 | 38181713 | 38181985 |
| AMPL7152999967 | GAGGTGGGCCAGTCTCAAAG | CGTGGACTCCCTGGAGTCA | chr9 | 139391377 | 139391594 |
| AMPL7153090200 | TTTTTACCCACAACCCACTCCAT | CCATCATGTCTTTTTGTTTGAAGAACTAGT | chr16 | 3799541 | 3799801 |
| AMPL7153031136 | TTCATATTTAACCACAGTTCTTTTCCCGTA | CTGCAGCATTCCAAATACTTCATGAATATT | chr11 | 108143418 | 108143620 |
| AMPL7153090215 | CATGTGAGAGGGAGGGCTATCT | TGTGCGTTCCATCTTATTTTACTTAAGAGT | chr16 | 3824522 | 3824796 |
| AMPL7153031836 | TTTTTAATGCACAAAAAGAAACACCCAAA | GGGAATTTAGTCCAAAGGAATGCCA | chr13 | 48922990 | 48923236 |
| AMPL7153090299 | CTACCTCCTTGTGCTTCTCCATG | GACCTACTTTGGCCTGAGCTT | chr16 | 3781773 | 3782046 |
| AMPL7153031851 | CCCATTAATGGTTCACCTCGAACA | GCTACTGCAAAAGAGTTAGCACTAATTACA | chr13 | 48936965 | 48937222 |
| AMPL7153090306 | GCTAAGGGATGGCAGTAGGAAA | ACAGATCATTCAGTTGCTTTTTACAGTTTT | chr16 | 3827978 | 3828246 |
| AMPL7153031874 | TTTCTCCCTTCATTGCTTAACACATTTTC | AAAAAGCAAGAAAAGATTATGGATAACTACATG | chr13 | 48947493 | 48947766 |
| AMPL7153090328 | TTCAAGAGATAAAACAATGGACACTCAGAA | GTTTTAAATGTGAGGTAGGAGGGATCT | chr16 | 3808781 | 3809044 |
| AMPL7153031945 | GAAGAGCAGCTATAATCCAAGCCTAA | TCTGCATGAAGACCGAGTTATAGAATACTA | chr13 | 49038936 | 49039209 |
| AMPL7153090366 | GCTTTTGTGCTTGCGGATTCAG | ACATAAACAACTGTCGGAGCTTCTAC | chr16 | 3900624 | 3900898 |
| AMPL7153031962 | TCTAGGGTAGAGGTAACCTTTAATTTGGT | CAATATGCCTGGATGAGGTGTTTG | chr13 | 49047372 | 49047645 |
| AMPL7153090382 | GCCAATGGGCAACACAGGAATA | CCTTACTAGACAAAGTGTGTTTGAGTCAC | chr16 | 3830673 | 3830946 |
| AMPL7153032318 | GGGTCTGCTCTCATCGGTTTTC | CTGATGATGACAGAAGTGAAAGGTCT | chr8 | 42147642 | 42147886 |
| AMPL7153090403 | CCAACCACACCAGCAGATAGTG | TTCTTGCAGAATGCACCTCACT | chr17 | 62006723 | 62006959 |
| AMPL7153032326 | CATGCGGCATTTATCTTTTGCAG | GCAAATACACACAAATATGCAGAGTGT | chr8 | 42162616 | 42162890 |
| AMPL7153090412 | ATGAGAGGGAGACAGGCGGTAA | GGTGCAGTTACACGTTTTCCTC | chr17 | 62009425 | 62009696 |
| AMPL7153032344 | GGCTAAACCATCTAGGTTTGTGTTATGTT | TGTAATGCCGCTGTGCCTTA | chr8 | 42171716 | 42171968 |
| AMPL7153090473 | GCCCTGTTGTCCTTCTACTCCA | GCCACCTATGAGGACATAGTGAC | chr17 | 62006390 | 62006663 |
| AMPL7153032354 | CTTGAAGAAATCGGTTTTCCAGTAACA | GGTAAACAGGATGGTGTGTACGA | chr8 | 42174184 | 42174458 |
| AMPL7153091116 | CTGGACATCTCGGCGAAGT | GAGTGGGATGCGGGAGATG | chr18 | 60985550 | 60985815 |
| AMPL7153032368 | CCAGTAGTGTCGAAGGCAGGATA | GTGGCCAAAGCCACCAAGGAATT | chr8 | 42175984 | 42176256 |
| AMPL7153095526 | GGGATATGCTGAACTAAGTTGCCA | TTCTTTGGTACTGCATCCACAGT | chr3 | 38182193 | 38182443 |
| AMPL7153415384 | CAAGGTGTACTAAGCTTACATCCAGT | GGCAGTTTTAAGAATTTGGCCAAGA | chr19 | 30313288 | 30313562 |
| AMPL7154392816 | ATCATGACTAAAGAACGTGTGTGATGT | ACTGAGCTGGATTACTCTGCTCT | chr1 | 27087212 | 27087461 |
| AMPL7153415388 | TGTAAGAGCTGTTTGCATCTTATCTCAC | CGTCCATGCTATGAGGGATAAAGG | chr19 | 30307990 | 30308263 |
| AMPL7154392858 | CGCGGTTAGGTAGGAAGTATTGA | GGCCATAGCTTAATTAATCAGGCTTCA | chr16 | 3900255 | 3900516 |
| AMPL7153415389 | CATGGAACATGGCTGCATTTTGA | ACGCCTATTCTAGAGTTCCTCACT | chr19 | 30312833 | 30313107 |
| AMPL7154392859 | GACAGTCCTGCGACGAACTT | GTGAAAATGGCTGAGAACTTGCT | chr16 | 3929659 | 3929923 |
| AMPL7153427805 | CTTCAAACCTTTTCGGCCACTT | GCATGACCTCACACAAACATTAAGC | chr3 | 93615341 | 93615614 |
| AMPL7154392866 | ACATAGGAACAACCCAAAGTCTTCTC | GAGTAAGCTCAATGTTTCCCATGATAATTT | chrX | 41202275 | 41202520 |
| AMPL7153457266 | GGGCAGTCTTAAAGACTGTCCA | CCATGACCCTCTATTTGTGCCT | chr6 | 44229338 | 44229611 |
| AMPL7154392878 | CTCTATGTCGCCAAGGCTGATAA | GAAGCAGCTGTGTACCATTCCT | chr16 | 3807070 | 3807342 |
| AMPL7153469687 | CTGGGTATCTGGGTCTCTCAGT | GGTTGGCTGTGTGTTTCTTCCT | chr19 | 13414481 | 13414730 |
| AMPL7154392880 | CTGTACACCCAGCTCTGATCAC | GCAGAGGTCTTTGAGCAGGAAA | chr16 | 3807636 | 3807875 |
| AMPL7153474191 | AAATAATAGGGAGGAGAAAGCATGAAGAC | ACCGCGTACTTCACCATCAAC | chr18 | 3067162 | 3067436 |
| AMPL7154392884 | TAAGATGAACAATTTGCTCTGTGGAGAA | AGGATCCAGTAGCGTTCTCTGT | chr1 | 27105357 | 27105630 |
| AMPL7153474195 | TGGGAGGAGCCATAATCGTAGG | TATGATCTCAGCTACCGCAACAAG | chr18 | 3214935 | 3215185 |
| AMPL7154392887 | CCACTATGGATGACATGTTGTCTACTC | CAAAGTCATTGCCTGGCACAAAT | chr1 | 27106115 | 27106387 |
| AMPL7153497064 | TCATTTCCTCATCAGGAGACTGGAA | TGTGTTAAAGCCTTTATGGAAGGGTATC | | |  |
| AMPL7154392913 | GCAAAACATGCCACCACAAATG | TGTTCGGTTCACGCCATGATAG | chr1 | 27101137 | 27101305 |
| AMPL7153497214 | AAAACAAAGAGAAGCTGATACAAAGACATG | GCACACACCAACAACATGATGG | chr16 | 3823609 | 3823883 |
| AMPL7154392915 | CGAACAGATGAAATGCTGCACA | GACCTGCCTTCTGCATTTTCATC | chr1 | 27101300 | 27101570 |
| AMPL7153515365 | TGGTCACATCTGACCAGTCAGA | GCAGGTAGCTCAGACCATTCTC | chr1 | 120458024 | 120458256 |
| AMPL7154392919 | CTGGTTTTTGGTCTTCAGCATTTCATT | CCTTTTCTGAGTGTTTCTTGCCA | chr7 | 2979404 | 2979622 |
| AMPL7153584996 | GGACATGATCATCCTACCCGAGAT | GCTTTACGGGCTTGTAGGTGATG | chr19 | 1440171 | 1440405 |
| AMPL7154392926 | TGTAATCTTGAATGACTTATGTAGTGGCAA | GACTATTCTACGAATCTGAGGCTCAAA | chrX | 41204230 | 41204502 |
| AMPL7153032378 | TGAGTTGCTTATTTCTGTTTCAGTTTTGTG | TGTTTGATGAGAAAGTGAAAAGTGGGA | chr8 | 42177024 | 42177244 |
| AMPL7153096216 | TGAGAAAAATGAACTCTGCCCAAAGA | TGATGTACGTGGGTCTCTCGAT | chr6 | 106552734 | 106553008 |
| AMPL7153032386 | AAGATTGCTTGTGAGCCCAGAA | CTCAAGGAAGCCAGCAGAGAGTA | chr8 | 42179283 | 42179544 |
| AMPL7153099074 | GTCCACTGCTGGCTGATCTATG | TGTGGGTAAGTTACTTAAACAGGAAATTGA | chr22 | 22127105 | 22127355 |
| AMPL7153032408 | AAAATGTGATTCATCACTTGGCTCCTA | GACAGACATTTACCAGTGACAAACG | chr8 | 42183437 | 42183705 |
| AMPL7153099105 | CGAGCAACAACACCTAAACCTC | GCTACACCAACCTCTCGTACATC | chr22 | 22221426 | 22221660 |
| AMPL7153032536 | AGATTTGACATTTAGTTAGGTCAGTTCCT | CAAATGGCAAGTTCTGCTGTCT | chr6 | 395777 | 396038 |
| AMPL7153099190 | TGAAGTGACTTTGATGTAAGCTGTACAA | CAGGAAGATCTCATCATGTCCTGTAAC | chr22 | 22142914 | 22143165 |
| AMPL7153032546 | CGTCGGACTCTCTGTCTAGACA | AAGTGTCTAAAGTCCCATCGAATCTG | chr6 | 398779 | 399032 |
| AMPL7153106532 | GCCAGCTCCTTGAAAAAGCAGTA | GTTGGATAAGCCTCTTATTCATATCCTGAA | chr1 | 27097676 | 27097906 |
| AMPL7153032563 | CTCTTGCTTCCTGTTTAACCTGGT | TCATTTGGGACTTTTCTAAATGAAACTCTG | chr6 | 404940 | 405213 |
| AMPL7153106705 | GTCAGACTTGGGAATACGTAGCA | ACACAGGTGTGCTGTTTATGGG | chr18 | 51056828 | 51057102 |
| AMPL7153032872 | TCCCTGTTCACTTGACGCAAAT | CTGAAGAGATGATACAAATCCCATAGCA | chr14 | 103357531 | 103357803 |
| AMPL7153106910 | AGGTCTGGCCTCTGGAGAATTA | GGCACGATTCACATTCACACAT | chr3 | 183245561 | 183245831 |
| AMPL7153032893 | TGACACTCATGCTGATGGATCAG | TTGCCTTCTGCTAAATCCACCT | chr14 | 103371895 | 103372156 |
| AMPL7153106955 | AGGTCATTTAAAGCTCTGAGTATCTTGC | TGTCGCGGTGATATACTTTGAAAGG | chr18 | 50451526 | 50451799 |
| AMPL7153034342 | ATTGACGGCTTTAATGAAGAGAAAAGC | CAGAACCGACATTACTGGCATTTTT | chr6 | 106547205 | 106547479 |
| AMPL7153106972 | CTGAATGTCAACATCATGATGAAATTTCCA | TAAGCAATTATAGGACAAGGGAGAGCTA | chr18 | 50929067 | 50929319 |
| AMPL7153034373 | GGACTCCTACGCTTACTTGAACG | CCGAGGAGATTGCTGTAGACAG | chr6 | 106553148 | 106553390 |
| AMPL7153107031 | AGCTTGGTAAAACTTCTGCCACAT | CCCAAAAGACAATGACTTCTTTCCTC | chr18 | 50976807 | 50977080 |
| AMPL7153034402 | AACACTTGAGTCTTGGAGCAGAA | GGAGGGTGACTCACAGACATTT | chr6 | 106554166 | 106554437 |
| AMPL7153107115 | TGAACAATAACCTCACGGAGCC | GTCGTGAGAGTTCTGCTGGG | chr1 | 27023232 | 27023506 |
| AMPL7153034713 | CACTGGACAAAACACTCTGAAGGA | GTCCTGACCTATTGATTGCTCGA | chr7 | 2977447 | 2977721 |
| AMPL7153107143 | GCCCTCGGAGCTGAAGAA | GTTCTTCAGGTCCGGCTCC | chr1 | 27022954 | 27023200 |
| AMPL7153584999 | CGGACCAAAGCGATCTCTTCTG | TTACGAGGACCCGGACATGAAG | chr19 | 1438390 | 1438644 |
| AMPL7154392928 | GCCGCAAACAATACCCAATCTC | CAAGTCTCGAATCTGCTGACCAATA | chrX | 41203302 | 41203566 |
| AMPL7153587314 | ATCTGGCCCATGGAGCTGC | ATCATGAACCCAGGACACAACC | chr16 | 3778257 | 3778531 |
| AMPL7154392935 | CAGGCAAGGATTCACTGACCTT | GCCCACTTAAAAGAAAACAAATACTTGAAC | chrX | 41205479 | 41205731 |
| AMPL7153587388 | CGCTTCGCAGCCTCAAAG | GCTGTCGATCTGGTCGATCA | chr6 | 392986 | 393254 |
| AMPL7154392936 | AGGAAGTAAAGGTAGGGAAAGAGCT | CCGTTTACCATGAGATCCTTATTGGA | chr16 | 3788341 | 3788611 |
| AMPL7153602107 | CGGAAGTCGCAGTTCCATCTAG | CATGGCCAGCTCCATAGATCAG | chr16 | 3777615 | 3777878 |
| AMPL7154392941 | CCCTCCCAGTTTATGGATCCTTT | CAGGATGAAGATGAAGTGCTTAATGC | chr7 | 2987006 | 2987266 |
| AMPL7153622470 | CTGGATTTCTTTTTATGCAAACTTCATCCT | CAGAAAGTATCTGCCACTTAGTAGCATTAT | chr22 | 22153268 | 22153535 |
| AMPL7154392948 | AGATGCTGGCTCGTGATTTCTT | AATGAGATCACACCTGTGAATAACCC | chrX | 41204655 | 41204895 |
| AMPL7153622714 | CCAAGCTAACCTTCACCCTTGA | GGATCACATACTCTTTCCTCTTATGCAG | chr12 | 49438517 | 49438776 |
| AMPL7154392991 | CTCATCATCAGTGCATAGCTTCTCA | GCTGTGGTGGTTGAGACTGT | chr1 | 27057553 | 27057823 |
| AMPL7153633172 | GCACGGCCTCGATCTTGTAG | GTCCTGACTGTGGCGTCAT | chr9 | 139397637 | 139397891 |
| AMPL7154393074 | GGGAAAGCCTCAATCAGCTGAA | AAAAATACCCAAGCCAAAACGACTG | chr16 | 3786470 | 3786738 |
| AMPL7153665817 | GGTGAGAGAGCAGAAGTCAACAC | TTCTGTGATGGGCCTTTTCTGTAG | chr1 | 120459100 | 120459341 |
| AMPL7154410390 | GGTTAGCTGTTAAGATTCGGCTCAT | TGGCTGAAGACAACCTTCTCTG | chr4 | 153253592 | 153253792 |
| AMPL7153667755 | GCCAAGCTCGTCTCAGAGAAG | GTCGAGGAGAGCAGAGAATCCGA | chr8 | 128750932 | 128751188 |
| AMPL7154410393 | CCCTCCTGCCATCATATTGAACA | CTTTTGAAAATGGTTGTTGCTGTGTAAAAA | chr4 | 153247262 | 153247529 |
| AMPL7153675022 | TGATAACAGTTTTGGTGTTTTATGTCTCCA | TCAGGAAGCCTTGAAAATGGAATATATGAT | chr18 | 50734018 | 50734292 |
| AMPL7154410396 | TCACCTGAAACATTTTTAGCCATTCCT | GCCAGATCATCATTCTTTGTACTAGAATCT | chr4 | 153268078 | 153268336 |
| AMPL7153704011 | ATCCATGATGAATGATACAAGCTTGGA | TTTAGATCCCAGTATTTCAAATGACATGTAGTA | chr3 | 93605146 | 93605418 |
| AMPL7154410403 | ATCCAGCCACCCACCAAAATTA | CCACATTAGAATCTGTGACATACCTACCT | chr4 | 153273504 | 153273773 |
| AMPL7153704019 | ATTTGCTAGTAAAATTTCTCTAACTGGGATTAT | GGTCTCCTGAAAAGTTCTCTGCA | chr3 | 93624551 | 93624825 |
| AMPL7154410408 | GGGCAGGTCCACAATACTACTG | CTCCTCAGGAAACCAAGAAGAACAAGAG | chr4 | 153332497 | 153332686 |
| AMPL7153034732 | CAGGTTGTAGATCCTGTTGACCA | CGTATCTGTTGCTGGTTCTTCTGTC | chr7 | 2978352 | 2978561 |
| AMPL7153107168 | CAACCACCAGTACAACTCCTACT | TGAGCAGTTGGTTGAGGGTG | chr1 | 27023518 | 27023792 |
| AMPL7153035623 | TGGAGGGCTGCAGTATGTTATG | CTTCCTTTTCAGATCCCAAGCACT | chr7 | 2998017 | 2998277 |
| AMPL7153107232 | GCAGTTGGACCTATCTCCATACC | GGTTCTTTCGGTCACTGAGGAA | chr1 | 27106596 | 27106870 |
| AMPL7153037783 | ACAGCAGTCCCATTTACTCAGC | GCTGCTGTGGAAACAGGTAGTC | chr5 | 137802731 | 137802919 |
| AMPL7153107243 | ACATGCAGAACCCACCCTTT | CAGTCAGTTTCTAAGTTCTCCACACA | chr1 | 27107039 | 27107311 |
| AMPL7153038279 | CCATTTGCCATTCACTGCAGTA | CGGTACCGTGTGTGATTTCTGA | chr6 | 106546827 | 106547097 |
| AMPL7153110102 | CGTCGCTGAAGTGGTCCTT | CAGCATGCAGTTCTAAGGCTCT | chr9 | 139399516 | 139399690 |
| AMPL7153038677 | GGGCTTGGTATATATGTGGTCAAAAC | TTTCCATTGTATTCTGACAGTTGTACTTCA | chr22 | 29191505 | 29191771 |
| AMPL7153111030 | GCGTGTATTTTGGGACTCCAGAA | TGACTGCCTACATGGGCTTTTT | chr6 | 138200417 | 138200582 |
| AMPL7153038716 | GGTCATGTCACTTGGAACCAGT | GGAAAGGAAACCAATTATACTGAATCAGGT | chr22 | 29193007 | 29193280 |
| AMPL7153115068 | TTTGGCTGTCAACTTTCGAATTCG | AGGAAAGTCATATCCTCCTAAAGAACACTA | chr11 | 108115578 | 108115851 |
| AMPL7153045138 | GTCCTGCGAAGGCACTTG | CCCACCATTTTCATTGGCAGC | chr12 | 49435028 | 49435189 |
| AMPL7153115404 | CAGGAGAGTCCTCACCCTCTT | GCGTTGTTGCTGCTATTGTGC | chr19 | 42382951 | 42383165 |
| AMPL7153045710 | CATGCTCCAACAGGTCCTCAAT | CTCCCTGCTGGAAAAGTTTGAG | chr12 | 49431373 | 49431518 |
| AMPL7153116961 | AGAGACTGCGTGCAGTTCTTC | CCAAATTCAACGGGCTCTTGT | chr9 | 139399867 | 139400095 |
| AMPL7153055922 | CAGACTCACATCACCAAGTGCA | GATTGTGGCCTTCTTTGAGTTCG | chr18 | 60985288 | 60985462 |
| AMPL7153124107 | TGGTCTTAAAATGGCCAGAGCAA | TCCACATTAAAGTGGTCATGATTAACAAGT | chr3 | 176755814 | 176756085 |
| AMPL7153056955 | ACGAGGAGGAGAACTTCTACCAG | ACTCTGGTTCACCATGTCTCCT | chr8 | 128750585 | 128750850 |
| AMPL7153124155 | TGAGTATGAGGCTCTGAGGGTT | GTTGCCTTGTTCTGATACGTGAGA | chr3 | 176767718 | 176767989 |
| AMPL7153059531 | TGTCAGAATGACTTTTTAGTACAGGGAGTA | CAAGGCATAAGGCTGAAAGCAT | chr6 | 138196754 | 138197028 |
| AMPL7153127680 | CGTCCTCGTCCTCCAGTGAT | GCCACTGACCAAGCTGAAGAG | chr5 | 137802382 | 137802618 |
| AMPL7153059557 | CCTATGGCCTTGTTTAGTAGAATACTGTT | AGATGACACAGGAGAGAGCTGAA | chr6 | 138198157 | 138198430 |
| AMPL7153131360 | TCAGAGAGTTAAACCATGCAGTAAAACA | TAGGGTTATATCCTGTGTTGTGACCT | chr3 | 176782539 | 176782813 |
| AMPL7153724067 | CGCTCTTCCTTCATCTTGTAGTACC | TCTGGAACCTCCTTTTTCCACTTC | chr7 | 2983970 | 2984233 |
| AMPL7154414765 | GTTCTTTTTCACAGTAGTGTTATGTGCA | CTCTCTCTCTGACATGATCTGGTATCTTTA | chr13 | 48881310 | 48881498 |
| AMPL7153774548 | GCCAGCTTCAGAGAATAGTGAGGA | CTCTGCTGTTGTCACATGCTTC | chr1 | 27105774 | 27106047 |
| AMPL7154414796 | TTTCTTCATCTGTATCCCTTGTAATATGCC | GAAGGCGTTCACAAAGTGTATTTAGC | chr13 | 49033618 | 49033871 |
| AMPL7153789030 | ATCTTGGCATCTGTGGGCTTTA | CCATACTGGTTGTATACATCTTGCTGA | chr1 | 27100748 | 27101013 |
| AMPL7154415225 | ACCCAAGCAGACTAATACAGTCCA | AGCCTCCATCAAAACGAAAACG | chr2 | 198281390 | 198281559 |
| AMPL7153804262 | AGTGAAGAAATCGGAAGAAAACACTAAAGT | CAAAAGACCAGTGTCCAAAAAGAGC | chr4 | 87655385 | 87655659 |
| AMPL7154418461 | CATCTTCAAGTTCAGTTGCAGCAA | GGGAGTTCTTTTGGAAAGTGAAATTAAGAA | chr2 | 198288531 | 198288750 |
| AMPL7153804264 | TTTAACACCTTGTCCCTTTCTCGT | AAAAATGCTCCCTAGAGACGTTTTTG | chr4 | 87706313 | 87706587 |
| AMPL7154418470 | CTACTGGATTTCTAGCTCTTCCTCTATG | CCGGAAAGTCAGAGATGTATATTGGAAAA | chr2 | 198256894 | 198257168 |
| AMPL7153804265 | AAAATGTTGCTGCCTTATTGTTCTATTGAT | TCCTAGGCATGGAACTCAAGACT | chr4 | 87674047 | 87674321 |
| AMPL7154418490 | GTTCTGTTGACTGTGGTATATCATTAAGCA | GCTTAGACATCACACTGTCAATAGATCTTT | chr2 | 198285755 | 198285974 |
| AMPL7153804270 | TGAAATGCTATTTTACTTATCCAGGTGGTT | GCGAGGAATAATTTAAAGGTGAGATTGTAG | chr4 | 87688946 | 87689175 |
| AMPL7154418503 | TTACGGCATAAATGTAGTCTTTTCCCAT | ACAGCTTGTTGACCCATTTGTTTTT | chr2 | 198260811 | 198261082 |
| AMPL7153804275 | CCTGAGTCCCTCCTCAAACAAA | ATCTCAATGAGAACTGATGTTATTCACACA | chr4 | 87687486 | 87687751 |
| AMPL7154418518 | ACAGGCTGTGTGTGTACCTCTA | CGAGACACACTGGTATTAAGATTGTACAA | chr2 | 198267128 | 198267378 |
| AMPL7153804282 | GGATTAGATTTGTACAACTCTATGTCTGCT | AAAAACAAACACTGGTCACTGGAAA | chr4 | 87638070 | 87638324 |
| AMPL7154418522 | CCCAAAAGCAGCTGGTTATTTATACG | GCTCAAAAATATGGGATCCTACACCTAG | chr2 | 198274379 | 198274649 |
| AMPL7153804285 | TTTCTGTGTTTCAGGCAGGCTAA | GTTTGGCCTAAGTTCCTAAATTCATGC | chr4 | 87679751 | 87680006 |
| AMPL7154418546 | CTCATTGGTGGTGTTCCATTCTTAATTTTT | CCATCTGGAAATCTTCCATTTTTAAAACCT | chr2 | 198269804 | 198270058 |
| AMPL7153804305 | GATTGTCTATTTGTACAGGAGACCGTTT | GGAATGAGACAGAAAAGTGAAACAGAATAC | chr4 | 87680077 | 87680280 |
| AMPL7154418551 | TTTTAAAAATCTTTAACTTACAGGCAGTGGG | GCTATTGGACCATGTAGAATGTTGC | chr2 | 198257461 | 198257734 |
| AMPL7153804306 | TTCACTTAGTTAAATTGGTTCTGGAGGAA | CTTACAAACTAAATGCATACACTTTGGGA | chr4 | 87724804 | 87725065 |
| AMPL7154418552 | CATAACTGCCTGAATTACATGAGGAGA | CTTATAAGACCGCATCTTAAAGGACTTTTT | chr2 | 198257759 | 198257957 |
| AMPL7153063301 | CGGTGGAACAAACACTCGCTTA | GCACCCAGAAAATGAAAAGTAAGGGT | chrX | 41193447 | 41193661 |
| AMPL7153134614 | GTGCAGGCAATGGAGGCAGAT | CCCAGGCTCACTTCCTAGGA | chr7 | 65557473 | 65557699 |
| AMPL7153063355 | GATTGGAAGGCTGGAGATTTTAATTTCC | CATAGAAAAACAACACCATGTTTAGACCTC | chrX | 41198177 | 41198451 |
| AMPL7153150263 | CCCAATGAGAAGAACCAAATGGTAC | TGAGTCTTATCACCAAGAAGGGACA | chr16 | 81956995 | 81957269 |
| AMPL7153063525 | AAAAACACTGTCATCTACCAATGTCTG | TAGCATCATTTAGAAAGCCCTCTCAATG | chrX | 41202920 | 41203151 |
| AMPL7153152909 | GTTTGCTTGCTCTCGTCTCTGA | GACTTGGAACTCTGAGAGGTTAAAGTATTT | chr16 | 3843481 | 3843679 |
| AMPL7153063902 | AGCAAGTTACTTTATGGAAGACCTTTGT | ACTATGAACTGTAAGTGAACACCCATTC | chrX | 41206056 | 41206321 |
| AMPL7153161403 | CACCCAAACATCTGTTGCTGTT | AGCAGCAGATATTGATCATAAACTTGAAGA | chr2 | 198265244 | 198265517 |
| AMPL7153063930 | TCAGCACTATAGAAACTTGATGGCAAA | CGAGTTCTTGAAGCTACACAAGGT | chrX | 41206792 | 41207066 |
| AMPL7153163436 | AATGACTTTCTAGTAACTCAGCAGCAT | CTCTTACCTAAACTCTTCATAATGCTTGCT | chr7 | 140453001 | 140453272 |
| AMPL7153069853 | GGCAGGAGCTAGAAGATCCATG | CCCAAACCCTGTACTAGTGTTAGC | chrX | 70470932 | 70471166 |
| AMPL7153164013 | CTGCTCACCATCGCTATCTGAG | TCTGTCTCCTTCCTCTTCCTACAG | chr17 | 7578363 | 7578578 |
| AMPL7153069895 | GTCCTTGAATACGCTCCCTCAT | GAGGCTCCTTCCATGAGTTCTG | chrX | 70469733 | 70470006 |
| AMPL7153184020 | CTGATGTTCGCTGCCTCATTTC | GGAGAATGTCCCAGGGAAGTGA | chr19 | 42384904 | 42385128 |
| AMPL7153069977 | ATTCTCCCTGGCTGGGAGTTATA | GCCAGATGTTACAGCTTGTCCT | chrX | 70467960 | 70468208 |
| AMPL7153185259 | GACCGAGATCCTGCTGCTTT | GTCAGAGCCCAGACCCAAAAGTG | chr12 | 69202070 | 69202317 |
| AMPL7153070050 | CTTGTCCAGCTCACGGACATAG | GTTGGTGGCACAAATGTTCCTC | chrX | 70466123 | 70466395 |
| AMPL7153185298 | TGCAAATTGGAAAGGTTATTTACAACAAGT | AGAAAGAATATCAAAAAGCTGTGTGAATGC | chr12 | 69218250 | 69218484 |
| AMPL7153070136 | TGTTGGAGAAAGGAGGTTAGGGAA | GGCTGCAGGGTTGAGATCTTAA | chrX | 70464526 | 70464799 |
| AMPL7153192804 | GGCGTCTCATCCTCCACATTC | CCTTGGCTGACATCCTTATTCCTC | chr6 | 44227799 | 44228048 |
| AMPL7153070203 | CGGGATGGTGGATTGGAAGAAT | GCTTCAGCATCATTATCTTTCTTCTAGGAC | chrX | 70462758 | 70463005 |
| AMPL7153192815 | CTAGGGCACCAGAAGAGCACATA | CCCTTTCCCAAGTCACCTTTTCC | chr6 | 44226850 | 44227084 |
| AMPL7153070271 | GATTGAGCATGCTCTCCAACATG | GCCTTGGTACTGATCTTCTGTCCT | chrX | 70460831 | 70461019 |
| AMPL7153192820 | GCTATATTAATCTCACTGACCTCCTCCTT | TGGCCCTTTAAAATAATTGCTATAGCTAGT | chr2 | 61118773 | 61119038 |
| AMPL7153804309 | AGAGTGGGATACAGAATTTTACATAAGCC | ATGCTCTAACATGGGAACTCTCAAAAA | chr4 | 87732095 | 87732369 |
| AMPL7154418578 | CCTGGAAATAGCTAAGAGAATGGAATGAC | GAGTATGACTCCTGAACAGCTTCAG | chr2 | 198272589 | 198272833 |
| AMPL7153804314 | TAACAACATCCCAGAAATGTGAGTTTTCT | GAAATCAGTTTGGTTCAAAACACAAACATG | chr4 | 87703255 | 87703497 |
| AMPL7154418596 | GGAAAGAATTACCATCTGCAAAAGGATC | TGTGCTTTATGGTGTTCTGATTTTTGTTT | chr2 | 198285140 | 198285356 |
| AMPL7153804317 | GTTGCAGAGTTGGTGGGAAAAC | GAAGGTAGGAGGGAAGTGTTAATGTAAAAA | chr4 | 87671958 | 87672164 |
| AMPL7154421425 | GGTGTTTTCACAAATAGGTGGTATTTTCA | CCATATGGCTGAGGTCTCATCTTG | chr1 | 27055918 | 27056191 |
| AMPL7153804319 | ACTGTGTAACTAGTGTCATTCTGTAAACAG | ATTACCAGATTTCAAAGCCAGGTAAGTATA | chr4 | 87614648 | 87614921 |
| AMPL7154422320 | GATGAGCTGGAAAGTCACAATGG | AGTCCCAGCAGATTGGATGAAC | chr1 | 120458415 | 120458601 |
| AMPL7153804320 | GCCACTGTTTGGAGTGTATTAATTGAATG | CTTCAGAGCATTTATAGATAGACAGAAGCA | chr4 | 87717868 | 87718142 |
| AMPL7154422322 | GCCAAAGGCTGCATTTCATGAA | GTAGGAGGAAGAAGTCTCTGAGTGA | chr1 | 120458713 | 120458959 |
| AMPL7153853150 | AATTACACACAAGGAAAAGGTATTATAAGCAGA | ACAAACAAGATGCTAAAAGTCTTGGACTAATAT | chr3 | 93593045 | 93593303 |
| AMPL7154422332 | TTGTTCAGGTGGCATCAATACCA | GCTCTTTTGCCTGTTCTCTGCTT | chr1 | 120466448 | 120466642 |
| AMPL7153854768 | GACCCACCGTAGGCATGATT | CTCCACACAGCAGGTGGAGGAA | chr12 | 49425902 | 49426166 |
| AMPL7154427663 | AGATACCAGATCCTTGGAGATTTCTCAAT | GACACTGAATGAGAAAATGGTAACACTTT | chr11 | 108119671 | 108119860 |
| AMPL7153912051 | CCCAAAATGGCTGTTGATCCCA | TGCCTTTAAGGCTGGGTCTCTA | chr12 | 49433852 | 49434122 |
| AMPL7154427669 | AAGTATCCTGCAAGTTTACCTAACTGTG | CTTTCTAGGTTTGACCTCTTGTCTTGA | chr11 | 108121456 | 108121613 |
| AMPL7153912053 | GAGGACGGGAATCTTCACCTTC | CACCCACTCAGCACAGCTATAC | chr12 | 49420820 | 49421086 |
| AMPL7154427671 | CGAGAGCTGGAGTTGGATGAATT | TAGAATATTGGGCTGAGTAACACTTGC | chr11 | 108200960 | 108201175 |
| AMPL7153977823 | CTTTACACCAGTGCCACTCACT | ACCTGCACTCTTCTGTTTTACCC | chr1 | 120465238 | 120465463 |
| AMPL7154429526 | CGGATTGTAGAGAGTGGAGTGAGT | CCAGACTATCCTGGATTCTTTCCATCT | chr10 | 64573592 | 64573728 |
| AMPL7153977826 | CAAGTTCAGGTCCAGGAGTTGA | TCATCTGATTTCATCCTACTGACTACTGT | chr12 | 49432603 | 49432813 |
| AMPL7154429528 | CTGCACAGCCAGAATAAGGAGGA | CAAGTTCTCCATTGACCCTCAGTA | chr10 | 64573863 | 64574116 |
| AMPL7153977829 | CATCGTTGTTCGGCTGAACTTG | TGCTGTGGCGAAAAGGAGAAATA | chr4 | 74486042 | 74486307 |
| AMPL7154480365 | CGGCTCACTTACAGCATCGT | CCTCGATCACCAGCTCATAAGG | chr4 | 134073138 | 134073273 |
| AMPL7153071639 | GTCCTAGGCCAATCCTTCTAAGG | GAAGACTAGTTCCTTGCCTTTCCT | chrX | 100611011 | 100611284 |
| AMPL7153192933 | CGGCCAGTTTTTCAGATTTTAACTGA | CTGGAGAGAAAATTTTCAAAGAAGCAGTT | chr2 | 61147631 | 61147893 |
| AMPL7153071672 | AGGTTGCAAAGTGTGAATTTTCCC | TCTTGTGACCGTGCCAAGAAAA | chrX | 100608781 | 100609055 |
| AMPL7153193374 | GCATAGGCTCAGCATACTACACA | CCTATGTTAAAATGAAGCAGTGCTCTT | chr11 | 108225438 | 108225707 |
| AMPL7153075220 | GGTTTTCTGGGAAGGGACAGAAG | ACTGCTCTTTTCACCCATCTACAG | chr17 | 7579381 | 7579614 |
| AMPL7153193521 | CTTCCTGGTTCCTGAAAACTTTGC | CAAATTTCAGAGTGAATGCCAGATAACAAA | chr3 | 89499292 | 89499563 |
| AMPL7153075265 | ACTTGTTCACATAACAAACAACTATCCCA | GAAGAACTGTAACCAGTTGCATTTACAAA | chr7 | 148506306 | 148506579 |
| AMPL7153193556 | ATTTCCCTTGTAGTAGAAAACAGAAGTGAG | GTCGAGGGAGGAAGCGGACATT | chr3 | 89390844 | 89391090 |
| AMPL7153079802 | TCCTAAATCAATAATGCATTTCCACTCCA | AGGATAAAATAGTTACATGGATTTGCTGCT | chr7 | 124532264 | 124532536 |
| AMPL7153193634 | TTATTTTTATGCTTTGCACATTCTGAGCA | TGTCAGGAGGAAGTTAACCAATTGTAAAT | chr3 | 89457129 | 89457369 |
| AMPL7153079849 | TGGCTCATCTATTTAAAAACATGGCAAATG | GCCTTCATACCAAACTTCAATCAATGAATT | chr7 | 124492893 | 124493167 |
| AMPL7153193975 | CGTCTGGACATCACCCTGAA | GGTAGTGAGAACTGCCAAAAACC | chr4 | 126238122 | 126238332 |
| AMPL7153080072 | ATTGCAGGGCATGGAAATTTAGC | TGAAACAAACAAGCACATTACAATATAGGG | chr7 | 124469281 | 124469551 |
| AMPL7153198833 | CCTCCTGAGATGCTCTGCATAG | ACCAATACAGGACAGCTTGGAATC | chr19 | 13482417 | 13482690 |
| AMPL7153080193 | AAGTGTGGGATTGTTAAAATATTCTTGCC | ACAATCTTGATAGAGGGAAACTGTCTAGA | chr7 | 124465274 | 124465539 |
| AMPL7153198959 | GCCGCTTTCGTGAGCCAT | CCTTGTCCACACACTGCTCT | chr19 | 13323318 | 13323590 |
| AMPL7153080420 | CCCAGCCTAGGAATCCACATTTA | GGGATAGACGAACAAGGAATCAAAATTG | chr12 | 49415734 | 49416008 |
| AMPL7153199094 | GGGCCTGTTTCCTGAGGAAG | GAAAGCAGAACTTGCTGGCC | chr19 | 13409860 | 13410102 |
| AMPL7153082293 | TGTAAACAAAGAAAGGACAGTCATGAGT | TCTGTACATGAGCATTTCATCAGTAATTGA | chr2 | 198267557 | 198267826 |
| AMPL7153199181 | AGAGTTGAGAGATATTAGTGAGTTGCTAGT | ATTGGCCATGTTAGGGCCATAA | chr1 | 27089397 | 27089666 |
| AMPL7153085624 | AAAACAAGAAAAAGTCTTATGTAACCAGCA | GTCAAGAGGTAAACTTTTGCATCAAATTCT | chr2 | 198264739 | 198264983 |
| AMPL7153199603 | TGTCTCTTGGGCGATAGGTGAT | TATGGTCTGTGTTGCAAAAAGGGT | chr19 | 13441002 | 13441208 |
| AMPL7153085628 | ACAACATGCATTCAAGTTGACTAAAGA | TCACGTAATCAGCAATGAGTATTCTCTTC | chr2 | 198266049 | 198266323 |
| AMPL7153204845 | TTCCCGTTACAACTAACGTGAGT | GCCTGACCTAAATACTCAGCTTCA | chr16 | 81962120 | 81962379 |
| AMPL7153977835 | GTTCTAAAGACCACTCCTTCCTTTTCT | CATGATTTGGGTCTGGTCAGGTT | chr4 | 74447390 | 74447664 |
| AMPL7154505596 | CCGGACTGGAGGTTCTTTGAAA | CGGAAGCAATGACAGTAGCCAA | chr4 | 126237420 | 126237671 |
| AMPL7153977838 | CCATACATCATGCTTAGAAAACTTTGACG | GAGACACAACAAAACACTCAACTTAAATCA | chr4 | 74477361 | 74477634 |
| AMPL7154505599 | CGCCCTGTTTGCCATAAACAGTA | GGCGGTGTCTAAGATGACTTGG | chr4 | 126237800 | 126238043 |
| AMPL7153977841 | GGTTAGATTTCAGAGAAACATATGGAGTCA | TGTAAATCTGGAAACCATCATAGGCTTC | chr4 | 74453472 | 74453724 |
| AMPL7154505608 | GAACATCTCCGTGCAAATTCTCG | CTTGCTGTGAAAAGACAGGAGGAT | chr4 | 126238742 | 126239001 |
| AMPL7153978044 | CGTGGCTTGACTCGCAGAA | CTGGTCGAGTGCGGTGAA | chr11 | 9685521 | 9685789 |
| AMPL7154505612 | TGAGTGGGATATCTGCCACTGAT | CTGGCTAAATACTGGCTTTTCATCATT | chr4 | 126239047 | 126239318 |
| AMPL7154018803 | ACATGTCAGTGCTTGTGATTTAGCA | AGCTATCAACGTCAGTAAAACAGATTTTCT | chr11 | 108151523 | 108151796 |
| AMPL7154505616 | CTGTGTCTGGGAGGTTGAGTACTA | ACAGTACCATTGGTACCCAAGTCT | chr4 | 126239473 | 126239737 |
| AMPL7154020593 | CAAAGCTGTGGACCCTGATGAA | TGGTGAATTGTCATTTACATCATGGACATA | chr4 | 126240311 | 126240548 |
| AMPL7154505628 | GGGATGCTTTTGGCATATTCCCA | TACAGCACTTACTTTGCCCACAAA | chr4 | 126240688 | 126240941 |
| AMPL7154023713 | GGAGAAGAGCATCTTCCGCATC | AGAGCCGAGGCCTCCTTTCC | chr6 | 393287 | 393546 |
| AMPL7154505632 | TCAGTTTGACAGGGAGTCTCTTATGA | TCATCAACATCTGAGGCAGATACTCTTA | chr4 | 126241043 | 126241282 |
| AMPL7154055851 | CGTGCCTTGAGGTCCTTGG | GACATCACGGATCATATGGACCG | chr9 | 139391655 | 139391929 |
| AMPL7154505639 | AATTGGCAAATTAGACTATGAAGCAACAC | GTGGTTGTTAGTCCCAGTAATACTGTAATA | chr4 | 126241373 | 126241646 |
| AMPL7154142092 | GCCGCTTCTTCTTGCTGG | ACCAGCCTGCTGCGTAG | chr9 | 139396758 | 139397004 |
| AMPL7154505644 | CTCCTCGTTCATCTACAATGTCAGT | GCAAATTCCCGATCTATTTCAGCATTAGTA | chr4 | 126241759 | 126242032 |
| AMPL7154142757 | GCTCAGAGCCACCAAAACTGTT | GGAGGCATAAGATGGTGGTGTT | chr3 | 183211727 | 183211982 |
| AMPL7154505648 | ACCCATCAGCTGTGATTGGTTC | TCAAGGCCCTGAAGAATGATGG | chr4 | 126242179 | 126242440 |
| AMPL7154143103 | GGTGAAAGGGTGCTCCCTTTAT | CCGAGTCGTAGTCGAGGTCATA | chr8 | 128750322 | 128750563 |
| AMPL7154505653 | CGCCTCTTTACTATTGGACGACA | AACATTTTTAGGTGTTCCTGTTCACAAATT | chr4 | 126242601 | 126242875 |
| AMPL7154154392 | CCAAAGAGCTACCCATTCCAGT | TTGGGCAAGCCTCAAAGCACAA | chr12 | 49433584 | 49433839 |
| AMPL7154524756 | CAACTCCTTGCGCGACTAC | CACTCGGATGGTGAGTAGGG | chr4 | 134071754 | 134072015 |
| AMPL7153085688 | TCACCCAATGGCCTTTGCAATA | CTTCAGCCTTTCTGAAGAGTAGTAAGTTTT | chr2 | 198262705 | 198262979 |
| AMPL7153204946 | CACCAGGATCTTGGCATGTCAA | CCTCAGACAAGAAATCCCACCAAAT | chr16 | 81953045 | 81953316 |
| AMPL7153086128 | CCTTCTCCCATAGAAAACCCTTATACAC | CCTGAGCATTGTCAGTGTTGGA | chr12 | 49436806 | 49437080 |
| AMPL7153205361 | AGAATAGTGTGCACCGCCAG | CTGCAGCCAGCAAACATCCAG | chr9 | 139390821 | 139391002 |
| AMPL7153086131 | GTTTGGACTCCAGCTACCAAAC | GGAGATTATATCAGTCTCCCTATGCCAT | chr12 | 49424009 | 49424283 |
| AMPL7153212895 | TTGAGCAACCAAGAGTGACCAGA | GCATCCACCTTCGGTTCATTTTT | chr1 | 16258506 | 16258758 |
| AMPL7153086145 | CCCAACACTCATTTTCCTAAATTCTCTTC | CGTCTGGTGAGTGGACTTTGTG | chr12 | 49448605 | 49448878 |
| AMPL7153212987 | GAAAAGTGATCCAGTTGATCCAGACA | CCCAGAAATGTCATTGATGATGGAG | chr1 | 16259146 | 16259419 |
| AMPL7153086160 | TAAATCCTCATAATGGGACCAGAGGAT | GCACTGAGAACTTTGGCCTAAC | chr12 | 49418283 | 49418557 |
| AMPL7153213068 | CCATTCCACTCCTCCTCAGTCAT | GTTACAGAAGGCAGTGTGGGTAT | chr1 | 16259905 | 16260162 |
| AMPL7153086598 | AAGACATCAGGTGTCTTTAACTCCAG | ACACCTGACCCATTCCTCAAAC | chr12 | 49434573 | 49434835 |
| AMPL7153213297 | CTGCCAAGACACCAGATGCCAA | GTACCGGTACTCTTGAAACAGCT | chr1 | 16262433 | 16262659 |
| AMPL7153086631 | GCGCTATGGAGAGAAGGACAAA | CCTCGCATTGCCTGAGCTTATT | chr12 | 49439748 | 49440001 |
| AMPL7153221799 | TTGGTGTGGCAGGAATATTAAATTGC | ATAAGGAGGGTGGGAAGAAGCAT | chr12 | 23818296 | 23818569 |
| AMPL7153086665 | CCATGAAACCACCAATGCCTATG | GTTCTGATTTGCCTCCATCTCTTCT | chr12 | 49438070 | 49438344 |
| AMPL7153221805 | GAATGAAAAATAAAGCTAAGTGGCAAGACA | ACAGTATTCTTTCTCCCTCTCTCTCTTT | chr12 | 23757270 | 23757506 |
| AMPL7153086689 | TTGGTCTCTCATTTGCCCTATGAC | GCTAGTGCCTGAACTGTTTGTCT | chr12 | 49447717 | 49447990 |
| AMPL7153221819 | CTGCAGAAAACAGAACAGTACCTTTATTC | GTTTTAACGCTAACACGAAGTGATGT | chr12 | 23696083 | 23696357 |
| AMPL7153086693 | CTCAAGAGAGGCCACCCACTAGA | GGGAGTGTGAAAACACTACAGTATGG | chr12 | 49435383 | 49435569 |
| AMPL7153221820 | CCATTCAATAGCCATAAACAGAAAATCAGC | CAGCCTTTCCTTATCACAGCTCA | chr12 | 23728541 | 23728815 |
| AMPL7153087054 | GCTCCTTCCCAAAGAAGGCTTC | CTCAACCCGAGTCTGACACTTC | chr12 | 49437544 | 49437817 |
| AMPL7153221878 | AATGCAAGAAGCAGAAGAGGTGA | TAGCAGATAGACTTTTAATGAGCTCCATTG | chr12 | 23908462 | 23908735 |
| AMPL7153087106 | GGGTGTGACTGGGAAAGAAAAGG | TCGCAGATATTCACTGGAGCATTG | chr12 | 49428543 | 49428780 |
| AMPL7153234348 | AAAAACCACTTCCATTCTCCTCCAT | ACAGCAAAATGTCTCTGAGGCAA | chr18 | 3075344 | 3075580 |
| AMPL7154274111 | GCTCTGAGCCATACTACCTCCT | TCTAGAGGGCTACCTGGGTATTG | chr19 | 42383578 | 42383802 |
| AMPL7154524761 | CTTTCCGCCTCAAGTCTTCCTT | GTTTTCAGTCACATACACGTCGTAGA | chr4 | 134072500 | 134072720 |
| AMPL7154350241 | CCACTTCACCATCTTATGGGCAT | CCGTCCATGATCCCATCTTGTC | chr16 | 3779813 | 3780075 |
| AMPL7154524763 | AACTCTGAGAACGGCTACTTGTAC | GGGCGTTGTCATTTTGATCCAC | chr4 | 134072850 | 134073009 |
| AMPL7154368804 | TATTCTTCAGGATTTCGTAATATTGCCGT | AAGCCCAAAATGCCCAGTTTAAAAA | chr11 | 108117783 | 108117927 |
| AMPL7154524767 | TAGACCTCACCCTCATCCTCATC | GTTACTGGGTACATTGGAGCTCT | chr4 | 134073430 | 134073689 |
| AMPL7154368969 | AGTTCTAGTCTTGTCACTACAAAAGTTCC | GCCATGATAGGCTCCTGAAAACTAA | chr11 | 108195902 | 108196138 |
| AMPL7154564638 | CACATCTCAATTTAGCTAGATTTCAAGTGC | CTGGAGTTCGAACACTCTCATAATATGTA | chr1 | 16254383 | 16254653 |
| AMPL7154368971 | ACAGTGATCTCCTAGTTGTTTTTAGAGC | TCTGACTTATATGCTTCCTCTTCAGCTAT | chr11 | 108126775 | 108127048 |
| AMPL7154564639 | TATCCAGCTCGAGGGAGAGAGT | GAACAGGACTTTGACTTCGTTCAATC | chr1 | 16254683 | 16254923 |
| AMPL7154368975 | GTAATTAATTGCTTCCCTGTCCAGACT | TGCAGAAGTAACGGAAAACTGGT | chr11 | 108216308 | 108216557 |
| AMPL7154564641 | CAAGTCTCGTTTGGAGCGCTATA | TATTCCCTCTTTGTCAGCTTTCTCTCT | chr1 | 16255069 | 16255336 |
| AMPL7154368976 | TCTGGATAAAGTATGATACTTTAATGCTGATG | TAGCAATGGACTTCACCTCATCAAAAT | chr11 | 108154834 | 108155024 |
| AMPL7154564643 | CCAAGCTTGATAATGACACTGTCAAATC | GGAATCTGCAAAGCGCCTTTTC | chr1 | 16255437 | 16255708 |
| AMPL7154368978 | GCCTATGAGGGTACCAGAGACA | GCTATGCTAGATAATGATTACCACAAGCTA | chr11 | 108155102 | 108155337 |
| AMPL7154564645 | AAAGCCTGTGAGGAAAGAAATTCTTAAAAG | GGTTTTGAGGGAATGGGCTTTTT | chr1 | 16255846 | 16256067 |
| AMPL7154368980 | TTGTATCGTCGTGACCAAGATGTTT | CAGATAGCAAAAGAAAAATAGGACCACAAA | chr11 | 108141991 | 108142178 |
| AMPL7154564647 | AAATTGGCATTGACATCGATCACAC | GCGTGAACTTCTGTAATTCCGTT | chr1 | 16256208 | 16256482 |
| AMPL7154368981 | TTTTTACAGACAGTGATGTGTGTTCTG | TCTGAATGCCGATCTAGATGTTTAATTGTT | chr11 | 108098313 | 108098573 |
| AMPL7154564649 | CCTATTCTAACATAACAGTCAGGGAAGAGT | CATCTTCGTCAGAATCAGAAGGCA | chr1 | 16256622 | 16256891 |
| AMPL7154368984 | TGGTCTTGTAGGAGTTAGGCCTT | GAGGCTTGTGTTGAGGCTGATA | chr11 | 108099721 | 108099985 |
| AMPL7154564651 | TACTCTTTTGCATTGGATAAGACAATCACA | GCTCTTCTTCTTTATGGTCCTCTTTCTT | chr1 | 16257029 | 16257293 |
| AMPL7154368986 | GGACCACACAGGAGAATATGGAAA | CTAAGGGCTAAGCCAGAGAAGGGAA | chr11 | 108205698 | 108205868 |
| AMPL7154564653 | GAGCAAACAGCACAACTGATTCC | CAGTGGTCTTCTCTAGTGCTGATG | chr1 | 16257438 | 16257710 |
| AMPL7154564655 | CCCGATAAAGAAGCTGCCATGA | CTCAGACTTCTGAGCTGGTTTTGAA | chr1 | 16257857 | 16258036 |
| AMPL7155373310 | TGCTGAAACTGGAACACAACCA | CCATGTCATCTACACAGTCAACCTCTA | chr2 | 136872890 | 136873162 |
| AMPL7154564657 | CCCAGCTTCTGAAGATTTAGAGGT | CAATTCCAAAAGCTTCTGTGCTTCT | chr1 | 16258132 | 16258339 |
| AMPL7155374327 | GTTGCCTAAAACTGAAAGACAGCA | CTCCACGGTCTTCACAAACTTTTC | chr14 | 103336411 | 103336682 |
| AMPL7154564660 | CGTGAAAGAGAGCTCCATGGAA | CTTGCAGATTTGTCCACTGCTAAC | chr1 | 16258801 | 16259010 |
| AMPL7155521134 | CAACTGTCCTTGCTGGGAATCA | AGTAGCAAGCTGGCTTTTGGAA | chr4 | 153332725 | 153332977 |
| AMPL7154564663 | AGCCTTCTGAGGAAGGAATGGA | CACTTTCTTGTTTGTGTTTCGCTTG | chr1 | 16259505 | 16259779 |
| AMPL7155530303 | CAGAGTCGTTCTGATGGCTTGT | GATTCTGAACTCTGAGGAGTTTCTTGA | chr3 | 183226001 | 183226147 |
| AMPL7154564668 | CCTGTCAGCATTGACCTGGAAA | GTTCACTGGAGTGGTGAGAACA | chr1 | 16260611 | 16260871 |
| AMPL7155531283 | CATCCTGCCGTCAGACAGA | CAACAACCGGCTCTACATCAC | chr3 | 183209705 | 183209913 |
| AMPL7154564670 | GCCGTCAACAAAGTGCAAACAG | CTGCTGAAATTCAATGTCCATTGCA | chr1 | 16261039 | 16261282 |
| AMPL7155531285 | GTAGCAATGTACTCTCCAAGATAAGACC | GCTGGAGAGTGAGAATAAGAAGTGG | chr3 | 183217200 | 183217448 |
| AMPL7154564672 | CCCATTCCACGTTGGTACTGAC | CCTTGGTCTCGATTTTAAGGGTGA | chr1 | 16261380 | 16261649 |
| AMPL7155531294 | CATTTTTCTGGTTTCTTCCCTCTTAACC | AATGCATCAATGCAGTGAGTTTCC | chr3 | 183210052 | 183210326 |
| AMPL7154564674 | GCAGATCGAACTGTCTCCCATT | GGTGGATGCTGGAGATGAACTG | chr1 | 16261760 | 16261952 |
| AMPL7155531298 | CGCGTCGTCAAATTTGACCTTTT | GTGACAGCTGGTGATAAGTGGAG | chr3 | 183273286 | 183273536 |
| AMPL7154564676 | CAGAAGCGCTTCACTCTCCT | CGTCACATGTGGATGCACCAT | chr1 | 16262076 | 16262347 |
| AMPL7155571708 | AAAAACAATTATGTCCAATGAGACAGTTCT | GACTCCTGGAGCCAGTAAAAGA | chr2 | 198273061 | 198273229 |
| AMPL7154588271 | GGAGTGCTGGTACCAGATGTTG | CAATCCCGTGTCTCCTTTGATTTTG | chr19 | 13318812 | 13318936 |
| AMPL7155576143 | TTGAGGCTGAAGATCACAAATAATAGTGTT | TGATGGAGTCCAGGACAAAATAAATGAAT | chr2 | 61719173 | 61719378 |
| AMPL7154598855 | AAAAGGATATGGCCATTCGGTGAT | CAAAGGATGTACAAGCAGTCAATGG | chr19 | 13616736 | 13616906 |
| AMPL7155730762 | ATCATGCCGAGGGAGCG | CCCAACCTGTCTCTTTTGTACTC | chr19 | 30303460 | 30303708 |
| AMPL7154625042 | TGTGCTACTAATTATTGTGCTTTTAAGTGC | TGCTCAAAATTAACTTACCCTGCTGAT | chr12 | 69213925 | 69214172 |
| AMPL7155780905 | AGGAAGCGAATAAGGTACAGATTTCG | CATTAAGGATTACCTGTTCTGTCAGTGT | chr9 | 5054624 | 5054897 |
| AMPL7160389067 | CCAGCACCCTGAAGCTATAGTG | TATAGGAGGCAAAGAAACACAGGATTAGG | chr1 | 27100019 | 27100291 |
| AMPL7160389780 | CTGTTGTTGCTGAGGAGACAGT | TTCAGCAGCAACAGCTTCAACAG | chr12 | 49426515 | 49426711 |
| AMPL7160389817 | CCTTGGGTTCCATCACTCCATT | CTGTTGGCACAGGGCAACAGAAT | chr16 | 3832610 | 3832882 |
| AMPL7160391047 | AGTCCTATGGCCACAAGTCTCT | TCATTATTTTCTCCTCTTTTGTTCCTTCCA | chr12 | 24048751 | 24048989 |
| AMPL7160399421 | AAATTGGACATATTTTGTGCATTGGACAT | CTGACTCAGCACCTCTTGCATA | chr4 | 74442057 | 74442288 |
| AMPL7160401578 | CTGTTTCAGGAAGAAGAACGATTATCCA | CGACCAAAGAAACACACCACATTTT | chr13 | 48954180 | 48954448 |
| AMPL7160403346 | GTCACAGGCGAAGGGCTTCT | CATTCTGAGGCCTCGCAAGTA | chr10 | 64573195 | 64573441 |
| AMPL7160490031 | AGCTCCTCAATGGTCTCTACAATCT | GCCAGTGCCTATGCATCTGTAC | chrX | 70465622 | 70465896 |
| AMPL7160639454 | GTAGAGCAGAAGAAGAAGCGGACCTTC | ACCACACTCAGTTCCAAACCC | chr19 | 1438812 | 1439033 |
| AMPL7160642252 | CCAACCAGGCAATAGTAACCATCA | TCAGCTGATAAAAGGATTGCTCTTCTC | chr4 | 126239887 | 126240159 |
| AMPL7160696256 | GTGACGAGCCAGCCCTTGAA | TGGGTTGGTTAGTTTGAGTTGCA | chr19 | 42381095 | 42381364 |
| AMPL7160864139 | AGGACCTGAAGGCCCTCAATA | CTCCACTGGGCAAGCGTAA | chr5 | 137803043 | 137803167 |
| AMPL7154630439 | CCAAGTTGTTATTTAATGACTGAACGTGT | CAGGAGTATTCATCATGATTGCATCTTCT | chr11 | 102201533 | 102201789 |
| AMPL7155866659 | AGAAACCATACCAATGCAAGGTGT | TTCCCTTTCAGGTGAACCTTGAG | chr6 | 106554850 | 106555009 |
| AMPL7154630440 | CTTTAGTAGAAGCCTGGTAAAACAGACA | AAAAACCTGACTGGATTGAGTATATTTTCA | chr11 | 102201815 | 102202088 |
| AMPL7155867320 | GCTGCACATCTGCCTGTACTAC | TGGCACAGTCTTTTCGCATAGAG | chr6 | 401431 | 401670 |
| AMPL7154630447 | TTCTTAGTTTTTCACTGAAGAAGCAAACTG | TGGTTCTTCTTCATGAAAGAAATGTACGA | chr11 | 102207579 | 102207843 |
| AMPL7155867323 | CCACAGTGCCTTCTCCTTTACC | GTCTTGTAGCCGGTCATGTTTC | chr6 | 106553511 | 106553717 |
| AMPL7154630493 | TGGATGAAGCTAACTTACCAAAAGAAATCA | AGCTTTGGGAGTTTGTTTTGATTCTTT | chr6 | 138199571 | 138199844 |
| AMPL7155867325 | TGCTTGGTTGAGTATTTGCTTATGATCT | AAAACGTGTGCCCTTTGGTATG | chr6 | 106543270 | 106543537 |
| AMPL7154630517 | GGGAGAGAACTCCAGAAGACATTTT | GCAAAAGAAACACAACAGAACAAGTCTTAC | chr6 | 138192429 | 138192689 |
| AMPL7155867329 | CTCTGTAGTGGAGAAGGAAATTCTGG | TGACTCACCAAGTCATAACTTAAGAAACAA | chr6 | 106555139 | 106555412 |
| AMPL7154632656 | GGGACAGATGTGGTCCCTCA | GGAGCCACACCTATCTCCG | chr12 | 49445071 | 49445195 |
| AMPL7155867348 | AACTGTACCAAGCACTCTGCAT | ACAATGTATGTACACTTCTCTTCAAACTCA | chr6 | 106535950 | 106536218 |
| AMPL7154632805 | CTGAGCCCAGATGAGGGAAACGA | CTGTAGCAACAAATGTCTACTTTTGGTC | chr12 | 49422913 | 49423052 |
| AMPL7155867510 | TCACCTGTCTCATTCACCAAACC | CTGGCACACATCTTATAGCCAAAG | chr14 | 103371510 | 103371784 |
| AMPL7154632923 | GCATCAGCTGAGGCGACAA | GGCCTGAGGAATCGCATCT | chr12 | 49444675 | 49444932 |
| AMPL7155867555 | CCACCACGTACTCCTCTGTTC | CTTTTTCTCCCTTTTCCCTTTCTTTCC | chr5 | 137803625 | 137803806 |
| AMPL7154632924 | GGGACATAGGTGATTCTTCAGGT | CACCTGAGGAATTGCCCACTT | chr12 | 49445530 | 49445796 |
| AMPL7155937111 | AGAGAGCGCAGAGACTCAATG | CGCTCCGAACCTTGTCCT | chr6 | 44233007 | 44233262 |
| AMPL7154632926 | CTCCAGTGGAGAAAAAGGTGATGAT | GTCACCACCACCTGAGGAATT | chr12 | 49445918 | 49446065 |
| AMPL7156015952 | GTAGGCTTGGAGTCTGTGGTT | TGCGTGGAACTAATGAATGGAGTG | chr6 | 44233447 | 44233571 |
| AMPL7154632931 | GTGGGCGAGAATAAGGGTCA | TCCAAGTCACCCAGACATCTTTC | chr12 | 49434193 | 49434467 |
| AMPL7156016219 | GGGACTTGAAGGATCTTCACG | CGGGCTGATTCCACCTATGG | chr6 | 44232615 | 44232882 |
| AMPL7154632943 | GGAACCTTTCTCCAGAGCTTCAT | TCTCCTATAACCAGCGGAGTCTT | chr12 | 49435725 | 49435994 |
| AMPL7156055160 | ACATTAAAACACAACTGTAGAGTATGGTCA | CAGCACATAATGGTTTGTGGATTTTG | chr13 | 50623083 | 50623321 |
| AMPL7161082808 | GATCCTTCCCAACGTCTGAAATGA | AAACCTGTGATGACAAAGAGGATAAACTT | chr4 | 48494709 | 48494958 |
| AMPL7161082813 | GCGGTACAGTGCTCGCT | CTCCAGGAAGGCGGTGAGAC | chr4 | 48492657 | 48492893 |
| AMPL7161661113 | AGAAATAGCTGTTTGTTGAATTTTTCCACA | ACTGTTTAGAGCAGTAGCAAAGCTATAATT | chr8 | 117661007 | 117661234 |
| AMPL7161661115 | ACCTCCTACTAACCATAAATGAGAACGA | GGCTTGTTTTTGATGTTGGTAACATCATTA | chr8 | 117671039 | 117671289 |
| AMPL7161661117 | CCCTGGTGTGACTTCAAGAGTT | AGCTTTCTGGATCATTTTACAATTTGTGG | chr8 | 117657190 | 117657406 |
| AMPL7161661118 | ACCACAACCAAGGATTCAAGAGATG | ATGTAACAGAATGTGCCTAGAACCAAT | chr8 | 117669366 | 117669640 |
| AMPL7161661120 | GGGAGAATGCTAGAAAGTACGTCT | CTTGTACTTCCGCTTCCGGTAA | chr8 | 117767819 | 117768093 |
| AMPL7161944523 | ACGGACTGACCTGTCAACG | TGGAACAAAAGGACCAACTTCCTC | chr13 | 50699428 | 50699701 |
| AMPL7162084782 | GCAAAATGACAGGTGCTGTCAT | CAGGCCAGGAGATCACTGAAATT | chr12 | 51583939 | 51584157 |
| AMPL7162084784 | AGTTCAGCTTCGTTTAGCCACTTT | CAACATGCTCTCACTGCCTCTT | chr12 | 51584295 | 51584419 |
| AMPL7162084786 | AAGAGATTGGGTGCCCACATAAA | ACCCAGGGATTTTAACTCCCTGA | chr12 | 51586047 | 51586321 |
| AMPL7162084788 | GCCACACTAGCTGAGTTCACTA | TTGAGGGATTTGTCCATTAGTCTGTTC | chr12 | 51589913 | 51590065 |
| AMPL7154632946 | CCTCGTCCCGCTCAATGTAG | GGCACTGATGAATGTGGAGGTT | chr12 | 49420265 | 49420524 |
| AMPL7156076123 | CACCATCAGTCTGAGGTACACA | GAAGGAAGCTGTCTGGATGTGT | chr17 | 3828667 | 3828802 |
| AMPL7154632948 | GTCACCCTCCTCATGACAGAAAC | CGCCTCAAGAAATGGAAAGGAGT | chr12 | 49420629 | 49420817 |
| AMPL7156079055 | CGCCTGGCCCAGAATAACATTATT | GTGGAGAACCTGCAGTCCTTTA | chr17 | 3844093 | 3844303 |
| AMPL7154632951 | GGGCTTTTGTTGCATGTACTTCA | TATGCAGCCAAGGACCTAGAAAAGC | chr12 | 49416227 | 49416477 |
| AMPL7156079059 | CACGGCAGTGCCTTTGTTATC | CGATTCTCCCTGCAGTACGA | chr17 | 3844466 | 3844715 |
| AMPL7154632974 | GTGTGCCCACTGCTAGAAAATG | CCTTGCCAATTCACCATTGCTAC | chr12 | 49431603 | 49431748 |
| AMPL7156079186 | CTTGTCGGAGCAGATGACTGA | CCACGCCATCTCTGTGATCTG | chr17 | 3850724 | 3850997 |
| AMPL7154632977 | CAGCCTTCTCATTAGCCGATTCTA | GATGACGATTTTGATGCCCACAA | chr12 | 49431898 | 49432159 |
| AMPL7156079220 | GGGAGAAGGAAGTCATGACCAGA | TGGGCTCAAACCATTTATCCTGTG | chr17 | 3856904 | 3857177 |
| AMPL7154632981 | CTGGAATAAAGGATCTCCAAGTCTAGGA | TACGGCTCTGTACTGACTCACT | chr12 | 49443341 | 49443601 |
| AMPL7156079222 | CCCTACAGAAGCCTGAGACAG | AAGGGCGTGCAGAGGATCC | chr17 | 3854353 | 3854605 |
| AMPL7154632983 | ACCCTGGCTCAGATTAGAGATCT | CCTCTGGATGGGATTGATGCTC | chr12 | 49443729 | 49444001 |
| AMPL7156079231 | GTTCCGGGACAGGTACTTGAG | CCTTCCTTTAACCAAACCTCCCTT | chr17 | 3831967 | 3832112 |
| AMPL7154632985 | GGTTCCAAGGCTGGGCATT | GCTTCTCCCATCCTGATGGA | chr12 | 49444108 | 49444337 |
| AMPL7156079238 | CGGTGTCGAACACGTTCATCTT | GTTACACGGAACTCCAGCAAAG | chr17 | 3846732 | 3846927 |
| AMPL7154632986 | AGGGTCAGTGCAGTTAGCTTC | GCACATCTAATCTAAGAGCCCTTCTTTTT | chr12 | 49444392 | 49444617 |
| AMPL7156367838 | TTTTTATCTGCTTTCCTGGTTTCTTTCT | AAAACAAAACCTATTTAAATATGAATCGATCCA | chr2 | 61147142 | 61147289 |
| AMPL7154632990 | CCAGTGCTGAGTTGCACATTC | TCTGGACCAGGTGAATGGACA | chr12 | 49425058 | 49425294 |
| AMPL7156367935 | TCATTACCTAGAAATGAAGCCACAAAGT | CTTCCACAATTCTTGTTTACACGACAAA | chr2 | 61145116 | 61145380 |
| AMPL7154632994 | GCTTTACCTCTCCTGGTTCAGT | CTCCTGACGGGCAAGGAACAAAAC | chr12 | 49426289 | 49426430 |
| AMPL7156367937 | CTAGATGACATAGAAGTTCGTTTTGTGTTG | TGTTATTAGCAGGACCACTTCAAGAAATAT | chr2 | 61145525 | 61145799 |
| AMPL7154632997 | AAGCTGCTGTAAAGAGCCCAT | GACAGGTGCTTATGACCCAGT | chr12 | 49426788 | 49427014 |
| AMPL7156367939 | AGAATGACCCATATAAACCTCATCCTCA | TGGAGAAGAAACAGAACATCCTATGTCTTA | chr2 | 61121581 | 61121746 |
| AMPL7162084836 | ACAAATTCTCCAGACAGCAGAGATG | TCTTCATTCCTCCTGGAAGAATACCT | chr7 | 56136231 | 56136364 |
| AMPL7162084852 | AGTCGCCTATTTAGGGTGCG | GCTGTCGAAGTACTCGGCG | chr4 | 48492265 | 48492539 |
| AMPL7162084854 | AAATGTTGAAGTCCTAGGAAATCACTAAGG | CACGGAAAAGCTCACTCGTCTA | chr4 | 48496048 | 48496319 |
| AMPL7162084856 | ATGGGTTACCGCTGCTGCC | TAAGGCTCTCTCCCGTCAGGG | chr7 | 56132015 | 56132147 |
| AMPL7162084858 | CCTCACTTAGACCACCTGAGTCT | TTCTTTGAAACTCAGTGTTCCTGGAT | chr7 | 56141748 | 56142020 |
| AMPL7162084860 | TTTCTCATCACCCTCTAACTTCCTTTTG | TTGCATTAGAACCCAAGCCTTCT | chr7 | 56144415 | 56144688 |
| AMPL7162084862 | GGCTGTTGGGACCATCATGTC | CAGCTGTGTCGATCCAGGAT | chr7 | 56145919 | 56146164 |
| AMPL7162084864 | GGGTAAGCCCTAGGCCTTTTTC | GAATTGCGCTGTTTGCAACATG | chr7 | 56147159 | 56147382 |
| AMPL7162873024 | CATACCCATGTCTCCACTAGCCTATATAA | GTTCTTCATTCCTTTCTGGTAGAATCACT | chr13 | 50678702 | 50678866 |
| AMPL7162873025 | CCCAGTCACACAACCTTTATTTCTC | CCAATGGGCTCCATGTTCTGTAG | chr17 | 3827171 | 3827401 |
| AMPL7154632998 | GAGCTTGGTTTGTCTGTACTCCA | TGGCAACCTTGCTCTTCGAA | chr12 | 49427183 | 49427385 |
| AMPL7156367941 | TTTTTCCTCCCACAGAACCAAACT | CGAATTAGAAGGAAGTGTCCTTGTTG | chr2 | 61148883 | 61149130 |
| AMPL7154633000 | GCTGTCCAGGTAGTGCCATA | TTAGGTCCGGAAACAGCAGAAGGA | chr12 | 49427486 | 49427751 |
| AMPL7156367958 | CATATTTGGATGCTATTCAAGGTTATTGGC | TCAAATTACCATGTTCATCAGGGAGAAAAA | chr2 | 61143900 | 61144107 |
| AMPL7154633003 | GCTAGGCTGAAGTTTGCTTTCC | GCTTCTTTTCCCAGATGAAGCTGA | chr12 | 49427823 | 49428096 |
| AMPL7156367971 | CCTATTTCCAGCTGCTGCAGTT | TCCTCCCTTGACTTTGAGCAAG | chr17 | 43344772 | 43344918 |
| AMPL7154696864 | GGGCTGATTGGCCACGTA | GGCCTGTGATATCCATGCAGG | chr16 | 3778709 | 3778938 |
| AMPL7156479555 | TGTGCGATCGTGCCTTCT | CGGTTCTCTGGGTCCAATAAATACC | chr19 | 16437781 | 16437945 |
| AMPL7154696906 | GCTTGTGCTGTTCAGGTTTGAT | TAAGGCTTCATCACAAGTAACATACACTTC | chrX | 41201733 | 41201946 |
| AMPL7156479561 | CTGCGGCAAGACCTACACCA | AAGGGAGGTCCTAAATTCATTTTCTGG | chr19 | 16436785 | 16436941 |
| AMPL7154725931 | CCCAGCCCTGATTCTCCAGATA | GCTCCAGATTCTCCAATAACACTGC | chrX | 70472403 | 70472673 |
| AMPL7156479914 | TCACGCCCATTGCCCTG | ATCCAGCTCGGGTCGCA | chr19 | 16436000 | 16436254 |
| AMPL7154725933 | TCTGGAGCCAAAGGCTCTAGT | GCCCTGGACCTGCTTGATAC | chrX | 70472666 | 70472940 |
| AMPL7156479916 | CGGCTTCGTGCATGCGA | GTCGAAGAGACCGAAGGCTGG | chr19 | 16436418 | 16436620 |
| AMPL7154725935 | GCACTCTGGTTGCTCACAAGAT | GAGGCTGAGGAGTTAGACAAGG | chrX | 70465314 | 70465557 |
| AMPL7156479924 | GCGGCTAAATTTAGGCTGCG | CTGGCGAAAGTGGAGAAGGAC | chr19 | 16435513 | 16435781 |
| AMPL7154725938 | CTCTTACCTGCCAGTCCAAGA | CAACATAAAGAGGGTTGGAGAATTGG | chrX | 70468281 | 70468498 |
| AMPL7156564272 | ATCTGGACTGTGATATGTCATTTGTGA | CTTTTTAACTTGGTTTTATGACAATTGCTGAT | chr11 | 108159531 | 108159802 |
| AMPL7154725939 | CTTGACCCATCAGCCACCTTTA | GGACTGAAAACCAACTGTTGTGAC | chrX | 70469226 | 70469448 |
| AMPL7156564273 | TGTTCTCTGTGTACTTCAGGCTCTA | CTAAAACATGGTCTTGCAAGATCAAAAGT | chr11 | 108114683 | 108114912 |
| AMPL7154725941 | CTGGCAGGTATGGTGGGTTATG | ACCCTCATGTTCTTCAACACTAAGTTTTT | chrX | 70461935 | 70462191 |
| AMPL7156573872 | TTTTTGCAAAAAGTAATTCCTTCCAAAGGA | TGAAGAGTGCAAACAATACATCATACTTCT | chr13 | 48919065 | 48919322 |
| AMPL7154725945 | ACCAACAGTCTCTGTCTTACTCATACT | CTACGCTCAAGTGTGTCACAAAG | chrX | 70470207 | 70470481 |
| AMPL7156580348 | CTGCCGGAATTATTAATGAACAAGAACA | ACCACATTCAGCCTGAATTATTTTCAGTA | chr11 | 102206781 | 102207053 |
| AMPL7154725946 | CAGCCTGAGGATGTCAAAAGGA | CAGTGAGGACTGTAAGAGCAAGT | chrX | 70467357 | 70467626 |
| AMPL7156580864 | AGAAGCTACAGGTCCTCTTATAGACATT | TATTGGAGCCTCCGCACCAACA | chr12 | 49419871 | 49420103 |
| AMPL7154725948 | AGAGAGAGCACACAGAAGGTGTA | CAAGACCAGCTTGTTCTGTTCC | chrX | 70468701 | 70468936 |
| AMPL7156643053 | CACAGTCCACCTTTTAGGCCTA | TTGAGGACAATGGCATCTACTTCTG | chr17 | 62007283 | 62007523 |
| AMPL7154725950 | CCTGGAACCTAGACAGCATTTCC | CATATGGTGTCAATGCTTGGAAGTG | chrX | 70463965 | 70464238 |
| AMPL7156643444 | GCATGGTGCTGGGTGATTTCTA | GCCAGGATGTACCACCTTTCTG | chr3 | 183225604 | 183225876 |
| AMPL7154738397 | GCAGGGTTCGGAAGCTAAAAGA | AGATCCGTCATTTCTGCAACCA | chrX | 70467037 | 70467296 |
| AMPL7156683346 | TGAGACGGGACTTATCACGGT | TGCCCACTTGAGCATTCTCATC | chr4 | 126238466 | 126238689 |
| AMPL7154811471 | GTGCCTGTGATGGGACCAAT | GGGCGTCAGCTACGTGTT | chr18 | 3112124 | 3112393 |
| AMPL7156686855 | GGCTCAGTCTACTCCATCTCCA | CTCCGAGGCTTGTATCTCAGAG | chr1 | 16260262 | 16260535 |
| AMPL7154811492 | GCTAGGACTTACCTCGAACAAAGAC | GGGAGACAAGAAGGAAGTTATAAAACAAAA | chr18 | 3164264 | 3164502 |
| AMPL7156799181 | AAAAAGAAACCTAACAGTCTCTCAATCAC | GCAGGGCAGATAAATCAGTTGAAC | chr2 | 198283151 | 198283390 |
| AMPL7154811498 | CCTAAGGTGATTTACATCTGAAGTCAGATT | AGCTCCAGAAAGAAGCTGAATTCC | chr18 | 3089311 | 3089585 |
| AMPL7157003830 | GAACGAGCTAAAACGGAGCTTTTT | GTTAGAAGGAATCGTTTTCCTTACTTTTCC | chr8 | 128752985 | 128753234 |
| AMPL7154920718 | GGGTCTTCCACTATTCTTTTCACAAATT | GAATCCTCATCAGCATGTCCTCT | chr16 | 81946005 | 81946257 |
| AMPL7157036678 | GGTATTTCTGATCCCAACATGCTGT | GCTGTTATCTGCACAACTGAAGTCA | chr2 | 61149260 | 61149529 |
| AMPL7154921648 | CCTACTGGAGTGAGTGGGACAT | GTGCCGAAGCTCCAGTAGT | chr8 | 42166207 | 42166480 |
| AMPL7157036680 | TTTTTCAAGTATAACTTGCAAGATTTAAATCCT | CTCTGAGTTGTCTTTTTATGACAGCTTC | chr2 | 61149657 | 61149897 |
| AMPL7154921725 | GTGCCTGGCTGGGTTTTAGTATA | GTGCTCTTCTTCTTCCGTCTGT | chr8 | 42188316 | 42188473 |
| AMPL7157036682 | TACGTGCCTGTACTGTCAGCTA | GCCATTACAAGTTTTATCTTAAATTTGCTTGAA | chr2 | 61150047 | 61150281 |
| AMPL7154922426 | CATCAGTATGAGCTGGTCACCTT | CCCAGCAGGATCAGAGAAAGTG | chr8 | 42129612 | 42129804 |
| AMPL7157036685 | TCCGGGTGAGGGTTGCT | CCTAGAGGCCTCCTCCTTCTT | chr2 | 61108623 | 61108855 |
| AMPL7154922522 | GGACTGGGAGATGCAGGTATGA | GCCGGACAACAGTCTTCTCATC | chr8 | 42179704 | 42179962 |
| AMPL7157070087 | AGGAAAGGGTGGTAGTGTTTGTTG | ACACCAAGATCCACCTGAGACA | chr10 | 64572877 | 64573151 |
| AMPL7154939445 | CGACGTGATCTGCGTGTG | TCTCCAGTCCCTGACTGTCC | chr7 | 65557770 | 65558027 |
| AMPL7157077238 | ACACTTGACTTCTCTCAATGAGATGC | GCTGTGGCAATGTCTTTCTTCG | chr22 | 17690273 | 17690516 |
| AMPL7154944526 | GCATTGCTGCTACCCACTACA | CAGAATGGTGCTAATGCTCAAAGTG | chr7 | 65547333 | 65547521 |
| AMPL7157088757 | CGGCGAGTTGGACTATGAAG | CGGTGCTGAAGCTGATCTCTG | chr4 | 134072234 | 134072382 |
| AMPL7154993950 | AAAAATGCCTAAGTTGCGTTCAGA | ACTGATGGTAACATTTGTGCCTCTT | chr3 | 89448287 | 89448527 |
| AMPL7157460459 | CAGAAGGAAGTACAGGCTGGAAA | AAACTGATTGTTTCCTTCAGTTTTGTCAAA | chr3 | 93646011 | 93646271 |
| AMPL7154993955 | GTGTTATTTTGTTTCAGCCTTGTATCCA | GGTGAACATAGCCCATGTCTGA | chr3 | 89480122 | 89480396 |
| AMPL7157716287 | CCGGCTTCAGATCTGGC | TATGGTGGTGGTGGCAGCC | chr22 | 29196245 | 29196513 |
| AMPL7155016616 | CGGTGCAGAACGTTAGCTGAAT | CCAGCTAATTGGTGTTTTACCTTAACCT | chr22 | 22160188 | 22160366 |
| AMPL7157793924 | TCTGTAGCAGACAAAGCTGAAAGAA | ACAAATTCACCTTGTTTCCATTTCCTTTC | chr18 | 3083877 | 3084151 |
| AMPL7155067314 | AGAATTACTGCTATCAGGTATGCCTGA | GAGATGAGAGATGGTGGTCCTTTC | chr4 | 134073745 | 134073963 |
| AMPL7158034404 | CCTGCACGCTTCTCAGTGT | CCAGCTTAGGGTAGTTGTCCATG | chr5 | 137801318 | 137801556 |
| AMPL7155098459 | CGTTTTTGATGAGCTCTTCCATTTTCC | GGTAAACAATTATTTGTCCTTTTGCTCTCT | chr12 | 23998926 | 23999200 |
| AMPL7158091266 | GAATGGTCACAAATCTCTTTAAATGTGTCC | AGCTGTCACTAAGTCATCTTCTCCA | chr4 | 87692287 | 87692518 |
| AMPL7155098476 | CACTTCTACACTACTTACCACCAAAGTAG | GAAACAGCGTCAGCAAATGGAG | chr12 | 23893576 | 23893846 |
| AMPL7158091270 | CCTGAGTTCTCAAGATTCCAGGACT | GGCTTTTTAGTTTTGCTTTGGTTACTATCA | chr4 | 87684055 | 87684246 |
| AMPL7155098478 | CATCCCATTAAGTATAACAGGAAGTTTGC | GCTGGAAAGCTATGACAAACCTAGAG | chr12 | 23689327 | 23689597 |
| AMPL7158091271 | GTTAAGTGTTTTCTGTTTCTGGCACAA | TTTCTTGTCTAGTTGGTTCAGAAATGTGA | chr4 | 87693845 | 87694058 |
| AMPL7155098482 | GGGAGAAAAGGGACAGAGAATCATT | GGTTACTTAAATGACCATGATGCTGTCA | chr12 | 23716070 | 23716338 |
| AMPL7158091275 | GCAGACAATATGAAACACCCTTTGAAG | GGTTTAGATGTGTAGTAACCTGAGAATCAA | chr4 | 87643364 | 87643622 |
| AMPL7155098483 | CAGTCAGTGTATGAGAAAGTTAATGTGC | GAGCCACATATCAAAGAAGAGATACAGG | chr12 | 23687053 | 23687290 |
| AMPL7158091278 | TAGATTCTTATCATTCCAGGAAGAAGCTCT | GGGCCACTAGTTTTGAACTGGTAAG | chr4 | 87622375 | 87622604 |
| AMPL7155098535 | AAGGTGGACAAACTATGGCTTTACA | CGTGTGTGAAGAATGCACACTATC | chr12 | 24103633 | 24103907 |
| AMPL7158091280 | GCAGTAACAGTGCGGACTTCAA | TTCAATTCTTTCTCAAACATGCACAGAATT | chr4 | 87622741 | 87622990 |
| AMPL7155131895 | AGAAAATGTGGTAAAGTGTGAAGAGCA | TCTTCTGCCAGTGGATTGTTGG | chr18 | 50432256 | 50432527 |
| AMPL7158433040 | CCTGTACTTGTCCGTCATGCTTC | GTTAATGCTTGCTGAATTGGAAGTGA | chr2 | 136873267 | 136873535 |
| AMPL7155131899 | GGAACTTTTTGCTGCCTGCTTT | GCTTGGGTACCCAAACACATCTAAG | chr18 | 49866922 | 49867194 |
| AMPL7158764061 | AAGAGTAGAGCTGCATGAATATTGAGG | CGTCTTGGACATTACTAAAATTGTGTGA | chr7 | 124503239 | 124503513 |
| AMPL7155131902 | CCCAGAATCCTTGCAACATTTATTTGT | GGTTTCCCACAGTGAGCTGAAG | chr18 | 50705124 | 50705387 |
| AMPL7159565027 | GTGCTGGTTAGTGGGAGTCTTT | GAAACATGCAAACCAAACAATGCTAC | chr11 | 9715647 | 9715884 |
| AMPL7155131921 | TATGCTAAAATAAAGAGTGACTTGGGCAA | CAACTCATGATGATGCAGTTAGTCTG | chr18 | 50865937 | 50866152 |
| AMPL7159565030 | GGAGAAGACGTCTGATGACTTCATAC | TCTTACGCACTTAATTTCTACATCTTCGTT | chr4 | 74459176 | 74459402 |
| AMPL7155245111 | TATGGACGAACTAAGTCATCAAGTTTGTAC | CACCTATTACTCTGCTCTTTTTCCCA | chr2 | 198268319 | 198268515 |
| AMPL7159565033 | CCCAAGATGTTAGACATGCTACTGT | CCTCTGATGATGGACAGAAAAGAAAGA | chr4 | 74450772 | 74450968 |
| AMPL7155255423 | CAAGGTTCCATCACCAGAGGAA | GCCTTAACATTTAACAAGAGTCAGGCTA | chr9 | 139392069 | 139392197 |
| AMPL7159565044 | GCGGCTCACAATGAAAGACATT | TCTTCCTTTTCCAAAGTTCTTTGGTTCTAT | chr1 | 27101689 | 27101853 |
| AMPL7155323969 | TGCGACATCTGTGGAAGAAAGT | GGGACGGGTAAGAGGTAGCAA | chr5 | 137803330 | 137803495 |
| AMPL7159565045 | GGAAAACCCAAGAAACTGCTGT | GTGAAATTTCAGACCCATCTAAGGTATGTA | chr1 | 27089719 | 27089935 |
| AMPL7155325629 | CCATTTCGAGTACAGGCACCAT | GCTGTTGAAGTAGATGAGGTAGCA | chr12 | 113515214 | 113515434 |
| AMPL7159581952 | TCAACAAACTGTAACGCAATTGAAATGT | TGGTAAGTCCTGATGGGTGTGTAA | chr3 | 89258794 | 89259062 |
| AMPL7155325634 | CTGCTGGTCAACAGCACG | GCCGGTTGAGGTTGTTCT | chr12 | 113515585 | 113515858 |
| AMPL7159581953 | TCAACCTGTACTACATGGAGTCTGAT | CCAGATTCTTCACTGTAAATGGGCA | chr3 | 89259215 | 89259484 |
| AMPL7155328686 | AAGGTAAAACACAGTAAAATGCTTATGCTG | CACAGTCCGAACAGAAACTCGA | chr4 | 87609992 | 87610244 |
| AMPL7159597432 | TCCAAAAAGTAGTTAATCTGAGCACACA | CATTCTCTGATCGTTTCCCACATG | chr13 | 51101963 | 51102236 |
| AMPL7155328691 | AGAATTTCTCATGATCTTTGACTCATGCA | GTGGATTTTTGCTCCCAAATCATCTG | chr4 | 87728618 | 87728875 |
| AMPL7159597473 | TCTTTCTGATTTGGCTGCCCATTA | GCATTTGACCAAATCAACATCATTCGTAAT | chr13 | 51007501 | 51007751 |
| AMPL7155328713 | TGATCGTTGTGCCTATGATTTCCA | TCGTCCAATAACTAATCTGACTGTTTTGG | chr4 | 87696142 | 87696416 |
| AMPL7159597493 | TGTTGTGCAACACGTTGGTATACT | GTTAATTGTCCTATCAGAGTGCCCATT | chr13 | 50657130 | 50657404 |
| AMPL7155328715 | ACATCTGTTGTAATAATTTCCAGTCTTGCT | GGCTCTGAGGCACTTCATAATCAG | chr4 | 87607668 | 87607926 |
| AMPL7159597540 | CTCTTGCTTTCCCGACATTTTTACG | CCTAAGCAGGACCCGTATTTGTT | chr13 | 50656244 | 50656516 |
| AMPL7155328716 | TTCTGCTTATGTGATTTGCAGAATTTCT | CCCGTTCAGAAGCATTATGTTTACTTT | chr4 | 87653532 | 87653804 |
| AMPL7159597581 | AGCATTTATTTGCTATGCCAACTTAACAG | TGCTAGCTTAAGTTTATTGAAACTGAGCT | chr13 | 50623590 | 50623864 |
| AMPL7155328724 | AGACTGACAGAATATGGAGTTCATTTTCAC | GCAGAGTCTAAGGAAAGTCTGTTAATATGT | chr4 | 87662796 | 87663059 |
| AMPL7159597582 | TCTTCTCCCAGATATGCAAGTGATATAACT | CCTACTTGGATTAGCAAGATTATGCTTTCA | chr13 | 50618747 | 50619018 |
| AMPL7155328747 | AATTCATTTTGATTCTAGCCAGCTTTGTC | CAAGCTGTTATCATTTTTAGCCAGTTCA | chr4 | 87685622 | 87685862 |
| AMPL7159597585 | GTAACCTTTGCCTTAGGTTTTATGGC | AGAGGGCAATAAATGCCACATGA | chr13 | 50601116 | 50601388 |
| AMPL7155328748 | GTGCTTAAACATAACTTTTACGTGGGAT | ACCCAGGCTTCCTTTTTCTGATTT | chr4 | 87695304 | 87695576 |
| AMPL7159597591 | ACTATAGGAGTATGGTAATACTTGCTGTGT | TGTGTGTGTGTGTTTTCTCATCATTG | chr13 | 50562604 | 50562866 |
| AMPL7155328752 | AAAGATTAAGCCAAATCAAGGCATTGTT | ACCATGGAGAAATGATGAAGCCAA | chr4 | 87593291 | 87593565 |
| AMPL7159606190 | GTTTGTTTCATTTGGTGGGTTTTTAAACC | GCACTTGCAGAAAAGCTGGTTC | chr18 | 50917993 | 50918247 |
| AMPL7155328779 | GTCTAGGATTCAGTTTTTCTCGAGAAGAT | GCCAATCCATCTCATCATTAAAAATGCTAT | chr4 | 87690961 | 87691188 |
| AMPL7159961956 | GGCACCTGGCTTCTGGTT | TCAACCTGGAGAGCAAGCTG | chr7 | 2976486 | 2976726 |
| AMPL7155328789 | AACATGTCATGATCCACTTATTCTGTT | TGTCTCATGTAGGAGATAAAAGTAAGCAGA | chr4 | 87730883 | 87731028 |
| AMPL7160144517 | CCATGCTGCCCACTTAGCAT | AGGCCATGTCTTTGCCACAT | chr12 | 49431054 | 49431261 |
| AMPL7155328793 | GTGGAGGTCATGACAGCCTTTA | ACACACGCACAATCAAAACTCATAAATAAT | chr4 | 87696607 | 87696845 |
| AMPL7160345320 | CCTTTTCTGTTCAGTGAGACCACT | GGCCTCTGCTGTAGTCCTTTTG | chr6 | 138199981 | 138200223 |
| AMPL7155328814 | AATGAATATACTGTACATCAGCACATGCT | GCTTTCACATAAATGCCACCATGT | chr4 | 87686411 | 87686594 |
| AMPL7160375527 | CGCGTTAAGATTCCCGCAT | GGCTTCCCGAACTCCAACC | chr8 | 42128866 | 42129140 |
| AMPL7155328820 | CTCATAAAAGTACTGTGGCGTTACCA | GAACTGTTTTGCAATGGCTGGTAA | chr4 | 87671568 | 87671821 |
| AMPL7160375529 | GCGATTTATGGGAACATTGTAAAGAAACA | GTAAAGAAACCAATGTGTTTGGTTGGT | chr8 | 42129200 | 42129469 |
| AMPL7155373308 | ACTCAGTGGAAACAGATGAATGTCC | GCTGCCTTACTACATTGGGATCA | chr2 | 136872468 | 136872742 |
| AMPL7160387579 | AGGGTTTACTCCAATAAAAGGCAAACA | TCTAACACTTCTTGCTTACTTGGAAAAGAT | chr4 | 87656691 | 87656963 |
| AMPL7152996965 | CCCAATTGCAGGTAAAACAGTCA | AGCACTAAGCGAGGTAAGCAAG | chr17 | 7576760 | 7577031 |
| AMPL7153086974 | CAGGACTCAGAGGGTGCTAAAG | TTCAAGTGTGTAAACCCAACACACTA | chr12 | 49442826 | 49443069 |
| AMPL7152996970 | TGTGATGAGAGGTGGATGGGTA | CCTCATCTTGGGCCTGTGTTAT | chr17 | 7577376 | 7577637 |
| AMPL7153087179 | AGTCAGGGATGTCAGGCAACTA | TGCCTGAGTCTCCTACCTGATC | chr12 | 49415951 | 49416222 |
| AMPL7152996972 | ATTGCAAGCAAGGGTTCAAAGAC | GCCTTAGGCCCTTCAAAGCATT | chr17 | 7572830 | 7573103 |
| AMPL7153087228 | ACAAAGTTGTTCCATTACTTATCTGCTACA | CCAGGATTCTGGGAAAAATGCTTC | chr12 | 49448941 | 49449215 |
| AMPL7152996973 | GGGACTGTAGATGGGTGAAAAGA | AACTGTGAGTGGATCCATTGGAAG | chr17 | 7579587 | 7579842 |
| AMPL7153087718 | CTGCAGAAAGGAGTGGATCAGA | AGTAGAGCTCACCTCAGACACTT | chr12 | 49421714 | 49421987 |
| AMPL7152997067 | ACATGTGGTTTCTTGCCTTTGTAAAG | GCCCAGCCCATGTAATTTTGAC | chr11 | 108224396 | 108224668 |
| AMPL7153087740 | TCTTACTTGAAATCTCCATTGGCCAA | CTGAGATCTCATGCTAATTAGGCCTAAAAT | chr12 | 49423093 | 49423357 |
| AMPL7152997082 | TACTCAAACTATTGGGTGGATTTGTTTGT | TCCATCTTTCTCTAGAACTGAGTTTACAGT | chr11 | 108175364 | 108175636 |
| AMPL7153087778 | GTATGGCCAGGACAAGGAACTA | CAGTGTTGTAGCGTCATAGTAGACTG | chr12 | 49441669 | 49441936 |
| AMPL7152997088 | GAGAAATATGAAGTCTTCATGGATGTTTGC | CCAAACAACAAAGTGCTCAATCTACTATAT | chr11 | 108216494 | 108216680 |
| AMPL7153087791 | CCATCAAATAACTTGCCAGCTCCTAA | CCTGCAGGGCATGAAGAAAACT | chr12 | 49437342 | 49437610 |
| AMPL7152997092 | AAGCCTATGATGAGAACTCTTTAACAACAA | ACTATTGGTAACAGAAAAGCTGCACT | chr11 | 108191930 | 108192204 |
| AMPL7153087842 | GTGAAGGTCTCTTTGGCTCTTGA | GCAGCAGAGTCAGGCAGTA | chr12 | 49425524 | 49425795 |
| AMPL7152997093 | AACTCTGAGAAGTTTAAATGTTGGGTAGTT | TCTCTACAGAGAGTAACACAGCAAGA | chr11 | 108203426 | 108203684 |
| AMPL7153087870 | CCTCAGAAGATGATCCACTGCCTA | GGCCGTTAGCAATAGGAACTACC | chr12 | 49426109 | 49426348 |
| AMPL7152997100 | CCTTTCAGTGAGTTTTCTGAGTGCT | CGGGAAAAGAACTGTGGTTAAATATGAAAT | chr11 | 108143188 | 108143445 |
| AMPL7153087913 | CTGTTGAAACTGCTGCTGTTGTT | GCAGAGTCTGATGTCACACAGT | chr12 | 49426584 | 49426857 |
| AMPL7152997105 | GATACGAGATCGTGCTGTTCCA | GACACTGTAATCACTAGATTCTCTTTGTGT | chr11 | 108119559 | 108119740 |
| AMPL7153087928 | ATAAGGCCCTGACCCTGCTGTG | CAGACAAACCAAGCTCTGGGT | chr12 | 49426945 | 49427198 |
| AMPL7152997109 | TCAGAAAGAAACAGAATGTCTGAGAATAGC | GCGACAGTAATCTGTTAAGCCATTTATTTA | chr11 | 108099923 | 108100100 |
| AMPL7153087984 | AAATGTGTTAAACAGTCAACCGTACTAGT | CCATGCTGACTCAAGATTTGATAGTTAGA | chr4 | 153271072 | 153271345 |
| AMPL7153283910 | ACCATGATCTACAGGAACTTGGTAGT | AGAACCTCAGTATGTGGTTTTAGTTCATAT | chr12 | 69214115 | 69214335 |
| AMPL7154387903 | TCAGCCTGTGAAAGCGAACAATAT | GGTTTGGTGTTTTGAAGTAAGCATATTCTT | chr7 | 124510967 | 124511183 |
| AMPL7153320575 | CCAGTTCAAATCTAAAAGCTCAGAGTCT | TCCAGTAAGTTTAGCTGTGATGTGAC | chr1 | 27092647 | 27092921 |
| AMPL7154387904 | ACCTCCATGTTCAGCACATGAC | GCCACGAAGACCTGGAACTT | chr7 | 124475254 | 124475402 |
| AMPL7153322519 | AAACAAAAGTGTTGTCTTCATGCTAGTT | CTATATGTGATCCGCAGTTGACTGA | chr11 | 108167957 | 108168164 |
| AMPL7154387906 | CTGAATTTTAGATCTGAAAATAGCACCCAC | TATAAGCCCAGAAGACTATTTCAGTCTGTTA | chr7 | 124482645 | 124482919 |
| AMPL7153322790 | ATTCTGTTTATGAAGGAGTTATGTGTGTGT | CAACATACTGAAATAACCTCAGCACTACA | chr11 | 108186672 | 108186906 |
| AMPL7154387937 | GCAAAAGGAGTATTCTAACAAAACAGTGAC | AAAGGACGAAAAGTAGCAGTTCATTTTG | chr7 | 124480984 | 124481108 |
| AMPL7153322840 | GGTAATATATGCCTTTTGAGCTGTCTTGA | ACATTGAAGGTGTCAACCAATAAACTTCT | chr11 | 108158248 | 108158522 |
| AMPL7154390022 | ACTATAGTGAATGATTCCAGAGAGTCATGT | GGACAATGCAACCATTTTTAGGTCG | chr12 | 69233246 | 69233507 |
| AMPL7153323021 | CCCTAACCTGTGTTGTGCCTAA | GCTGAGGCTTACAAGGAATCCATAG | chr12 | 49447209 | 49447478 |
| AMPL7154392663 | GGAATGGCAGGCAAGAAAGGTA | GTTAAAGAGGAAGAAGAGAGTAGCAGTAAC | chr16 | 3817590 | 3817796 |
| AMPL7153323057 | CTGGCAACAGGGCCAAAGTGAG | GGTAGGTTGGGTGCTAAAGCTC | chr12 | 49440002 | 49440249 |
| AMPL7154392791 | GGCCAGTGGACAGTATTTTCTAGT | GAGATCCAAAACTGCTATACGCATC | chrX | 41200534 | 41200808 |
| AMPL7153323076 | TGGAGGCAAGCTTGGTTATGTC | CCTGTGGTGTCACCTGAACTTC | chr12 | 49436473 | 49436720 |
| AMPL7154392796 | CACCATCAGGTACAGACACCA | GCACTGGGAGTTCTCCTCCTT | chr16 | 3781090 | 3781343 |
| AMPL7153366671 | CGTTGCGGTCACACCCTT | TGGATGATGATGTTTTTGATGAAGGTCT | chr8 | 128750730 | 128750900 |
| AMPL7154392817 | AGTGAGCACATCAGGGATTTCC | CACCTTTCCCTCTCCCTAAAGC | chr1 | 27087406 | 27087626 |
| AMPL7153415379 | CCTCATAGCATGGACGCATTCT | AGGTCCTAATACACAAAAATGCTGGAAA | chr19 | 30308248 | 30308519 |
| AMPL7154392857 | CTTATGAACCAGAGAGCTGCTGTA | GCAGCTGGTTCTACTGCTTCAT | chr16 | 3843301 | 3843535 |
| AMPL7153415380 | CCATCGGCCATCTTCCTGG | CCTTCGCATCCCTGTGGAC | chr19 | 30303332 | 30303606 |
| AMPL7154392860 | GAGCTGAGTTTGGCTCTTTTGG | CTGTTGCTGTGGCTGAGATTTG | chr16 | 3929862 | 3930111 |
| AMPL7153415381 | GGAACGGAGCTCATAACCTGAT | AGTTCACTCTCACCAGAGTGTCT | chr19 | 30303778 | 30304052 |
| AMPL7154392867 | CCATTGAATTTCTTAACAGTTCAGTGATGT | TGCAAAACTGCATGACCCATAAAAA | chrX | 41202450 | 41202698 |
| AMPL7152997223 | ATTTTCCATCGCATGTGATTAAAGCAA | ATTTCAATTAATGCTGACAAGTAAAATACCATT | chr11 | 108159733 | 108160007 |
| AMPL7153087989 | CAATTTTGAACCTTACCCTCTTCTTTGC | GCCTTTCATAATTTTAGAAATCAGAGTGCA | chr4 | 153253732 | 153254006 |
| AMPL7152997235 | AGTATTCTTTACATGGCTTTTGGTCTTCT | CAGCTACTACCCAGCTAAAATTATCATCTT | chr11 | 108123487 | 108123721 |
| AMPL7153087992 | TAGAGGAAGAAGTCCCAACCATGA | TCACTTTTCCTTTCTACCCAAAAGTAATCA | chr4 | 153249320 | 153249589 |
| AMPL7152997270 | TGTATTCAGGAGCTTCCAAATAGTATGTTC | ATGGCATCTGTACAGTGTCTATAACAAAAT | chr11 | 108183077 | 108183309 |
| AMPL7153088016 | AGAAGAGGAGTGTCATATTATACAGTTTGC | TGGTGAAGGCAATTTACTCTTGAACT | chr4 | 153251784 | 153252058 |
| AMPL7152997291 | TCTTTGTTGCTTGGTTCTTTGTTTGT | AGCTATATGTTGTGAGATGCATCCTTATTT | chr11 | 108129676 | 108129905 |
| AMPL7153088027 | TAGTGATACAGGAAAATCACAAGAGCTTC | GGCCAGGATATTGAAGGTTGCT | chr12 | 49432917 | 49433191 |
| AMPL7152997331 | TGTTCTGGAATATGCTTTGGAAAGTAGG | GGATATTCATAGCAAGCATATGATAACAGC | chr11 | 108150132 | 108150388 |
| AMPL7153088050 | GTTTGGTATCTGGGAAGACTGAATGA | CGATTCTCCTGACAGCATTGTG | chr12 | 49422724 | 49422998 |
| AMPL7152997337 | AGTACCCATTAGAAAGACCTTCAGATAAGA | ATCCTAGGCCTCCCATCATCTT | chr11 | 108201990 | 108202264 |
| AMPL7153088094 | GGAGCGGAAAGAACTGAGGTAA | GCAAGACTACCCAGGTACCTGT | chr12 | 49435614 | 49435885 |
| AMPL7152997366 | TGGTTTGAGTGCCCTTTGCTAT | ACTTCACCCAACCAAATGGCAT | chr11 | 108206480 | 108206754 |
| AMPL7153088102 | GCCAATGTCAGTTCTTCCAACCT | ACTTTCGTTTAGTCTTTGCTGTCTGA | chr12 | 49446641 | 49446914 |
| AMPL7152997376 | TGACTGGCTTATTTGTATGATACTGGTTC | ATCTGAAAAACTGACAACAGGACCTT | chr11 | 108235714 | 108235988 |
| AMPL7153088125 | GTGACAGAAGAGATGGAGGCAAA | GGCCCTGATGACAAGAAGGATG | chr12 | 49438315 | 49438584 |
| AMPL7152997802 | TGAAAAGGCAGTTTATGGCAATTCATT | CCAGTCCATTTTCACCCTCCTT | chr7 | 148508592 | 148508866 |
| AMPL7153088150 | CCTACTCTCTCCCACAACACCA | CGGCTTCTCATCCACAACTCTG | chr12 | 49437855 | 49438120 |
| AMPL7152999903 | GGGAAGATCATCTGCTGGC | CCCTGTACGACCAGTACTGC | chr9 | 139399290 | 139399554 |
| AMPL7153089776 | AAAGTTGCGATACGCAGTCAATG | ATTTATGTTACCTTGCTTACTGAAGTCAGT | chr16 | 3819129 | 3819403 |
| AMPL7152999951 | CCAGTCGGAGACGTTGGAAT | TGGCGGTGCACACTATTCTG | chr9 | 139390595 | 139390839 |
| AMPL7153089857 | TAACACTGAGGGCCAAGGGTAA | ACCACAGCTGATGTTACCTCTTTTT | chr16 | 3860577 | 3860813 |
| AMPL7152999953 | CGGCTCTCCACTCAGGAAG | GTCCCAGATGATGAGCTACCAG | chr9 | 139390877 | 139391114 |
| AMPL7153089866 | GGAATGGAAAGAAGAAAGGGTTAGAAAGA | GGCTGTTTTCCCTTTTAAGTTTGAAGT | chr16 | 3827498 | 3827772 |
| AMPL7153415383 | AAGCCTATGGTAATTGAAGCCCAA | CTCCGCTGCAACAGACAGAAGAGA | chr19 | 30314491 | 30314755 |
| AMPL7154392879 | ACGCTTACCTATTCTGATAGCTGTAGTAG | TGCTATCCCAAAATGTCTTTAAAGCTACT | chr16 | 3807281 | 3807535 |
| AMPL7153415385 | CATGCCAGGGTACTGAGAAGTC | GCACTCACACTTCAGGTGGAAA | chr19 | 30312533 | 30312797 |
| AMPL7154392885 | ATCTTTGGCATTTTAAAGGAGTATGAGGT | GATCTTTACTGGAAGCTTGTCAAACTTAC | chr1 | 27105568 | 27105837 |
| AMPL7153415390 | GCAGCTGGAGTGAGGAACTCTA | CTTAATCTGAGGCTGACAACTGGA | chr19 | 30313076 | 30313331 |
| AMPL7154392888 | GCGTCTGTGTGTCCAATACCATT | TCCTCCTCCTTTTCATAAGTTAGTGGT | chr1 | 27106331 | 27106496 |
| AMPL7153433118 | CCCTCCTCTTAGAGTCCAGATGT | GCGTTCCATCAGATATGTATTCTCACA | chr7 | 2985408 | 2985640 |
| AMPL7154392889 | CGCCTGGAGAAGTTGTATAGCA | GCCGCATCATGTCCACACTA | chr1 | 27106816 | 27107086 |
| AMPL7153454452 | TCCAGATGGCTGGTCATTAACAC | GATGTTGTCTCCAAGCAAATAAAAACCTTA | chr2 | 198270050 | 198270322 |
| AMPL7154392911 | GAGATGTACAGCGTGCCATACA | CCAAACTGGAATGGAAATTGGTTCTG | chr1 | 27100877 | 27101097 |
| AMPL7153464594 | CCGGGTCCATTTCGTTATACAGG | CAATGCTCTAAGTCCTGTGACCAT | chr19 | 13410049 | 13410201 |
| AMPL7154392914 | CGTAATGACATGACCTATAATTATGCCAAC | GAATGTGATTCTGCATGCTTGGT | chr1 | 27101219 | 27101465 |
| AMPL7153469704 | GTTAACCGTGAGGCAGTTCTGT | TAACCCGGAGCCCTTTGCT | chr19 | 13616814 | 13617066 |
| AMPL7154392929 | AGTTCGTCCTTGCGTGGTTTAT | CTCTGCCAAAAACAAAAGAACAAAACAAAA | chrX | 41203509 | 41203716 |
| AMPL7153510598 | TCAGTGGGAAGAGATCAGTGGT | GTAAACTGATGCTCTCGAAATTTCACC | chr3 | 89259006 | 89259278 |
| AMPL7154392930 | GGCCATTGAGAGGGCTTTCTAA | CTGTACTGCCAACTCTCTCGTT | chrX | 41203121 | 41203360 |
| AMPL7153515406 | CAGACTGCATCAAAGTGGGAAGA | TTCTTACTCTTAAGGATCTCTCTTTGTCCA | chr3 | 93611730 | 93612004 |
| AMPL7154392934 | ACTTTTCCTCCTCAATTCAAAACATGC | AGAATCTGCACCCTTTTTGGTCT | chrX | 41205328 | 41205534 |
| AMPL7153538914 | CCGAAACAAAGCGAGAAGTAACTG | TGAGCTCTGAAGTCAGTCGGAA | chr18 | 3214842 | 3214989 |
| AMPL7154392937 | CCAGGCAGCCAAACTATAATGC | GTTTCTTGGGTTTTCCGGTTCATG | chr1 | 27089473 | 27089734 |
| AMPL7153580954 | CTTCACAACCAGCCCGTATTTG | CTCTCCCATAAAACCTGTCTTGCA | chr18 | 3067371 | 3067578 |
| AMPL7154392942 | CCTGCTCGGTTGATCTTGGATG | GTGAGACTGCTTTATTAAGCTTGTTGG | chr7 | 2987208 | 2987464 |
| AMPL7153584994 | ATCAGCTGACACCCAGCTTT | GCAAAGGACTGGAGTCATGTG | chr19 | 1440310 | 1440502 |
| AMPL7154392947 | GGTGTTAGATGAAGCTGATCGGAT | CAACTCTTCCTACAGCCAAGAAGA | chrX | 41204439 | 41204711 |
| AMPL7152999955 | GCAGAGGGTTGTATTGGTTCG | CATCGGGCACCTGAACGT | chr9 | 139391212 | 139391462 |
| AMPL7153089901 | TCTCTCTTAATCGCTGAATTCTTGCTG | GACGCACACACAGACTTCTACA | chr16 | 3795161 | 3795435 |
| AMPL7153005387 | GCGAACAGTGAATATTTCCTTTGATGA | AAGGTTTTCTTTTTCTGTTTGGCTTGA | chr7 | 140481274 | 140481547 |
| AMPL7153090134 | GAGCATTGCACTCTGTTCGG | ATCTCCCTGGCCAGCAGATCG | chr16 | 3777821 | 3778062 |
| AMPL7153022223 | GCGGAGAGTTCGGCATGA | CGCGGTTGTAGTCCTGCTT | chr6 | 393178 | 393345 |
| AMPL7153090169 | GGTGAGATGCTCCTGGGTG | AACATCAACAACAGCATGCCC | chr16 | 3778857 | 3779122 |
| AMPL7153022990 | GCGACGAAGCCATGAATCTCAT | CCTTGGACTGCTCTCTCTCAAG | chr6 | 106553663 | 106553836 |
| AMPL7153090196 | CTGCCACACATTTAGAAAGAATCAGTTT | TGGCATGTTGGTTATCTGTCATCA | chr16 | 3831116 | 3831390 |
| AMPL7153030879 | ATGTGTTACGATGCCTTACGGAA | GTCAGACATAATGCATGCTGAACTTAC | chr11 | 108121553 | 108121826 |
| AMPL7153090312 | AAAAACATTGCAAGGAAGTCAAAATGG | TTGGGTGGCTGTGTGTTATGAT | chr16 | 3801607 | 3801866 |
| AMPL7153031100 | GTTGTGCCCTTCTCTTAGTGTTAATGA | TCCAAGAGCTTCTTCATTTAACGTAACTC | chr11 | 108139093 | 108139367 |
| AMPL7153090348 | CGCCTTGTCCAGCATCTTTTTG | TCCTCTGCTATAACTCCTTAAAGGCA | chr16 | 3786681 | 3786893 |
| AMPL7153031863 | TCTTTAATGAAATCTGTGCCTCTGTGT | AAAGCAAATCAATCAAATATACCATGTGCA | chr13 | 48941547 | 48941773 |
| AMPL7153090372 | TCCTATTCCTGGGTTGATACTAGAGC | GTTGCTTAGTTTCTCATTTCCATTTCTGTT | chr16 | 3900835 | 3901050 |
| AMPL7153031887 | AGCAGGCTCTTATTTTTCTTTTTGTTTGT | TGATGCCTTGACCTCCTGATCT | chr13 | 48953695 | 48953894 |
| AMPL7153090393 | GGGAGATGGAGACCCTGCATAT | GCTGCCCTTGTAGGCCTAAAAG | chr17 | 62007047 | 62007314 |
| AMPL7153031914 | TACCTGGGAAAATTATGCTTACTAATGTGG | AAAATCTATTTGCAGTTTGAATGGTCAACA | chr13 | 49027073 | 49027296 |
| AMPL7153090421 | ACCATAGTCGGACACAGGACAT | AGTAGCTCCGGGAACATAGAGG | chr17 | 62008627 | 62008839 |
| AMPL7153031974 | ATAGCATAAAGTAAGTCATCGAAAGCATCA | ACCTGCCAACTGAAGAAATTATACATTCTT | chr13 | 49051391 | 49051644 |
| AMPL7153090488 | CTCACCTACAGACCACTTCACTTC | GATCACACCTACGAGGTAAGGAGA | chr17 | 62006604 | 62006808 |
| AMPL7153032313 | CCCACTGTGCTGTTTCTGTAGG | GCAAGACTCTCCTTTCTAGACACAC | chr8 | 42146131 | 42146323 |
| AMPL7153091072 | TCACTGACAATGCATATTATTTCTACTGCT | TGGCATTCTCTGCTCTCTCTCTA | chr18 | 60795784 | 60796055 |
| AMPL7153032321 | GTCACATGGGCTGGTAAGAGAC | AGAGACCAAAGGATTGAAGTGTCTG | chr8 | 42150876 | 42151136 |
| AMPL7153095352 | GTACCAAAGAACTGCTGAAGATCTCT | CAGACAGTGATGAACCTCAGGAT | chr3 | 38182433 | 38182707 |
| AMPL7153584995 | CACAAAACTGCCTGGTGCA | GCACACACCTTGATCTCCACC | chr19 | 1439989 | 1440260 |
| AMPL7154392988 | CCTAAGAACTGTGGTTCTACAAAGATGA | GTTCAATCTTGCATTCAAAGGCATAGA | chr1 | 27097494 | 27097736 |
| AMPL7153584997 | CATGTCCGGGTCCTCGTAAC | CGCCCTTACTAGGACATGTCCA | chr19 | 1438626 | 1438900 |
| AMPL7154392990 | AAGAGACTTCTGAGACCCTTAGCA | CCACCAATCTCCTTCACAGACA | chr1 | 27094207 | 27094480 |
| AMPL7153602957 | GTCAGCATCCCAACCAGAGGAT | GGATAGCACCATGATGACTGACA | chr6 | 138202290 | 138202524 |
| AMPL7154392992 | CCTCATGCCCAACCTTCGTAT | TGCTGCTGGTAAGGAGACTGA | chr1 | 27057772 | 27057980 |
| AMPL7153605173 | CAGCAATATCCAAGGGACTGCAT | GGGATTCCCTATACTGAGACCATTTTT | chr16 | 3807820 | 3808087 |
| AMPL7154393071 | CTGATGCCTTGGGATGGAACA | GTCCAATGACCTGTCCCAGAA | chr16 | 3781592 | 3781828 |
| AMPL7153605180 | GGAGCTCAGAGAAGGGTCTGTA | CCTCATCTCACTGTTGTGCTTTG | chr16 | 3786014 | 3786236 |
| AMPL7154393088 | TTTTTCTTTCAGCATACCACTTCATAACTG | GCAAAGTGGGAAGAATTTCATCTCCTA | chr11 | 108115424 | 108115639 |
| AMPL7153622716 | GGTGCAATGCCTCAGGAAGT | CATCGTGTTGTTGTGTCTGCA | chr12 | 49446013 | 49446229 |
| AMPL7154409890 | CCTCATCTTGTTCACCAGCATGT | AAGTTGTTGGAGTAGAACCTAGACCT | chr4 | 153332625 | 153332783 |
| AMPL7153704010 | AAAAATGAAAAGAAAAACAAGGCTTATATAGGT | TTCTGATGCACTTTAGGAGTGCATT | chr3 | 93597959 | 93598214 |
| AMPL7154410392 | TTTTTGGACTGTACTGGATCAGCA | CATGTTTTGATGGGTCATGTTGCA | chr4 | 153247056 | 153247319 |
| AMPL7153704013 | TTACCATGGGTGTACTTTACCTACAGA | TACAGGAGCATAAATGTCCTACCTCTT | chr3 | 93629395 | 93629652 |
| AMPL7154410395 | TCATATAAAGAGAATCAACATGTGGCCTTT | GACGCCGAATTACATCTGTCCA | chr4 | 153267900 | 153268143 |
| AMPL7153723958 | CGCTTTACAGAGGAGGAGACAAAG | AAGAAGGAGCAGGTTCTGGAAC | chr7 | 2979212 | 2979463 |
| AMPL7154410397 | ACATTTCCTCAAGAGTAGACTAGTTCTGT | TATTCTTGTCTCTGGGAATGCAGATTC | chr4 | 153245163 | 153245415 |
| AMPL7153804252 | TCAAACACTGATCTAAGGTGGAGAGA | AGGGCATTTAGAGTAATTAGATGACTGATT | chr4 | 87705480 | 87705752 |
| AMPL7154410400 | GGAAGGGCAGGGAGTATATCGT | CAGCTCAGATGATGGAACTGTAAAACTA | chr4 | 153243977 | 153244240 |
| AMPL7153804266 | CAACATCACTTCCAATAGTGGTATAGCT | TGCTCTGTGATCTTTATTTTGGATAGAGAG | chr4 | 87735519 | 87735793 |
| AMPL7154410407 | CTTGTAATACTTTCCATTACCTTCTGTAAGACA | CAGAGAAGATGAACATACACATACTAACAGT | chr4 | 153332288 | 153332560 |
| AMPL7153804269 | TATGTGGAATAGCTAATGAGTTTGCTTTCT | CGTGGTTTCTTAAAAACAGTTTGCTTG | chr4 | 87637628 | 87637822 |
| AMPL7154412509 | AACGTGTTTTGATCAAAGAAGAGGAGTA | TGGACCCATTACATTAGATTTTTCCAATCA | chr13 | 49039141 | 49039296 |
| AMPL7153032330 | CCTCGTAGAAGGACATTGTGTTCT | CATGCATCTCCAAAACATTCAGAGAG | chr8 | 42163802 | 42164076 |
| AMPL7153099141 | TCCCTTGCTAGAGCTCACTGTA | TGAGCAGTGGTCATTTTATGAATCAGTAAA | chr22 | 22123447 | 22123694 |
| AMPL7153032350 | TATACTGGGAGACGCACACTGTA | GTCCTAAGAACTGCATGTTCCACT | chr8 | 42173654 | 42173927 |
| AMPL7153099155 | AACAAGGTTACCAAGCAGTGGAA | TCTGAGGGCTGTTTTTAATGCCA | chr22 | 22161905 | 22162179 |
| AMPL7153032359 | GGGAAAGCTGTGGAACTTCTTC | CCCTGCCTTGTCCCACATATTA | chr8 | 42175090 | 42175352 |
| AMPL7153099204 | GTGACCTAACAATTTAACAAATAAACGCGA | CTCAGAGTTGATGGTGTGTAGTCATTTA | chr22 | 22142474 | 22142747 |
| AMPL7153032373 | GGGTTTTGTTATTGTACAGGAAATGGA | AAAAATGGTGTCTTTAAACTCATTGCAAAG | chr8 | 42176713 | 42176980 |
| AMPL7153101671 | ATACTGTGAATAGTGGCTTATTGGCAA | ACTTTCCCTGTCTCTGTGGACT | chr1 | 120464822 | 120465096 |
| AMPL7153032382 | CGCCTACCACATCAGTTGACAT | GGAAGCATGCTCTAGAGAATGGTC | chr8 | 42178211 | 42178482 |
| AMPL7153105646 | CCCTCATCCCTTTCAGGGAAGA | GCCATTCCCTAAATGTGGATTCCTAG | chr12 | 49415496 | 49415765 |
| AMPL7153032391 | CTGCCTTGGGCTTCTCCTTATC | GAGGGAGGAAGGTGACCTCATA | chr8 | 42179482 | 42179742 |
| AMPL7153106388 | CCCACCCTAATCCTGTGTTTCTT | GCTTCTTAGACTTCAACACGTTAGATTAGT | chr1 | 27100258 | 27100479 |
| AMPL7153032402 | GTGGAAGAGGTGGTGAGCTTAA | AGTCAAAGAACAGCGACGTGAA | chr8 | 42179911 | 42180163 |
| AMPL7153106398 | CGTTCGTGTGTTTGTGTGAGAGTTA | GCAAGGAGTTCCCATGCACTTA | chr1 | 27098918 | 27099190 |
| AMPL7153032422 | GTTGACTTTCAGGCCCTAGACT | GAGCTCCATCTGGATACCAGAGA | chr8 | 42188420 | 42188685 |
| AMPL7153106408 | TTGTGTTATCTTCAGAGTAGCTTCACTG | GAAGATCCCAAACCCTCTCAATCTT | chr1 | 27101969 | 27102243 |
| AMPL7153032531 | ACCAGACATGTATTTTGACTTTTCGTTCT | GCCTCTGTTAGGTGACCCAAAT | chr6 | 394773 | 395045 |
| AMPL7153106511 | GGTAGATTACCAGGCTTGTCAACT | CAGATTGAGCCCACTATAGCTTCAG | chr1 | 27099777 | 27100051 |
| AMPL7153032875 | AAGCTAACAGAAGGCCTATATTGTGAAAA | CCTTTGATCTGGCCGTAGTCCTA | chr14 | 103363497 | 103363771 |
| AMPL7153106542 | AGAGCATTTGTTCGCATTGTATAAAGC | TGTTTTCTCCTCTCACCCGTATCT | chr1 | 27088585 | 27088859 |
| AMPL7153032889 | CCCTTTACAGCCAGCCTTTCTA | TGAATGCATCTCCCAAATGACGT | chr14 | 103371730 | 103371951 |
| AMPL7153106617 | CATAACCCTTTCACAGTGAAGTAAGC | CTTCCAACCAGAAACTAACTAGTTTGC | chr1 | 27059088 | 27059362 |
| AMPL7153033306 | TCATAGGGCACCACCACACTAT | TACAAGCAGTCACAGCACATGA | chr17 | 7578187 | 7578443 |
| AMPL7153106630 | GCGATAACCTCGTTCTTCTCGT | TTAAGAGGGAAGAAACCAGAAAAATGGAG | chr3 | 183209860 | 183210077 |
| AMPL7153804271 | GTGACTGAGCAATTAACACACTTTGT | TGCCAATGCTATGCTATAGACTTTTCC | chr4 | 87679304 | 87679578 |
| AMPL7154412512 | ACCAGTACCAAAGTTGATAATGCTATGTC | GAGCTAACATTAAAAGGGACAAGTCTAAGA | chr13 | 48919253 | 48919472 |
| AMPL7153804274 | CCCAGTATTCAAAATCAAAGGCCAA | GTTAAAGGTAACAACACCAAACAAAAATGG | chr4 | 87655838 | 87656083 |
| AMPL7154414766 | GAAACAGAAGAACCTGATTTTACTGCATT | GAGGTAAATTTCCTCTGGGTAATGGAATTA | chr13 | 48881429 | 48881686 |
| AMPL7153804276 | TGCAAGTATAGTTAAGTTGCTGATGCA | CAAAAATCTAATCTCTGACCCTGTATCTGT | chr4 | 87556350 | 87556612 |
| AMPL7154415261 | GCAACAAACATGACAATTTAACAAACTGG | AATCAACACTTAGTCCAGAAGAGCAAA | chr2 | 198269764 | 198269888 |
| AMPL7153804286 | AACTTAATGTAAGGCATACTCCATTTCTGT | GTGGCAAATCCAAAGATATAAATGGTTCAA | chr4 | 87653342 | 87653616 |
| AMPL7154415269 | AAATCTTTAACTTACCTGTAAACAATATTGCAA | CTTGTTGAACTATGTATGGCCCAATG | chr2 | 198257681 | 198257822 |
| AMPL7153804288 | ACTTGTTACTCTCATTGATGGATTTTGACT | CATTGACCTGGCTTTGAGACAAG | chr4 | 87683845 | 87684113 |
| AMPL7154418460 | CCATAAATTCTCAGAACAAAGCATTTCCA | AAGTGACAGCAGATTTGCTGGATA | chr2 | 198288340 | 198288589 |
| AMPL7153804303 | TGCCTTCTTTTGCAGGCAAATG | CAGGAAAGAATCTCTGATTATTTCACCTTC | chr4 | 87706916 | 87707181 |
| AMPL7154418471 | CGTCCTGGGAACCAATGTAGAT | CAGCTTAAGAGCAGTGTAATTAGTAGGTTC | chr2 | 198257107 | 198257334 |
| AMPL7153804313 | TGTAGAGAAAATCATTCAGGGCCATG | AAACAGCATATCCATATAGTCTGAAAATCACTT | chr4 | 87720173 | 87720447 |
| AMPL7154418486 | CCAGGAGTCTGATCAGCTGTTT | CGCTAATGGTAGAAAAATACATTGAAAGCA | chr2 | 198281505 | 198281697 |
| AMPL7153804318 | CATCTGTATTTAACACTCAGAATCAGCCTT | ACTGATCACATATTAAATCTGAAGCCAAAC | chr4 | 87726306 | 87726562 |
| AMPL7154418489 | AGACAGCAAAACCTTGTGTCAAAAAG | TCAGAAGAAGCCAGGATATCATGC | chr2 | 198285588 | 198285819 |
| AMPL7153853154 | CAGCAATTATTGCAGAACGTCTGT | GGATATTAAAGTTTGTGTGCGTGTGTT | chr3 | 93617179 | 93617453 |
| AMPL7154418502 | TCCTAAGACTCCAGGCTAGCAA | TGTTAAAATCGCTTTCCTTCTTGTTTGAAT | chr2 | 198260647 | 198260879 |
| AMPL7153854706 | GCAGAGTCTTGCCCTGAGTATT | CCACATGATGGACTGATAGAATTGTGATAT | chr11 | 108196889 | 108197163 |
| AMPL7154418548 | AATGAAGAGAATACTCATTGCTGATTACGT | GGATGCAGAATATGCCAACTACTATACTAG | chr2 | 198266292 | 198266565 |
| AMPL7153854717 | GAAAGTGTCCTATTTGTAGGAGTACAATCA | CGTTCATAAGAACAAAAGCCTATCATTCTC | chr11 | 102207775 | 102208045 |
| AMPL7154418560 | CATTCCAATAATCAGAAGGTCAGTGGTT | GCTATTCGTAGAGCCACAGTCAAC | chr2 | 198262549 | 198262761 |
| AMPL7153854790 | CCGAAGCTGTAGTCCAGGAT | CGTGTGAGAATGACGCTCGTA | chr9 | 139400050 | 139400317 |
| AMPL7154418579 | GCGATTTCTCTCATCAATTTCTCTTTCC | TTTTTCCTTTGTGGTATTCTGTGTACTATT | chr2 | 198272770 | 198273040 |
| AMPL7153034365 | TGCCAGAAGACTTTTTGAAAGCTTC | GTAGCCAGGGTAGGAGCCCAAA | chr6 | 106552949 | 106553211 |
| AMPL7153106737 | CTTACGTTTGGGTTACTTCTTTGACC | AGCATTTAATTCATCAAAACCTTGCAAGA | chr18 | 50831866 | 50832139 |
| AMPL7153034380 | GCATCAACAACTTTGGCCTCTT | GCTACAGGCCTTGTCCTTCATG | chr6 | 106553333 | 106553568 |
| AMPL7153106749 | GTTTCTTCTGTGCTGTTTTATCAAACTTTG | GAATAATTTTCATTCCAGGTACCAAAGCA | chr18 | 50592310 | 50592583 |
| AMPL7153034438 | TTTTTCCTGTTTAGGTTATTGGAGTGATG | ATGGGTCTGAAGAAATTTCCCTTACTTAC | chr6 | 106543476 | 106543638 |
| AMPL7153106765 | CCCGAAGGTGTTGGCTGAAATA | GCCAAATGAAATGAGAAGGGAAGT | chr18 | 49867137 | 49867319 |
| AMPL7153034676 | TCCAGGTTGTTGCTGTCCTT | AGCCTTCGTCCCGCTTCTC | chr7 | 2976675 | 2976895 |
| AMPL7153106816 | CCTTGAAGAGGGCATGTTTCTCA | CATTTGTCCAGAAAGTTCAGGGAAAA | chr18 | 50994223 | 50994497 |
| AMPL7153034723 | GGGTCTGATCTAGTCCCTGGAA | CCATCCTGGACATCTTGGAACAC | chr7 | 2978189 | 2978431 |
| AMPL7153106997 | TTATTTCAAGAGGGCACTAGCTTTGAA | AGTGTCTGCTTCATCAGGTGTATG | chr18 | 50942371 | 50942645 |
| AMPL7153035184 | CTTCCTGCACAGTCGGTAGAAG | GGATGTGGGAACCCTTACCTCT | chr17 | 43344631 | 43344886 |
| AMPL7153107123 | GCCGTCTTCCACCAACAACAT | GGTAGTAGGAGTTGTACTGGTGGTT | chr1 | 27023393 | 27023543 |
| AMPL7153037714 | CCCGTTCGGATCCTTTCCT | GGCGTAGGCCACTGCTTAC | chr5 | 137801504 | 137801776 |
| AMPL7153107138 | CTGGGAAAGGAGCTGCAGGAC | TTGCCCGAAGCCGTAGG | chr1 | 27023099 | 27023350 |
| AMPL7153037771 | CAACAGTGGCAACACCTTGTG | GCTCAGGGAAAATGTCAGTGTTC | chr5 | 137802567 | 137802796 |
| AMPL7153107175 | TCCTCGTCGTCTTCGTCCTT | ACTGCGAGGCCATGTCC | chr1 | 27023672 | 27023900 |
| AMPL7153038355 | GGGAGAATGTGGACTGGGTAGA | GTTTCTACTGCGACATTAGCCAAAAA | chr6 | 106534386 | 106534583 |
| AMPL7153115461 | GACATCCAGTTTGTGCAGGAGAT | CCCTGGGAATAGCTTCAGGAAG | chr3 | 38181926 | 38182194 |
| AMPL7153038731 | ACAACTTGGGATGGGAAGCAAA | TGAAAGCACCAGCTGGTAAAGT | chr22 | 29194958 | 29195227 |
| AMPL7153117862 | TCTAGAAGCTGGCTCCAGAGAT | TGACCAGTGGTCAAGTTCATCAC | chr1 | 120457818 | 120458079 |
| AMPL7153038778 | CAAGCATCAAACAGATGGAATTAACTGG | GCTGTCTAGCAAGCATTTCCTTG | chr22 | 29191971 | 29192244 |
| AMPL7153118902 | ATGAGTATTTGGCTATAGTCTAAAGTGTTCATT | CACTGGTAGATCTAGAAAGAGGAAAGAATTTTT | chr11 | 102207387 | 102207650 |
| AMPL7153054652 | GCTGCTGCCTGGACTGTAG | GACACCACCTGGGATGACT | chr16 | 3820686 | 3820835 |
| AMPL7153124122 | CAACAACAGAAAACCTGAATAATGCATATG | CATGTTGTAATACTGCCTAACTGGAAATG | chr3 | 176768176 | 176768450 |
| AMPL7153854844 | GCCTTTATGCCATGAGTGCTCA | GTGCTGCACATTCAGTGTGAAG | chr6 | 138199785 | 138200055 |
| AMPL7154418592 | TCTGTTCTTGGAAAGCATAAAGAATACCAT | CCTTGGAAAAGCAGTCTAAAAGGTTTTT | chr2 | 198265447 | 198265708 |
| AMPL7153856665 | ATCTGACTAGCTGGTGTTTCATCC | TCAAACAACTTTAAATGGGTCACCATTTT | chr2 | 198273173 | 198273446 |
| AMPL7154418595 | TTTTTCTTGTCCTTCTACCTGGAAAGA | AGGCGGACCATGATAATTTCCC | chr2 | 198284938 | 198285200 |
| AMPL7153864950 | CCTGGAGGTCTTTCTGGAGAAC | GTCCAGGGAGAAGTAGCTGTTG | chr4 | 134071577 | 134071805 |
| AMPL7154418602 | TATGAACCTCTTACGGCAAAGATGAC | CAAGAGCGTCATTTACTTGTGAAAGTTATT | chr2 | 198268124 | 198268389 |
| AMPL7153890445 | AAGTCAATATCCAGTGCCTAACAAGAAA | GTCATCTGAAATAGTCTGCTTCTCCTT | chr4 | 87666036 | 87666310 |
| AMPL7154422319 | ACAGAGGCTGGGAAAGGATGATA | CCTGAAGGGAAGCACATAACCAC | chr1 | 120458206 | 120458480 |
| AMPL7153912047 | GACATGAGCGAGTCCTCCG | CCACCTGAGGACTCGCCTAT | chr12 | 49445316 | 49445581 |
| AMPL7154422321 | AACATCTCATTGTACTGGGTCTCATTC | GCCCAGCATGCACTATCTTTTTCT | chr1 | 120458542 | 120458762 |
| AMPL7153934092 | CCAGCTTGTACCTGCAGGAT | GGGCTTCGCTTACCAGAGT | chr8 | 128751029 | 128751278 |
| AMPL7154422323 | GGGATAAAGTTACTGAACTCTCAGACA | GCCTTCTGGATGAATACAATGTGAC | chr1 | 120458897 | 120459166 |
| AMPL7153977811 | TTGAAGGAGGAGCTGCTCAAAG | TTCCACCCAAGGGAAGTACAGAG | chr11 | 9685736 | 9685894 |
| AMPL7154422331 | TAGCCTTGAAGTTCAGAAACCAAACA | CTGAGAACCTGGCAGAAGGTAC | chr1 | 120466229 | 120466503 |
| AMPL7153977830 | GAAAGCAGAACAATTATGCAAGAGGT | TGGAAAATTATCTTCCTTCTTTTCCTGCA | chr4 | 74442253 | 74442519 |
| AMPL7154429525 | GCGTCTTGCTGGGTCTGTT | CCCAGACCGTAAGCCCTTTC | chr10 | 64573398 | 64573669 |
| AMPL7153977840 | CTGTTTCAGAAGAGTGGATTTGTGTG | TTTTTGCCCTATAAGAAGATTATGTTCAAGATA | chr4 | 74447824 | 74448097 |
| AMPL7154429527 | CCAGCTGTACCATGTAGGTCT | ACCTGGACCACCTGTACTCTC | chr10 | 64573670 | 64573919 |
| AMPL7153978102 | CTTATATCGGACTGATTTGTTGGAGTTTTT | CAGGTCTATTAAATGCTAAAATGACCAAGT | chr11 | 9734967 | 9735241 |
| AMPL7154429529 | CTCACAATATTGATTATGCCTTCTGGGTA | GTATTTTCATTGTTCCACCTCCATTCTG | chr10 | 64574048 | 64574272 |
| AMPL7153985444 | GTATGGAACAGCCACTCCTCAT | TCAGGGAGTGATCAGTGAGTATGG | chr12 | 49428040 | 49428206 |
| AMPL7154429530 | CCACTCCGTTCATCTGGTCAAA | GGCTTTTCTGACACTCCAGGTA | chr10 | 64575627 | 64575848 |
| AMPL7154014115 | CTGGGAGGAGAAGATGCCCG | GTTGCTTTTCCTCTGGGAAGG | chr18 | 60985744 | 60985920 |
| AMPL7154505601 | GTGGTCACTTTCAAGGAAGACAGT | CTTGGACACCAGATGCAGGAA | chr4 | 126237987 | 126238181 |
| AMPL7153056929 | GGGCACTTTGCACTGGAACTTA | AGGAATGGGAGAAAAGACACCCTA | chr8 | 128748695 | 128748968 |
| AMPL7153124368 | TAAGGTGCTAGCAAGGTTACCTAAAATG | TCTTCATTCTCTTCTGTACCTGGGAT | chr3 | 176765166 | 176765440 |
| AMPL7153059517 | TGACTTTCCTTCTCTTCTCCTCCTT | CATGGAGCTCTGTTAGTAGATAATTAGGG | chr6 | 138195946 | 138196213 |
| AMPL7153125712 | ACTCACCTCCAGGTTCTCCTAG | GGTACTGATGCTTGTGTGTCCA | chr12 | 49448305 | 49448559 |
| AMPL7153059644 | CTATGTGGTACTAACTAGCATCCATTCTC | CTCCAGCAAAAAGCATCGAACACAC | chr6 | 138201173 | 138201447 |
| AMPL7153131340 | CATGCTGCCTCCTAGGAAGTG | CACCATACTGCTCCACACTGT | chr7 | 65557671 | 65557819 |
| AMPL7153063324 | TCACATTCTCATTAAGGGTTGGTATTAGC | GCCCAATGAAATCTTAAGATGTTAAGCTC | chrX | 41196510 | 41196782 |
| AMPL7153150566 | TGCAGATGAGCAGTAGTGGGTA | CATGGAAAAATTCACCCACTGAGATG | chr16 | 81960616 | 81960879 |
| AMPL7153063872 | TGTTTTCTTTTAAGTGGGCCATATCTCA | GTTCTTTCCTGCACACATCACAG | chrX | 41205713 | 41205936 |
| AMPL7153158682 | GGAGTACGGGTACCTCCAATGT | CTCGGAGCGTGTTTTTATAAAAGTCC | chr2 | 136875504 | 136875764 |
| AMPL7153063956 | CTTTAGTGGAATTTCATCTTCATGTGAACC | AAAAACCCTTCCCAAAGCTATGAAG | chrX | 41206514 | 41206746 |
| AMPL7153164265 | CCACTCACAGCCTGAAGCATAC | ATACCCAGGTAGACAGCAGACA | chr19 | 42381191 | 42381457 |
| AMPL7153069842 | CCTAACTCTCCTTACACACACTCTCT | GTTTTCCCTTTCAGTTTTGAGTAAGTTCTT | chrX | 70471293 | 70471567 |
| AMPL7153164310 | GCATCCAGGAGGGTCTGAAAGATA | GAAATGAGGCAGCGAACATCAG | chr19 | 42384665 | 42384925 |
| AMPL7153069883 | CAATGTGCACAAGTCATCTTGGT | GGTGGCCTTTCTCATGTCCATC | chrX | 70470414 | 70470613 |
| AMPL7153185311 | CAGAGGCACAGGGATGAGTTAG | GTCGAGAGATTCAACTTCAAATTCTACACT | chr12 | 69229525 | 69229707 |
| AMPL7153069956 | CCCAATTCTCCAACCCTCTTTATGT | TACACCTTCTGTGTGCTCTCTCT | chrX | 70468472 | 70468723 |
| AMPL7153185434 | CCCTTTATTGAACTTGATGGATATGTTTGC | GCCAATTTCTCCACATGGTCTTG | chr12 | 69207242 | 69207493 |
| AMPL7153070035 | GGATCTGCAAAGACAGAGGAACA | CCTGTTAATGAAGCTCTCCTGGAA | chrX | 70466359 | 70466630 |
| AMPL7153192727 | CTGCCAGGGCACAAACAGGTAT | TTGATGTGTTCCTTCTAACTCCAAGAC | chr6 | 44230247 | 44230485 |
| AMPL7153070121 | TCCTGATGGCACAAATGCTACC | ATCAGTCCCTGAAAGCTTGTGAG | chrX | 70465134 | 70465407 |
| AMPL7153192740 | CAAGGACAGGGTGTAGGTGA | GAGGAGAGCCAGTACGACTCT | chr6 | 44232832 | 44233050 |
| AMPL7153070184 | CTGCCTGCTGTGTTAAGAGTGA | AGCTAAGTGAAATTCTCCATGTAGAGTTTT | chrX | 70463628 | 70463895 |
| AMPL7153192809 | CCTGGGCTCTGGATAAGGAAGT | GGAGTTGGTGGATGCAGATCAG | chr6 | 44228071 | 44228330 |
| AMPL7154030564 | CCTGAGCAGAGCCTTAGAACTG | GTGACTGCTCCCTCAACTTCAA | chr9 | 139399662 | 139399918 |
| AMPL7154505604 | GTAAACGTGACTGTGCAAGACATTAAT | TGCACCGTAAGCGAGTATTGG | chr4 | 126238272 | 126238537 |
| AMPL7154030569 | TGAGCGAGCTCCCTAGGAA | GGTTCCCTGAGGGCTTCAAA | chr9 | 139396570 | 139396805 |
| AMPL7154505606 | CGGTAGTGAAGTTCCGCTACTTC | GCTGCTTTGCACTTCAAAGTGG | chr4 | 126238618 | 126238802 |
| AMPL7154034295 | TTCCATATAGTTATCCATTTTGAACCTGGA | AGTCTATAATTCTCTCCAGTTGCTAGGATT | chr11 | 102201720 | 102201883 |
| AMPL7154505610 | GCAAGCCTGGTGATTTTTGTTAATGA | GCTGTAACGCAGATTAGCATTGAGA | chr4 | 126238941 | 126239105 |
| AMPL7154042250 | CTGAAGGAGTGGCGAACACT | CCATCCTGGAGACACCCATCA | chr12 | 49444284 | 49444444 |
| AMPL7154505614 | CCTATGCCCAGCTTGTAGTAACTC | GTAGAAGGCTTGCTCTTCTCTGTC | chr4 | 126239257 | 126239531 |
| AMPL7154142081 | GGCGGAGTGCCATTCAGA | CTCGCAGTGCTTCCAGAGT | chr9 | 139397470 | 139397734 |
| AMPL7154505626 | CCACAGCTCTCCTCTAGTGTCA | AGTTCACGGTCCAGTTCACTTTTTATATAC | chr4 | 126240486 | 126240751 |
| AMPL7154167590 | GTGTGAGCCGCAAACCCAG | CCTTCAGAAGAGCTCCTTTCCC | chr2 | 61108805 | 61109039 |
| AMPL7154505630 | ACACATTTTACTTCGAAGAAGAGCAGA | TGACTGTAAAGCTAAACACAGCAGT | chr4 | 126240880 | 126241104 |
| AMPL7154200711 | TTAATCCAAACTCACCCTAATTTCTTCACA | CCTGAGTTAAACATGTGCCTCCTT | chr16 | 3788545 | 3788704 |
| AMPL7154505636 | ACAATATCAGAATCAGCAGCCAATCT | CCTGAATCCACTGCTTGAATTACAAG | chr4 | 126241218 | 126241438 |
| AMPL7154200764 | ACCTCAAACTCAAGAGCTTTGCA | CCTGCTCCTTCTGGACTTCCTA | chr16 | 3790309 | 3790573 |
| AMPL7154505642 | CAACTGATTCCGATTCAGGTGACA | TAGGAGGATTGTCATTAAAGTCCCTCA | chr4 | 126241582 | 126241821 |
| AMPL7154200765 | GAGTAGAAGAGGGATGGCAAAAACT | ATCCGTGCACTCCTGTTCTG | chr13 | 48936746 | 48937020 |
| AMPL7154505646 | GCAACCACTTTACCATAGATGAAGTCAAA | TCTGGGTCAGCAGCCATAATTG | chr4 | 126241963 | 126242233 |
| AMPL7154236549 | CCAAGAGATCCATTCATGACTTGC | CTGAGCCAGGGAGATTCTTCAG | chr16 | 3900455 | 3900729 |
| AMPL7154505651 | GGCCTGAAAGGAGGAAATCGAC | AGAATGGCTGCGGTCTGAATTA | chr4 | 126242386 | 126242653 |
| AMPL7154250781 | TGAGCCCTGGTGACATTTGTTT | CTGGAGGATGAGAAGAAGCAGATG | chr7 | 2983781 | 2984055 |
| AMPL7154512776 | ACGAAGGGCATCCTACGACTAT | AGTAGTCTCCTTGGTAAGTTTCCCA | chr1 | 16254592 | 16254740 |
| AMPL7154280485 | GTAGACCTCCGAGGTGTTGTTG | CCACGTTTCATAGCCAGGAAAC | chr17 | 62007465 | 62007716 |
| AMPL7154524754 | CGGGAAGCTACTTCCTTTCCTT | CGCGGTCTATTTTCTCGTTCAC | chr4 | 134071268 | 134071539 |
| AMPL7153070242 | GACAGAAGATCAGTACCAAGGCAA | CCCTGAACTGTGCTCTTGGATG | chrX | 70460998 | 70461270 |
| AMPL7153193018 | ATAATCCCACTTCTTACTCTCTCATCTTTATCA | CAAACATACTTACCATTGAATGGATTGATTCC | chr2 | 61127959 | 61128231 |
| AMPL7153070258 | GGCCCTTCAGAATGAGTAGCAT | GTCACCTCTCTGGTATTCTGTGATC | chrX | 70460662 | 70460892 |
| AMPL7153193043 | GTGACCTCAATGTGGTGAGACT | GGAGGATGAGATTAGAGAAGATTTCCTTTC | chr2 | 61144045 | 61144319 |
| AMPL7153071653 | TCGGCAGAAAACGCTAGAATGA | GCTTCATTCTACTGGTCAGCAGAA | chrX | 100609495 | 100609768 |
| AMPL7153193488 | AGAATTCCTTACATTTTGTTGCTGTTCTG | CATACGGAAACACAGGTCTTATGACT | chr3 | 89468315 | 89468577 |
| AMPL7153075214 | CCAGGCATTGAAGTCTCATGGA | GTCCAGATGAAGCTCCCAGAATG | chr17 | 7579266 | 7579511 |
| AMPL7153193573 | AAATCTCCTGCTTATACACTATCGAGGA | AGAATTATTGTCTGTCTGCAGGATGATG | chr3 | 89528486 | 89528732 |
| AMPL7153079789 | ATCAATATCAGACTACAAAGTGGACTGAA | ACTAAAAGCATGTAATCACATTGGAGGT | chr7 | 124537110 | 124537333 |
| AMPL7153193581 | GAGCATGGTAACTTCTCCAGCAA | CGGTGAGAACGTGCTTTGAAGA | chr3 | 89156801 | 89157057 |
| AMPL7153079830 | TGACAAGTCAGGTCAAAATACTGCA | TCTGTTTTCTACTTTGCCCTACTTTCTTAG | chr7 | 124503450 | 124503724 |
| AMPL7153193610 | GATGCTTCGAGGGATAGCATCTG | CACGTTTTGGGTCATGATTTCTACTTATTT | chr3 | 89480338 | 89480602 |
| AMPL7153079840 | CATCACCTTCAGAGATCTTGCCA | GATCATAAAACTACTCTACTCTCTTATGGCAG | chr7 | 124499005 | 124499198 |
| AMPL7153193954 | AGTATCTCAGCTCCTTCGAGTGT | AAAGGACCACCAGGTTGATCAC | chr4 | 126237626 | 126237900 |
| AMPL7153079858 | GTACCTCCATGAAGATGAAACTCTAAACTT | CACTCGTATAGCATACTTTGCAGATACTTT | chr7 | 124493089 | 124493341 |
| AMPL7153198782 | AGGCTCACTTTCCCAACTTTCTG | ACCTAGGAAATGCTTGCCAGTT | chr19 | 13419140 | 13419414 |
| AMPL7153079904 | GAAAGCTGTCGTCAGGTTCTGA | TGGATTTTGTGGGTAGAGCTAAATTTACT | chr7 | 124491929 | 124492083 |
| AMPL7153199082 | TACCGTCATTCTGCGGATTCG | GGGAGCATCGACGTCATC | chr19 | 13409320 | 13409589 |
| AMPL7153079961 | GAAAGAAATGGATTAGCTGGTAAGTGTAGA | TTTCTCTCAGCAACGCTCCATT | chr7 | 124486909 | 124487135 |
| AMPL7153199307 | TCTGTCTAGTGAATGAAAACAAAGCTTCA | TTCCCTCACCTGTCAGGTTCTA | chr19 | 13446520 | 13446794 |
| AMPL7153080130 | ATTTTTAAGGTGCCTTAAGTCCTTTTAGGA | GGAATAGCAGACTCATATGGTTTGATCT | chr7 | 124467155 | 124467403 |
| AMPL7153221831 | GGCACTACCTAGACACTTTCTTGC | ATTGCATCCTTTCCAGGAGTTAATGTATTA | chr12 | 23699192 | 23699465 |
| AMPL7153085590 | GGGAAGAAGTAAGAATTTGATGCAAAAGT | GTGCAGTCATAAACCAAATGAATATGTCTT | chr2 | 198264943 | 198265209 |
| AMPL7153221837 | AGCCCTAGCTAAAGGCTTCTCT | GGGAAGAGACAGAGAGAAAATCAATTGG | chr12 | 24102376 | 24102650 |
| AMPL7154368967 | TGCCAATTTAGGAAGTAGGACATAGTAACT | GGGTAGAAGCTGAGATAGTATCATCAGTAA | chr11 | 108121250 | 108121524 |
| AMPL7154524760 | CTAGTGCGAGTACTGGATGCTAA | GGCTTCGGTAACGATGGTGTAG | chr4 | 134072328 | 134072552 |
| AMPL7154368970 | CAGAAACACTCCCAGCTTCTCAA | GGAAAGTCAAGAGGTAAGATGACATAGTTT | chr11 | 108196080 | 108196315 |
| AMPL7154524762 | AAGTGTCGGATGTGAACGACAA | CCTTCAGCTGCTCATAGTCGAAG | chr4 | 134072650 | 134072907 |
| AMPL7154368972 | GTGTCCTTGGCTGCTACTGTT | GTCTTCCAAACAAATGTAATAATTTTCACAGGA | chr11 | 108126988 | 108127151 |
| AMPL7154524764 | GCTGGTAACGCCACTGTCAA | AGTCCATGCGAAAGAGGTTCATTT | chr4 | 134072958 | 134073192 |
| AMPL7154368977 | TGTTATAAGGTTTTGATTCCACATCTGGT | TTGGTAGCAGTCTCTCTTTGCTG | chr11 | 108154958 | 108155157 |
| AMPL7154524766 | CGGCCTTATGAGCTGGTGAT | ATGGCCAGCAGGAAGATGAAGGA | chr4 | 134073249 | 134073493 |
| AMPL7154368979 | GCTTTTAAAGGAGCTTCCTGGAGAA | GTTTTTCACTACATGAAGGACATGGTTTAA | chr11 | 108141799 | 108142056 |
| AMPL7154524768 | AAGAAGAAACTCAGCAAGTCAGACAT | GCTTAAGAAACATCAGGTCGGTCTT | chr4 | 134073630 | 134073810 |
| AMPL7154368982 | GAAGTTGAGAAATTTAAGCGCCTGATT | ATGCAAGGCATAATGATATATAGGAAGCAA | chr11 | 108098506 | 108098742 |
| AMPL7154564640 | GTCTGACCGGGACAGAGACCAT | GGATCAAAAGTTCGTTCTTTATCTGTCTT | chr1 | 16254865 | 16255131 |
| AMPL7154368985 | CGGCCTAAAGTTGTAGTTCTTAACCA | CTAAGCGAAATTCTGCTTTAAATGACTGTA | chr11 | 108205552 | 108205762 |
| AMPL7154564642 | CCTGAAAAGCCCAGGAGTTGTA | TCTGAGAGACCTGAAGTTTCTGGT | chr1 | 16255277 | 16255499 |
| AMPL7154368987 | GTGAGCAAGCAGCTGAAACAAA | AGGAAGAACAGGATAGAAAGACTGCTTATA | chr11 | 108160360 | 108160582 |
| AMPL7154564644 | GTAAGTGCTGTGGATCTGGAGAAG | CAACAGTATTAAGTCTGTCCAGTTTGATTT | chr1 | 16255652 | 16255916 |
| AMPL7154368989 | TGTTCTTGAATGGTGCACAGGAA | CAGCTGTCAGCTTTAATAAGCCATTAAAT | chr11 | 108213975 | 108214164 |
| AMPL7154564646 | GCAGGTGAATCTGTGGAAAATCAAG | TCCTACGACTCTGTTCCATTTGTTTTC | chr1 | 16256009 | 16256270 |
| AMPL7154368991 | GACTCTGCCATATTCTTTCCGTCT | TTGTTTAGAATGAGGAGAGAGGCAAAAA | chr11 | 108188116 | 108188302 |
| AMPL7154564648 | AGGATGGATCATGTCGATTTTGATATCTG | TGTTCTCTCCTGCTAGAATCATAAGGATT | chr1 | 16256420 | 16256691 |
| AMPL7154368993 | AGAAGTGGGTCCTATAGATTTCTCTACC | TGTGAAGTATCATTCTCCATGAATGTCAT | chr11 | 108170463 | 108170677 |
| AMPL7154564650 | GAAAAAGGTCTGTACGAGATCTGGA | GGATTTAGCTCTTTCAAGCAAAGCT | chr1 | 16256832 | 16257094 |
| AMPL7154368995 | ATTTCTTCAGACAACTTTTGACAAGATGG | CCTTCCTAACAGTTTACCAAAGTTGAATCA | chr11 | 108124595 | 108124831 |
| AMPL7154564652 | GAAGAAAATTAGGACTGATTCAGAAGGGA | CCATAAATCTGCTATGGAACAGAACTACT | chr1 | 16257226 | 16257500 |
| AMPL7153085595 | AGAAACCATAGAAAAAGGCAGAATCCTAG | CTCTAGGTGGCAGTTCTGTCAC | chr2 | 198299605 | 198299879 |
| AMPL7153221857 | TTGCTTTAACACCATAATAGCTGTTTTCG | GTGAATGCATGAGTTTAATTTGCAGGT | chr12 | 23793672 | 23793905 |
| AMPL7153085645 | GTGTATCACCTCGTCCAGGAGTA | TTCTGTGTGGGTGTGTGAAATAAAATTTTT | chr2 | 198274578 | 198274774 |
| AMPL7153221880 | GCTTGAAAGTCATTTTTATTTTGGAGGTCA | ATCAGTGTCAAATCAAACTTTGAGAAACAG | chr12 | 23887509 | 23887783 |
| AMPL7153085721 | ATTTTGCTAATTGAATACAAAGTGGCCAA | GACATTGCTAAGTAAAAGGAAAGTGAACAA | chr2 | 198263108 | 198263381 |
| AMPL7153233216 | GCCATATTTTACATAGAGGTGACACTTAGT | AAATCGATCACAAAGCAGTTCACATG | chr18 | 3173886 | 3174045 |
| AMPL7153086135 | GGGTTAGGCCAAAGTTCTCAGT | GTTAGGGACTGGAGGTCAGTGA | chr12 | 49418534 | 49418794 |
| AMPL7153234363 | TCTTCTCCCGCTGGTAGTGA | CTCAAAAAGTGTTGTTTTCCTTCAGGT | chr18 | 3215122 | 3215274 |
| AMPL7153086139 | CTGGGTCTGAGTTTCCTCCTCTTA | TGGTAAAGCCTGTGGGTGAGTA | chr12 | 49439588 | 49439862 |
| AMPL7153234368 | CTTCATTCCTTTTAAAAGGCTCATTCATCT | CCCTGGATGGAAGCAAATGCTA | chr18 | 3079113 | 3079387 |
| AMPL7153086164 | AAAGAAGGGCTCTTAGATTAGATGTGC | TCCTTATCTCCCTTGCTTGGAGA | chr12 | 49444591 | 49444798 |
| AMPL7153234484 | GGGTAAGATAGAGGGTAGAGAGCTAA | CTGCATTAAGAACTATTCAACTTCACAAGA | chr18 | 3085979 | 3086225 |
| AMPL7153086177 | GGGTGGCTTCTCAAGCTCA | CCTGAGGAGCCATGCCTATG | chr12 | 49444889 | 49445146 |
| AMPL7153234517 | GAATAACTGAGAATTCAAAGCAACATTCGA | AGTAGTATACACAAATGCCCAGATTCAG | chr18 | 3100260 | 3100515 |
| AMPL7153086573 | CGTCCGCAGAGGTAGACAAG | TAGTGGGTGGCCTCTCTTGA | chr12 | 49435132 | 49435403 |
| AMPL7153243386 | TTCCTTCCAAAATCAATATGCCAGATACA | TCATTTACTGCATTAAGCATACTTAGGCAT | chr15 | 75706488 | 75706761 |
| AMPL7153086600 | CCCAGTATACCCATGGTCCTTCT | CCTGAGCCAGCAATCCTCTTTT | chr12 | 49447996 | 49448264 |
| AMPL7153247999 | TCTCCACACATCAGCACAACTAC | GGGCCTTTTCATTGTTTTCCAACTC | chr8 | 128752789 | 128753055 |
| AMPL7153086654 | CTGCAATGAGAGAGGCTGCTAA | CCCTACTTGTCCCACGTCTCTT | chr12 | 49428260 | 49428527 |
| AMPL7153254010 | GGATTGCCATACATCCTCCCAT | GGCACTCATAGTGTCTGACACT | chr7 | 65554400 | 65554643 |
| AMPL7153086706 | CCCTCACCTTCCCAAGAACTTC | CAAACGCTGCAGGTTTTGAACT | chr12 | 49424329 | 49424594 |
| AMPL7153254070 | CTCCTCAGGGAAGCAACACAT | CCTAGGTCTCCTGTTCAGGCTT | chr7 | 65548009 | 65548228 |
| AMPL7153086749 | GCCCAACTCCTCATCCTTCTC | GGGCTCTAGGCAGTTTGTCTG | chr12 | 49435942 | 49436186 |
| AMPL7153254128 | CTCCTGGGACTGTGCAAAAGAT | GAAGCTGGGCTGAAGGACTAAA | chr7 | 65552577 | 65552839 |
| AMPL7154368996 | CTGAGAACCCTGAAACTTTGGATG | CACAGTGACCTAAGGAAGCTTCTAATAAAA | chr11 | 108151732 | 108151942 |
| AMPL7154564654 | CCAAGTGTCACAGTCGTAACTCT | TCACCTGATGAACCTTCCTCAAC | chr1 | 16257653 | 16257912 |
| AMPL7154368998 | TTTGATGCTTAGGAAGGTGTGTGA | AGCGTTTACGATCCTCTTTCAGTG | chr11 | 108200744 | 108201017 |
| AMPL7154564656 | CGTAGATCCAGAGCCTGACAGT | TTTGGCTTTTTATCCTTTGCAGCAA | chr1 | 16257979 | 16258191 |
| AMPL7154369000 | GCAGTTTTAAAATCCTTTTTCTGTATGGGA | TAGCCACTAAAACTCTATGAACATCTTGTG | chr11 | 108114564 | 108114748 |
| AMPL7154564658 | GGAAAAACTCAAGCGGTCCAAT | GAAGCTCTCGCTCCAATCTCTT | chr1 | 16258282 | 16258550 |
| AMPL7154369002 | GGATATAGACGTTTAGAAGACTTTATGGCA | GTATGGGTATGGTATGTGTGTTGCT | chr11 | 108153464 | 108153706 |
| AMPL7154564659 | GTCGCCAAGGTCCCAGAAAACT | CTTTTCTGTCACGTTTCTGTTCACTC | chr1 | 16258681 | 16258874 |
| AMPL7154369003 | CCTCATAGGCCTCTGCCTTTTT | CTCTGGTTTAGTCAGAAATTCATCTCTGT | chr11 | 108202447 | 108202704 |
| AMPL7154564661 | AAAAACTCCAAATCAAAGAGAGGAAGATCT | CAGAGGCAGACACGTCTTCTTT | chr1 | 16258946 | 16259204 |
| AMPL7154369005 | GGACTCTTCAGCCATGTTATCTTATAATGT | CTGAGCTACCTTGGCAACTTCT | chr11 | 108180709 | 108180962 |
| AMPL7154564662 | CTGCACACCAAGCAAGTGAAACAGAG | AGTTTCCAGGATGCCAGATACAG | chr1 | 16259352 | 16259560 |
| AMPL7154369007 | TGGGTTGGACAAGTTTGCAATAGT | CTTCATTTTTCCATTTCTTAGCTCATCACT | chr11 | 108199597 | 108199813 |
| AMPL7154564664 | CACTCTTGTTCGGAAAGACAAAGG | CTCTGTGGAGGGAATCTTGCTTAG | chr1 | 16259704 | 16259962 |
| AMPL7154369011 | AAAACCATCTTCATGTTATTGTTGGTACAC | CAAGTGCTCTGTAATTGATTACAAAGGTT | chr11 | 108163450 | 108163698 |
| AMPL7154564665 | GGTGCATTCCATCATTGAAAGTGAC | AACATCAGAGGCCTTTGTGTCT | chr1 | 16260082 | 16260316 |
| AMPL7154369012 | AGTAGAACCTAATTTTTCTGCTGCCTAAA | CCAGGCCTTCAAAAGGGTTTTC | chr11 | 108173354 | 108173628 |
| AMPL7154564667 | CCAGTTGACTCTAAAAAGCCTTTAGAAGAA | GAGCTGGTTTTGCCAAGGTTAT | chr1 | 16260458 | 16260665 |
| AMPL7154369014 | ACGATTAAGCTTTTAGATCCTTTTCCTGA | GCCTGGCCTACGTATATATTTTTAATCACA | chr11 | 108164100 | 108164338 |
| AMPL7154564669 | AGGGCCTGTGAATGTTCTTACG | GGAACCGACTGTTTTCATTAGCAC | chr1 | 16260817 | 16261091 |
| AMPL7154369015 | CCAGGTGTCTTCTAACGCTGAT | TGACAGATATCTGCCATCAATTCAATCA | chr11 | 108117580 | 108117850 |
| AMPL7154564671 | ATCAGCGCCAAGATCAGCCAGAT | GACGAAATCACAGGAGAGGCATTAT | chr1 | 16261223 | 16261437 |
| AMPL7154382976 | CCCTCAACGTTAGCTTCACCAA | GCAGCTCGAATTTCTTCCAGATATCC | chr8 | 128750513 | 128750674 |
| AMPL7154564673 | CATTGTCACCACAAACAAGAAGCT | TCGAGGAGAATGAGCATCTAGCTT | chr1 | 16261591 | 16261816 |
| AMPL7154564675 | CTCCACCAACGCCACAGTCAT | GGCCCTGACCTCTATTTGCTG | chr1 | 16261885 | 16262134 |
| AMPL7155531295 | GAAGCCATTACTCACCAACGACATA | CATGACTGACAGCTTCTTCTTGTG | chr3 | 183210267 | 183210535 |
| AMPL7154564677 | CCCTATACTGTGCCACGGG | GGTAACTGTGAGGATACGGGC | chr1 | 16262297 | 16262554 |
| AMPL7155531297 | TTCAAGATTGGGCTCTCACACTAAC | GAAAACAGGAGACTTGGTCGAGAT | chr3 | 183273069 | 183273343 |
| AMPL7154564678 | GAGCTGTTTCAAGAGTACCGGTA | CACACATCTTGGCTTAAAAGCCTG | chr1 | 16262636 | 16262841 |
| AMPL7155576140 | CTCCAACCTGAACCTGAACGAA | CTCACTGGAAATTTCTGAAGACTGTAGTT | | |  |
| AMPL7154598854 | GGGTGGAGAGATTCTTTCACACT | CGAAGACAACGTGGTGAGAAAAT | chr19 | 13616574 | 13616793 |
| AMPL7155576142 | GAAACACTTCTATTGGATCCAATAGTTGC | GGAGAAGTGATGCCATTTATTGATGAAATT | chr2 | 61719062 | 61719244 |
| AMPL7154630437 | ATCAACATCAATGCCTTACTGATTACGA | TTGTAAAGACGTCTGTGTCTTCTGTT | chr11 | 102206572 | 102206845 |
| AMPL7155662979 | TTCAAGATGTGGTGCAGAGTGT | CTTGTGGACTCACCATTTTCTGG | chr3 | 183226088 | 183226348 |
| AMPL7154630492 | TGACTCGAACATGTTGGGAAATGT | GTACTCATGCTGAACAAGTTCAAAGTAATC | chr6 | 138199367 | 138199641 |
| AMPL7155662980 | GTCAAAGCCTCCGATCACATAGA | CACATCTGATGGTTTCTTAAATGCTTTGTA | chr3 | 183211927 | 183212109 |
| AMPL7154630516 | TAAGATCTTTTGCCTACAGATCAGGGTA | GCATGGTTTTAAAATGATGAATGATCCCAT | chr6 | 138192221 | 138192494 |
| AMPL7155867319 | CTCATGCTGTCTGTGGATCTCT | GTCAGCTCCTTCACGAGGATTT | chr6 | 401225 | 401478 |
| AMPL7154632699 | CGCACATCCAGATTGGAGACAT | GGAGGGAGCTAGTTAGATGAGGA | chr12 | 49421038 | 49421162 |
| AMPL7155867322 | GCCCAGAGCCTTCTAGAATGAG | GGATTTCTTTCACGCTGTACTCTCT | chr6 | 106552539 | 106552795 |
| AMPL7154632925 | GGAGACAGGCGAGATGCTTC | CCTGAGGAGTCTCCTCTGTCT | chr12 | 49445745 | 49445974 |
| AMPL7155867327 | CCTCCCGTTGGCAACTCTTAAT | GCAGTTTCAGGTGCACAAACTG | chr6 | 106554712 | 106554912 |
| AMPL7154632928 | AAAAAGAGAAGAGGAATAAGCCCATTCT | ATCACCTCTCCTGTCGAAAAGC | chr12 | 49433410 | 49433641 |
| AMPL7155867328 | CCAGTGCCACAAGAACTACATCC | TTCAGGCCAGTTTCTTCTTTCTCTTT | chr6 | 106554953 | 106555201 |
| AMPL7154632929 | AGTTCCCTGTGGCTACTGTGTA | GTTTCACTTTCCCTCAGGCAGTA | chr12 | 49433794 | 49433949 |
| AMPL7155867343 | CGCCCTGATTTCTGCTGATTCA | GGAGAGTGTGCTGGATTCACATAG | chr6 | 106547001 | 106547266 |
| AMPL7154632930 | CCAGCCTTAAAGGCAACTTCAG | CTTTTTGCCCATCACCCGTTA | chr12 | 49434108 | 49434252 |
| AMPL7156016224 | CCCTGTAAGAGTCCAAATTTGGCT | GCATCTCATCCACTCTGTGCAA | chr6 | 44227591 | 44227865 |
| AMPL7161082679 | ACCGTCGCCGTGTACTC | GCGCCTTCTTCGTCCAC | chr4 | 48492840 | 48493008 |
| AMPL7161082812 | CGGCCTCGTTGTCCTTC | CCCAGCGAGCACTGTAC | chr4 | 48492481 | 48492676 |
| AMPL7161362724 | TTATGAGAAGATGGTTCAAGTTGAGAGTG | CCCTGGCTGCTACACACTTAC | chr7 | 65556833 | 65557094 |
| AMPL7161661114 | CTCCAGCATTCCAAAATGTACACATC | TCAGTTTAGTTACCAGCATGCCAT | chr8 | 117670828 | 117671101 |
| AMPL7161661116 | TTGCAAAAAGTTCTAAAACATGGGCTT | TTTAGCACTTACTTTTGACCATCAGACTAA | chr8 | 117668046 | 117668297 |
| AMPL7161661119 | AAAAGTTATGGTGAGACCTGTGTCAA | CTAGGATGGTTGTTAAGCTCCCTT | chr8 | 117738180 | 117738454 |
| AMPL7161661121 | GCATGCTTTTCTGTTTGTAAATTTCTCAC | TTTCCTCCTCAGATGGAGAAAAATAACC | chr8 | 117658621 | 117658895 |
| AMPL7162084780 | CAGCCCACAGGCATTTAGTCTA | GTCTTCAAAACGAGAACAGTAATAGTTCCT | chr13 | 50656461 | 50656725 |
| AMPL7162084783 | CACTACCTCACGGTCGTAGTTG | CCAAGAGTGCCCAGAAGCTAAAG | chr12 | 51584102 | 51584352 |
| AMPL7162084785 | AGCAGGCCCTAATCCTGTCTA | TGGTTCCCATGTTACACACTCATC | chr12 | 51585342 | 51585594 |
| AMPL7162084787 | CCGGTGCCAGATAGCAGA | CCTTATTTCCTCCCAGGTTATTGGAAC | chr12 | 51589699 | 51589972 |
| AMPL7162084789 | CAGAGGAGATAGGAACAACAGCTT | CCCTCACTACAGCTCCAGTCAT | chr12 | 51590453 | 51590667 |
| AMPL7154632932 | GCTGAGCATATGGGTCAGTGTA | CAAACCTGGGCTTTGTTGACTC | chr12 | 49434412 | 49434648 |
| AMPL7156055161 | TTTTTGAGGCAGCACAATATGGC | CAGAATGGACTTCAGTTAAGTTTTTGATGT | chr13 | 50623262 | 50623533 |
| AMPL7154632933 | CACAGCCAAGTTATCCAGCGA | AAGTGCCTTCGCAGGACCC | chr12 | 49434782 | 49435044 |
| AMPL7156079058 | GGCCTCACCATAGCAGTGATC | CATCCGCGTGGTCATGATCA | chr17 | 3844257 | 3844511 |
| AMPL7154632945 | CTCACCAATAGAACAGCGATAGCA | AGAGCTGAGCTCTTTTGCTGTC | chr12 | 49420047 | 49420317 |
| AMPL7156079184 | GCCCACAGATTCTCTCCTCCAT | GGCTGTCATCACTACATGCCT | chr17 | 3850572 | 3850844 |
| AMPL7154632947 | GCACAGGGAGCACTTGGTTAG | CTGACAAGGTACCGCGAGACAT | chr12 | 49420464 | 49420684 |
| AMPL7156079187 | GAAGTGGCCGATGTTGATGAC | GCTGAGCAGCTTTTTCCTTGTC | chr17 | 3850943 | 3851185 |
| AMPL7154632949 | GCCAAGCTGCTCCATAAACTCT | TACTGAGCCCTTGGTTGAACTTC | chr12 | 49420698 | 49420962 |
| AMPL7156079218 | GAACTCCAACAACTCGAGCTGTA | AGGGAATTCAGGGAATTATTAGGCAG | chr17 | 3838429 | 3838659 |
| AMPL7154632971 | TCTCTAGCCTCAGTGCCCATTTA | CATCCATGGCTATGGTGTCCAA | chr12 | 49430832 | 49431105 |
| AMPL7156079223 | ACCTGCCACTTCTACAATGTCC | CCCAGAGGAGTCACTCCAA | chr17 | 3854543 | 3854760 |
| AMPL7154632973 | GTCCACCAGGCAAGGTCAAAGC | TAAAGCCAGGACAGAGCATGATG | chr12 | 49431463 | 49431678 |
| AMPL7156079230 | CACACCCTCCACTTGGTGATC | CCAGATATCTCTGCCTGTCATCCT | chr17 | 3831811 | 3832024 |
| AMPL7154632976 | GGTAGCAATGGTGAATTGGCAA | GGACAAGAAGGATATCTTCAATGAGCA | chr12 | 49431725 | 49431959 |
| AMPL7156079237 | CAAAACGAAACAAAACAAAAACTTCAGTGT | CACGGAGACAGCTCTGACTTG | chr17 | 3846581 | 3846784 |
| AMPL7154632978 | CAGGTGAGCAAGCTCTTCATCAT | CCTGGCGGTATCAGGTCTTC | chr12 | 49432103 | 49432370 |
| AMPL7156367936 | ATTTAGGTGCTCCAAATACTGCAGAA | GCTTGTGAAAAGATGCCTTTTGCT | chr2 | 61145320 | 61145589 |
| AMPL7154632979 | AGGTCCCAGTCCTTTCTGTACA | TTACAGCAGCAACAGCAACAACTGT | chr12 | 49432469 | 49432711 |
| AMPL7156367938 | GTATTCTCTGGAAAGTTTCATCTAAACTGC | TAGCCGTCTCTGCAGTCTTTTC | chr2 | 61121368 | 61121641 |
| AMPL7154632982 | AGCATCGCACAATAGTGAGTCAT | CACCCACTCTCATCAAATCCGA | chr12 | 49443546 | 49443784 |
| AMPL7156367940 | CGTTACAGAATTTCATTTTGGATTCGTGT | GGCATTTCTCTCACAACTGCATC | chr2 | 61148707 | 61148940 |
| AMPL7154632984 | GCCTTTAAGTTCACTAGCCAAACTG | CACGAGATGGAGACTGAGAAAGT | chr12 | 49443921 | 49444160 |
| AMPL7156367973 | CGGTGGATGTCTTGGTTCTTTATATTCTC | CTTTGTTTGCCTCTAGCCCTTCT | chr17 | 43345071 | 43345232 |
| AMPL7162084853 | TATGTGTGGTGTGTACAGGGAACTA | GCCTGGCCTCTTTTTGGAAGTA | chr4 | 48494846 | 48495119 |
| AMPL7162084855 | GCCTCGCTCCGTCAGTTT | CCGGTTCACTGACCTAGCTTG | chr7 | 56131849 | 56132084 |
| AMPL7162084857 | CCAGCTGAACCAGCTGAACTAT | GTAACCCATGTCCCACAGGATT | chr7 | 56140658 | 56140879 |
| AMPL7162084859 | CTAGCCTGCCTCCGACTTAATG | GGGAGAAGAATAAAGGAAAATCGCCTT | chr7 | 56142218 | 56142469 |
| AMPL7162084861 | ACAGCAAGACGACCTTGGTTTT | TGCCTCCTCTCTCAGACATGAT | chr7 | 56145703 | 56145953 |
| AMPL7162084863 | GAGTGGACAGCATCACCGTA | GGACCGGGATTCAGACATCAG | chr7 | 56146086 | 56146267 |
| AMPL7162873023 | ACTTCTTTGTAAGGCACTGCAGAT | ACACTTCTCTACAGAGAGTTTGATGAAAAG | chr13 | 50678551 | 50678771 |
| AMPL7162873026 | CCCAGGAAAGAAGCAGAGAATCTC | CAAGACTCACATCCCTTTCCTAGTC | chr17 | 3827259 | 3827461 |
| AMPL7154632989 | GAGCCCTCATCTCTTCTGTCTG | GCATCTGCTCTTGCAGAAGCTA | chr12 | 49424860 | 49425111 |
| AMPL7156479913 | CAAGGCCCTGGTTAGGGATAG | GCTACATGTGCCGTTTCATGTG | chr19 | 16437569 | 16437843 |
| AMPL7154632991 | GTTCCTGCTTGATGCTGAGTTG | CTTGCAGATACCTTGTTTAGCAAGG | chr12 | 49425233 | 49425395 |
| AMPL7156479917 | TTCGGTGGCCCTGGTTTCG | CGTAGCTGCAGGTGTGAGTG | chr19 | 16436540 | 16436780 |
| AMPL7154632993 | GCTGCTGCTGAGGACTTAAGTG | GTCCATGACCCAGAACCTTCTG | chr12 | 49425830 | 49426050 |
| AMPL7156479923 | GTGTCTGAGCTGCTGCGA | GGACCTCGCATGCACGA | chr19 | 16436225 | 16436439 |
| AMPL7154632995 | CCTCAGTGGCCTCTGAAGAAAC | GCAGATGGGCCTTTTAAACCAG | chr12 | 49426370 | 49426569 |
| AMPL7156479925 | CATGGCGCTGAGTGAACC | TCCTCTCCTGAGTCTGCAGTC | chr19 | 16435734 | 16435926 |
| AMPL7154632999 | GCCTTTCCTGTAAAAGCCTTGAATC | CACCAAGCTCCCTGGTCAG | chr12 | 49427330 | 49427604 |
| AMPL7156619374 | GGGACCCAATCTGCTGCTT | TTCCAGCAGCCTCAAGGACC | chr16 | 3778126 | 3778357 |
| AMPL7154633001 | GCTTGTTCCGATATTCTGCCATGA | GATCCAGAAACAACTGGATCAGGT | chr12 | 49427693 | 49427871 |
| AMPL7156619376 | GAGGAGTCTGTCCCAACTACATAGA | AAAAAGTAGTTTGATAGATGTGTTATCCTTTCA | chr16 | 3828586 | 3828852 |
| AMPL7154633006 | CCCATCCTCAGGTCCTGTAAATG | GGGACTCTTGCTCATAGGAGATGA | chr12 | 49436956 | 49437228 |
| AMPL7156643214 | AAAGGATAGGTAACCATAAGGAAGCCTA | AAGTCTTCCCTTGCCCTGTTATAAC | chr16 | 81954702 | 81954975 |
| AMPL7154696861 | TGTGGATTCATACTCGCCATGTT | ATGGCAGCTTTCATCAAACAGC | chr16 | 3778485 | 3778759 |
| AMPL7156643286 | AAGTGTATGTTACTTGTGATGAAGCCT | GGTCTACATGACTTTATGAAGCCCAT | chrX | 41201918 | 41202185 |
| AMPL7154696865 | TTCCCTGTGGACACCGTGG | CATGAACACCCGCAACGTG | chr16 | 3779238 | 3779435 |
| AMPL7156643445 | GCTGGTTAAATATAGCACCTCTTATCTTCT | CTCAGGTGTTTGAGACCGTGAT | chr3 | 183225811 | 183226058 |
| AMPL7154696867 | CGTTGGTCTTGCGTTTGCA | CGACCTCTGCATCAACTGCTATAA | chr16 | 3779608 | 3779870 |
| AMPL7156693029 | CGTCTGAGGTGCGACTTTACAC | ACAGTCCAGGAGAGGTTTAAAACAATG | chr18 | 50918193 | 50918338 |
| AMPL7154696905 | AACGAGGTTATGTAAATGATGCTGGT | GCCAATGCCATCGTAATCACTC | chrX | 41201568 | 41201787 |
| AMPL7156698184 | GAAAGTCACCTAAGGGCCTCAT | GAAAACCCTGATGTTTCAGTGTCTAGTTA | chr6 | 138197087 | 138197360 |
| AMPL7154703197 | GCCTCATACAGGGAGAGACAGA | GGTCTTGAGTGAATATTCAAACCTGTTC | chrX | 70469955 | 70470091 |
| AMPL7156698224 | TTACTGTCTCCTTAGAATCTGAGTGCT | AAAAAGGAAAAAGAAAACCACTCATCTTGA | chr6 | 407366 | 407634 |
| AMPL7154725932 | AGGTGGTCTCCTCCTCTTCAAT | GATGCAGGAGATCAGACCCTAGA | chrX | 70472616 | 70472790 |
| AMPL7156698226 | CTCTCCTGCACTCCTTTAGCA | GCCCATGGCCACATTAGCA | chr6 | 397089 | 397309 |
| AMPL7154725937 | CGCTTGAAGTCCTCACAGCAAT | ACTACTGTCACAGCCTCTTCAGT | chrX | 70468113 | 70468337 |
| AMPL7156718208 | TGGGTTGGTGTTTTAAGACACTCA | GAATCGCAGGAGTCTCACACAA | chr8 | 42186492 | 42186762 |
| AMPL7154725940 | TCCCGGTCTTGGTGTAGATGTAA | CACTGTCTGCTGTGAAGGTCTAG | chrX | 70469391 | 70469634 |
| AMPL7156728015 | TCCTCTAATGCTGCATAAGCTTTTTAAAAA | CTCCAGTATTAACTGTGCTGCTGAA | chr13 | 50622878 | 50623144 |
| AMPL7154725942 | GGAGAGTTGCATGTGTTCCTCA | TGAGAATTGTGTGGGAAATGAGCA | chrX | 70462126 | 70462375 |
| AMPL7156798379 | ACTGGTTCTGTGGCAGAAACTG | GGTGGTGCTTGTTGTCTCGTAG | chr16 | 3823820 | 3823953 |
| AMPL7154725949 | GCCTGCTTCACTTTGTAAGAAGTG | GATACACAGGTCTTTGTTACATCATCATCT | chrX | 70468880 | 70469151 |
| AMPL7156799048 | AATGATGGTGCCAATGTACTCGAT | GCTGTCTTGTCATGGATTATATCACCTC | chr12 | 49416418 | 49416690 |
| AMPL7154725951 | GCTGGTTTCTCCATTGGCATATTTTG | GTGCTGACTAGCTTTCACCTTG | chrX | 70464177 | 70464389 |
| AMPL7156803955 | AGGCCACGTCTGACAGGTA | GTGCAGGGCAAGAAGGTCC | chr9 | 139391548 | 139391734 |
| AMPL7154738398 | TTCTGCTGGCTATAGAAACGCA | CAGCCAGCCTTCCATCTCTAAG | chrX | 70467242 | 70467485 |
| AMPL7156807728 | TCGTCAGTCCGGTCTTGTCT | GCAGGCTTCCTAGGTAATTTTCAAAATT | chr7 | 65541001 | 65541213 |
| AMPL7154738400 | CAGGACTCCAGGGTCTTTTTCC | TCTTCTACTCCTAATCTGCAGTACATATCC | chrX | 70472887 | 70473145 |
| AMPL7157036677 | GCTCAGGCAATACAAACCCACT | CACTGCTGGTTGTCATCATATTCAC | chr2 | 61149072 | 61149320 |
| AMPL7154738402 | CCACCATCTATGGCATACCACAAAG | GGTTCTTGGCATTTAGTTGGGATTC | chrX | 70465500 | 70465727 |
| AMPL7157036679 | CTACTGTTTTTGTTTCACAATCAGATGCA | CAAAATGCTGCATCTATATAGGTGGTATCA | chr2 | 61149465 | 61149728 |
| AMPL7154738404 | TGACCCTCCTTACCTTGCAGTA | TCTCTGGACTCTCTTTCCTCTTCTC | chrX | 70467571 | 70467791 |
| AMPL7157036681 | AGCATACAACTTTGGACATAGCGA | TTCCTGGGTTCAAACAATTATTGTGC | chr2 | 61149835 | 61150108 |
| AMPL7154811472 | CGCCTGGTTTATGGCTCGAA | GTTTAAATGACATAAAACAGCCACTGCTT | chr18 | 3112351 | 3112477 |
| AMPL7157070088 | GCACTGCTTTTCCGCTCTTTC | TGACCACCTCACCACCCATAT | chr10 | 64573109 | 64573252 |
| AMPL7154811483 | AGGATTTCTTGTTCTTGTCTCCCTCTA | CTTCCGAGTTCGAGCTGTGAAT | chr18 | 3151571 | 3151792 |
| AMPL7157088756 | GAGTGCTGGACTCCAATGACA | GGCTTGCACGTACACTTGGTA | chr4 | 134072011 | 134072285 |
| AMPL7154811491 | TGTTACTGGGCTGGTCTTGAATC | GTGTACGGATGGGAGAATATTATGAACAAT | chr18 | 3164057 | 3164329 |
| AMPL7157456506 | CTTTTCTACTGATACTACTGATTTGGGACA | TCTTTTGTTTCTTTGCTTTATTGCCGTA | chr2 | 61147004 | 61147208 |
| AMPL7154917282 | TCTCAACGCAGAGGGAGACTTA | GTACAGGGCTCCAGAAATTATGTTGA | chr22 | 22153208 | 22153334 |
| AMPL7157460460 | GCTTCCTAACCAGGACTTGTGA | TTGTTAACCAACGTGCTTCTGTATCT | chr3 | 93646210 | 93646422 |
| AMPL7154920644 | GGTTGACGCTCTCCACACA | TGTGGTCCACCTGACCCTCC | chr18 | 60985410 | 60985627 |
| AMPL7157793929 | GCCTATTTTTACCTTGTTTCCTGATCCA | TGCATTTATACCACCCAACTCAATTTCA | chr18 | 3089523 | 3089647 |
| AMPL7154920650 | TCACCACACCATGCCGTAG | TCGGCGTCTTCCTTCCTCC | chr22 | 22221608 | 22221876 |
| AMPL7157965586 | GCCCAAAAGGAATTTAAAGCCAGA | GATGGCAAAGGGTTTCACTGTC | chr7 | 56136025 | 56136299 |
| AMPL7154920652 | GGATAAGGAAGAAGAAGAGGACTACCA | CTCTACCAGATCCTCAGAGGGTT | chr22 | 22159970 | 22160243 |
| AMPL7158091269 | GCCAAACCATATCATACAGCTTCAAT | GCAATATTCCTGTTTGTGACTTCTTCTCA | chr4 | 87662704 | 87662865 |
| AMPL7154920719 | GCCGCAGGTGGTACTATGAC | GTGGTTGTTGTTGTCGCTGAT | chr16 | 81946195 | 81946457 |
| AMPL7158091272 | CTGCTTCCTCTAGTTCGATGGATAAG | AGGGAAACTTTATGAACACACCTTCTTTTA | chr4 | 87693993 | 87694183 |
| AMPL7154921650 | CTACTGGAGCAGCAGAAGTACAC | GTACGTGAAGAGAAAGGAACAGACA | chr8 | 42166428 | 42166606 |
| AMPL7158091279 | AGATAATTCTGGACGTGAAGATTCTGAAAA | CCATCTGAGTATCTTGCCTCCTTTTT | chr4 | 87622539 | 87622799 |
| AMPL7154993952 | TGTGGGTGGAATATAAAACAGTGTGAG | CAGACGTGATATGATAAGAGTAAGAAGCAT | chr3 | 89391030 | 89391285 |
| AMPL7158729250 | GGAGGATGCGGATATGACTCTG | TCTTCTTGTCAATAAACCAGAGGCTTAC | chr6 | 106536156 | 106536352 |
| AMPL7155096721 | GTCGGAGTTGCTCCTTCATTTG | GCTAGCCTAGACATGCTTTTATACAAAAGA | chr12 | 23716266 | 23716393 |
| AMPL7159430908 | CGGCCAGTTTTTCAGATTTTAACTG | GTATCTTCCTTCTCCAATTGAACCGA | chr2 | 61147631 | 61147767 |
| AMPL7155098458 | AAAAATTGAGATAGACAGGGCTAAGTGT | GCAGTTTAGCTGATGTTGTTGACAC | chr12 | 23998732 | 23998988 |
| AMPL7159565029 | CGGCCATTGCTGGGAAATTAAA | GCTAAAAATACAAGATGAGAAGCCATTCTC | chr4 | 74459028 | 74459242 |
| AMPL7155098466 | AAGAAACGAAAACAGCGTATCTTATGC | CTCTGAGGAATTTCAGCCAGTTTCT | chr12 | 24048535 | 24048808 |
| AMPL7159565031 | TGAATAACACCGGATTGCCTTTCT | GCTTCCACTTCCAATGACTTATGTTAAGTT | chr4 | 74464320 | 74464594 |
| AMPL7155098477 | CTAGTTCTTACTCACTTGTTCTTGTTGC | CTGTATACAACAGAGCCATTTTCATTGAAA | chr12 | 23893786 | 23894055 |
| AMPL7159565032 | TCGTATAAATGCAAGTACAGAACTTATGCA | GTAAGTCAAGAGCATGCACATTGG | chr4 | 74441861 | 74442126 |
| AMPL7155098479 | GACGGGCTTGCTCCTCATAATA | TATCACTTTCTGGAATGCTTAGAAGCTG | chr12 | 23689541 | 23689665 |
| AMPL7159565034 | TGGTTATAGAAGTGTCCATTAATAGAGGCT | GGCAGCTGAATGATATGAGCTTATAATCT | chr4 | 74450901 | 74451121 |
| AMPL7155131903 | GGGAACGAGCATTGAATACAACAC | TGAAAGAATTCAAGGATATTGCAGAGGAAA | chr18 | 50705331 | 50705558 |
| AMPL7159565046 | CAGCATTAGCGTTCCAGCAATG | CGAGTGAACCCAATTTTCCACTTTTAGATA | chr4 | 74481521 | 74481763 |
| AMPL7155131922 | TTCCTGATCAACCAAGCTCTCTTC | CAGGGAACAGTTTTCCAAGAAATACAATAA | chr18 | 50866092 | 50866363 |
| AMPL7159565226 | CTCACGATGTCGTGATGCATG | CGGAGGAGGTTGTACTGCT | chr9 | 139391859 | 139392111 |
| AMPL7155177116 | CCGACAGAAGCAGAACTTTAGGG | CCATAGCCACGGTCCTGAAAC | chr22 | 29196449 | 29196691 |
| AMPL7159581950 | CAAAGCAGGAACAAGAAACAAGTTATACC | CCTCCTCTAGACTGCATTGAAATAATACAT | chr3 | 89448463 | 89448662 |
| AMPL7155245108 | AGGTAATTGGTGGATTTACCTTTCCT | GCATAGTTAAAACCTGTGTTTGGTTTTGTA | chr2 | 198266690 | 198266885 |
| AMPL7159581954 | GCCTTGGTGTCTGTGAGAGTAT | TGCTCAAAGAAAAATAGAGAAGGCTCTTAC | chr3 | 89259427 | 89259700 |
| AMPL7155245110 | CAAGATGGCACAGCCCATAAGA | CTCATGACTGTCCTTTCTTTGTTTACATTT | chr2 | 198267317 | 198267583 |
| AMPL7159597483 | GTGAAACATCAGCTTCTAGAAGATCCA | AGATGACCACAGGAGTTGAGTTTTT | chr13 | 51066691 | 51066965 |
| AMPL7155323966 | GTGGCTTCCAGGTTCCCAT | TGCGGCTGGGTTTGATGA | chr5 | 137802872 | 137803099 |
| AMPL7159597548 | GCAATCAAGTCACAGAAACCTACCA | AGAGTGATGAAGGTTACTGGTAAACATG | chr13 | 50964245 | 50964519 |
| AMPL7155323968 | ACGAACGCCCTTACGCTT | TGGTATGCCTCTTGCGTTCATC | chr5 | 137803139 | 137803384 |
| AMPL7159597550 | GTGCTGTGCTGATATATAAATTCCAAGTT | TGGAAAAATGGAGAAGGAAGAATAACCTTT | chr13 | 50567881 | 50568155 |
| AMPL7155323970 | CCACCTCCTCTCTCTCTTCCTAC | GAGTTGGTGACAGCTGAGGAA | chr5 | 137803442 | 137803700 |
| AMPL7159597573 | ATTTCAACTTTACCAATTGTCTAGTCCGAT | CAACTGTGGAACTGCAGGAGAT | chr13 | 50649616 | 50649862 |
| AMPL7155325628 | CTCTCCGTGCATGTGTTGAC | CGACGGGTCGTAGAAGTTGC | chr12 | 113515064 | 113515269 |
| AMPL7159597586 | CCCAGTTACTTACAGTTTTCTGCTG | TGGTAAAGCACTTTCCCTTCTATATTGATT | chr13 | 50601330 | 50601594 |
| AMPL7155325633 | CGTGGCTCGACCTCTCAT | GCGCGTGCTGTTGACCA | chr12 | 113515382 | 113515605 |
| AMPL7159597593 | AGCCCACAGTGTTGAAAGGG | AGCTTTGCTGAAACTGCACAAAAA | chr13 | 50699403 | 50699675 |
| AMPL7155327710 | ATCCACATTTATTCTCTTGGAATGACACT | GTGTGGAAGAGCTATGATATAAGGTGTAAA | chr4 | 87607863 | 87608004 |
| AMPL7159606161 | AGTGCTACTCAAGTGTGAAGTCATTG | AGGCAACAAAGAGCATTGCAATAC | chr18 | 50432469 | 50432722 |
| AMPL7155327774 | ATCACCTCCTAAGCCTGGAGAT | GCTGCTGGAAATTAAAGAAGAGAAAACATT | chr4 | 87685802 | 87685929 |
| AMPL7159617425 | TGTGTGCCGTATGTGTCTATGG | CTTGAGTTTGATCACCATTTTCCTCTTTC | chr18 | 3134581 | 3134855 |
| AMPL7155328687 | CATACTGCTTGGAATGTGTGAGGAT | TCCATTTCTAGTCTTCATTGAGAAAATGCT | chr4 | 87610187 | 87610459 |
| AMPL7159772206 | AAAACTCAGGTCAGGAAAAGAAGCT | CAGTGGTAAGCTTAGGTACCTATTTCAG | chr7 | 124463937 | 124464211 |
| AMPL7155328714 | GACTCATACAGATGCAGTTAATCTGCT | ACCTCCACATAAGGAAAAACCTTGAAATAA | chr4 | 87696350 | 87696614 |
| AMPL7160345524 | GACATTTAATGGGATCTGCAGTACTTG | CCTTGTTTTCTGGAGTCCCAAAATAC | chr6 | 138200172 | 138200446 |
| AMPL7155328717 | AATTGTAGACTAAGAAAGGGAAGAATGAGG | AGTCTGCTTGTTGCTAAATTTCTATACTGT | chr4 | 87653737 | 87653975 |
| AMPL7160375528 | GGGAGCGTTTCACTTGTGC | GCAGAACCTACTTCTGAGCATCG | chr8 | 42129102 | 42129262 |
| AMPL7155328718 | CCCTTCATTTGGTGGACCAAATT | CTTTAGATGTTGGAGATTGTCCCTTTTCT | chr4 | 87688838 | 87689015 |
| AMPL7160375530 | GTGAGAGGGACAAAAGTTTGCC | GACCAGCTCATACTGATGTCGT | chr8 | 42129410 | 42129629 |
| AMPL7155328749 | TTTTCCCTTAGGAAGTAGAACTCCTCA | GCTTGCTCTTTGTCTACATTTACACAAAT | chr4 | 87695515 | 87695738 |
| AMPL7160387580 | TTTTTACATTAACACTTCCCTCCTACCTT | CATGAGGTATGGGACAAAGAAGGTA | chr4 | 87672135 | 87672319 |
| AMPL7155328753 | CAATTACAAACCAGTAAGCCTAGCTGA | TAGCCCATTGTCTTTATTTGACCTATCC | chr4 | 87593504 | 87593765 |
| AMPL7160387581 | AATGATGAGATGGATTGGCCTTTCA | GAACAATTCATGATGAAACCACCACA | chr4 | 87691170 | 87691443 |
| AMPL7155328770 | TGTAATATCCAAAGCCACCGAGAAA | GCTATTTTGCTTTGTTAAATGAGAAAACCA | chr4 | 87684181 | 87684395 |
| AMPL7160387582 | CATCCAGAAGAGACAGTTACAGTGAC | CTTTGAGCGGTTCACATTATATTGATGAT | chr4 | 87692457 | 87692719 |
| AMPL7155328778 | TGTAAGATACAGCTTCAGTATACCATTGGA | CTTACTATGCTGGCATTAATTTGCTCC | chr4 | 87690753 | 87691027 |
| AMPL7160387584 | AGGTCAAGGCTACAATGTCCAAG | GATGTGCCATTTTACATTCCCATTACC | chr4 | 87701500 | 87701686 |
| AMPL7155328791 | CCATGCCCAGCCTATATATTTCTTCA | TGCCGTTTTAGCATGATCTCTTGATT | chr4 | 87643188 | 87643427 |
| AMPL7160387586 | GGACCACTGCCTACAACTGTTG | CCAACAGGGAAAGATTTCATTCACTTAC | chr4 | 87728817 | 87729078 |
| AMPL7155328797 | GACTGGACCTAGGCATATTTATCAGTTC | GACTCCCTCCAGACTCACACTAT | chr4 | 87679864 | 87680138 |
| AMPL7160387587 | GCCAGACCATGATACACCTTCT | GCTTACTCAGCCAATATCTTGTACTCAC | chr4 | 87730966 | 87731165 |
| AMPL7155328815 | GTCTTTTTCTCTTTAGGGAGGTGTGAAT | GCATCAAAGAGTTTCTTAATGCTACAGTTT | chr4 | 87686532 | 87686793 |
| AMPL7160389066 | ATATCCAGACCCACTTCGAGAGCAAGA | TCTTAGCTCCATCCTCGGTCAA | chr1 | 27105950 | 27106174 |
| AMPL7155328816 | AAGAGACGATGAACTATGAAAAGTTATGCA | AGAGTGGAGGCTTTTGTATGTCTAGT | chr4 | 87622189 | 87622441 |
| AMPL7160389779 | CTGTTGGGACCCAGCCAAACT | CACTGGTAGCCAGCGAGTTA | chr12 | 49431206 | 49431423 |
| AMPL7155328821 | TCTGAAGAGGCTATCCTGCTCA | CCAAAGATTCTGCATCAGATCTTGACAT | chr4 | 87671765 | 87672018 |
| AMPL7160391012 | ACAAGGTTCGGAGCGCTG | TGAATGAATCAACGAAGGAGTGAGT | chr6 | 44233248 | 44233503 |
| AMPL7155352568 | CCTCTCCTAGCAAGTCTCCATTC | AGATTAGGCAACCGAATGAGGAAG | chr1 | 27101514 | 27101745 |
| AMPL7160391014 | ATGGCCAGAGGAAAAACATTGAGAAGCT | CATTCTAACAGATAACGAGGTGCCTTA | chr6 | 401571 | 401805 |
| AMPL7155373307 | AACACAAGACAAAAATCCAACAAGCAATA | CAGCCTCAAGATCCTCTCCAAAG | chr2 | 136872314 | 136872526 |
| AMPL7160391046 | CTCGTCGTACTCATCATAAATTTCTCCATT | ATGTCTTTTTGGAAATTCCTTTCTTGACAG | chr12 | 23687222 | 23687486 |
| AMPL7155373309 | GCTTGATGATTTCCAGGAGGATGAA | GACAGATATATCTGTGACCGCTTCTAC | chr2 | 136872684 | 136872954 |
| AMPL7160391048 | ACGCAGATAGGCCATCTTTAGATAAC | CCTTCACTCATCTCCCAAATATAAACCA | chr12 | 23737350 | 23737620 |
| AMPL7155373313 | AGACTGATGAAGGCCAGGATGA | TCATCCTGGTCATGGGTTACCA | chr2 | 136873103 | 136873322 |
| AMPL7160399420 | GGGCCAGATAAAACAGTACATATAAATAAAAAG | CCAGCATGCAGTCCCAGATC | chr9 | 139390305 | 139390569 |
| AMPL7155374328 | GCCAGTTTTTGTCCCTGAACAAG | TCTTTAGGGCTTTAAACCTCTAGAGGTTTA | chr14 | 103336625 | 103336894 |
| AMPL7160404392 | GTGCGATTGCCAAAGAAAGGGT | CTCACTCTCCCTCTTGCCTTTTA | chr19 | 13321307 | 13321546 |
| AMPL7155519658 | TCTTATTCCCACAGTGTATCGGCTA | GAGAAGGTGAAGTGCTTGATTTTCTTAC | chr13 | 49033810 | 49033997 |
| AMPL7160490032 | ATCGAGAAAGGCACCGGGACTTT | CTTTTGGTGGGTCAGATGTGAC | chrX | 70465846 | 70466013 |
| AMPL7155521204 | CTTTATTTTGTAGCCATCCAGTCCAATG | GGTCAAGGTAATCACAATCACCATCTAC | chr1 | 27056129 | 27056382 |
| AMPL7160642251 | GTAGCTACATCACCACTGTGTCT | GAATACAGGTGGGTTGTCTTGAGT | chr4 | 126239680 | 126239945 |
| AMPL7155522848 | CTGCTTGGCAGTCTCTGACAATAT | TTCTCAGTCAAGCTGCATGTATGT | chr4 | 153273710 | 153273906 |
| AMPL7160642253 | CTGGGCAGCTTACCACAGCAAA | GGTTTTGCTTCAGACTATAGAGTACCATG | chr4 | 126240100 | 126240372 |
| AMPL7155531282 | CCTCCTGAGGTAGGTCATGCA | CAGGAAGTCGTACACCCACAT | chr3 | 183209506 | 183209769 |
| AMPL7160718625 | GTCCCAGCATCATTGATGGTGA | GTTCACATTCTGGATGATCAGCGTA | chr19 | 42383092 | 42383271 |
| AMPL7155531286 | GACCTCATTTTTCAATGTCACGCAT | TGTTGTCCCTGCCTTCATGTAC | chr3 | 183217388 | 183217653 |
| AMPL7161055331 | GGGTCTATGGGATTTGGGTTACTT | TTGTCTGTCTGTCTGTTTGTCGTTATAG | chr16 | 3832825 | 3832955 |

Amplicons : Series 2

| amplicon_name | forward_sequence | reverse_sequence | chromosome | amplicon_start | amplicon_end |
| --- | --- | --- | --- | --- | --- |
| BTK_EX15_1MPLXID1012 | CCCCTCAACCATGTATGATATATCTT | TGTCTGTGAAGCCATGGAATAC | X | 100610955 | 100611094 |
| BTK_EX15_2MPLXID1013 | CGGTGAAGGAACTGCTTTGA | TCATCACTGAGTACATGGCC | X | 100611047 | 100611191 |
| BTK_EX15_3MPLXID1014 | ATCTCCCTCAGGTAGTTCAGG | GAAGACTAGTTCCTTGCCTTTCC | X | 100611140 | 100611284 |
| BTK_EX15_4MPLXID1015 | TGAAGATGGGGCGCTG | CCCTTCCTCCCCAGC | X | 100611192 | 100611331 |
| BIRC3_EX6_1MPLXID287 | AAGGCTATCCTAATATGTGTTAAATTCTTTG | GGCTTCTACTAAAGCCCATTTC | 11 | 102201689 | 102201828 |
| BIRC3_EX6_2MPLXID288 | CATGATGAATACTCCTGTGATTAATGC | CTGCATTGAGTAAGTCTAACACAAG | 11 | 102201773 | 102201918 |
| BIRC3_EX6_3MPLXID289 | CCTAGCAACTGGAGAGAATTATAGAC | GAAAATAGACTGTATTAATAAATCTACATACTTGA | 11 | 102201857 | 102202003 |
| BIRC3_EX6_4MPLXID290 | AAGAGGAGAGAGAAAGAGCAAC | TTGAGTATATTTTCAGATATGCTAGATGAGT | 11 | 102201934 | 102202073 |
| BIRC3_EX7_1MPLXID291 | TGTGAGCAGAGTTTGAACATGT | TCCGGCAGTTAGTAGACTATCC | 11 | 102206649 | 102206788 |
| BIRC3_EX7_2MPLXID292 | CATTTGACTTGTGTAATTCCAATCCT | GGCTGCAATATTTCCTTTTACTAAAATC | 11 | 102206741 | 102206893 |
| BIRC3_EX7_3MPLXID293 | CAGAAGACACAGACGTCTTTACA | TTTTCTATCACTCACCAAATAAATGCTC | 11 | 102206822 | 102206966 |
| BIRC3_EX7_4MPLXID294 | ACTGTATTCAGAAACTCTCTGCAA | TTTTCAGTAGTCATAAATTATTAATAAGAGCGTAT | 11 | 102206894 | 102207033 |
| BIRC3_EX8_1MPLXID295 | TCCATAGCTAAATATTAACCTTATTTGTCATAG | ACTAAGAAAATAAAGACAATGTTCTCTATTGATT | 11 | 102207432 | 102207586 |
| BIRC3_EX9_1MPLXID296 | AACATTGTCTTTATTTTCTTAGTTTTTCA | GTCCATACACACTTTACATGTTCTTTC | 11 | 102207564 | 102207704 |
| BIRC3_EX9_2MPLXID297 | TGGAAGAACAATTGCGGAGA | CCTACAAATAGGACACTTTCTTAAAGAAG | 11 | 102207649 | 102207794 |
| BIRC3_EX9_3MPLXID298 | AAGAAGTGTCCATAGTGTTTATTCCT | GATGTTTTGGTTCTTCTTCATGAAAGA | 11 | 102207706 | 102207850 |
| BIRC3_EX9_4MPLXID299 | AGTACAATCAAGGGTACAGTTCG | AATAAATAAAAATTTTAAGGAAACCAAATTAGGAT | 11 | 102207795 | 102207934 |
| TET2_EX3_1MPLXID851 | CAACTAGAGGGCAGCCTTG | TGGAGCTTTGTAGCCAGA | 4 | 106155058 | 106155203 |
| TET2_EX3_2MPLXID852 | CTCCCATTTGCCAGACAGAA | CTGGCTTCCCTTCATACAGG | 4 | 106155164 | 106155309 |
| TET2_EX3_3MPLXID853 | TGGCACTCTTTCAAAAGTTATTATGG | AAGGTTCACTAACTGTGCGTT | 4 | 106155259 | 106155403 |
| TET2_EX3_4MPLXID854 | GGGTATTCCAAGTGTTTGCAAAA | TTCTTTCTTGGCTTACCCCG | 4 | 106155349 | 106155493 |
| TET2_EX3_5MPLXID855 | AAAAGGCTAATGGAGAAAGACGT | GTGAAATCTTTAACTGCATTTTCTTGG | 4 | 106155446 | 106155590 |
| TET2_EX3_6MPLXID856 | AGTGATAAGAAAGAATCTGTGAGTTCT | GGTAATTAGCACTTTTCCCCTCC | 4 | 106155532 | 106155676 |
| TET2_EX3_7MPLXID857 | AAAATCCAGAGCTTCAGATTCTGA | TTCACCATGTGTGTGTTCCA | 4 | 106155623 | 106155771 |
| TET2_EX3_8MPLXID858 | CCTAATGGTGCTACAGTTTCTGC | CCTGACTGTTAATGGCATTTATGTG | 4 | 106155721 | 106155865 |
| TET2_EX3_9MPLXID859 | AGATTGTGTTTCCATTGCGG | CTCAGAGTTAGAGGTCTGTGC | 4 | 106155804 | 106155948 |
| TET2_EX3_10MPLXID860 | TACCTCAGGGCAGATCAATTC | CTGAAAGGAACAGGTATTTAGCATTG | 4 | 106155906 | 106156050 |
| TET2_EX3_11MPLXID861 | GATGCTGATGATGCTGATAATGC | CTAGCTTTGTGGTTCCCTGG | 4 | 106155988 | 106156138 |
| TET2_EX3_12MPLXID862 | GATATGCCCATCTCCTGCAG | ACCATTCATTTCATTTTGTTTTAAATACCG | 4 | 106156089 | 106156233 |
| TET2_EX3_13MPLXID863 | CAGCAGCAATTTGCAAGCT | AAGCAATTGTGATGGTGGTG | 4 | 106156167 | 106156314 |
| TET2_EX3_14MPLXID864 | AAGGATTCCTTTTCTGCCACTA | AGTGGTGGTGTTCTTCTAAAACT | 4 | 106156264 | 106156408 |
| TET2_EX3_15MPLXID865 | AAGGAAAAAGCACTCTGAATGGT | ACATGTGTAGATGGATTAGGACTC | 4 | 106156361 | 106156505 |
| TET2_EX3_16MPLXID866 | ATAGAGGGTAAACCTGAGGCA | GAACAGTCATTGTCCCTGCA | 4 | 106156450 | 106156594 |
| TET2_EX3_17MPLXID867 | GTGTGAACAGGAATGACATACAGA | TTTCTCATCAACTGCTGGCA | 4 | 106156550 | 106156703 |
| TET2_EX3_18MPLXID868 | AGTGGAGAGCTACAGGACA | GGGCCTTCAATTCAATCCATC | 4 | 106156663 | 106156807 |
| TET2_EX3_19MPLXID869 | CCCAACACAGCACTATCTGAA | TTGGAGGTCATTTGATTGGAGA | 4 | 106156761 | 106156907 |
| TET2_EX3_20MPLXID870 | GCCATCAATTCTTCAGTATCAACC | CTTGGTACATTTGTGACTTGTG | 4 | 106156857 | 106157005 |
| TET2_EX3_21MPLXID871 | TTACACCCAGAAAACAACACAG | CTTTTGGTAAATGGTCTGTTTTGGA | 4 | 106156956 | 106157110 |
| TET2_EX3_22MPLXID872 | AAAACCCTCACACCAGGTG | AGTGCTGTTTCAACACTGGG | 4 | 106157061 | 106157206 |
| TET2_EX3_23MPLXID873 | AAGAGCAGATTCCCAAACTGAA | ACTCTGGGATGGTTGTGTTT | 4 | 106157154 | 106157304 |
| TET2_EX3_24MPLXID874 | CAAACTCACACCTTTTGCAACA | TGAGGAAAAGTCTGGAGTATTTCC | 4 | 106157240 | 106157384 |
| TET2_EX3_25MPLXID875 | CAACAGCAGCAAAAATTACAAATAAAGAA | ACTGATTTTCACCATGAAAACATTCT | 4 | 106157326 | 106157470 |
| TET2_EX3_26MPLXID876 | CTTTGGCCAGACTAAAGTGGA | TGATTTCATGGTCTGACTATAAGGG | 4 | 106157424 | 106157574 |
| TET2_EX3_27MPLXID877 | GAGGAAGTACAGAATATAAATCGTAGAAATTC | GCAAAAAGTTCAGGATGTGTAGTC | 4 | 106157518 | 106157663 |
| TET2_EX3_28MPLXID878 | ATACACACCTAGTTTCAGAGAATAAAGAA | GCTCCTGTTCTTGAAAGCAC | 4 | 106157609 | 106157761 |
| TET2_EX3_29MPLXID879 | AAATAATGTGATCCCAAAGCAAGATC | TACCTTTGCTGAGCAAGTTGC | 4 | 106157706 | 106157855 |
| TET2_EX3_30MPLXID880 | ACCAAGATATGTCTGGTCAACAA | GCAGCATGCTTTTGAGTGTC | 4 | 106157807 | 106157951 |
| TET2_EX3_31MPLXID881 | GAGGAAGTCACACTCAGACC | TTAATTGGCCTGTGCATCTGA | 4 | 106157900 | 106158044 |
| TET2_EX3_32MPLXID882 | AACCCCAAACTGAGTCTTGC | CTTGCAGGTGGATTCTCTTG | 4 | 106157999 | 106158143 |
| TET2_EX3_33MPLXID883 | TGCCTGTATGCACACAGC | CTTGGCGTGAAACTGCTTC | 4 | 106158069 | 106158213 |
| TET2_EX3_34MPLXID884 | CATCATTGAGACCATGGAGCA | AGCAGTGGTTTGTCTAGTCAAA | 4 | 106158168 | 106158312 |
| TET2_EX3_35MPLXID885 | GTTGAAATGTCAGGGCCAGT | AATTATTGAGAACAGAAGCAGCTG | 4 | 106158265 | 106158409 |
| TET2_EX3_36MPLXID886 | CAACTTCTTCAGAAAAGACACCAAC | CACATCTGCAAGATGGGAAATC | 4 | 106158353 | 106158505 |
| TET2_EX3_37MPLXID887 | TTTATTGGATACACCTGTCAAGACT | AAAAGAAGGAAAATCCCATATCTGAAG | 4 | 106158453 | 106158592 |
| TET2_EX4_1MPLXID888 | AGTATAATTGAGGTCTAAAATAATAATCTTCTATT | ATCTGCCCTGTGCCTTTG | 4 | 106162451 | 106162614 |
| TET2_EX5_1MPLXID889 | ATGCTCAAATGTTCAAATATTTTGATTGC | CTTTACCAGTATAGATGACTCTTTCAATC | 4 | 106163907 | 106164046 |
| TET2_EX5_2MPLXID890 | TTTGGACAGAAGGGTAAAGCTATT | AAATCAAAATGCCCAAGATTTAAGACC | 4 | 106163992 | 106164136 |
| TET2_EX6_1MPLXID891 | ACATACATAAGTGCCCTTATCTGC | CCGCACCAAACACAGTAGC | 4 | 106164635 | 106164774 |
| TET2_EX6_2MPLXID892 | CAGAAGCAGCAGTGAAGAGAA | CGGTAAGCTCCGAGTAGAGT | 4 | 106164735 | 106164880 |
| TET2_EX6_3MPLXID893 | AAGGAATCCCGCTGTCTCT | TATCAGTGGCCGCAAAGAG | 4 | 106164833 | 106164979 |
| TET2_EX7_1MPLXID894 | ATCCATAGCAATGAATTTGGTCTTTT | TTCTGGCAAACTTACATCCATTG | 4 | 106180740 | 106180879 |
| TET2_EX7_2MPLXID895 | CTCTTTTGGTTGTTCATGGAGC | GCTAATGAATTCTCTATGAAAAATAAAGCG | 4 | 106180827 | 106180981 |
| TET2_EX8_1MPLXID896 | TGTTTGGGATTCAAAATGTAAGGG | GGACAGGTTTTGCAAATGAGAC | 4 | 106182812 | 106182951 |
| TET2_EX8_2MPLXID897 | CACTTTATACAGGAAGAGAAACTGGA | CAAGTAAGTTGTTACAATTGCTGCC | 4 | 106182904 | 106183049 |
| TET2_EX9_1MPLXID898 | TTTTCRGTGTAAGAGTAAAACTAA | CCAAACATGCAGTGACCC | 4 | 106190708 | 106190848 |
| TET2_EX9_2MPLXID899 | GTCTGAAGGAAGGCCGTC | AGAACAGACTCAACAGCTGC | 4 | 106190804 | 106190948 |
| TET2_EX10_1MPLXID900 | ACCTGTAGTTGAGGCTGTAATG | TCATCCACGTCAGAGACTTTG | 4 | 106193682 | 106193821 |
| TET2_EX10_2MPLXID901 | GATGAGCAGCTTCACGTTCT | GCTCTGCTAACATCCTGACTT | 4 | 106193772 | 106193916 |
| TET2_EX10_3MPLXID902 | TCAGGTACTGAGTTCTTTTCGG | GGGCTGACTTTTCCTTTTCATTT | 4 | 106193870 | 106194024 |
| TET2_EX10_4MPLXID903 | GAAAAGCTTTCCTCCCTGGA | CCACACAACACATTTATCTACAAATGC | 4 | 106193967 | 106194118 |
| TET2_EX11_1MPLXID904 | ATCCTCACTAGCCTTCATAAAATAATCA | CTGCTTCTGTAGAGGCTGG | 4 | 106196130 | 106196269 |
| TET2_EX11_2MPLXID905 | TTTCAGGACCAGTCATgcag | GATCCAGAAGCAGAATAAGAGTTGA | 4 | 106196217 | 106196361 |
| TET2_EX11_3MPLXID906 | ACATCACCCTCAGACAGAGT | AAGTTCATAGGGCTGGTGC | 4 | 106196314 | 106196457 |
| TET2_EX11_4MPLXID907 | AACTCTTCACACACTTCAGATATCTATG | GATATTGGGTATTCTGATTCAAAAGCC | 4 | 106196408 | 106196552 |
| TET2_EX11_5MPLXID908 | TTGAATTCTTCTAATCCCATGAACC | TACAGATCCATCGGCTGAGA | 4 | 106196492 | 106196643 |
| TET2_EX11_6MPLXID909 | CCCATATCTGGGTTCCTATTCTC | AGATGTAAAACTCTGGCTATTTCCA | 4 | 106196596 | 106196740 |
| TET2_EX11_7MPLXID910 | TAAGCTCAGTCTACCACCCA | GATGTACATTTGGTCTAATGGTACAAC | 4 | 106196671 | 106196816 |
| TET2_EX11_8MPLXID911 | GAAACCAAAATATGCAGGGAGATG | TGCTCAGATTGGGTGGTAATC | 4 | 106196757 | 106196903 |
| TET2_EX11_9MPLXID912 | GATGGCCACTTCATGGGAG | GAAGAGAGCTGTTGAACATGC | 4 | 106196855 | 106196999 |
| TET2_EX11_10MPLXID913 | TAATCCATAACTACAGTGCAGCTC | GCAGTTCTATCATGGTTAAGAGCT | 4 | 106196952 | 106197096 |
| TET2_EX11_11MPLXID914 | CACAGCTAATGGGTTATCAAAGATG | TTGTCCTCTGCACCAGAAG | 4 | 106197043 | 106197195 |
| TET2_EX11_12MPLXID915 | CAGCCATTGGCACTAGTCC | CACACTCAATGAGAATTGACCCA | 4 | 106197149 | 106197293 |
| TET2_EX11_13MPLXID916 | CTTTCTGGATCCTGACATTGGG | AAAAGACGAGGGAGATCCTG | 4 | 106197226 | 106197371 |
| TET2_EX11_14MPLXID917 | CCTTTAAAGAATCCCAATAGGAATCAC | CACACTCTTCCTCTTTCTCACG | 4 | 106197320 | 106197464 |
| TET2_EX11_15MPLXID918 | CCAAAACATGGCTTGGCTC | AAGTTTCATGTGGCTCAGCA | 4 | 106197395 | 106197545 |
| TET2_EX11_16MPLXID919 | CAGACTATGTGCCTCAGAAATCC | TTACTGTGGAGTCTGTGGTCA | 4 | 106197477 | 106197620 |
| TET2_EX11_17MPLXID920 | CAAGTCTCTTGCCGAAAGGA | GGTCTTTTCAAGTGAGGTAACCA | 4 | 106197571 | 106197715 |
| PRDM1_EX1_1MPLXID686 | CTGTTATTTTCCCGAACATGAAAAGA | TCCCCTACTGCCTAGTTAGAAA | 6 | 106546922 | 106547075 |
| PRDM1_EX2_1MPLXID687 | ACTTCCTTTTACATGCCTGTCT | TTTTGCTCCCGGGGAG | 6 | 106547139 | 106547278 |
| PRDM1_EX2_2MPLXID688 | CGCTATGTGAATCCAGCACA | TAGTGAAGCCTTTCTGCAAA | 6 | 106547241 | 106547392 |
| PRDM1_EX2_3MPLXID689 | GCCAACCAGGAACTTCTTGT | GGCAGAACCGACATTACTGG | 6 | 106547337 | 106547481 |
| PRDM1_EX3_1MPLXID690 | TGTTACTCAGGTTTTCTCAAGAAGG | TTTTCTCAGTGCTCGGTTGC | 6 | 106552602 | 106552741 |
| PRDM1_EX3_2MPLXID691 | TTCTTTATTTCAGCACAAACACAGAG | GAGGTCCTTTCCTTTGGAGG | 6 | 106552687 | 106552833 |
| PRDM1_EX3_3MPLXID692 | CAGCGTGAAAGAAATCCTAAAATTGG | TAAACGACCCGAGGGTAGAA | 6 | 106552779 | 106552931 |
| PRDM1_EX3_4MPLXID693 | AAAGGACCTCGATGACTTTAGAAG | AGTGATGTACGTGGGTCTCT | 6 | 106552866 | 106553010 |
| PRDM1_EX3_5MPLXID694 | GAAAGCTTCCCTGGCCTAC | TATTCCCAGGGCTGCTGT | 6 | 106552965 | 106553110 |
| PRDM1_EX3_6MPLXID695 | CGACCAAAGCCTCAAGAGC | TAGCCAGGGTAGGAGCC | 6 | 106553064 | 106553210 |
| PRDM1_EX3_7MPLXID696 | TTACTTGAACGCGTCCTACG | CATTACAATTCATGCCGTAGGG | 6 | 106553160 | 106553305 |
| PRDM1_EX3_8MPLXID697 | GCTTTCATCCCCTCGTACAA | CCGAGGAGATTGCTGTAGAC | 6 | 106553236 | 106553390 |
| PRDM1_EX3_9MPLXID698 | CAACTTTGGCCTCTTCCCG | GATGCTCCGGCTGGAG | 6 | 106553340 | 106553485 |
| PRDM1_EX3_10MPLXID699 | CCTCAGATGGAGCCCG | TTCCCGCCGTGGGAG | 6 | 106553447 | 106553596 |
| PRDM1_EX3_11MPLXID700 | CATGAAGGACAAGGCCTGTA | TTTTAATGAGATTCATGGCTTCGTC | 6 | 106553547 | 106553689 |
| PRDM1_EX3_12MPLXID701 | CAAAGCTACCTCAGCAGCG | AACGTTGCATTCGTACTTGATCT | 6 | 106553628 | 106553772 |
| PRDM1_EX3_13MPLXID702 | CCGCTGAAGAAGCAGAACG | CAAGCTAAATACAAACACAAGCATG | 6 | 106553728 | 106553872 |
| PRDM1_EX4_1MPLXID703 | CTTGAGTCTTGGAGCAGAAATGT | TAAAGCCCTTGTTGCAAGTCT | 6 | 106554170 | 106554309 |
| PRDM1_EX4_2MPLXID704 | GGAGAACGGCCTTTCAAATG | CAAAGGTCAGAAGGTCTACCC | 6 | 106554267 | 106554411 |
| PRDM1_EX5_1MPLXID705 | TCCCGTTGGCAACTCTTAATC | TTCTCTCCAGAATGGAGTCG | 6 | 106554714 | 106554853 |
| PRDM1_EX5_2MPLXID706 | CAGCACCAGCAATCTCAAGA | CACTGGGAGCACTTGTGG | 6 | 106554806 | 106554958 |
| PRDM1_EX5_3MPLXID707 | GAAACTGCACAAGCGTCTG | GTCAGATCTTCCAAGGGCAG | 6 | 106554905 | 106555054 |
| PRDM1_EX5_4MPLXID708 | GTTCACCTGAAAGGGAACTGC | TCACACTGATGTCATCCTCCA | 6 | 106554993 | 106555137 |
| PRDM1_EX5_5MPLXID709 | ATCAGTGACAATGCTGACCG | CATTCCCCATGTTTCTTTGCAA | 6 | 106555086 | 106555230 |
| PRDM1_EX5_6MPLXID710 | AGAGAAAGAAGAAACTGGCCTG | CTTTACAGGTACCAGAGGTAGTG | 6 | 106555178 | 106555325 |
| PRDM1_EX5_7MPLXID711 | GATCTACCCCTCATGAAGTTGC | CTGACTCACCAAGTCATAACTTAAGA | 6 | 106555269 | 106555413 |
| ATM_EX2_1MPLXID120 | ACAGACAGTGATGTGTGTTCTG | ACTAATCACACTTATTTCAAGGAAAAATTGA | 11 | 108098318 | 108098472 |
| ATM_EX3_1MPLXID121 | TTTTCCTTGAAATAAGTGTGATTAGTAACC | TCCTTGTTTGGAATCTGAATGC | 11 | 108098447 | 108098586 |
| ATM_EX3_2MPLXID122 | GATTCGAGATCCTGAAACAATTAAACAT | TAATATCACAACAGAAATAAATATGAAAGAGAAAA | 11 | 108098529 | 108098683 |
| ATM_EX4_1MPLXID123 | TCAACGAGTTTCTGAAATTGC | TCCTGCATCTTTTTCTGCCT | 11 | 108099867 | 108100006 |
| ATM_EX4_2MPLXID124 | ACCAAATGTATCAGCCTCAACA | CTCACGCGACAGTAATCTGTT | 11 | 108099956 | 108100105 |
| ATM_EX5_1MPLXID125 | TAATAGTAATTTCCCAAATGGAATTATTTAA | CCATGATATAATTTAAGAGTTCTTGACATTTTAG | 11 | 108106295 | 108106441 |
| ATM_EX5_2MPLXID126 | TTTGTTTATTTTGAAATAGGAGCACCTAG | CTCACAGAAAGAATGTCTTTGAGTAGT | 11 | 108106378 | 108106523 |
| ATM_EX5_3MPLXID127 | ACGGAGCTGATTGTAGCAAC | GTGCTTTCTTTGGTGAAGTTTCA | 11 | 108106475 | 108106627 |
| ATM_EX6_1MPLXID128 | TCCTTTTTCTGTATGGGATTATGGAA | GTTTCAGATAGAGCCTGAAGTACA | 11 | 108114576 | 108114715 |
| ATM_EX6_2MPLXID129 | AATTGTAACATTTAATACAKTTTGATTTTTAAAAA | CCTTTGGTAACAGCATGAATT | 11 | 108114613 | 108114773 |
| ATM_EX6_3MPLXID130 | CTTCACAAGATSTTCATAGAGTTTTAGTG | AGAAAAAGAGATTAGATTACCTCGC | 11 | 108114716 | 108114865 |
| ATM_EX6_4MPLXID131 | ATGCTGTTCTCAGACTGACG | TCTAAAACATGGTCTTGCAAGATCA | 11 | 108114774 | 108114913 |
| ATM_EX7_1MPLXID132 | CCCCTGTTATACCCAGTTGAG | TAATTCACACACTCGAATTCGAAAG | 11 | 108115475 | 108115614 |
| ATM_EX7_2MPLXID133 | GCTCTTACTATCTTCCTCAAGACTTT | TGCAGTTGAAATAATTCAATAATGACTTCT | 11 | 108115555 | 108115709 |
| ATM_EX7_3MPLXID134 | GACTCAACATAGGCTTAATGATTCTTTAAA | CTATGTTTGAATGAAGAAGCAAATTC | 11 | 108115650 | 108115804 |
| ATM_EX8_1MPLXID135 | GCTTGACAGCTGAATAATTTTGTGG | GCAGATCATATAAGTTGTATAAAATACTTCTCC | 11 | 108117605 | 108117744 |
| ATM_EX8_2MPLXID136 | TTTGGATTACAGGTGCTTATGAATCA | CAAATTTTCTTTGACGGCAATATTACG | 11 | 108117679 | 108117824 |
| ATM_EX8_3MPLXID137 | AAGTAGAGGAAAGTATTCTTCAGGATTT | GCCCAAAATGCCCAGTTTAAA | 11 | 108117770 | 108117925 |
| ATM_EX9_1MPLXID138 | gatacgagatcgtgctgttcc | TTGAGAAATCTCCAAGGATCTGG | 11 | 108119559 | 108119698 |
| ATM_EX9_2MPLXID139 | GGATTTATTTTTATTTTACAGGTTTTTAATGAAGA | AGGTGATCTTTTATTACTTCCCA | 11 | 108119639 | 108119793 |
| ATM_EX9_3MPLXID140 | GTCCCTTGCAAAAGGAAGAAAATAG | AACATACAAGAGATTAAAATGACACTGAAT | 11 | 108119738 | 108119880 |
| ATM_EX10_1MPLXID141 | TCTAATTAGGATATTGTAAGAGTACCATGTC | AAACTTGCAGGATACTTTGATATTAA | 11 | 108121328 | 108121472 |
| ATM_EX10_2MPLXID142 | TTTTAGGCTACAGATTGCAACCC | GCATCGTAACACATATGGTGTAC | 11 | 108121422 | 108121566 |
| ATM_EX10_3MPLXID143 | CTACCCCAACAGCGACATG | GGTAATACACCAAATTTTATTCCAGAGTT | 11 | 108121519 | 108121665 |
| ATM_EX10_4MPLXID144 | CCTAGAAAGCTCACAAAAGTCAGA | CTCTGTCAACCTCAACTAAACTA | 11 | 108121605 | 108121756 |
| ATM_EX10_5MPLXID145 | TTTGGCTTACTTGGAGCCAT | TGTCTGTGTGTGTTTATCTGTAAGT | 11 | 108121705 | 108121849 |
| ATM_EX11_1MPLXID146 | CAGGCACTGTCCTGATAGATAAAG | ATTTTTACCGTTCCTGGAACTATACT | 11 | 108122487 | 108122626 |
| ATM_EX11_2MPLXID147 | GTATGCTGTTTGACTTTGGCA | GTCACCCTCTAACTGATAGAATAAGAG | 11 | 108122571 | 108122720 |
| ATM_EX11_3MPLXID148 | GCTTTTCTTTAAAGGAATCAATAATGAAATGG | GAGAGAGCCTGATAAAACAAACAAAA | 11 | 108122662 | 108122816 |
| ATM_EX12_1MPLXID149 | AGTCAAGATTTATAGCTAAACATGGATGT | TGAGACTCACAAGAATTTTCTCCA | 11 | 108123448 | 108123587 |
| ATM_EX12_2MPLXID150 | TTCTTTGTAGTAATTTTCCTCATCTTGTAC | AAAGCTATAGCTTAAAATGATAAAGAGCATT | 11 | 108123534 | 108123684 |
| ATM_EX13_1MPLXID151 | AGGCAAAGCATTAGGTACTTGG | AAGTCCATCTTGTCAAAAGTTGTC | 11 | 108124488 | 108124627 |
| ATM_EX13_2MPLXID152 | AACTTTCATTCTCAGAAGTAGAAGAACT | CAGCGATCCAGTGATTCCTT | 11 | 108124567 | 108124720 |
| ATM_EX13_3MPLXID153 | GCTTCTCTGTCCACCAGAATC | CTTCCTAACAGTTTACCAAAGTTGAAT | 11 | 108124678 | 108124830 |
| ATM_EX14_1MPLXID154 | ACAAAAGATAGAGTATACtaaattatttatgaaat | ACACCCATRTAACAGTAGCAG | 11 | 108126876 | 108127018 |
| ATM_EX14_2MPLXID155 | GTTCACGTCTTTTGGTGGGT | CAGGAATACATTTCATTCAAATTTATCCGA | 11 | 108126970 | 108127123 |
| ATM_EX15_1MPLXID156 | TAATTTTAACTGGAATTTGCATTTTTCCTTC | ATAGCTGCATCATATTTCTCAAGGA | 11 | 108128165 | 108128304 |
| ATM_EX15_2MPLXID157 | AAAATAAGACAAATGAGGAATTCAGAATTG | GGGTGACAGAGAAAGATCCTATC | 11 | 108128248 | 108128389 |
| ATM_EX16_1MPLXID158 | TGTTGCTTGGTTCTTTGTTTGT | AGAATCAAAATATGATAGCAAAACAGGAA | 11 | 108129680 | 108129841 |
| ATM_EX17_1MPLXID159 | GCCATCTTGAACATCTTTGTTTCT | TAGATTCATGGATGACTGATCCTC | 11 | 108137863 | 108138002 |
| ATM_EX17_2MPLXID160 | ATGGAAGATGATACTAATGGAAATCTAATG | CCCAAGTAGTAAATATGTATTTACCTATGGT | 11 | 108137943 | 108138093 |
| ATM_EX17_3MPLXID161 | TTTAACGATTACCCTGATAGTAGTGTTAG | CAATGAGGCCTCTTATACTGCC | 11 | 108138003 | 108138142 |
| ATM_EX18_1MPLXID162 | AAGAGGAGGAAATTTGAGTTAATATGACT | CTAAGAAAAGYAGATCTTGCTTTGACAG | 11 | 108139054 | 108139193 |
| ATM_EX18_2MPLXID163 | AGGTGCCATTAATCCTTTAGCT | CAATTTCCTCCGAATATCAGCTG | 11 | 108139135 | 108139279 |
| ATM_EX18_3MPLXID164 | CTGCTCAGACCAATACTGTGT | AAAATCCAAGAGCTTCTTCATTTAACG | 11 | 108139227 | 108139371 |
| ATM_EX18_4MPLXID165 | TTAATGTTAATTGATTCTAGCACGCTAG | TGTGATTCTTACATGTCAACTCATTACA | 11 | 108139280 | 108139419 |
| ATM_EX19_1MPLXID166 | AGTAAATGATTTGTGGATAAACCTGATTT | TTCTTACGATAGTGGTTTCAGAAGTT | 11 | 108141741 | 108141880 |
| ATM_EX19_2MPLXID167 | CTCCTACCATCTTAGTATCTAATGCTTT | TTTATAACTTTAAAGTGAACATAACATAAGGTTTT | 11 | 108141776 | 108141918 |
| ATM_EX20_1MPLXID168 | TCTGTTAAGCTTATAAAGTTGAACT | CTTGTGTTCTCAGAGTCCATATTG | 11 | 108141932 | 108142091 |
| ATM_EX20_2MPLXID169 | CCTTCATGTAGTGAAAAACCTAGGT | GCATTCGTATCCACAGATAGCA | 11 | 108142038 | 108142191 |
| ATM_EX21_1MPLXID170 | ACAGAAAGACATATTGGAAGTAACTTACA | ACAGAGAATATATATTTCCTCTCCTTTGTT | 11 | 108143155 | 108143294 |
| ATM_EX21_2MPLXID171 | GGAAAACTTACTTGATTTCAGGCAT | GACTGCTTTAAAATTTTCAATGGAGATC | 11 | 108143238 | 108143387 |
| ATM_EX22_1MPLXID172 | GCAGTCTTTGTTTGTTAATGAGTAATTTTT | GTGTAAATACTTCATTTACAGGAAAGTCTT | 11 | 108143382 | 108143521 |
| ATM_EX22_2MPLXID173 | TGGGCCATTCTTAATGTAATGGG | GCAGCATTCCAAATACTTCATGAA | 11 | 108143467 | 108143618 |
| ATM_EX23_1MPLXID174 | TTTGTTCTGGAATATGCTTTGGAAAG | AGGAAGTGCTTTCAGTAACCTG | 11 | 108150130 | 108150269 |
| ATM_EX23_2MPLXID175 | CAGGACACGAAGGGAGATTC | GCAAGCATATGATAACAGCAAATACA | 11 | 108150225 | 108150377 |
| ATM_EX24_1MPLXID176 | AAATCTGGAGTTCAGTTGGGATT | CAGATTTTCTATTATAAATTTCATCCAAAGTTTCA | 11 | 108151637 | 108151776 |
| ATM_EX24_2MPLXID177 | TAAGTCCCATAGTGCTGAGAAC | CTCTTTCACAGATTTACACAGGG | 11 | 108151718 | 108151865 |
| ATM_EX24_3MPLXID178 | CTGTAGCCCTATCTGCGAAAA | TAAATTATTTCACAGTGACCTAAGGAAG | 11 | 108151808 | 108151952 |
| ATM_EX25_1MPLXID179 | TCCTACTCTAAATAATATTAACAAGCATTTAAATG | GCCATAAAGTCTTCTAAACGTCTATATC | 11 | 108153353 | 108153492 |
| ATM_EX25_2MPLXID180 | TTTGGTTCGTGCAGGTTTTAGA | AAGGAAAAGAAGATAAGTTGTATTCAGTATC | 11 | 108153423 | 108153566 |
| ATM_EX25_3MPLXID181 | CTCATTTAGATTATCTGGTTTTGGAATGG | AACAAATTTCACATATGTCATGTATAAACTTAC | 11 | 108153495 | 108153639 |
| ATM_EX25_4MPLXID182 | TTATTTTATTAAACTACACAAATATTGAGGATTTC | CACATGTGTATGGGTATGGTATGT | 11 | 108153567 | 108153713 |
| ATM_EX26_1MPLXID183 | TTATAAAATTTTACTTGGAAAAGTTATATATAACC | TCCAGTCCTCTTGAATCTGATTAG | 11 | 108154893 | 108155045 |
| ATM_EX26_2MPLXID184 | AAGTCATTTTGATGAGGTGAAGTC | CTCTCTTTGCTGTGCCATCC | 11 | 108154993 | 108155146 |
| ATM_EX26_3MPLXID185 | TTTGCCTATGAGGGTACCAGA | CTCATATCATTCAGGGAATGAAAAGTAC | 11 | 108155099 | 108155247 |
| ATM_EX27_1MPLXID186 | TATGCCTTTTGAGCTGTCTTGA | GGCTCATGTAACGTCATCAATAAC | 11 | 108158255 | 108158394 |
| ATM_EX27_2MPLXID187 | CATTAGTAATTTACCAGAGATTGTGGTG | CTTGTGAAGTTAGAGCTAGTTCTCTAA | 11 | 108158341 | 108158486 |
| ATM_EX28_1MPLXID188 | CTGGTCTATGAACAAAACTTTTTAAAACG | GACAATTGCTGATATAGGCAAATGTT | 11 | 108159644 | 108159783 |
| ATM_EX28_2MPLXID189 | ATTTTCCATCGCATGTGATTAAAGC | AAAAGAAGGAATGTTCTATTATTAAACTCATCA | 11 | 108159733 | 108159874 |
| ATM_EX29_1MPLXID190 | ATAAAGTGTATTTATTGTAGCCGAGTATCT | GTGCTTCTTATAAACATTATTTGTTTCAGC | 11 | 108160261 | 108160400 |
| ATM_EX29_2MPLXID191 | TCTTCTTGCCATATGTGAGCAA | CGTCTCGAAGAACAAAGGCC | 11 | 108160346 | 108160494 |
| ATM_EX29_3MPLXID192 | ATTACTGAAAGATATAAAAAGTGGCTTAGG | ACAGGAAGAACAGGATAGAAAGAC | 11 | 108160436 | 108160584 |
| ATM_EX30_1MPLXID193 | TGAACAAAAGGACTTCTGAATGAATTTAT | GTCACAACAAAGGGAGAAGCT | 11 | 108163258 | 108163397 |
| ATM_EX30_2MPLXID194 | TATATTAGGCCTTCTTGTATCATGGATG | GGTATAAGTGTACCAACAATAACATGAAG | 11 | 108163338 | 108163486 |
| ATM_EX30_3MPLXID195 | GTGACTTACTGTAAGGATGCTCTAG | CCTTCTAACAATACTTTATATATTTTAAATACCAT | 11 | 108163425 | 108163579 |
| ATM_EX31_1MPLXID196 | TAATCTGATCTAGGTTAATAGATTTTATCATTTAT | TCTATCACTAAGTATTTCAACAAGTCCA | 11 | 108163932 | 108164071 |
| ATM_EX31_2MPLXID197 | GTTGGCTTACTTTAAAATTATTTCTCTCCt | GTCAGGAAAAGGATCTAAAAGCTTAATC | 11 | 108163980 | 108164129 |
| ATM_EX31_3MPLXID198 | AACAAGGATAATGAAAACCTCTATATCAC | GAAATTTTTATTACCTCCAAGAGTGAAAAG | 11 | 108164073 | 108164218 |
| ATM_EX31_4MPLXID199 | CATGTTGTTTTTAAGGATTTGCGTATTAC | CAGATTTTTGAAAAGTACTACTATGTTCTCTAATA | 11 | 108164130 | 108164269 |
| ATM_EX32_1MPLXID200 | TTCACAGGCTTAACCAATACGT | CCAGTTGTCTTCGAAGATCCTT | 11 | 108165602 | 108165741 |
| ATM_EX32_2MPLXID201 | TATGATGCACTTCCATTGACAAGA | AAGAAACAGGTAGAAATAGCCCAT | 11 | 108165684 | 108165828 |
| ATM_EX33_1MPLXID202 | TTAATATATATGCAATTATAAACAAAAGTGTTGTC | GCCATCTTGGATAACTGCAAC | 11 | 108167938 | 108168077 |
| ATM_EX33_2MPLXID203 | GGATTATGGTGAAACTAGTTGTCAATTT | TAATCTGTCCTATATGTGATCCGC | 11 | 108168029 | 108168173 |
| ATM_EX34_1MPLXID204 | GAAGTACAGAAAAACAGCATTATAGTTTTG | TATAGGACCCACTTCTCCCAA | 11 | 108170339 | 108170478 |
| ATM_EX34_2MPLXID205 | AAATAGAATTTCTATATGTAGAGGCTGTTG | GGTCCACTGAAGTTCTTTATCTTCA | 11 | 108170420 | 108170568 |
| ATM_EX34_3MPLXID206 | GCATCTTATACCAAGGCCCTT | TCTCCATGAATGTCATATTGAGATTACA | 11 | 108170515 | 108170664 |
| ATM_EX35_1MPLXID207 | TAACTGGTGTACTTGATAGGCATTT | GGATCTGTTGTCATCTTATAAATCTCCC | 11 | 108172334 | 108172473 |
| ATM_EX35_2MPLXID208 | TAGCCACAAAGACTGGACATAG | CAGAACTGTTTTAGATATGCTGGGT | 11 | 108172419 | 108172570 |
| ATM_EX36_1MPLXID209 | AACTGTATTTAGCTTTATTCAGAAAGATTTGT | TTTTCTTTGTCAAATCTGGGTACTTC | 11 | 108173472 | 108173611 |
| ATM_EX36_2MPLXID210 | TTTACATTTTCTAATCCCTTTCTTTCTAGTTT | GCACAAGTCAGTGTCTTTATCCA | 11 | 108173551 | 108173695 |
| ATM_EX36_3MPLXID211 | TATAAATCTGTGGATTCCTCTAAGTGAAA | TCAGACTAATTAATCTTCTTACTTCACACA | 11 | 108173633 | 108173778 |
| ATM_EX36_4MPLXID212 | TTTTTGGACAGTGGAGGCA | GATGATGTGCAGTATCACAGCA | 11 | 108173697 | 108173836 |
| ATM_EX37_1MPLXID213 | GCATGAAAATTTTAAGTAAAATGTATTAATTTTAC | TTGGAGTAAAATATCATGAATCAAGTATGG | 11 | 108175322 | 108175461 |
| ATM_EX37_2MPLXID214 | CTAGGTGAAAACTGACTTTTGTCAG | CTCGTTTGCGAGAAGTGTCG | 11 | 108175398 | 108175547 |
| ATM_EX37_3MPLXID215 | GTTCAGGGATTTTTCACCAGC | GTGGGATTCCATCTTAAATCCATCT | 11 | 108175501 | 108175654 |
| ATM_EX38_1MPLXID216 | CAAGAATGCCTGGGACTGA | TCCTAAACGTAAGAAGCAACACT | 11 | 108178582 | 108178745 |
| ATM_EX39_1MPLXID217 | TTCTGTTAAGCAGTCACTACCATT | GCCAGAAAGCATCATTAAAAATTGTTC | 11 | 108180785 | 108180924 |
| ATM_EX39_2MPLXID218 | ATATCTCATTTTTCTTTAGACCTTCTTCAG | TCTGCATAGATTTCTGCATAGAGTAAA | 11 | 108180868 | 108181012 |
| ATM_EX39_3MPLXID219 | GTAGCTCAGTCTTGTGCTGC | CCCTTATTGAGACAATGCCAAC | 11 | 108180954 | 108181101 |
| ATM_EX40_1MPLXID220 | TTTTGTTTGCCACCTTCATTAGTT | TTCTGGCTTCCTTCTTCAAATG | 11 | 108183028 | 108183167 |
| ATM_EX40_2MPLXID221 | GAGGTGTTCTTGTGACAAACAG | TGAGATAAATACTGTCATAAATAATAGAGCCT | 11 | 108183116 | 108183268 |
| ATM_EX41_1MPLXID222 | TTGGGAGTTACATATTGGTAATGATACA | TCTGGCTCCCCTATACTTCTG | 11 | 108186451 | 108186590 |
| ATM_EX41_2MPLXID223 | TCCATGTTTTCAGGATCTTCTCTTAG | CCTTCATAAACAGAATTACTATACCGTTG | 11 | 108186537 | 108186687 |
| ATM_EX42_1MPLXID224 | AACAACGGTATAGTAATTCTGTTTATGAAG | CGAGGTCATATGTTACTAGGGC | 11 | 108186657 | 108186796 |
| ATM_EX42_2MPLXID225 | GAACATATGAACACGAAGCAATGT | CAGCACTACACTAGTGATGGC | 11 | 108186743 | 108186887 |
| ATM_EX43_1MPLXID226 | TCTGTTGATATCTTTGATTACTTAACTTAAAAACA | CTAGTTCAGGACACCAGTCTTTATT | 11 | 108188048 | 108188187 |
| ATM_EX43_2MPLXID227 | CCATATTCTTTCCGTCTATTTAAAAGGATT | ATCAAGTCAAATTTCTTACCTGACG | 11 | 108188123 | 108188267 |
| ATM_EX43_3MPLXID228 | AAGAACTTCATTACCAAGCAGCA | TATTCATAGAAGAGAAAAACAGTTGTTGTT | 11 | 108188188 | 108188327 |
| ATM_EX44_1MPLXID229 | TTTGTCCTTTGGTGAAGCTATTTATAC | GAGATTGTAGAGCATTGTACAATGATTC | 11 | 108190597 | 108190736 |
| ATM_EX44_2MPLXID230 | GAAGTAGAAGGAACCAGTTACCAT | GAAGCCATGACATTAAATGTTAAAATACAG | 11 | 108190685 | 108190837 |
| ATM_EX45_1MPLXID231 | AACAACAAATTTAAACATTTATTTCCCTGAAA | TGTGGGATAGAGCGAATACAC | 11 | 108191952 | 108192091 |
| ATM_EX45_2MPLXID232 | AAGAGATGTGTAAGCGCAGC | GCTGCACTTTAGGATAACAAAGTC | 11 | 108192042 | 108192186 |
| ATM_EX46_1MPLXID233 | ATTTCTCTTGCTTACATGAACTCTATGT | CATGATAGGCTCCTGAAAACTAAAATC | 11 | 108195997 | 108196136 |
| ATM_EX46_2MPLXID234 | AAACACTCCCAGCTTCTCAAG | TCTACAAGGTGTTTGGTGAGAAT | 11 | 108196083 | 108196234 |
| ATM_EX46_3MPLXID235 | GGAAAAGGAAATGGACAACTCAC | AAAGTCAAGAGGTAAGATGACATAGTT | 11 | 108196169 | 108196313 |
| ATM_EX47_1MPLXID236 | gttaagtcctcaatgaatggtagttG | ACTGTTTAATTTGAAATATTGCCCTTTCA | 11 | 108196679 | 108196818 |
| ATM_EX47_2MPLXID237 | ATTTATTCCCATATGTCATTTTCATTTCAG | TACTCAGGGCAAGACTCTGC | 11 | 108196755 | 108196908 |
| ATM_EX47_3MPLXID238 | CTGGAAGAAGCACAAGTATTCTG | TTTAAATAACAGTAAAACACTAATCCAGCC | 11 | 108196854 | 108196998 |
| ATM_EX48_1MPLXID239 | TTGGGTACAGTCATGGTAATGC | CAGGATTTTCTAAGCACGTTTCTG | 11 | 108198317 | 108198456 |
| ATM_EX48_2MPLXID240 | CACAGAATGTCTGAGGGTTTGT | AAGATGAAGCATATTCATGCTAAGTAAC | 11 | 108198398 | 108198542 |
| ATM_EX49_1MPLXID241 | GTGATTCTTTAGATGTATTTAGTATTTGTAAATAT | GAGAGAAATGCCTTCATTTTTCCAT | 11 | 108199685 | 108199824 |
| ATM_EX49_2MPLXID242 | GTTGCTGGAAATTATGATGGAGAAA | CAGGAGAGCTTGCTTGTTTTC | 11 | 108199757 | 108199909 |
| ATM_EX49_3MPLXID243 | ACCAAAGAATTGAAAACTACATGAAATCA | GGTGAACATAAAATTGTCACTTGTAGAA | 11 | 108199851 | 108200003 |
| ATM_EX50_1MPLXID244 | ATTTTGTAGTTCTGTTAAAGTTCATGGC | AGAAGCGTTTACGATCCTCTTT | 11 | 108200881 | 108201020 |
| ATM_EX50_2MPLXID245 | GAGTTGGATGAATTAGCCCTGC | TTTCAAGCCAGAGGGAACAA | 11 | 108200969 | 108201113 |
| ATM_EX50_3MPLXID246 | ACATGATATGTGGGTATTCCGAC | ACGATTCCTGACATCAAGGG | 11 | 108201070 | 108201224 |
| ATM_EX51_1MPLXID247 | GCTTAGATGTGAGAATATTTGAAATACCTT | TCTTGGTCCCCATTCTAGCA | 11 | 108202107 | 108202246 |
| ATM_EX51_2MPLXID248 | GACGGAATGAAGATTCCAACATATAAATT | AATTATTGTGGTTTGATTTTCAGGTTTACT | 11 | 108202174 | 108202317 |
| ATM_EX51_3MPLXID249 | TGATGGGAGGCCTAGGATT | GTATTTCCATTTCTTAGAGGGAATGG | 11 | 108202247 | 108202386 |
| ATM_EX52_1MPLXID250 | CCCACTGCAGTATCTAGACAG | GGGGTGATCCATTGAAATTCTAGA | 11 | 108202496 | 108202635 |
| ATM_EX52_2MPLXID251 | TGCATAAATCTAATAGTTCTTTTCTTACAGC | TGCTTCTTCTGGCTACCTCT | 11 | 108202576 | 108202720 |
| ATM_EX52_3MPLXID252 | CAAATGCAAACAGAGATGAATTTCTG | GACTGAATATCACACTTCTAAAAGGTAC | 11 | 108202667 | 108202811 |
| ATM_EX53_1MPLXID253 | GTTTAAATGTTGGGTAGTTCCTTATGTAAT | CATCACAAAGTGCCTCAACAC | 11 | 108203437 | 108203576 |
| ATM_EX53_2MPLXID254 | CTATCAGAAGTAGGAGACCTCAGA | CACAGCAAGAAAGTAACGTTTCTT | 11 | 108203523 | 108203668 |
| ATM_EX54_1MPLXID255 | GCCAGTGGTATCTGCTGAC | CTTCTAAATTCTTAAGTTTAGTAATTGGCTGG | 11 | 108204527 | 108204666 |
| ATM_EX54_2MPLXID256 | CCTCAGTTTGTCACTAAAATCTCTTC | TCAAGAGTTAATTGCAAATTACCTTAATTTC | 11 | 108204573 | 108204717 |
| ATM_EX54_3MPLXID257 | ATACAGAAGGCATAAATATTCCAGCA | AAAATACTATTTCAAATGGTTCAATCTAATAAAAA | 11 | 108204607 | 108204771 |
| ATM_EX55_1MPLXID258 | TAAGTGCAAATAGTGTATCTGACCTATTAT | GGTAAATTTACACCTCCTGCTAAG | 11 | 108205642 | 108205781 |
| ATM_EX55_2MPLXID259 | ATCTGGTGACTATACAGTCATTTAAAGC | TAAGGGCTAAGCCAGAGAAG | 11 | 108205721 | 108205867 |
| ATM_EX56_1MPLXID260 | GACTCTGTGTTTTTATAATAAAATAAACTGTACTT | CGTGTTTCTCTGCAGTAATGTATTAC | 11 | 108206510 | 108206649 |
| ATM_EX56_2MPLXID261 | GCAACAGGTCTTCCAGATGT | CCACTTCACCCAACCAAATG | 11 | 108206604 | 108206756 |
| ATM_EX57_1MPLXID262 | AAATGCTCTTTAATGGCCTTTTAAAATTAAA | TCTTCATTGTTAACAAGAAATTCACCAA | 11 | 108213895 | 108214034 |
| ATM_EX57_2MPLXID263 | CTTGAATGGTGCACAGGAAC | ACCTTTAATTTTGGGTGTCACTCA | 11 | 108213979 | 108214123 |
| ATM_EX57_3MPLXID264 | TGGTGCTCATAAAAGATACAGGC | TAATATCTGACAGCTGTCAGCTTTAA | 11 | 108214035 | 108214174 |
| ATM_EX58_1MPLXID265 | GTTTAATTGAACACAATATTGAAAAATAATTATAT | ATGCAGAAGTAACGGAAAACTG | 11 | 108216418 | 108216558 |
| ATM_EX58_2MPLXID266 | GTCTTCATGGATGTTTGCCAAAA | GTACAAGAAGATTACCAATAGAAGAAGTAG | 11 | 108216506 | 108216650 |
| ATM_EX58_3MPLXID267 | GGAAAAATTCTTGGATCCAGCTATTT | AAACAAAAATAAAACCTGCCAAACAAC | 11 | 108216559 | 108216698 |
| ATM_EX59_1MPLXID268 | TACCAAGTCAGTGGTCTTAATTGAA | TCAAGATATTCTGTACATGTCTATCACC | 11 | 108217914 | 108218053 |
| ATM_EX59_2MPLXID269 | CTCCAGTTGGTTACATACTTGGA | CAACATTCCATGATGACCAAATATTTACT | 11 | 108218000 | 108218154 |
| ATM_EX60_1MPLXID270 | ACTGGAAAGAAAGTAAATTAGCTGTC | GCCATCCACAATATCTCTGGT | 11 | 108224433 | 108224572 |
| ATM_EX60_2MPLXID271 | CCTACTCCTGAGACAGTTCCT | CCAGCCCATGTAATTTTGACATC | 11 | 108224522 | 108224666 |
| ATM_EX61_1MPLXID272 | TGTGTAACAAAATCCGTATTTATAATGTGT | GATACACAGTCTACCTGGTAAGAAAA | 11 | 108225498 | 108225658 |
| ATM_EX62_1MPLXID273 | TTAAAATGTACATTGTTCTTTTAATACATATGTTC | GGTGAAGCTCAGTTTCATCTTC | 11 | 108235763 | 108235902 |
| ATM_EX62_2MPLXID274 | TTGAAAGCTTTGTATTTACAGCAGAG | GCCTTGGGAATAAGAAAATCTGAAAA | 11 | 108235851 | 108236005 |
| ATM_EX63_1MPLXID275 | GAAGGTCCTGTTGTCAGTTTTTC | GTCTCATTAAGACACGTTCAGCT | 11 | 108235962 | 108236101 |
| ATM_EX63_2MPLXID276 | GTCCTTAGTGATATTGACCAGAGTTT | GGTCTATGGCCTGCTGTATG | 11 | 108236044 | 108236188 |
| ATM_EX63_3MPLXID277 | TTGGTGGACAAGTGAATTTGC | AAGGCTAAAATATAATTTCTAAAGGCTGAAT | 11 | 108236147 | 108236291 |
| CIITA_EX1_1MPLXID1031 | CCAGACTCCGGGAGCT | AGGACCAGCTGAGACTGC | 16 | 10971147 | 10971291 |
| CIITA_EX2_1MPLXID1060 | CTTTCCAACACCCTGTGAGG | ATAGAAGTGGTAGAGGCACA | 16 | 10989093 | 10989239 |
| CIITA_EX2_2MPLXID1061 | GGAGCTTCTTAACAGCGATGC | TCTCTACAGCAAGCTGAGGG | 16 | 10989191 | 10989345 |
| CIITA_EX3_1MPLXID1062 | TCCTTCTTCATCCAAGGGACT | TCCTTTTCTGGGCTCAGG | 16 | 10989494 | 10989650 |
| CIITA_EX4_1MPLXID1063 | cttgttgattgactgCGCTTT | GAACAAGGCAGGAAATGCAG | 16 | 10992491 | 10992645 |
| CIITA_EX5_1MPLXID1064 | TTGATTGTGTGAGTTGGTCTCT | AAGCAAGGCTAGGTTGGATC | 16 | 10992743 | 10992897 |
| CIITA_EX6_1MPLXID1065 | CCTTCTGGCTTGGGACATC | CAAGGCATCCACCCTTCC | 16 | 10995314 | 10995460 |
| CIITA_EX7_1MPLXID1066 | CAAGCACCCAGTCTCTAACA | GCTCCTGGTTGAACAGCG | 16 | 10995857 | 10995996 |
| CIITA_EX7_2MPLXID1067 | GTGAGCGACTGCTCCAC | CAAGAGGGAGAAAGCCCAC | 16 | 10995939 | 10996083 |
| CIITA_EX8_1MPLXID1068 | TATGCAAGATCCCACCTCAC | AGATTTGCCAGAGCCCATG | 16 | 10996477 | 10996616 |
| CIITA_EX8_2MPLXID1069 | GACCCATCCAGTTTGTCCC | CAGGGAGCAGTCAGGTAG | 16 | 10996560 | 10996714 |
| CIITA_EX9_1MPLXID1070 | ATCAATACCTGGTTATTCTCACACC | AGCGAAGGGGCTGGT | 16 | 10997543 | 10997688 |
| CIITA_EX9_2MPLXID1071 | CCAACATCTCCAGACCGG | CTGCTTCTAGTACCTCTCACAG | 16 | 10997647 | 10997791 |
| CIITA_EX10_1MPLXID1032 | CTAAATCTGGCACCTGCTTCT | ATATGGGCTTCCATCTCCAC | 16 | 10998570 | 10998721 |
| CIITA_EX11_1MPLXID1033 | TGTAAATGATGGTGGCAGTGC | GGCACCATACGTGTCCTG | 16 | 11000263 | 11000402 |
| CIITA_EX11_2MPLXID1034 | GCAGTTCTACCGCTCACTG | TTCTGCCCAGTCCGGG | 16 | 11000366 | 11000516 |
| CIITA_EX11_3MPLXID1035 | GAGCGGGAACTGGCC | CAATAGCTCTTGCCCTGACC | 16 | 11000484 | 11000635 |
| CIITA_EX11_4MPLXID1036 | TGAGACACGAGTGATTGCTG | CGGACGGTTCAAGCAATG | 16 | 11000582 | 11000726 |
| CIITA_EX11_5MPLXID1037 | ACGACTTTGTCTTCTCTGTCC | CGGTCAGGTCTCTTCAAGATG | 16 | 11000683 | 11000827 |
| CIITA_EX11_6MPLXID1038 | CTGCAGGATCTGCTCTTCTC | TGTGCAGGAAGCCATCTTG | 16 | 11000742 | 11000886 |
| CIITA_EX11_7MPLXID1039 | GTTCTGCTCATCCTAGACG | GAGGAGGGTGCAACCTC | 16 | 11000829 | 11000981 |
| CIITA_EX11_8MPLXID1040 | GCCTTTTCCAGAAGAAGCTG | AAGTAGCGCATCACGTATGC | 16 | 11000941 | 11001094 |
| CIITA_EX11_9MPLXID1041 | TATTTGAGCTGTCCGGCTTC | CACAAAGTAGGGCTGTGGC | 16 | 11001037 | 11001190 |
| CIITA_EX11_10MPLXID1042 | GCCACTTCTTCTCAGTCACA | CTGTCGAGGGCTGCAC | 16 | 11001152 | 11001304 |
| CIITA_EX11_11MPLXID1043 | GGGACTCTATGTCGGCCT | CCTCACGTCTGCGGATG | 16 | 11001263 | 11001407 |
| CIITA_EX11_12MPLXID1044 | GACATCAAAGTACCCTACAGGAG | CCCAGGAAGCATTGCAGG | 16 | 11001358 | 11001502 |
| CIITA_EX11_13MPLXID1045 | GCCGCAGAGTCCGAG | TGTCATAGGGCCTCTTCTTC | 16 | 11001450 | 11001594 |
| CIITA_EX11_14MPLXID1046 | AATCAAGGACAAGGAGCTCC | CCCGAGTAGGGCTCCC | 16 | 11001530 | 11001674 |
| CIITA_EX11_15MPLXID1047 | GGCGTGCCACGCTTT | CGCGCCCGCAGTGTC | 16 | 11001606 | 11001760 |
| CIITA_EX11_16MPLXID1048 | GAGGTACCTGAAGCGGC | GTGCCCAGAAAAGAGAGGC | 16 | 11001719 | 11001862 |
| CIITA_EX11_17MPLXID1049 | CACGTGGTACAGGAGCTC | CTCCCCAATCCAGAGGG | 16 | 11001819 | 11001973 |
| CIITA_EX11_18MPLXID1050 | CACGCCTCCTGATGCAC | GTCTCTTCCCCAAGCCC | 16 | 11001869 | 11002033 |
| CIITA_EX12_1MPLXID1051 | TAAGGTCTAGCCTGGTCACC | GTGAACTTCTCCTCTGCTGC | 16 | 11002836 | 11002975 |
| CIITA_EX12_2MPLXID1052 | GGGAGACCAAGCTACTTCAG | ATGGCGTGAACCACCTC | 16 | 11002936 | 11003081 |
| CIITA_EX13_1MPLXID1053 | GTAAGGGCTCAGTGACAGC | CCCACAAGACAATCCACCC | 16 | 11003989 | 11004145 |
| CIITA_EX14_1MPLXID1054 | TGGAGGTCTTACCCTTGCT | ACCCAGATGTTGAGAGGAAG | 16 | 11009395 | 11009549 |
| CIITA_EX15_1MPLXID1055 | GACGCTAGCTGATGGCC | ATTCAGCAGGAAGGGCAG | 16 | 11010186 | 11010349 |
| CIITA_EX16_1MPLXID1056 | CAAGTTTGGTCCTGAGCCC | CCTGACCGGTATCCGGG | 16 | 11012255 | 11012411 |
| CIITA_EX17_1MPLXID1057 | ATGCACAGGCCTCCAATC | CCACCGCCCAGACTC | 16 | 11015979 | 11016132 |
| CIITA_EX18_1MPLXID1058 | CTGGGGAGTCCCAAGGG | ACCCACCAGGGTTGC | 16 | 11016217 | 11016372 |
| CIITA_EX19_1MPLXID1059 | TCTAACCTRGCTCTGAGTCCC | CAAGCCCACCCTGGTTAC | 16 | 11017050 | 11017200 |
| SH2B3_EX2_1MPLXID791 | CTTTCAGCCCGGCCG | CTACGGCGTGCAACTCA | 12 | 111855916 | 111856055 |
| SH2B3_EX2_2MPLXID792 | ggcTGGAGCGAGTTCTG | AGCGCTGGAAGAGGTC | 12 | 111856022 | 111856169 |
| SH2B3_EX2_3MPLXID793 | GAGCATCCGCAGCACG | TCGGCCTTGGCTGGG | 12 | 111856100 | 111856251 |
| SH2B3_EX2_4MPLXID794 | CGACTACCGGGACACAG | AAAGTGCTGGAAGGAGCAG | 12 | 111856210 | 111856363 |
| SH2B3_EX2_5MPLXID795 | CTGCCCAAGGCCCGC | GCAGCGGTGTGGGCC | 12 | 111856292 | 111856431 |
| SH2B3_EX2_6MPLXID796 | GCCACATCTTCCGCCG | CTCGGGTGGCGGCTC | 12 | 111856377 | 111856531 |
| SH2B3_EX2_7MPLXID797 | CCCGGAGAGGCTGCT | CGCTGTCCATGGAGGC | 12 | 111856445 | 111856589 |
| SH2B3_EX2_8MPLXID798 | GGTGCTGCGCTACAGC | GGGCTTACTTACCTTGGGTG | 12 | 111856546 | 111856693 |
| SH2B3_EX2_9MPLXID799 | GGGCACGCTGGCAGC | GGCGAAGGGCGCAGT | 12 | 111856590 | 111856730 |
| SH2B3_EX3_1MPLXID800 | GCCTTGAGTACCCCAACTT | CCAGGGTGTGAAAAGCCT | 12 | 111884528 | 111884686 |
| SH2B3_EX4_1MPLXID801 | ATACAGCAGACCCAACCC | CAGGGACGAGGGCCC | 12 | 111884707 | 111884862 |
| SH2B3_EX5_1MPLXID802 | AGATCCTTAACCTCAGCCTCT | AGAAGACAATGGCCTAGCTATTG | 12 | 111884898 | 111885056 |
| SH2B3_EX6_1MPLXID803 | GTAAACCAATAGCTAGGCCATTG | TTCTGGCAGGCCGGG | 12 | 111885028 | 111885173 |
| SH2B3_EX6_2MPLXID804 | CTAACAGGTGCTTCTCCTGG | AACACTCCATGAGCATCAGG | 12 | 111885127 | 111885278 |
| SH2B3_EX6_3MPLXID805 | CCCATCTCCAGAGTGAAAGC | CACCCCATACCTTGGCTATC | 12 | 111885214 | 111885358 |
| SH2B3_EX6_4MPLXID806 | AGCTCAGCTGGTTCAGC | CTGCCCCACCCCACC | 12 | 111885234 | 111885379 |
| SH2B3_EX7_1MPLXID807 | GGGACAGCCCGAGCC | ACTCGAGTGGGATGGGC | 12 | 111885424 | 111885571 |
| SH2B3_EX7_2MPLXID808 | ACCACTTCCAGCGCTC | TACCCTCTACCCAGTGTCAG | 12 | 111885539 | 111885685 |
| SH2B3_EX8_1MPLXID809 | TTCTGTGTCCTGTCAGCAC | CTGAACCCCAGTGAGGAAG | 12 | 111885722 | 111885861 |
| SH2B3_EX8_2MPLXID810 | CTTCCTCACTGGGATTCAGAG | CCAGGTGGAAGATCTGCTC | 12 | 111885819 | 111885963 |
| SH2B3_EX8_3MPLXID811 | CCCAGGGCGATCCTCA | CTCCGGGAGGATGAGTCC | 12 | 111885923 | 111886066 |
| SH2B3_EX8_4MPLXID812 | CCGGGACTCGGACTACG | TCACAAGTTCACACTTCTGCC | 12 | 111886028 | 111886172 |
| SOCS1_EX2_1MPLXID813 | AATAACAAAATAACACGGCATCCC | TCGTGGCCACCGTGG | 16 | 11348643 | 11348791 |
| SOCS1_EX2_2MPLXID814 | GAGCCAGGTTCTCGCG | CTTCGACTGCCTCTTCGAG | 16 | 11348759 | 11348907 |
| SOCS1_EX2_3MPLXID815 | CGCCGCCACGTAGTG | GAACTGCTTTTTCGCCCTTAG | 16 | 11348865 | 11349009 |
| SOCS1_EX2_4MPLXID816 | CTCGTGGGTCCCGAGG | CCTGCGGATTCTACTGGG | 16 | 11348962 | 11349109 |
| SOCS1_EX2_5MPLXID817 | GTCGCGCACCAGGAA | TTCCGCACATTCCGTTCG | 16 | 11349021 | 11349173 |
| SOCS1_EX2_6MPLXID818 | GTGATGCGCCGGTAATCG | GACGGCGGCCAGAAC | 16 | 11349133 | 11349277 |
| SOCS1_EX2_7MPLXID819 | GTGCGTGTcgccggg | CACAACCAGGTGGCAGC | 16 | 11349174 | 11349326 |
| SOCS1_EX2_8MPLXID820 | GGGGCTCTGCTGCTG | CGCCCCAGCTCACCT | 16 | 11349278 | 11349417 |
| EPOR_EX8_1MPLXID0 | AAGTCTTGAGTCTGCACTGGTTC | CTTATCCGATGGCCCCTACTCC | 19 | 11488595 | 11488750 |
| EPOR_EX8_2MPLXID1 | GGGATAAGGCTGTTCTCATAAGG | CCACCCCACCTAAAGTACC | 19 | 11488703 | 11488842 |
| EPOR_EX8_3MPLXID2 | AGTTGAGATGCCAGAGTCAGATA | CTGCTGCCAGCTTTGAGTA | 19 | 11488789 | 11488928 |
| EPOR_EX8_4MPLXID3 | TACAGGTACTTTAGGTGGGGTG | ATCCTCCTGCTCATCTGCTTTG | 19 | 11488820 | 11488978 |
| EPOR_EX8_5MPLXID4 | AAAGCTGGCAGCAGAGG | GGTTGCTGCCCCGGAA | 19 | 11488915 | 11489066 |
| EPOR_EX8_6MPLXID5 | GATGCTTCTGAGCCTTCATCC | CCAGTGGGCAGTGAGCAT | 19 | 11488976 | 11489115 |
| EPOR_EX8_7MPLXID6 | GGGCAGCAACCATTTGTCC | GACCCACCTGCTTCCCTG | 19 | 11489056 | 11489211 |
| EPOR_EX8_8MPLXID7 | GGCCCTCATCATCTGTCCC | ATTTCTTCAGCTGTGGCTGTACC | 19 | 11489127 | 11489281 |
| EPOR_EX8_9MPLXID8 | GGGTCCTCCGTGAAGGG | CTCAGTGCCTGGGCTTC | 19 | 11489207 | 11489346 |
| NRAS_EX3_1MPLXID667 | CTTCCCTAGTGTGGTAACCTC | CTCTGTGTATTTGCCATCAATAATAGC | 1 | 115256337 | 115256476 |
| NRAS_EX3_2MPLXID668 | TAGTACCTGTAGAGGTTAATATCCGC | GTTTGTTGGACATACTGGATACAG | 1 | 115256415 | 115256559 |
| NRAS_EX3_3MPLXID669 | GTCTCTCATGGCACTGTACTC | AAAATTGAACTTCCCTCCCTCC | 1 | 115256504 | 115256648 |
| NRAS_EX2_1MPLXID665 | TCCTCTTATTCCTTTAATACAGAATATGGG | TCCAGCTAATCCAGAACCAC | 1 | 115258581 | 115258720 |
| NRAS_EX2_2MPLXID666 | CACCTCTATGGTGGGATCATATTC | CTGGTTTCCAACAGGTTCTTG | 1 | 115258668 | 115258812 |
| CD58_EX6_1MPLXID1030 | AAATAATTTAGTTATGCTGTTGTCTTCATCT | AGGGCTATTCACTTTAGAATAGGAG | 1 | 117057393 | 117057543 |
| CD58_EX5_1MPLXID1029 | CTTCCCAAGTAATGGGCATCT | TTCAGTTCTTTAATTGAGAAGACAATTTCT | 1 | 117061778 | 117061929 |
| CD58_EX4_1MPLXID1028 | CAATGCAAGTTTTCAAACTATTTTGTTTTAAAA | CAGTTCAACTAGTTGAATTTCTTTTCTTTTC | 1 | 117064484 | 117064646 |
| CD58_EX3_1MPLXID1024 | TGTAACTTGTTTTACATTTCAAAATTGTGAA | ATACAACATCATCAATCATTTTGACAACC | 1 | 117078492 | 117078631 |
| CD58_EX3_2MPLXID1025 | TAGTTATTTACTCACCGCTGCTT | GGAGCAATGTAAACGTAACTCAAC | 1 | 117078572 | 117078726 |
| CD58_EX3_3MPLXID1026 | TTGTGGAAGATCATTTTCCATCTTAAAAT | TGGAAGCATTGAAGTCCAATG | 1 | 117078666 | 117078810 |
| CD58_EX3_4MPLXID1027 | CGATGGCTGTTGTAATGCTCT | TGCAGATGATAAGTCTAAATATTACTATTTTCTG | 1 | 117078760 | 117078911 |
| CD58_EX2_1MPLXID1020 | TTGTGCTAGTCTCTGAAAACAGA | GATGAGTATGAAATGGAATCGCC | 1 | 117086857 | 117086996 |
| CD58_EX2_2MPLXID1021 | CACATAAAGAAAGAACTTCATGGTATCAG | TGAATTCAGAGCTTTCTCATCTTTTAAAA | 1 | 117086937 | 117087090 |
| CD58_EX2_3MPLXID1022 | GCTACCTGACACAGTGTCTAAATAA | GGAATGTAACTTTCCATGTACCAAG | 1 | 117087030 | 117087181 |
| CD58_EX2_4MPLXID1023 | TTTCCATAGGACCTCTTTTAAAGGC | CTGCCACCAAAGTCATGTTG | 1 | 117087126 | 117087278 |
| CD58_EX1_1MPLXID1019 | CTGCCCAGTAcccgc | ACTCGCGCAGAGGCC | 1 | 117113473 | 117113622 |
| CBL_EX1_1MPLXID318 | TCGAGCCGAGCCGGC | GCGGCTGGAAGGCGT | 11 | 119077081 | 119077233 |
| CBL_EX1_2MPLXID319 | CCTGATTGGGCTCATGAAGG | ACAGCGGGCGCTCAG | 11 | 119077199 | 119077349 |
| CBL_EX2_1MPLXID320 | TTAAAATTCTCCAAGTAATAGCCCTTCT | AGATAGTACGGAGATGCTGGT | 11 | 119103114 | 119103257 |
| CBL_EX2_2MPLXID321 | ACCTTATATCTTAGACCTGCTACCA | GAGGCTTATGGTTTGCTTAGTTTTC | 11 | 119103205 | 119103358 |
| CBL_EX2_3MPLXID322 | AGACACTTGGAGAAAATGAGTATTTTAG | TGTGTAGTATTTCTCCATTACCTAGG | 11 | 119103282 | 119103426 |
| CBL_EX2_4MPLXID323 | TTCAAGGAGGGAAAAGAAAGAATGT | CATAAAATTAAAACCAACTCAACTACTTCTATG | 11 | 119103359 | 119103498 |
| CBL_EX3_1MPLXID324 | GTGCatttaaaataaaaataattttatGTGTTTAA | GTCTCCCTGAAAGAGTCC | 11 | 119142386 | 119142532 |
| CBL_EX3_2MPLXID325 | GGCAGAACTAAAAGGAATCTTTCC | TCGTAAGCTTTAGTTTTTCATAGTTCAAAT | 11 | 119142487 | 119142640 |
| CBL_EX4_1MPLXID326 | GAATTATCTCTGTTATTTCACTTTATGCCT | GTGGATTTCAGAGCCATGGC | 11 | 119144528 | 119144667 |
| CBL_EX4_2MPLXID327 | GAAGTGCATCCCATCAGTTCT | AGCAGTAACCAGTTTAGTCAACTT | 11 | 119144618 | 119144772 |
| CBL_EX5_1MPLXID328 | ATTGCCCTCTGAGTTGGTTG | GCTTTCACTTCGTCATACGTCA | 11 | 119145479 | 119145630 |
| CBL_EX5_2MPLXID329 | CTGTAACTCATCCTGGCTACATG | GCAGAAGTCTCAAGATAACGAAATTC | 11 | 119145579 | 119145723 |
| CBL_EX6_1MPLXID330 | AATACCAGCATAATCTACTAAAGCTTCT | AGGTTTATTGTGAGGGATTGTCT | 11 | 119146661 | 119146806 |
| CBL_EX6_2MPLXID331 | GTATGTTACTGCTGATGGGAACA | AATGATGAGGTTGGACAGCC | 11 | 119146755 | 119146909 |
| CBL_EX7_1MPLXID332 | GCTTAAATAAAACCCAGGGTTGG | TTGTGTCCAGTGATATGGTTAT | 11 | 119148431 | 119148595 |
| CBL_EX8_1MPLXID333 | TGCAGTTATTTATTCAACTAATAGTCTTTTAATTT | CAGGGCTCAATCTTTACATCCT | 11 | 119148828 | 119148967 |
| CBL_EX8_2MPLXID334 | ACATTCCAACTATGTAAAATATGTGCTG | CGAATTTTCCAAGGTTATTACATAGCT | 11 | 119148909 | 119149061 |
| CBL_EX9_1MPLXID335 | TAATATTTTAAGTATTTTCAGATGCATCTGTTACT | CTCTAGGATCAAACGGATCTACC | 11 | 119149165 | 119149304 |
| CBL_EX9_2MPLXID336 | CCGATGTGAAATTAAAGGTACTGAAC | GAGAGTATCATCAGCTCGTTCATC | 11 | 119149249 | 119149393 |
| CBL_EX9_3MPLXID337 | GCTCCCTCCCCAAATTATGAT | TTTACGGCTTTAGAAGACAACTCA | 11 | 119149340 | 119149484 |
| CBL_EX10_1MPLXID338 | GATGCCATTTCCCCAAACG | CTCGCTGCGGCAGAA | 11 | 119155627 | 119155766 |
| CBL_EX10_2MPLXID339 | GCCACCACGACTTGACC | CAGGGTGAAAGCAAATCAGTAAAA | 11 | 119155735 | 119155889 |
| CBL_EX11_1MPLXID340 | AGTGGGTTTTTACTGATTTGCTTTC | CAACAGAATATGGCCGGTCT | 11 | 119155859 | 119155998 |
| CBL_EX11_2MPLXID341 | TTCGAGATCTTCCACCACCA | CATGAGTCTCCAAGGCGG | 11 | 119155951 | 119156104 |
| CBL_EX11_3MPLXID342 | CTGTCCCTCCAGAGACAAAC | TGAGTGCCGGTTGGTTAATT | 11 | 119156048 | 119156192 |
| CBL_EX11_4MPLXID343 | AAGTTCCAGTGATCCCTGGA | ATTAAGACTCACCATGGAGGTATC | 11 | 119156144 | 119156288 |
| CBL_EX11_5MPLXID344 | TTCCATTTTCATTGCCCTCAC | GTGCCTGGCCCACAC | 11 | 119156194 | 119156333 |
| CBL_EX12_1MPLXID345 | TTGTATCTGTTATTGTTATTTTGAAAAGGTTC | GGGTGGTCACACTCTGG | 11 | 119158466 | 119158605 |
| CBL_EX12_2MPLXID346 | CAGAGTATGAATAGCAGCCCAT | TACAAGTAACAACTCCACTGTACAAA | 11 | 119158559 | 119158703 |
| CBL_EX13_1MPLXID347 | GCCATTTATTTTATACTTCAAAGTTTACTGG | TCATGTACTCTGTGTCCTCTTCA | 11 | 119167556 | 119167695 |
| CBL_EX13_2MPLXID348 | TCCTGTGCCAAAACTGCC | ACATAAAGGTATAGGGCCCAAAA | 11 | 119167634 | 119167786 |
| CBL_EX14_1MPLXID349 | GATATTGGCAAAACGAGAAGATGAAT | ATTGCTTCATACGTACAGCTATCA | 11 | 119168002 | 119168141 |
| CBL_EX14_2MPLXID350 | TTCAGAGCATGTGATTGCGA | TGACATATCAATGATTTTTAAAGTAGCAGAA | 11 | 119168089 | 119168233 |
| CBL_EX15_1MPLXID351 | TAAAAATGAATGGCTGCCCC | ATTTTCTGACTCCTCGGGAC | 11 | 119168984 | 119169123 |
| CBL_EX15_2MPLXID352 | TTCTCCAGGTGAAGGGAATTTG | AGCTGGCATTAGAGATATCTGAGA | 11 | 119169060 | 119169208 |
| CBL_EX15_3MPLXID353 | CTGGCCCGCCGAACT | TCAATATAAACATACAGGCCACACT | 11 | 119169169 | 119169313 |
| CBL_EX16_1MPLXID354 | GGAAGTCAGTAATATCTTGATATTAGCACA | GTTTTGGAGGCCTCTCGG | 11 | 119170107 | 119170246 |
| CBL_EX16_2MPLXID355 | CTTCTAGATGTCACTGAAGGTTCC | ATCTCACTGGAGAGCTGAGG | 11 | 119170198 | 119170352 |
| CBL_EX16_3MPLXID356 | CAGCAAGGTAGTGGTCCTG | TTGGCCATCTCGATGTTGTT | 11 | 119170291 | 119170436 |
| CBL_EX16_4MPLXID357 | GACATCCAGAAAGCTTTGGTCA | ACTCACTGGTCCTCTAAACCT | 11 | 119170387 | 119170531 |
| NOTCH2_EX34_1MPLXID648 | GCTGGCTCCAGAGATTTCTT | GAGCCACCACACAACAACA | 1 | 120457825 | 120457964 |
| NOTCH2_EX34_2MPLXID649 | TGGACTCTCTCACGCATAAAC | AAGTTCATCACCCCACTCTG | 1 | 120457920 | 120458067 |
| NOTCH2_EX34_3MPLXID650 | TGGTCACATCTGACCAGTCA | ATGCTTCCTCAAATGCTGCT | 1 | 120458024 | 120458170 |
| NOTCH2_EX34_4MPLXID651 | CACTGTGACTGGGTGTTCG | CATGATGCCCCAGCAGG | 1 | 120458129 | 120458277 |
| NOTCH2_EX34_5MPLXID652 | GGAGAATGGTCTGAGCTACC | CAGCCTCAGTCCACCTG | 1 | 120458234 | 120458378 |
| NOTCH2_EX34_6MPLXID653 | CATTTCTGGAATCTGGTACATGGT | TAACCACCCCTCGGGAG | 1 | 120458310 | 120458464 |
| NOTCH2_EX34_7MPLXID654 | GATGAGCTGGAAAGTCACAATG | CCAGTACAATGAGATGTTTGGTATG | 1 | 120458415 | 120458559 |
| NOTCH2_EX34_8MPLXID655 | GGGAGCTATGCCAGGATG | CAGTGCTGGAAGCTTGAGT | 1 | 120458493 | 120458637 |
| NOTCH2_EX34_9MPLXID656 | TTCATCCAATCTGCTGGGAC | CATGAAATGCAGCCTTTGGC | 1 | 120458581 | 120458732 |
| NOTCH2_EX34_10MPLXID657 | GATAGCAACTGGCTCACTGAG | CCTCACCCAACCCTATGTTG | 1 | 120458668 | 120458812 |
| NOTCH2_EX34_11MPLXID658 | AGAAAAAGATAGTGCATGCTGG | AGAATCTCCTCACACGTATGTTTC | 1 | 120458739 | 120458883 |
| NOTCH2_EX34_12MPLXID659 | CCCAGGGGATGTAATCATTGG | CAAAGGATGCCAAGGGTAGT | 1 | 120458823 | 120458977 |
| NOTCH2_EX34_13MPLXID660 | TCTCACTCAGAGACTTCTTCCT | ATCTTTCCTCAGCCTGAAGC | 1 | 120458933 | 120459078 |
| NOTCH2_EX34_14MPLXID661 | TCTAGACTTCTTGCCCATTGG | CATGCACCATGACATTGTGC | 1 | 120459033 | 120459186 |
| NOTCH2_EX34_15MPLXID662 | GGTCACATTGTATTCATCCAGAAG | GAGCTATGAAGCAGCCAAGA | 1 | 120459141 | 120459282 |
| NOTCH2_EX34_16MPLXID663 | TGTCTCGATTGGCAAAATGGT | AAACTGCTATATTCTCAAGAGTGTTATTAAC | 1 | 120459233 | 120459377 |
| NOTCH2_EX28_1MPLXID645 | ACAAGATATGCTTTTCTAGTCATCCC | TTTCACTCTTCGCCGAGATG | 1 | 120464791 | 120464930 |
| NOTCH2_EX28_2MPLXID646 | CTCACGACGCTTGTGATTG | ATCTCCTTGCTGTTGCTGTT | 1 | 120464888 | 120465033 |
| NOTCH2_EX28_3MPLXID647 | CCCCAGCAGAATAATAAACAGAATG | GCTGACATTGAGAGGTTAATGTTTTAT | 1 | 120464984 | 120465128 |
| NOTCH2_EX27_1MPLXID642 | GGCTACATAATGAAAATTGTTCCCC | CAGCTCTCCTGGCCTCT | 1 | 120465182 | 120465321 |
| NOTCH2_EX27_2MPLXID643 | TATGACAGGGTCCCCTGTAT | CTTCTCCCTTGGCATCCC | 1 | 120465279 | 120465423 |
| NOTCH2_EX27_3MPLXID644 | CTGCTGCATCCGTGTTCT | CTGCACTCTTCTGTTTTACCCC | 1 | 120465322 | 120465461 |
| NOTCH2_EX26_1MPLXID638 | AATTAGCCTTGAAGTTCAGAAACC | GGGAACTCATGGTGTACCC | 1 | 120466226 | 120466365 |
| NOTCH2_EX26_2MPLXID639 | CTTCATAGCAGCTGACTTCTCA | TTGATGCCACCTGAACAACT | 1 | 120466316 | 120466465 |
| NOTCH2_EX26_3MPLXID640 | AAGAAGCTGCGAGCATCC | GACAACCACTGTGACCAGG | 1 | 120466422 | 120466576 |
| NOTCH2_EX26_4MPLXID641 | AACCACACTCCTCACTGTTG | AAATGAAAAAGCAAATAGAGCTCCAG | 1 | 120466534 | 120466678 |
| GATA2_EX6_1MPLXID553 | CTGCTAAGGGTTTGGTCCA | CTCCGACGCCCATCC | 3 | 128199802 | 128199941 |
| GATA2_EX6_2MPLXID554 | TCTGTTCCCTAGCCCATGG | GACACATGGCACCTGTGG | 3 | 128199854 | 128200001 |
| GATA2_EX6_3MPLXID555 | CAGGATGTGTCCGGAGTG | TCCAAGAAGAGCAAGAAAGGG | 3 | 128199946 | 128200095 |
| GATA2_EX6_4MPLXID556 | CACTTTGACAGCTCCTCGAA | AACTGGCCCTCTGAAAACTG | 3 | 128200046 | 128200195 |
| GATA2_EX5_1MPLXID551 | GCACAAAGCGCAGAGGT | GACAACCACCACCTTATGGC | 3 | 128200604 | 128200743 |
| GATA2_EX5_2MPLXID552 | TTGTAGTAGAGGCCACAGGC | GGAGTCCAGCCTGCTG | 3 | 128200672 | 128200823 |
| GATA2_EX4_1MPLXID549 | GTAATTAACCGCCAGCTCCT | ACTACCTGTGCAATGCCTG | 3 | 128202643 | 128202782 |
| GATA2_EX4_2MPLXID550 | ATTCATCTTGTGGTAGAGGCC | AACTTGCCGGTTAAGCAGG | 3 | 128202742 | 128202896 |
| GATA2_EX3_1MPLXID542 | CCACGAAGTCCCCAGC | TATGTGCCGGCGGCT | 3 | 128204542 | 128204687 |
| GATA2_EX3_2MPLXID543 | CCGCTGCTGTAGTCGTG | AAGTACCAGGTGTCACTGAC | 3 | 128204653 | 128204807 |
| GATA2_EX3_3MPLXID544 | GACTGCCACTTTCCATCTTCA | GAAGTGTCTCCTGACCCTAG | 3 | 128204759 | 128204903 |
| GATA2_EX3_4MPLXID545 | CCCCGCGGAAGATGAG | CAGCGGGAGCTCAGTG | 3 | 128204844 | 128204988 |
| GATA2_EX3_5MPLXID546 | GAAGCCGAAAAGGTGGGA | CAAGACGCCACTGCACC | 3 | 128204922 | 128205075 |
| GATA2_EX3_6MPLXID547 | CCTGGGTACACAGAGAGTG | TTTGCCCTGGCTGGAC | 3 | 128205013 | 128205159 |
| GATA2_EX3_7MPLXID548 | CAGAGAGGGCTGCTTTGC | CTCTGCCTACCCTGATCTTTC | 3 | 128205122 | 128205266 |
| GATA2_EX2_1MPLXID539 | GATTCCTGCGGATCCTACATC | AACCCCGCTCACGCG | 3 | 128205540 | 128205691 |
| GATA2_EX2_2MPLXID540 | GCTGTAGGAGACGCGC | ACTCACACCACCCGGG | 3 | 128205656 | 128205807 |
| GATA2_EX2_3MPLXID541 | GTTCCATGTAGTTGTGCGC | GTTGCCGTCTGCACCC | 3 | 128205769 | 128205915 |
| MYC_EX1_1MPLXID593 | CGCTTCTCTGAAAGGCTCTC | TATTTAGGCATTCGACTCATCTCAG | 8 | 128748802 | 128748946 |
| MYC_EX2_1MPLXID594 | CTCAAGACTGCCTCCCG | TCTCCTCCTCGTCGCAG | 8 | 128750457 | 128750596 |
| MYC_EX2_2MPLXID595 | CTCGGTGCAGCCGTATTT | CGGAGCGGCGGCTAG | 8 | 128750559 | 128750710 |
| MYC_EX2_3MPLXID596 | AGAAATTCGAGCTGCTGCC | ACCATCTCCAGCTGGTCG | 8 | 128750660 | 128750813 |
| MYC_EX2_4MPLXID597 | ACAACGACGGCGGTG | ACAGTCCTGGATGATGATGTTTT | 8 | 128750762 | 128750907 |
| MYC_EX2_5MPLXID598 | CAGAGTTTCATCTGCGACCC | TGCCGCTGTCTTTGCG | 8 | 128750845 | 128750989 |
| MYC_EX2_6MPLXID599 | AAGCTCGTCTCAGAGAAGCT | GGTCGATGCACTCTGAGG | 8 | 128750935 | 128751079 |
| MYC_EX2_7MPLXID600 | CTTGTACCTGCAGGATCTGAG | TCGAGGAGAGCAGAGAATCC | 8 | 128751033 | 128751187 |
| MYC_EX2_8MPLXID601 | GCCTCGCAAGACTCCAG | GGCTTCGCTTACCAGAGTC | 8 | 128751133 | 128751277 |
| MYC_EX2_9MPLXID602 | CGGAGTCCTCCCCGC | AATGAAAATGGGAAAGGTATCCAG | 8 | 128751188 | 128751327 |
| MYC_EX3_1MPLXID603 | ATGTAACCTTGCTAAAGGAGTGAT | CAGCAGAAGGTGATCCAGAC | 8 | 128752598 | 128752737 |
| MYC_EX3_2MPLXID604 | CTCCTGGCAAAAGGTCAGA | GGATAGTCCTTCCGAGTGGA | 8 | 128752699 | 128752843 |
| MYC_EX3_3MPLXID605 | CACATCAGCACAACTACGCA | TTGACATTCTCCTCGGTGTC | 8 | 128752795 | 128752948 |
| MYC_EX3_4MPLXID606 | AACAACCGAAAATGCACCAG | TTTTCCAACTCCGGGATCTG | 8 | 128752896 | 128753041 |
| MYC_EX3_5MPLXID607 | ACGAGCTAAAACGGAGCTTT | TCAGAAATGAGCTTTTGCTCCT | 8 | 128752987 | 128753131 |
| MYC_EX3_6MPLXID608 | CACAGCATACATCCTGTCCG | AATCGTTTTCCTTACTTTTCCTTACG | 8 | 128753081 | 128753225 |
| MYC_EX3_7MPLXID609 | AGGACTTGTTGCGGAAACG | TTTGAAACAAGTTCATAGGTGATTGC | 8 | 128753134 | 128753273 |
| CALR_EX9_1MPLXID1016 | CGTAACAAAGGTGAGGCCT | AGCCTCTGCTCCTCGT | 19 | 13054434 | 13054573 |
| CALR_EX9_2MPLXID1017 | AGCAGAGAAACAAATGAAGGACA | CTTCCTCCTTGTCCTCCTCA | 19 | 13054529 | 13054683 |
| CALR_1 | AGAGGCTTAAGGAGGAGGAAGAA | CCTCTCTACAGCTCGTCCTTGG | 19 | 13054567 | 13054732 |
| CALR_EX9_3MPLXID1018 | caaggaggatgatgaggacaaa | GCAGGAGCGCTCAGG | 19 | 13054625 | 13054771 |
| CXCR4_EX2_1MPLXID416 | CTGTACAATATTGGTCAGTCTTTTATATCTG | TCATCTGTTTCCACTGAGTCTG | 2 | 136872347 | 136872486 |
| CXCR4_EX2_2MPLXID417 | TTAGCTGGAGTGAAAACTTGAAGA | TGGAGCCAAATTTAAAACCTCTG | 2 | 136872439 | 136872583 |
| CXCR4_EX2_3MPLXID418 | TCTGCTCACAGAGGTGAGT | TGAGTTTGAGAACACTGTGCA | 2 | 136872532 | 136872676 |
| CXCR4_EX2_4MPLXID419 | CTCGGTGATGGAAATCCACTT | CACAGTCATCCTCATCCTGG | 2 | 136872634 | 136872778 |
| CXCR4_EX2_5MPLXID420 | TGTAGTAAGGCAGCCAACAG | ATCCTGCCTGGTATTGTCATC | 2 | 136872729 | 136872873 |
| CXCR4_EX2_6MPLXID421 | CAGCTTGGAGATGATAATGCAATAG | TGAGGCAGATGACAGATATATCTG | 2 | 136872820 | 136872964 |
| CXCR4_EX2_7MPLXID422 | AAACTGGAACACAACCACCC | TTGGCTGAAAAGGTGGTCTAT | 2 | 136872895 | 136873047 |
| CXCR4_EX2_8MPLXID423 | GCAGGGATCCAGACGC | ACACAGTCAACCTCTACAGC | 2 | 136873007 | 136873151 |
| CXCR4_EX2_9MPLXID424 | CGGTCCAGACTGATGAAGG | TCTTTGTCATCACGCTTCCC | 2 | 136873097 | 136873241 |
| CXCR4_EX2_10MPLXID425 | AAAGTACCAGTTTGCCACGG | ATGGATTGGTCATCCTGGTC | 2 | 136873186 | 136873331 |
| CXCR4_EX2_11MPLXID426 | CATGCTTCTCAGTTTCTTCTGGT | ATGAAGGAACCCTGTTTCCG | 2 | 136873282 | 136873428 |
| CXCR4_EX2_12MPLXID427 | CAGGAAGATTTTATTGAAATTAGCATTTTCTT | TTGGAAGTGAATGTCCATTCCT | 2 | 136873375 | 136873519 |
| CXCR4_EX1_1MPLXID415 | GCTGCGCTCTAAGTTCAAAC | CAGCAGGTAGCAAAGTGACG | 2 | 136875555 | 136875703 |
| TNFAIP3_EX2_1MPLXID921 | AGCTATAGAGGAGTCGTATTAAAGTCA | TCACAGCTTTCCGCATATTG | 6 | 138192283 | 138192422 |
| TNFAIP3_EX2_2MPLXID922 | CAAGTCCTTCCTCAGGCTTT | TGAACATTTCCAGTGTGTATCGG | 6 | 138192374 | 138192518 |
| TNFAIP3_EX2_3MPLXID923 | ACTAATGGGATCATTCATCATTTTAAAACC | TTTCTTCTGGCTTTCCAGGG | 6 | 138192461 | 138192610 |
| TNFAIP3_EX2_4MPLXID924 | CAAAGCCCTCATCGACAGAA | GAGCTATCACCCAGGCAAAA | 6 | 138192559 | 138192703 |
| TNFAIP3_EX3_1MPLXID925 | CCCCTAGAATAGCAGTAGGGC | GTACTGAGAAGTGGCATGCA | 6 | 138195880 | 138196019 |
| TNFAIP3_EX3_2MPLXID926 | CTTTCTGTCCTCAGGTGACG | CCAGCGGAATTTAAAGTTGCG | 6 | 138195968 | 138196112 |
| TNFAIP3_EX3_3MPLXID927 | CACGCTCAAGGAAACAGACA | CCATGGAGCTCTGTTAGTAGATAATTAG | 6 | 138196070 | 138196214 |
| TNFAIP3_EX4_1MPLXID928 | AAATAAGCTGAGTTATATAAATGAATAATTGTAGA | GATAAGATTGTCCCATTCATCATTCC | 6 | 138196715 | 138196854 |
| TNFAIP3_EX4_2MPLXID929 | GGAGTACAGGATACATTCAAGCTT | TGTATTTCTTCCAGTGAGTTGTACT | 6 | 138196778 | 138196922 |
| TNFAIP3_EX4_3MPLXID930 | CATGGCCCGAAGTGGAC | ATAAGGCTGAAAGCATTTAAGTACAG | 6 | 138196878 | 138197022 |
| TNFAIP3_EX5_1MPLXID931 | TCCTGGAGAAAACCACACTG | GAAATTGGAACCTGATTCCAAACTT | 6 | 138197031 | 138197170 |
| TNFAIP3_EX5_2MPLXID932 | CCTTTCTCTTTCTTTGAACAGACAAA | TCATAGCCGAGAACAATGGG | 6 | 138197112 | 138197256 |
| TNFAIP3_EX5_3MPLXID933 | GCCCAGGAATGCTACAGATAC | GAAAACCCTGATGTTTCAGTGTC | 6 | 138197216 | 138197360 |
| TNFAIP3_EX6_1MPLXID934 | TGTAAGTTATATCTTTTATACATTTTCAAAATGAG | CTTCCCCGGTCTCTGTTAAC | 6 | 138198113 | 138198252 |
| TNFAIP3_EX6_2MPLXID935 | TTTTCCTTAGAAATCCGAGCTGT | GGGATTTCTATCACCATTAAGTACTCT | 6 | 138198203 | 138198345 |
| TNFAIP3_EX6_3MPLXID936 | ATCCTGAAAATGAGATGAAGGAGAA | AGATGACACAGGAGAGAGCT | 6 | 138198285 | 138198430 |
| TNFAIP3_EX7_1MPLXID937 | AATCTTGTGTGTGATTTTGTGTATTCT | TGCCATTTCTTGTACTCATGCT | 6 | 138199513 | 138199652 |
| TNFAIP3_EX7_2MPLXID938 | AAATCAATCTGGTAGATGATTACTTTGAAC | TTTACATCCATGAGAGAAAGCTGG | 6 | 138199595 | 138199739 |
| TNFAIP3_EX7_3MPLXID939 | CAGAATCCCATGGAACCTTCC | GTTTGTTTTGATTCTTTTGCCGC | 6 | 138199690 | 138199834 |
| TNFAIP3_EX7_4MPLXID940 | CTTTATGCCATGAGTGCTCAGA | ACTCCTCAGGGTTCCACG | 6 | 138199787 | 138199939 |
| TNFAIP3_EX7_5MPLXID941 | GAGAAGCCTATGAGCCCTTG | TGCACATTCAGTGTGAAGGG | 6 | 138199901 | 138200051 |
| TNFAIP3_EX7_6MPLXID942 | GACCACTGCCATGAAGTGC | CTTCCCGGGATCCAAGTG | 6 | 138199998 | 138200142 |
| TNFAIP3_EX7_7MPLXID943 | TTTTGTGAACGTTGCCACAAC | TTTTGAAGCAAGTACTGCAGATC | 6 | 138200062 | 138200206 |
| TNFAIP3_EX7_8MPLXID944 | CTCCAGGATGTTACCAGGAC | GGCTCCGGACGAGCC | 6 | 138200155 | 138200302 |
| TNFAIP3_EX7_9MPLXID945 | CAGCGTTCCAAGTCAGATCC | TTTTCTGCACTTGCTCGTCC | 6 | 138200263 | 138200409 |
| TNFAIP3_EX7_10MPLXID946 | CTGTCTCAAGCTGCACGG | TTCACTCACGTTTGTTTTCTCTG | 6 | 138200353 | 138200497 |
| TNFAIP3_EX7_11MPLXID947 | ACTCCTGGGGACAGGAC | GCTGTGTTAGGAAGTCAACCA | 6 | 138200371 | 138200519 |
| TNFAIP3_EX8_1MPLXID948 | TGGCCTAATCTGTATTTGGAACC | CTGTGGGACTGACTTTCCC | 6 | 138201104 | 138201243 |
| TNFAIP3_EX8_2MPLXID949 | CATCCATTCTCATGTAGATTTTGCTG | GAAACACTTCTGGCAGTATCCT | 6 | 138201191 | 138201335 |
| TNFAIP3_EX8_3MPLXID950 | CCTTGGAAGCACCATGTTTG | AAAGCATCGAACACACGGG | 6 | 138201293 | 138201438 |
| TNFAIP3_EX9_1MPLXID951 | TTTCCTGACTTTTTAATGATCTGCC | TCCTCGCTGCGGCAG | 6 | 138202131 | 138202275 |
| TNFAIP3_EX9_2MPLXID952 | TCCTGCAAGAACATCCTGG | GCCAAAATGATCACAGGC | 6 | 138202241 | 138202396 |
| TNFAIP3_EX9_3MPLXID953 | CAGCGTTGCCGGGCC | TGACTGACAGCTCGAGGC | 6 | 138202361 | 138202510 |
| NOTCH1_EX34_1MPLXID621 | ACAGACGCCCGAAGG | AACGTCTCCGACTGGTCC | 9 | 139390470 | 139390609 |
| NOTCH1_EX34_2MPLXID622 | AATGCGGGCGATCTGG | CTACAGGTGCCTGAGCAC | 9 | 139390541 | 139390687 |
| NOTCH1_EX34_3MPLXID623 | GAGCTGGACCACTGGTC | CTGGTCCCACCCGTGA | 9 | 139390623 | 139390777 |
| NOTCH1_EX34_4MPLXID624 | CAGGAACTGGGCTGCG | AGCTTCCTGAGTGGAGAGC | 9 | 139390745 | 139390897 |
| NOTCH1_EX34_5MPLXID625 | GAATAGTGTGCACCGCCA | AAAGCCTGCAGCCGC | 9 | 139390822 | 139390974 |
| NOTCH1_EX34_6MPLXID626 | CTGAGCTCACGCCAAGG | CCAGCCTCACCTGGTG | 9 | 139390921 | 139391066 |
| NOTCH1_EX34_7MPLXID627 | TAAGTTTTGTGGCTGCACCT | TGCAGCATGGCATGGTAG | 9 | 139391021 | 139391166 |
| NOTCH1_EX34_8MPLXID628 | GCAGCAAGGCTACTGTGC | AATGGTCAATGCGAGTGGC | 9 | 139391124 | 139391278 |
| NOTCH1_EX34_9MPLXID629 | TTGGTTCGGCACCATGC | CTCGTCTCTCCCACCTGC | 9 | 139391225 | 139391379 |
| NOTCH1_EX34_10MPLXID630 | CCCACAGTGAAATTCAGGGC | CCAAGCCCGAGATGGC | 9 | 139391295 | 139391439 |
| NOTCH1_EX34_11MPLXID631 | CAGTCTCAAAGGCCAGCC | TCCCCGTTCCAGCAGT | 9 | 139391386 | 139391530 |
| NOTCH1_EX34_12MPLXID632 | GGTGTCGGGCATCCC | CAGCTCCGGCATGCTC | 9 | 139391471 | 139391615 |
| NOTCH1_EX34_13MPLXID633 | CACGTCTGACAGGTAGCC | CTGGCCTGTGGAAGCAA | 9 | 139391552 | 139391695 |
| NOTCH1_EX34_14MPLXID634 | TCTTCCTCCGTGCCTTGA | CCACCCTGTCGCCCC | 9 | 139391647 | 139391793 |
| NOTCH1_EX34_15MPLXID635 | AGCCGTTGGGCGAGC | CCGCGCGACATCGCA | 9 | 139391758 | 139391902 |
| NOTCH1_EX34_16MPLXID636 | CCTCACGATGTCGTGATGC | GAGACACCCCTGTTTCTGG | 9 | 139391858 | 139392007 |
| NOTCH1_EX34_17MPLXID637 | CAGGCGGTCCATATGATCC | GGTCCCCACGCCTGG | 9 | 139391903 | 139392063 |
| BRAF_EX15_1MPLXID300 | ACTTTCTAGTAACTCAGCAGCATC | AGCTACAGTGAAATCTCGATGG | 7 | 140453005 | 140453144 |
| BRAF_EX15_2MPLXID301 | CCAGACAACTGTTCAAACTGATG | GGAAAATGAGATCTACTGTTTTCCTTTAC | 7 | 140453091 | 140453236 |
| EZH2_EX20_1MPLXID508 | GAAGCTAAGGCAGCTGTTTCA | GATGGGTGGCCATCCAG | 7 | 148504691 | 148504841 |
| EZH2_EX19_1MPLXID507 | AAAGCCCTTAGAGATCATGCTAG | GCTCACTGACACCAGTGTG | 7 | 148506123 | 148506277 |
| EZH2_EX18_1MPLXID506 | GAAAAGGGAGTTCCAATTCTCAC | ACTGGGCTGTGCTTACTTTT | 7 | 148506364 | 148506512 |
| EZH2_EX17_1MPLXID505 | CAACTCAGGAACTCACTGCC | TCCAAAAGAATTTTCTCCTGTGTCT | 7 | 148507386 | 148507541 |
| EZH2_EX16_1MPLXID503 | AATCAAACCCACAGACTTACCTAAT | GATCCTGTGCAGAAAAATGAATTCA | 7 | 148508622 | 148508761 |
| EZH2_EX16_2MPLXID504 | CTTACCTCTCCACAGTATTCTGAG | AGTCCATTTTCACCCTCCTTTT | 7 | 148508712 | 148508864 |
| EZH2_EX15_1MPLXID501 | ACTACAAACAATCTTCCAGAAGTGA | AGAGTGTGACCCTGACCT | 7 | 148511014 | 148511153 |
| EZH2_EX15_2MPLXID502 | CGGCTCCACAAGTAAGACAG | GAGTGAAGAACCTCCAAACCT | 7 | 148511116 | 148511260 |
| EZH2_EX14_1MPLXID499 | TATTTAGTTCTCATGCAATTGCATCAA | CAACCCTGTGATCATCCACG | 7 | 148511963 | 148512102 |
| EZH2_EX14_2MPLXID500 | CACGAACTGTCACAAGGCT | GTACTCGTGTTAATTGTGTATGATCG | 7 | 148512062 | 148512206 |
| EZH2_EX13_1MPLXID498 | AAGCAGATATTGTTAAGCTAATAATGAGAG | CAGCTCTCTGTTGGATTTGTAGC | 7 | 148512550 | 148512703 |
| EZH2_EX12_1MPLXID496 | AAAATAGACTAAGTAGAAACCAACAACAG | ATGTGGATACTCCTCCAAGGA | 7 | 148513676 | 148513815 |
| EZH2_EX12_2MPLXID497 | ACCGGTGTTTCCTCTTCTTTT | GCCTAAATAAGACTGTCCTCATGG | 7 | 148513774 | 148513918 |
| EZH2_EX11_1MPLXID493 | GGAGCTTAGTAATAACCAAGAATTTTCTTT | CCATTGCTAGGTTAATTGGGAC | 7 | 148514212 | 148514351 |
| EZH2_EX11_2MPLXID494 | TTTTATCGGATATCTTACCTGTCTACATG | TATTGAACCTCCTGAGAATGTGG | 7 | 148514296 | 148514440 |
| EZH2_EX11_3MPLXID495 | CTAAACATTGAGGCTTCAGCAC | TATTATTTCCAATCATTTCTTGACCAGTG | 7 | 148514387 | 148514531 |
| EZH2_EX10_1MPLXID490 | TTCAATGCATTATACATCCTTAATCCTC | CAGGGACTGAAACGGGG | 7 | 148514889 | 148515043 |
| EZH2_EX10_2MPLXID491 | TCTTTCTTCTCTTCTTCTTCTTTATCATTG | AGAAGAGGACGGCTTCCC | 7 | 148514989 | 148515134 |
| EZH2_EX10_3MPLXID492 | CTGGGCCTGCTACTGTTATT | CTTTGCCCTGATGTTGACATTTT | 7 | 148515097 | 148515243 |
| EZH2_EX9_1MPLXID488 | CATTAACGCTGACTTGATCACC | GAACACAGAAACAGCTCTAGACA | 7 | 148516606 | 148516745 |
| EZH2_EX9_2MPLXID489 | CTGTGGTCCACAAGGTTTGT | TCCAGTGGAACTGGAAGAGT | 7 | 148516703 | 148516854 |
| EZH2_EX8_1MPLXID485 | TTCTAGTTGTAATAAATGATAGCACTCTCC | GCTTTTCTGTAGGCGATGTTTTAAA | 7 | 148523465 | 148523604 |
| EZH2_EX8_2MPLXID486 | CAATAATTGCACTTACGATGTAGGAAG | CACTTCCTCCTGAATGTACCC | 7 | 148523545 | 148523689 |
| EZH2_EX8_3MPLXID487 | GCATTTGGTCCATCTATGTTGG | GGAAACCTTTTAGAAACTGTTTTCAAAAA | 7 | 148523647 | 148523801 |
| EZH2_EX7_1MPLXID483 | GCTACATTGATTCCATTTGTAATAAACCA | AGCCATTTCCTCAATGTTTCCA | 7 | 148524170 | 148524309 |
| EZH2_EX7_2MPLXID484 | CCTTTAGTTCTTCTGCTGTGC | AGATCAAAAACTTGTTTACTTCCATTCTT | 7 | 148524260 | 148524414 |
| EZH2_EX6_1MPLXID481 | TTGAAAGAAAGCTGTAATGGCTAC | GCCCTTGGTCAATATAATGATGAT | 7 | 148525783 | 148525928 |
| EZH2_EX6_2MPLXID482 | CTTCAGGATCGTCTCcatcatc | ACATTATTGCTTCTCCTGTGTGT | 7 | 148525874 | 148526018 |
| EZH2_EX5_1MPLXID479 | AAGTGTCTCTCAATTCTTTAGCCC | CCTTATATGGGAGATGAAGTTTTAGATCA | 7 | 148526765 | 148526910 |
| EZH2_EX5_2MPLXID480 | TTTATTAGTTCTTCAATGAAAGTACCATCC | TTTCACATTTGATACTTAGAGTAAACCTG | 7 | 148526852 | 148526996 |
| EZH2_EX4_1MPLXID477 | TTTTTACTTCAAATAAGTTATTATCAAATAAGCAG | ACAAGTCATCCCATTAAAGACTCT | 7 | 148529671 | 148529810 |
| EZH2_EX4_2MPLXID478 | AGAATACATTATGGGTACTGAAGCAA | GGCTACAGCTTAAGGTTGTCC | 7 | 148529753 | 148529902 |
| EZH2_EX3_1MPLXID475 | TAACCAAACAAATGTTCCAATAGCATAA | GAAACAGCGAAGGATACAGCC | 7 | 148543489 | 148543628 |
| EZH2_EX3_2MPLXID476 | CAATGAGCTCACAGAAGTCAGG | TTCTCCTTTCCTCTCCTTCATTTT | 7 | 148543577 | 148543724 |
| EZH2_EX2_1MPLXID473 | AACTTATTGAACTTAGGAGGGGAAA | CGTGTAAAATCAGAGTACATGCG | 7 | 148544200 | 148544339 |
| EZH2_EX2_2MPLXID474 | CGTCTGAACCTCTTGAGCTG | TCAGGCTGGTTAGATTAGTGATTTT | 7 | 148544290 | 148544444 |
| TCF3_EX19_1MPLXID1160 | CAGGGCTGAAAGCGGG | CGAGAAGAGGAAAAGGTGTCA | 19 | 1611664 | 1611810 |
| TCF3_EX19_2MPLXID1161 | GAGCTGAAAGCACCATCTGG | ACATCTTTCTCCTCCCTGGG | 19 | 1611753 | 1611903 |
| TCF3_EX17_1MPLXID848 | AAGCACAGGAGGACCCC | GCTGCTCATCCTGCAGC | 19 | 1612125 | 1612264 |
| TCF3_EX17_2MPLXID849 | CCAGGATGACCTGCACG | ATGGCCAATAACGCGCG | 19 | 1612227 | 1612374 |
| TCF3_EX17_3MPLXID850 | CTCGTTAATATCCCGCACGC | TTGGCGCATCACTAACAGAG | 19 | 1612327 | 1612472 |
| TCF3_EX18_1MPLXID1157 | AAGGAGAACGAGGGCAG | CCAGACCAAACTGCTCATCC | 19 | 1615212 | 1615351 |
| TCF3_EX18_2MPLXID1158 | GTTCAGGATGACCGAGACAG | CAATAACGCCCGGGAGC | 19 | 1615303 | 1615447 |
| TCF3_EX18_3MPLXID1159 | GTTGATGTCACGGACCCG | CTCACTGTCTCCCTCTGGC | 19 | 1615408 | 1615562 |
| NPM1_EX10_1MPLXID664 | ATGTCTATGAAGTGTTGTGGTTCC | TGTTACAGAAATGAAATAAGACGGAAAAT | 5 | 170837477 | 170837626 |
| BCL6_EX10_1MPLXID984 | TGTTACAGTATCCTTTGGGTAGATTC | CCATCACCAACACCAAGGT | 3 | 187440180 | 187440319 |
| BCL6_EX10_2MPLXID985 | GCTGACACGCGGTATTGC | TGATGACAGCTAACAGTTTGCC | 3 | 187440283 | 187440427 |
| BCL6_EX9_1MPLXID1010 | CACATCCCCGCAGGTC | AAGCCCTATCCCTGTGAAATC | 3 | 187442685 | 187442824 |
| BCL6_EX9_2MPLXID1011 | GTGGCTCTTCAGAGTCTGAAG | CACCCCAGAAGACCTCTCA | 3 | 187442762 | 187442904 |
| BCL6_EX8_1MPLXID1008 | tcctccgctcgcctg | ACCTGAAAACCCACACTCG | 3 | 187443224 | 187443363 |
| BCL6_EX8_2MPLXID1009 | TTTGTAGGGCTTCTCTCCAGA | CCTTCCTTATTGAATATGGCTTTTCTTT | 3 | 187443317 | 187443466 |
| BCL6_EX7_1MPLXID1006 | CCCAATAATTGTGGAGAGTTCG | GCAGACCCACAGTGACAA | 3 | 187444474 | 187444613 |
| BCL6_EX7_2MPLXID1007 | GCGGTCACACTTGTAGGG | CTTCCTCGTGCTGACTTTGG | 3 | 187444577 | 187444730 |
| BCL6_EX6_1MPLXID1003 | AGCATAAGTAAAATGGAGCACAAAA | ACGTTCCCTGAGGAGATGG | 3 | 187446063 | 187446202 |
| BCL6_EX6_2MPLXID1004 | AGCTAGAATCTGAGTACTCAGACT | CACTCACCACTCTACATGCA | 3 | 187446151 | 187446295 |
| BCL6_EX6_3MPLXID1005 | GGACGTGCACTTCGGG | CTTGCCTACACACAGGGAG | 3 | 187446257 | 187446411 |
| BCL6_EX5_1MPLXID992 | CTGCCCAGGTAACCCC | AACCTTGACCTCCAGTCC | 3 | 187446790 | 187446929 |
| BCL6_EX5_2MPLXID993 | TAACGATGTTATTGAGCCGGC | CTTTCCCCACGAGCCTAC | 3 | 187446842 | 187446986 |
| BCL6_EX5_3MPLXID994 | TCAGGCTCCATGGGTGG | GCAACTGGAAGAAATACAAGTTCAT | 3 | 187446931 | 187447075 |
| BCL6_EX5_4MPLXID995 | TTGAGGCTGTTGAGCACG | AAATCTGACTGCCAGCCC | 3 | 187447033 | 187447187 |
| BCL6_EX5_5MPLXID996 | CATTCTTACTGCTGCAGGACT | AAGAAGAGAGACCCTCCTCG | 3 | 187447136 | 187447288 |
| BCL6_EX5_6MPLXID997 | AATGCAGGGCAATCTCATCT | GAGGCACGAAGTGATATGCA | 3 | 187447247 | 187447391 |
| BCL6_EX5_7MPLXID998 | CAGGTTTGAGGCCCTCAG | GATAGTGCCAGGCCAGTC | 3 | 187447343 | 187447496 |
| BCL6_EX5_8MPLXID999 | GACACCTCCAAAGTCGGC | ACCTCCCTGTCAGCAGC | 3 | 187447444 | 187447594 |
| BCL6_EX5_9MPLXID1000 | CGAAACTCCTCATCGGAGAAG | CAACCTGCCACTGAGGAG | 3 | 187447552 | 187447698 |
| BCL6_EX5_10MPLXID1001 | ACATGGAATAAGAGGCTGGC | GATGCCCCAAGACATCATGG | 3 | 187447601 | 187447746 |
| BCL6_EX5_11MPLXID1002 | CCTCACGACCCCGATAGG | AACAGTTCTGATTTGGGGCC | 3 | 187447709 | 187447858 |
| BCL6_EX4_1MPLXID989 | TATGCATGAATTATCAAAGGTTTCTGAT | ACGGCTATGTACCTGCAGA | 3 | 187449419 | 187449558 |
| BCL6_EX4_2MPLXID990 | CAAGTGTCCACAACATGCTC | ATCTAGATCCTGAGATCAACCCT | 3 | 187449518 | 187449662 |
| BCL6_EX4_3MPLXID991 | GTCCAGGAGGATGCAGAATC | TTCGATATGTCTACTATAGTGGGACA | 3 | 187449616 | 187449760 |
| BCL6_EX3_1MPLXID986 | CTTGGCTGCCAGCACC | AGACATCTTGACTGATGTTGTCAT | 3 | 187451259 | 187451398 |
| BCL6_EX3_2MPLXID987 | CTCTAAACTGCTCACGGCTC | AGTGAAGACAAAATGGCCTCG | 3 | 187451349 | 187451493 |
| BCL6_EX3_3MPLXID988 | CGACTCCGGAGACGATTAAG | GAAACAGGTTCTGGAGGACTAAC | 3 | 187451399 | 187451538 |
| MEF2B_EX9_1MPLXID1141 | CGCCGTACCTGGCGA | CTATCCCTTGCTCCTCGC | 19 | 19256560 | 19256699 |
| MEF2B_EX9_2MPLXID1142 | GGGTGATCTCCTACCGGG | CGCCTCTCTCCGGCC | 19 | 19256596 | 19256749 |
| MEF2B_EX9_3MPLXID1143 | GAAGGTCTTAGGAAAGTcgcc | CCTCCCTCTCTCTTTCCCC | 19 | 19256702 | 19256856 |
| MEF2B_EX9_4MPLXID1144 | CTCAGACTTGATGCTGACTGG | CTGGCTTCCTTCCCTTCC | 19 | 19256750 | 19256889 |
| MEF2B_EX8_1MPLXID1138 | TGTGCGCAGTACCAGG | CCTGGCAGCCCTCGA | 19 | 19256994 | 19257133 |
| MEF2B_EX8_2MPLXID1139 | TCGTCTCAGGGCACTTACC | ATTCCTGCCCACCAGAATATG | 19 | 19257064 | 19257208 |
| MEF2B_EX8_3MPLXID1140 | TGGGCTGGGAGGACA | CCAGGCCTCAGCCCC | 19 | 19257083 | 19257225 |
| MEF2B_EX7_1MPLXID1134 | GGAGTGCGGACGCTTC | AAGCCTCTACAGTGGCCT | 19 | 19257316 | 19257455 |
| MEF2B_EX7_2MPLXID1135 | GGGTCCGGGAGTTGC | ACACCTCCGTGAGTGAGG | 19 | 19257404 | 19257558 |
| MEF2B_EX7_3MPLXID1136 | CCACACCCAGGTCGC | CCCCACTGTACCTGCC | 19 | 19257492 | 19257633 |
| MEF2B_EX7_4MPLXID1137 | CAGGTCTGACCTCCGC | ATTAGTTGACATGGCCTGCC | 19 | 19257593 | 19257737 |
| MEF2B_EX5_1MPLXID1132 | CAGAGCCAAGATGCACCC | GGCTTGGGGAAGCACT | 19 | 19257780 | 19257919 |
| MEF2B_EX5_2MPLXID1133 | GGAGATGGGCGGCTC | GGTCCTCTGACCCATCCC | 19 | 19257880 | 19258024 |
| MEF2B_EX4_1MPLXID1130 | AGGAGGAATGCCTCATTCAC | AGAGCTGGAGCCGGAT | 19 | 19258467 | 19258606 |
| MEF2B_EX4_2MPLXID1131 | CTCCGAAACTTCTCTCCTGG | ATAGAACTGGGTTCCCTGGG | 19 | 19258556 | 19258706 |
| MEF2B_EX3_1MPLXID1127 | TTTTGTGCAAACCAAGAGTCC | AGTACACAGAGTACAGCGAGC | 19 | 19259951 | 19260090 |
| MEF2B_EX3_2MPLXID1128 | CCTCGAGGATGTCAGTGTTG | TGTGACTGTGAGATAGCCCT | 19 | 19260034 | 19260178 |
| MEF2B_EX3_3MPLXID1129 | CGGTTGGCGCTGTTGA | CTTCTTCTTCTCTCCCCTGC | 19 | 19260135 | 19260279 |
| MEF2B_EX2_1MPLXID1126 | CCCTGCTAGGAATGTCTTTCC | CTTCTCCTCCTAGACAAAGATCATTC | 19 | 19261442 | 19261586 |
| SF3B1_EX18_1MPLXID788 | TGCTTTTCTACAAATATTAAAGTTAGTAGCAA | TTTGGGAGCAGCAGATATTGAT | 2 | 198265384 | 198265523 |
| SF3B1_EX18_2MPLXID789 | TACCATCAATCAGTTGTTCTTCAAGT | GGTGCAGCAGAAATTATATCCAG | 2 | 198265471 | 198265624 |
| SF3B1_EX18_3MPLXID790 | CGGCTTCATCTTTCAGATCATC | GCTTTATTTCCTTGGAAAAGCAGT | 2 | 198265573 | 198265717 |
| SF3B1_EX17_1MPLXID785 | ATAAACTGTGAGATAATCAAGGCAAAAA | ATGGCTTTGGATAGAAGAAATTACC | 2 | 198266014 | 198266153 |
| SF3B1_EX17_2MPLXID786 | TGAGAATATTCTTTTACAATAAAAGCTTACCT | AACAGTGTTGTGGGACAGAT | 2 | 198266094 | 198266242 |
| SF3B1_EX17_3MPLXID787 | CCTGTGCTGCCAGAAGT | ATTTCAGGTCAGTTGATTTATTATTACTATTTCTT | 2 | 198266154 | 198266294 |
| SF3B1_EX16_1MPLXID782 | GTTAGAACCATGAAACATATCCAGTTTAC | TGCCAACTACTATACTAGAGAAGTGA | 2 | 198266414 | 198266553 |
| SF3B1_EX16_2MPLXID783 | CATTTCCTCATCAGGAGACTGG | TTTTATTTAAAAATAGGGTTTGGCTGCT | 2 | 198266484 | 198266628 |
| SF3B1_EX16_3MPLXID784 | ATTCTGCATCCATAAGAGGAATAAGAT | TACCTTTTGATTTATCTTCATTAAAGTTAAGGC | 2 | 198266555 | 198266694 |
| SF3B1_EX15_1MPLXID779 | CCTTAACTTTAATGAAGATAAATCAAAAGGTAATT | TGCCTTGGCTGAAGCAG | 2 | 198266663 | 198266802 |
| SF3B1_EX15_2MPLXID780 | CTTTAACACAGAATCAAAAGATTCGATACC | GGGCATAGTTAAAACCTGTGTTTG | 2 | 198266745 | 198266887 |
| SF3B1_EX15_3MPLXID781 | GCAATGGCCAAAGCACTG | AATAGTTGATATATTGAGAGAATCTGGATGAT | 2 | 198266803 | 198266942 |
| SF3B1_EX14_1MPLXID775 | ttgagcccaaaggtttgagt | GCCATCTTGCCACATCTTAGA | 2 | 198267186 | 198267325 |
| SF3B1_EX14_2MPLXID776 | CATTACAACTTACCATGTTCAATGATTTC | AAGCTGTGTGCAAAAGCAAG | 2 | 198267267 | 198267411 |
| SF3B1_EX14_3MPLXID777 | TTAATACCAGTGTGTCTCGCTTG | ATAACATGGATGAGTATGTCCGTAAC | 2 | 198267360 | 198267504 |
| SF3B1_EX14_4MPLXID778 | GCTACAACAGCAAAAGCTCTAG | AATATTACCAACTCATGACTGTCCTT | 2 | 198267450 | 198267594 |
| IDH1_EX4_1MPLXID562 | AAAACACATACAAGTTGGAAATTTCTG | CCCGGCTTGTGAGTGG | 2 | 209113015 | 209113154 |
| IDH1_EX4_2MPLXID563 | CATGACGACCTATGATGATAGGTTT | TTCAAGTTGAAACAAATGTGGAAATCA | 2 | 209113107 | 209113251 |
| IDH1_EX4_3MPLXID564 | CCACCCAGAATATTTCGTATGGT | GACCAAGTCACCAAGGATGC | 2 | 209113193 | 209113347 |
| IDH1_EX4_4MPLXID565 | GCCAACATTATGCTTCTTTATAGCTT | GCAGTTGTAGGTTATAACTATCCATTTG | 2 | 209113297 | 209113448 |
| CDKN2A_EX3_1MPLXID381 | CCTGTAGGACCTTCGGTGA | GGATGTGCCACACATCTTTG | 9 | 21968162 | 21968303 |
| CDKN2A_EX2_1MPLXID377 | CAAGACCGGAGACTGGTC | CATGCCCGCATAGATGCC | 9 | 21970795 | 21970934 |
| CDKN2A_EX2_2MPLXID378 | TCATCAGTCCTCACCTGAGG | TGCGCGATGCCTGGG | 9 | 21970887 | 21971041 |
| CDKN2A_EX2_3MPLXID379 | GACATCGCGATGGCCC | CTCTCACCCGACCCGT | 9 | 21970980 | 21971128 |
| CDKN2A_EX2_4MPLXID380 | CCGGGCAGCGTCGTG | CCCACCCTGGCTCTGA | 9 | 21971097 | 21971244 |
| CDKN2A_EX1_1MPLXID375 | AGAGTCGCCCGCCAT | CTGGCTGGCCACGGC | 9 | 21974631 | 21974785 |
| CDKN2A_EX1_2MPLXID376 | TCTACCCGACCCCGG | GTCACCAGAGGGTGGG | 9 | 21974750 | 21974907 |
| CDKN2B_EX1_1MPLXID382 | ACCTCCGGCCAACGG | AGGCGCGCGATCCAG | 9 | 22008672 | 22008811 |
| CDKN2B_EX1_2MPLXID383 | CCCCGATCCGCCGAG | GAGAAGGTGCGACAGCTC | 9 | 22008727 | 22008871 |
| CDKN2B_EX1_3MPLXID384 | TGACTCCGTTGGGATCCG | CGCTGCGCGTCTGGG | 9 | 22008823 | 22008976 |
| CDKN2B_EX1_4MPLXID385 | CACTAGTCCCCGCGC | CGTTAAGTTTACGGCCAACG | 9 | 22008872 | 22009011 |
| ID3_EX2_1MPLXID561 | GTTTAAACCTCCCTCTCCAAGAG | CGCGTAACTCTTCCCTCTTTT | 1 | 23885390 | 23885542 |
| ID3_EX1_1MPLXID557 | CATCCTTGCCTGGGTGTT | TCCTACAGCGCGTCATC | 1 | 23885573 | 23885712 |
| ID3_EX1_2MPLXID558 | CTACCTGCAGGTCGAGAATG | TGAGGAGCCGCTGAGC | 1 | 23885671 | 23885816 |
| ID3_EX1_3MPLXID559 | AGCAGTGGTTCATGTCGTC | CTCACTCCCCAGCATGAAG | 1 | 23885776 | 23885930 |
| ID3_EX1_4MPLXID560 | GCTGCCGGGCCCTTC | TTTCTTTCTCTTTGGGGCACC | 1 | 23885817 | 23885957 |
| TNFRSF14_EX1_1MPLXID1162 | TGGAGTTCATCCTGCTAGCT | TGCGAGCAGACGGAGA | 1 | 2488052 | 2488206 |
| TNFRSF14_EX2_1MPLXID1163 | CTCCCAATGCCTGTCCTG | TGGCCCGCCAAAGGG | 1 | 2489137 | 2489298 |
| TNFRSF14_EX3_1MPLXID1164 | GTGGGCTCCCGAAGG | CCATTGAGGTGGGCAATGT | 1 | 2489724 | 2489869 |
| TNFRSF14_EX3_2MPLXID1165 | CTGACGGGCACAGTGTG | CATCCAGGCTGCCCAA | 1 | 2489811 | 2489956 |
| TNFRSF14_EX4_1MPLXID1166 | CCGGCCTCCACGTAC | GGTCCCCGTCCTGGA | 1 | 2491218 | 2491357 |
| TNFRSF14_EX4_2MPLXID1167 | CAGGCCACTTCTGCATCG | ACAGCGGGGCACTGG | 1 | 2491325 | 2491477 |
| TNFRSF14_EX5_1MPLXID1168 | GGTGCCCTCAGCCCC | GAGCTGGGCCTCCCC | 1 | 2492024 | 2492181 |
| TNFRSF14_EX6_1MPLXID1169 | AGTGAACACTGGGCGC | ATGACGAGGCTCCCTGA | 1 | 2493056 | 2493195 |
| TNFRSF14_EX6_2MPLXID1170 | ACTGGGTATGGTGGTTTCTC | AGAGCTCCAAGAGGTGACG | 1 | 2493159 | 2493313 |
| TNFRSF14_EX7_1MPLXID1171 | TCCCCTGATCAGACACTGC | CACCAGCCCCAGCGG | 1 | 2494253 | 2494406 |
| TNFRSF14_EX8_1MPLXID1172 | AATGAACCCGAGAACCTGG | GGTGACGTCCGGAGG | 1 | 2494516 | 2494655 |
| TNFRSF14_EX8_2MPLXID1173 | CACAGTCATTGAGGCCCTG | CTCTTTCAGCAGCCGTCG | 1 | 2494616 | 2494769 |
| KRAS_EX3_1MPLXID589 | tgcatggcmttagcaaagACT | TTCTTTGTGTATTTGCCATAAATAATACTAAATC | 12 | 25380086 | 25380225 |
| KRAS_EX3_2MPLXID590 | CAATTTAAACCCACCTATAATGGTGAATAT | GATATTCTCGACACAGCAGGTC | 12 | 25380154 | 25380298 |
| KRAS_EX3_3MPLXID591 | GGTCCCTCATTGCACTGTAC | AGTAAAAGGTGCACTGTAATAATCCA | 12 | 25380250 | 25380394 |
| KRAS_EX2_1MPLXID587 | ATGGTCAGAGAAACCTTTATCTGTATC | AAGAGTGCCTTGACGATACAG | 12 | 25398134 | 25398273 |
| KRAS_EX2_2MPLXID588 | TTCGTCCACAAAATGATTCTGAATTAG | GTGTGACATGTTCTAATATAGTCACATTTT | 12 | 25398226 | 25398377 |
| DNMT3A_EX23_1MPLXID464 | TGTGTCGCTACCTCAGTTTG | TCCAACATGAGCCGCTTG | 2 | 25457116 | 25457255 |
| DNMT3A_EX23_2MPLXID465 | CACGCTCCATGACCGG | TAGACGGCTTCCGGGC | 2 | 25457202 | 25457356 |
| DNMT3A_EX22_1MPLXID462 | CAAGCACAGCAATCAGAACAG | TCCATAAAGCAGGGCAAAGA | 2 | 25458519 | 25458658 |
| DNMT3A_EX22_2MPLXID463 | TTTCTCATTCATGAAGACAGGAAAATG | TAGACGCATGACCAGTGTTG | 2 | 25458608 | 25458762 |
| DNMT3A_EX21_1MPLXID461 | CCCCAGCAGAGGTTCTAGA | TTATCCAGGTTTCTGTTGTTACAGT | 2 | 25459773 | 25459917 |
| DNMT3A_EX20_1MPLXID460 | CCCCAGGCCCAGGAG | TGGCTCATCTTCAAACCGTC | 2 | 25461973 | 25462117 |
| DNMT3A_EX19_1MPLXID458 | CAGCAGTCCAAGGTAGAAGC | CCCAAGGAGGGAGATGATC | 2 | 25463113 | 25463266 |
| DNMT3A_EX19_2MPLXID459 | CCACATTCTCAAAGAGCCAGA | CACTGTCCTATGCAGACAGC | 2 | 25463218 | 25463372 |
| DNMT3A_EX18_1MPLXID457 | GGAAGCACCAGCTGAGAA | GCCCATCACGTTGCCT | 2 | 25463465 | 25463627 |
| DNMT3A_EX17_1MPLXID455 | GAAGACGGGCTGCGC | GCTACATTGCCTCGGAGG | 2 | 25464398 | 25464537 |
| DNMT3A_EX17_2MPLXID456 | CGTGATGGAGTCCTCACAC | TCACCTGCCGAGACCA | 2 | 25464500 | 25464644 |
| DNMT3A_EX16_1MPLXID454 | CATCCTGGGACAAGGCG | GCATCTGACCTGTTGTGCT | 2 | 25466720 | 25466880 |
| DNMT3A_EX15_1MPLXID452 | AAGGGAGCTCGAGACCG | TACATGTGCGGGCACAA | 2 | 25466986 | 25467125 |
| DNMT3A_EX15_2MPLXID453 | AGCAGCCCGTAGGTACC | AGAGTCTCCTCTGCTCACTG | 2 | 25467091 | 25467240 |
| DNMT3A_EX14_1MPLXID450 | ACCACTGGAGGCCACA | GAACTGCTTTCTGGAGTGTG | 2 | 25467383 | 25467522 |
| DNMT3A_EX14_2MPLXID451 | ACAGCCTCACCTGCAG | TTTCTGACCCTTCCCGC | 2 | 25467399 | 25467548 |
| DNMT3A_EX13_1MPLXID449 | TCAACGGCACCTCTCCT | TACTCACCCCATCCCCTC | 2 | 25468087 | 25468233 |
| DNMT3A_EX12_1MPLXID448 | TCATTCAAACCTTCCTAAGTGCC | TCTGACGCCAGCTCTCC | 2 | 25468807 | 25468961 |
| DNMT3A_EX11_1MPLXID446 | TGCTCCTCGGATGCAG | GCACCACCTCCACCAG | 2 | 25468974 | 25469113 |
| DNMT3A_EX11_2MPLXID447 | CTTCTCCGCTGTGCTCTTC | CTGTAACTGACCTTGGCACC | 2 | 25469066 | 25469214 |
| DNMT3A_EX10_1MPLXID444 | TGGTGTGGATCTGCCTG | TGGAGGTGCAGAACAAGC | 2 | 25469433 | 25469572 |
| DNMT3A_EX10_2MPLXID445 | CAGGGCCCATTCAATCATG | CTGTCCTGACAACCCCAAC | 2 | 25469535 | 25469684 |
| DNMT3A_EX9_1MPLXID443 | CTCCGAGCTCCCAGCA | GCAAACAAGGCCTGGC | 2 | 25469893 | 25470055 |
| DNMT3A_EX8_1MPLXID441 | CAGGCTACTGCCAAACCC | GGGCTTCTCCTGGTGG | 2 | 25470432 | 25470571 |
| DNMT3A_EX8_2MPLXID442 | ATCCACCAAGACACAATGCG | GTGGCAGGGCCTCGT | 2 | 25470530 | 25470671 |
| DNMT3A_EX7_1MPLXID438 | CAGGACGGGAGGAGCT | GAGCCCGTGGGGTCC | 2 | 25470835 | 25470974 |
| DNMT3A_EX7_2MPLXID439 | CTTTGGTGGCATTCTTGTCC | TGCTGTGGAAGAAAACCAGG | 2 | 25470932 | 25471086 |
| DNMT3A_EX7_3MPLXID440 | ACCTTCTGAGACTCCCCG | ATGGTCCCCTTGAGTGTCA | 2 | 25471045 | 25471198 |
| DNMT3A_EX6_1MPLXID436 | ACTTCTGGTTTTCCAGTTCTGC | GAGGCTCACCTTCCAGG | 2 | 25497752 | 25497891 |
| DNMT3A_EX6_2MPLXID437 | TGCGCTTGCTGATGTAGTAG | CCTAATGCCCTAATGTCTGTCTC | 2 | 25497845 | 25497989 |
| DNMT3A_EX5_1MPLXID435 | CACCCGTGTCCTTCTTCTAG | TAATTCTGCCGACCAACGAA | 2 | 25498310 | 25498461 |
| DNMT3A_EX4_1MPLXID431 | AGAGCCAAGTCCCTGACT | AGCCTCAAGAGCAGTGGA | 2 | 25505238 | 25505377 |
| DNMT3A_EX4_2MPLXID432 | CTCGGCCCTCCTTGG | GAAGCGGAGTGAGCCC | 2 | 25505328 | 25505476 |
| DNMT3A_EX4_3MPLXID433 | CAGGGTCTCAGCTGCAC | ATCCATGGCCCAGGACT | 2 | 25505383 | 25505530 |
| DNMT3A_EX4_4MPLXID434 | CCCCATTGGGTAATAGCTCTG | TAAGCCCACTGATCTAACCCT | 2 | 25505484 | 25505630 |
| DNMT3A_EX3_1MPLXID429 | TACATCACTGCCATCGACAG | AAGAGCCCAGCACCAC | 2 | 25522930 | 25523069 |
| DNMT3A_EX3_2MPLXID430 | GTGCTTGCGCTTCCTCC | AATGCTACACTGCTGGGATC | 2 | 25523014 | 25523168 |
| DNMT3A_EX2_1MPLXID428 | GACAGGGCTCTCCCTCT | CCCCAGCCTGCCTCC | 2 | 25536727 | 25536878 |
| ARID1A_EX1_1MPLXID0 | GAGAAGACGAAGACAGGGC | TGCTGCTCGGCTTTCTT | 1 | 27022844 | 27022983 |
| ARID1A_EX1_2MPLXID1 | CTGGGCAACCCGCCG | GCCCTCGCTTTCCTGC | 1 | 27022931 | 27023074 |
| ARID1A_EX1_3MPLXID2 | TGAAGGCAGCCGCCG | TTCAGGTCCGGCTCCG | 1 | 27023043 | 27023196 |
| ARID1A_EX1_4MPLXID3 | GAAAGGAGCTGCAGGACG | TCCGTGAGGTTATTGTTCAGG | 1 | 27023103 | 27023250 |
| ARID1A_EX1_5MPLXID4 | GGAACGCGGGCCCTA | GTAGGGTTGCCCGAAGC | 1 | 27023208 | 27023356 |
| ARID1A_EX1_6MPLXID5 | CCTCACTCAGCCGCG | TCTGCAGCGCTGCCA | 1 | 27023300 | 27023450 |
| ARID1A_EX1_7MPLXID6 | CAACATGGCGGACAACAAAG | TAGGCGCTGCGGTTG | 1 | 27023408 | 27023559 |
| ARID1A_EX1_8MPLXID7 | CAGAACTCTCACGACCACG | TTGGAGCCGGCAGCC | 1 | 27023492 | 27023643 |
| ARID1A_EX1_9MPLXID8 | GGCCTACGCGCTGAG | CCCCATGGCCCCGAA | 1 | 27023575 | 27023719 |
| ARID1A_EX1_10MPLXID9 | GTCTTCGTCCTTCGCTCAG | GTAGCCCTGGTAGCCCC | 1 | 27023680 | 27023827 |
| ARID1A_EX1_11MPLXID10 | ACGTCGCCCAGCTCG | CCCCTCCCGAGGCGG | 1 | 27023792 | 27023951 |
| ARID1A_EX1_12MPLXID11 | CATGGCCTCGCAGTGTT | CAGCTGTGTACCTGAGGG | 1 | 27023887 | 27024042 |
| ARID1A_EX1_13MPLXID12 | CCCAACAAAGGAGCCACC | CAGCCGCCACCCACC | 1 | 27023952 | 27024092 |
| ARID1A_EX2_1MPLXID13 | TTGGGTTATATATTCAGTGGCCAG | CTGCGAGTATGGGTTAGTCC | 1 | 27056074 | 27056213 |
| ARID1A_EX2_2MPLXID14 | GATGGGCAAGATGAGACCTC | CTGCATGGTCATCGGGTAC | 1 | 27056162 | 27056309 |
| ARID1A_EX2_3MPLXID15 | CCATACGGGTCCCAGAC | ACAGATCAGATTTTGGACAGCA | 1 | 27056265 | 27056409 |
| ARID1A_EX3_1MPLXID16 | ACAAAACACTTCATCTTTCCTCATG | CTTTGCTGGTTGTAATATGGAGTC | 1 | 27057577 | 27057716 |
| ARID1A_EX3_2MPLXID17 | CCAGCGGGTATGGTCAAC | TTGAGACTGTGGCTGCTG | 1 | 27057668 | 27057813 |
| ARID1A_EX3_3MPLXID18 | CTCATGCCCAACCTTCGTAT | CTGTGTCGTCGACTGCTG | 1 | 27057773 | 27057918 |
| ARID1A_EX3_4MPLXID19 | AGTCCCCGGCTCCATA | GATACGCAGCCTGCTGG | 1 | 27057878 | 27058030 |
| ARID1A_EX3_5MPLXID20 | CCAGCACCCTCGACG | TGTAGTCACACACAGGGAAG | 1 | 27057994 | 27058138 |
| ARID1A_EX4_1MPLXID21 | TGAGAGACAGTCCCATAACCC | TTACTGGAGGTCATTGAGGG | 1 | 27059075 | 27059228 |
| ARID1A_EX4_2MPLXID22 | GATTCATTTGGGTCTCAGGCA | TCTGCCCCTATCACCTTTCA | 1 | 27059179 | 27059323 |
| ARID1A_EX5_1MPLXID23 | TCTTTCTGCCTAATATTACTAATCCATGT | CCTTGGCTGCTGGAAATCC | 1 | 27087299 | 27087438 |
| ARID1A_EX5_2MPLXID24 | TCCTGGAGTGAGCACATCA | GAGACTGAGCAACACTGGC | 1 | 27087400 | 27087554 |
| ARID1A_EX5_3MPLXID25 | CCCCGTCCCCTGTTG | ACTAGGGCTCCTTAATATTACTGG | 1 | 27087513 | 27087657 |
| ARID1A_EX6_1MPLXID26 | GCCATGATATGCTTATGTTGTTCTTT | CTAGCTCCCTGCCCCT | 1 | 27087838 | 27088001 |
| ARID1A_EX7_1MPLXID27 | GGCTGCTWAAGAAAATGTAGATGG | TGTACTGGGGCATCTGGG | 1 | 27088539 | 27088678 |
| ARID1A_EX7_2MPLXID28 | ATGCTACCCACAAATAGGTTATATGC | CCATGGAGTTCTGCTGGTAG | 1 | 27088626 | 27088771 |
| ARID1A_EX7_3MPLXID29 | GGAGGACAGATACACACAGG | CAAGGAACTGTTTTCTCCTCTCA | 1 | 27088723 | 27088867 |
| ARID1A_EX8_1MPLXID30 | ATATTGAATGACATTGTTTGGTGTTCTAG | TTGGCATTGGGCAAGGC | 1 | 27089370 | 27089509 |
| ARID1A_EX8_2MPLXID31 | CCCAGGCAGCCAAACTATAA | GACTCATCCTCCCTGGAGG | 1 | 27089472 | 27089622 |
| ARID1A_EX8_3MPLXID32 | CCTGGCATCCCACCTTATG | CTTGGGTTTTCCGGTTCATG | 1 | 27089577 | 27089730 |
| ARID1A_EX8_4MPLXID33 | CAATATGCCACCTCAGGTTGG | TTTCTTGCACTGACACCCTC | 1 | 27089663 | 27089815 |
| ARID1A_EX9_1MPLXID34 | CTAAAAGCTCAGAGTCTAACCTTTGT | TGCCAGCCATACTATTAATCCC | 1 | 27092658 | 27092797 |
| ARID1A_EX9_2MPLXID35 | GGGAACTGGACCTCCTTATG | TCTTGAAGAAATCCCTAGTGAGAATG | 1 | 27092751 | 27092895 |
| ARID1A_EX10_1MPLXID36 | TTCTTCAAGAGTCACATCACAGC | CTGCCTTGTTGTTCATTTTGGT | 1 | 27092886 | 27093025 |
| ARID1A_EX10_2MPLXID37 | GGGATGTAAAGTTAACTCCAGCC | CTTTTCTTTTTCCTTCCTATCACTGAAA | 1 | 27092981 | 27093130 |
| ARID1A_EX11_1MPLXID38 | TCTGAGACCCTTAGCACAGG | TTCCTCTCAGGCTCACCA | 1 | 27094215 | 27094354 |
| ARID1A_EX11_2MPLXID39 | CAATGAGAAGATCACCAAGTTGTATG | ATAGAGGTCCAGAGGTTTCCTA | 1 | 27094304 | 27094448 |
| ARID1A_EX11_3MPLXID40 | CATGACAAATCTGCCTGCTG | CCAGTGAGTACCTAGAAAGGG | 1 | 27094403 | 27094557 |
| ARID1A_EX12_1MPLXID41 | ATACCTTACAGCCTGATGGG | TGCCCACATTGAGGTTGG | 1 | 27097522 | 27097661 |
| ARID1A_EX12_2MPLXID42 | AAAATGGCGGGAACTTGC | ATCAGCAGCTGCAAAGATGT | 1 | 27097624 | 27097777 |
| ARID1A_EX12_3MPLXID43 | CTTTGAATGCAAGATTGAACGGG | CAACAGGCCCTCTCAGC | 1 | 27097717 | 27097863 |
| ARID1A_EX13_1MPLXID44 | TGTGTGAGAGTTAAACACTGTCAT | GGCTTTAAGTCTCCTCCTTCTG | 1 | 27098930 | 27099069 |
| ARID1A_EX13_2MPLXID45 | AGTCAACCAGCAGTTCCATG | TTATCTTCAGCCATCCTCCCA | 1 | 27099027 | 27099171 |
| ARID1A_EX14_1MPLXID46 | CAAGGTGGTCCTTGCCTC | AGGCATCCTGGATCCCA | 1 | 27099195 | 27099334 |
| ARID1A_EX14_2MPLXID47 | CTCTGGAGCAGGAGCAATT | TTTGGCTCATAGGACATGCG | 1 | 27099295 | 27099449 |
| ARID1A_EX14_3MPLXID48 | AGTATGAATACCTCTGACATGATGG | AGCCATTCTCAAGATTTCCCC | 1 | 27099403 | 27099557 |
| ARID1A_EX15_1MPLXID49 | TCAACTTACCAGTTTGTTCACCG | CACATTTCCTAGCCCAGGG | 1 | 27099795 | 27099934 |
| ARID1A_EX15_2MPLXID50 | GATGGGTGACCCCTACAGT | CTATAGCTTCAGGGTGCTGG | 1 | 27099886 | 27100038 |
| ARID1A_EX16_1MPLXID51 | CTGAAGCTATAGTGGGCTCAAT | GGAGAATACATCCCCGAGTCT | 1 | 27100027 | 27100166 |
| ARID1A_EX16_2MPLXID52 | CGAATCTCATGCCTTCCAAC | AAACACAGGATTAGGGTGGG | 1 | 27100124 | 27100277 |
| ARID1A_EX17_1MPLXID53 | CACCCTAATCCTGTGTTTCTTTG | GCCAGTCCATTCCCAGC | 1 | 27100260 | 27100418 |
| ARID1A_EX18_1MPLXID54 | GTGGGCTTTATGTCCCTGAG | CTGTATGGCACGCTGTACAT | 1 | 27100760 | 27100899 |
| ARID1A_EX18_2MPLXID55 | CCAAGCGGCACGAAGG | TGGTTGTATACATCTTGCTGAGG | 1 | 27100860 | 27101007 |
| ARID1A_EX18_3MPLXID56 | CCAGCCAGCAACAAGCT | GGCCAAACTGGAATGGAAATT | 1 | 27100956 | 27101099 |
| ARID1A_EX18_4MPLXID57 | GCCGACCAGCAGGCG | CTTGCTGAGCAACCTCAGC | 1 | 27101052 | 27101201 |
| ARID1A_EX18_5MPLXID58 | ATGCCACCACAAATGATGGG | TTCACGCCATGATAGGCG | 1 | 27101144 | 27101298 |
| ARID1A_EX18_6MPLXID59 | GACCTATAATTATGCCAACAGGCA | CTGGCGTGTGCCATGG | 1 | 27101230 | 27101374 |
| ARID1A_EX18_7MPLXID60 | CAACCACGAAGGCTCGT | CTGGATACCTGAGGAATGTGATT | 1 | 27101335 | 27101478 |
| ARID1A_EX18_8MPLXID61 | CCCCATATGGTCCCTCTG | ACTTGCTAGGAGAGGTGC | 1 | 27101376 | 27101528 |
| ARID1A_EX18_9MPLXID62 | CTGCCCCGGCCAATG | GATATCCCGCCGAATCATGG | 1 | 27101489 | 27101635 |
| ARID1A_EX18_10MPLXID63 | GTACCTGCCTCGCACATAG | TCTCCTTACCAATGTCTTTCATTGT | 1 | 27101576 | 27101720 |
| ARID1A_EX18_11MPLXID64 | CACCTGCCCCTGTGC | AGGCAACCGAATGAGGAAG | 1 | 27101595 | 27101740 |
| ARID1A_EX19_1MPLXID65 | TCAGAGTAGCTTCACTGATGG | GCCAGGAGACCAGACTTG | 1 | 27101980 | 27102119 |
| ARID1A_EX19_2MPLXID66 | CATGGCGGGTAATGATGTCC | AACCCTCTCAATCTTCCCCA | 1 | 27102080 | 27102233 |
| ARID1A_EX20_1MPLXID67 | TCTGTTCTTAGGCCACTTTTCTC | CACCTCATACTCCTTTAAAATGCC | 1 | 27105458 | 27105597 |
| ARID1A_EX20_2MPLXID68 | ATTTCCGACGATGCCTGATT | TCTTCTTCTTCTTCCCCACC | 1 | 27105545 | 27105689 |
| ARID1A_EX20_3MPLXID69 | GCAAGGTGTCTAGTCCAGC | TTCTCTGAAGCTGGCTTGTC | 1 | 27105641 | 27105788 |
| ARID1A_EX20_4MPLXID70 | TGAAAATGATGAGGAGATAGCCTTT | CTTATCTGAGCAGTCCACCAC | 1 | 27105735 | 27105879 |
| ARID1A_EX20_5MPLXID71 | AGTAAAGATCGTACAGAAGAATGATCC | CTGTCTTGCTCTCGAAGTGG | 1 | 27105828 | 27105979 |
| ARID1A_EX20_6MPLXID72 | ACCACTGAGCATATCCAGAC | GAGGTCCATCAGGTGGG | 1 | 27105940 | 27106096 |
| ARID1A_EX20_7MPLXID73 | GGTACACCAGGGACAACAG | CCTTGATGGCCTCTGAACTC | 1 | 27106048 | 27106192 |
| ARID1A_EX20_8MPLXID74 | ACCTTGACCGAGGATGGA | TCCAGAAGGGTACACAGTGG | 1 | 27106150 | 27106304 |
| ARID1A_EX20_9MPLXID75 | GGACGAACCCCACAGTAAG | GTGTTTGGACATCTCAAAGTCATTG | 1 | 27106257 | 27106401 |
| ARID1A_EX20_10MPLXID76 | CATTCGAAGCCTGTCATTTGTG | CTCCTCCTTTTCATAAGTTAGTGGT | 1 | 27106350 | 27106494 |
| ARID1A_EX20_11MPLXID77 | AAGCACCCAGAACGGAAG | AGATGTTGGCGAGTGTAACC | 1 | 27106447 | 27106591 |
| ARID1A_EX20_12MPLXID78 | GACTGCTTGGAGATGCTCC | CTTCAGCTGAAGGGCAAACT | 1 | 27106540 | 27106684 |
| ARID1A_EX20_13MPLXID79 | TGGACGGACTCCTACACTG | TCCACATTGTTGTCCTGGATG | 1 | 27106643 | 27106787 |
| ARID1A_EX20_14MPLXID80 | GAGACTGGTCTTGGAAACCC | GCACACCGGGTTCTTTCG | 1 | 27106734 | 27106878 |
| ARID1A_EX20_15MPLXID81 | TATAGCACTATGGTGCGCTTC | CAGGAGGTTGCCGATACTG | 1 | 27106831 | 27106977 |
| ARID1A_EX20_16MPLXID82 | CTGTGGTACTGCTGGCC | CTGCATGTGGAGGAGGC | 1 | 27106889 | 27107046 |
| ARID1A_EX20_17MPLXID83 | TGCCGCCACACAGTTC | AAACTCTGAGTGGTTCTCGTC | 1 | 27106998 | 27107142 |
| ARID1A_EX20_18MPLXID84 | GCCAACTAGTGTGGACATGAT | TGAAACCAATGAGTTCATCAACG | 1 | 27107061 | 27107205 |
| ARID1A_EX20_19MPLXID85 | CTGTTGGACATCTCGGTATCAC | GTTTCTAAGTTCTCCACACACG | 1 | 27107161 | 27107305 |
| FLT3_EX20_1MPLXID509 | AACGACACAACACAAAATAGCC | TCACCCACGGGAAAGTG | 13 | 28592550 | 28592689 |
| FLT3_EX20_2MPLXID510 | CGAGCCAATCCAAAGTCACA | AAGATTGCACTCCAGGATAATACA | 13 | 28592644 | 28592788 |
| FLT3_TKD_1MPLXID511 | CATCTTTGTTGCTGTCCTTCC | GCAGGGAAGGTACTAGGATCA | 13 | 28607991 | 28608132 |
| FLT3_TKD_2MPLXID512 | CGTTCATCACTTTTCCAAAAGCA | CCAAGAGAAAATTTAGAGTTTGGTAAGAA | 13 | 28608087 | 28608240 |
| FLT3_TKD_3MPLXID513 | AAATGCTGCAGAAACATTTGGC | GCCAGCTACAGATGGTACAG | 13 | 28608182 | 28608335 |
| FLT3_TKD_4MPLXID514 | AATCAACGTAGAAGTACTCATTATCTGAG | AATTCATTATTCTTTCCTCTATCTGCAGA | 13 | 28608276 | 28608420 |
| FLT3_TKD_5MPLXID515 | TTCAGAGATGAAATGATGAGTCAGTT | GGTGTTTGTCTCCTCTTCATTGT | 13 | 28608353 | 28608497 |
| FLT3_TKD_6MPLXID516 | CTTTTACCTTTTTGTACTTGTGACAAAT | TTCATCGCTGAGTGACACTC | 13 | 28608431 | 28608575 |
| CARD11_EX9_1MPLXID315 | ACTGGCCACAGCCCT | GGCCTGCATCGTCAACC | 7 | 2976598 | 2976737 |
| CARD11_EX9_2MPLXID316 | GTTGTTGCTGTCCTTGGAGA | CTTAATCGAAAAGGACAAGTACAGGA | 7 | 2976680 | 2976824 |
| CARD11_EX9_3MPLXID317 | CTCCAGCTCGCGGATC | CCAGCTGGATTTGGCTGG | 7 | 2976779 | 2976930 |
| CARD11_EX8_1MPLXID313 | GCTGTGCAGCCTCGC | AGTGCTCGACCCTGGG | 7 | 2977496 | 2977635 |
| CARD11_EX8_2MPLXID314 | CATGCGGTGCTTGTACATTTC | CAGCTTTCAGTCCTGACCTATT | 7 | 2977589 | 2977730 |
| CARD11_EX7_1MPLXID311 | GCTGGGCGGGCGATC | CCTGGACATCTTGGAACACG | 7 | 2978287 | 2978427 |
| CARD11_EX7_2MPLXID312 | GTAGATCCTGTTGACCAGCTC | GACCCTGACCCTCTGAAAC | 7 | 2978358 | 2978502 |
| CARD11_EX6_1MPLXID308 | CTTGGGGACTCACACCC | GAAATGCTGAAGACCAAAAACCA | 7 | 2979288 | 2979427 |
| CARD11_EX6_2MPLXID309 | CTCACCTGGATGATGGACTG | AATGTAAGCTGGAGAGAAATCAGT | 7 | 2979378 | 2979522 |
| CARD11_EX6_3MPLXID310 | GCCGATTTTCAATGTCATTCTTCA | CTGAGTGTTTCTTGCCACCT | 7 | 2979466 | 2979616 |
| CARD11_EX5_1MPLXID304 | TATGGGAGAATTGAGCCCTG | CTACGCACAGCTCAGTGAG | 7 | 2983770 | 2983909 |
| CARD11_EX5_2MPLXID305 | CATGACCGCCATGTTCTTCT | GCTGCTAACCTTCCAGGAG | 7 | 2983870 | 2984014 |
| CARD11_EX5_3MPLXID306 | CCGCTCTTCCTTCATCTTGTAG | CTGCAGCAGCAGATGAAGG | 7 | 2983969 | 2984118 |
| CARD11_EX5_4MPLXID307 | CAGCTCGCAGCGTTGC | TTTCCACTTCCCAGTCCTCA | 7 | 2984074 | 2984219 |
| CARD11_EX4_1MPLXID302 | CTCCTCTTAGAGTCCAGATGTTC | TATGTGGTCTTCTTGGAGAGC | 7 | 2985410 | 2985549 |
| CARD11_EX4_2MPLXID303 | CAGTTTGTACAGTTCTGGGTAATAAAATT | GCGTTCCATCAGATATGTATTCTCA | 7 | 2985496 | 2985640 |
| ASXL1_EX12_1MPLXID86 | ACCCAGTCAGTTAAAACTATTTTCTAATTC | CCGGCAGGAGGACTCC | 20 | 31022194 | 31022333 |
| ASXL1_EX12_2MPLXID87 | TGGTTAAAGGTCAGCCCAC | TCTCTATGGCAGTGGTGACC | 20 | 31022266 | 31022419 |
| ASXL1_Ex12_1 | GGACCCTCGCAGACATTAAAGC | CTCACCACCATCACCACTGC | 20 | 31022350 | 31022525 |
| ASXL1_EX12_3MPLXID88 | CCTCGCAGACATTAAAGCCC | TCTGCCACCTCCCTCAT | 20 | 31022354 | 31022498 |
| ASXL1_EX12_4MPLXID89 | ACCACTGCCATCGGAG | TGACGTACACTTTCCAGGG | 20 | 31022427 | 31022582 |
| ASXL1_EX12_5MPLXID90 | CCAGGGGAGGCCCGA | TTCTCAGAGAAGGCAGGTCC | 20 | 31022545 | 31022691 |
| ASXL1_EX12_6MPLXID91 | CTGCCATGTCCAGAGCTAG | CATGGCTGGTCCCCAG | 20 | 31022647 | 31022791 |
| ASXL1_EX12_7MPLXID92 | GGCTACAGTTGGACTCACAG | TAACATCCGGATGCAACTGAG | 20 | 31022717 | 31022871 |
| ASXL1_EX12_8MPLXID93 | CCCAAACCTCAGTAGCTGAG | AGAATGGGACCATTGTCTGC | 20 | 31022815 | 31022959 |
| ASXL1_EX12_9MPLXID94 | GATGATGAGGAGCAAGGACC | TCAGGTGTGGAACTGGG | 20 | 31022910 | 31023064 |
| ASXL1_EX12_10MPLXID95 | GCCAAGCTCTTGACAGTCA | ACTTTCCCTCATAGGAGGGC | 20 | 31022998 | 31023149 |
| ASXL1_EX12_11MPLXID96 | ACAGAGCATTTGATGACGAATTAG | ACCTCATCATTCGATGGGATG | 20 | 31023091 | 31023235 |
| ASXL1_EX12_12MPLXID97 | ACTTGAAAACCAAGGCTCTCG | CTTTCTCCCACTCCTCTCCA | 20 | 31023166 | 31023312 |
| ASXL1_EX12_13MPLXID98 | GAGAACACATACCATCTGTTGAGC | GATGGCACAGTCCAGAGTG | 20 | 31023262 | 31023406 |
| ASXL1_EX12_14MPLXID99 | GGGTCTAGATCCTCTTGACAG | TGAGGACTCAGTGCTTCAGA | 20 | 31023359 | 31023499 |
| ASXL1_EX12_15MPLXID100 | TGGCAGTTACTGTCAACAGG | TCTAGTGTCAGCCTCACTGC | 20 | 31023428 | 31023572 |
| ASXL1_EX12_16MPLXID101 | CCTCTGACTTTGAAGGTCACC | ATGTCACCATTCACCTTGGAC | 20 | 31023520 | 31023664 |
| ASXL1_EX12_17MPLXID102 | CAATTGGAACCAATCTGCCC | AGTACTGGCCAGCAGTAGG | 20 | 31023620 | 31023770 |
| ASXL1_EX12_18MPLXID103 | GTCTCGAGTATGTGCGGTC | CTACCCTGCAGCAACTGC | 20 | 31023713 | 31023859 |
| ASXL1_EX12_19MPLXID104 | GCCACTAACCCACTTGTGA | TAAAGATCCCATGCGTAGCG | 20 | 31023822 | 31023971 |
| ASXL1_EX12_20MPLXID105 | ACAAAAGACCAGAGCCATGG | CAAGACCAGTGCCTGTTTCA | 20 | 31023930 | 31024080 |
| ASXL1_EX12_21MPLXID106 | GCAGCCCCAGTTCTTTAAGG | AGGGAGTCAAAACTTGGGAC | 20 | 31024009 | 31024153 |
| ASXL1_EX12_22MPLXID107 | GCACCCCAAAAGAATTGCAA | GGACTTCCTTCTGATCTTCACAA | 20 | 31024110 | 31024254 |
| ASXL1_EX12_23MPLXID108 | ATCCTCTAGGAAACTGGAAGAAATG | TCGAGGTAGTAAGATCTCCTGG | 20 | 31024178 | 31024323 |
| ASXL1_EX12_24MPLXID109 | AGTAATTCAAATGCTGCTCCAG | CTTGGCCTGTAACATTGCTCT | 20 | 31024272 | 31024416 |
| ASXL1_EX12_25MPLXID110 | AAATGTGATCTCCTTTGGTCCA | AGGCATCGGGTCCGC | 20 | 31024346 | 31024487 |
| ASXL1_EX12_26MPLXID111 | CTGCAACCCTTCAGCGC | CTTTGGAGCCCAGTCTTCC | 20 | 31024447 | 31024592 |
| ASXL1_EX12_27MPLXID112 | GTTGGGACCAAGCACAAAC | TTCCTGTTCTCGGCATTTGC | 20 | 31024526 | 31024672 |
| ASXL1_EX12_28MPLXID113 | CATGCCTTTGTTGGCAGC | AAGGGCAAGTCCATGACAAA | 20 | 31024596 | 31024744 |
| ASXL1_EX12_29MPLXID114 | GGTCACTTGGAAGGGATGC | AAAGCTTCCCATAAAATGCCTG | 20 | 31024704 | 31024848 |
| ASXL1_EX12_30MPLXID115 | AATTACCCCGAGAGCCAG | AGCTAGAGGAATAATTAAAGCTGGT | 20 | 31024750 | 31024893 |
| ASXL1_EX12_31MPLXID116 | AGGGGCTCAGTGAGCC | GCTGCACCACACTTCCA | 20 | 31024771 | 31024935 |
| ASXL1_EX12_32MPLXID117 | TCCCACCTTTCCCAAAGG | CACACTGGAGCGAGATGC | 20 | 31024895 | 31025034 |
| ASXL1_EX12_33MPLXID118 | AAATGTTCACTGACAGCAGCA | ACAAGGCACAATACACAGAGC | 20 | 31024987 | 31025131 |
| ASXL1_EX12_34MPLXID119 | CAGCCTGAAAGCCATGATCA | ACACTAAATATACAATGTTTCCCATGG | 20 | 31025039 | 31025178 |
| RUNX1_EX9_1MPLXID730 | cccggaggcgaaggc | CGTGGTGGAGGCCGA | 21 | 36164379 | 36164522 |
| RUNX1_EX9_2MPLXID731 | CTCCTCCAGGCGCGC | GCGCATCCTGCCGCC | 21 | 36164453 | 36164597 |
| RUNX1_EX9_3MPLXID732 | GTTGAGCAGCGCGGA | GTTCCAAGCCAGCTCGC | 21 | 36164546 | 36164690 |
| RUNX1_EX9_4MPLXID733 | GCGCCGTAGTACAGGTG | TCACCTCGGGCATCGG | 21 | 36164649 | 36164796 |
| RUNX1_EX9_5MPLXID734 | CGAGCCCATGGCCGA | CGACCTGACAGCGTTCAG | 21 | 36164756 | 36164900 |
| RUNX1_EX9_6MPLXID735 | CCGGCGTCGGGGAGT | AACCTCCTACTCACTTCCGC | 21 | 36164797 | 36164951 |
| RUNX1_EX8_1MPLXID727 | ACCATGTTTTACTCAATAATGTTCTGC | ACCCTCTCTGCAGAACTTTC | 21 | 36171492 | 36171631 |
| RUNX1_EX8_2MPLXID728 | GTGTTTTCAAGTGGCTTACTTGAG | ACGATCAGTCCTACCAATACCT | 21 | 36171579 | 36171723 |
| RUNX1_EX8_3MPLXID729 | GGAGAGGCAATGGATCCC | GGCCACTCATTTCTTATTAAAAGACAT | 21 | 36171684 | 36171839 |
| RUNX1_EX7_1MPLXID724 | CCAGTTGGTCTGGGAAGG | CCACTCCACTGCCTTTAACC | 21 | 36206611 | 36206750 |
| RUNX1_EX7_2MPLXID725 | TACCCTGCATCTGACTCTGA | CTTGTCCTTTTCCGAGCGG | 21 | 36206704 | 36206858 |
| RUNX1_EX7_3MPLXID726 | GCTGCTCCAGTTCACTGAG | TTTTCTGATCTCTTCCCTCCCT | 21 | 36206821 | 36206964 |
| RUNX1_EX6_1MPLXID722 | GAGACATGGTCCCTGAGTATAC | CAAGTCGCCACCTACCAC | 21 | 36231692 | 36231831 |
| RUNX1_EX6_2MPLXID723 | CCCATCCACTGTGATTTTGATG | AGATATGTTCAGGCCACCAAC | 21 | 36231787 | 36231931 |
| RUNX1_EX5_1MPLXID720 | AACGTGTTTCAAGCATAGTTTTGA | CAATGATGAAAACTACTCGGCTG | 21 | 36252818 | 36252957 |
| RUNX1_EX5_2MPLXID721 | CTGCGGTAGCATTTCTCAGC | ATGTATAACATCCCTGATGTCTGC | 21 | 36252914 | 36253058 |
| RUNX1_EX4_1MPLXID716 | CCGGCCTCCGCCTGT | CCCGGGCGAGCTGGT | 21 | 36259097 | 36259236 |
| RUNX1_EX4_2MPLXID717 | CACGGAGCAGAGGAAGTTG | CAAGATGAGCGAGGCGT | 21 | 36259188 | 36259341 |
| RUNX1_EX4_3MPLXID718 | CCTCAGCTTGCCGGC | GCTTGTTGTGATGCGTATCC | 21 | 36259275 | 36259419 |
| RUNX1_EX4_4MPLXID719 | CCTGGGCTCAGCGCG | GCTGAAGATCCGCGCC | 21 | 36259342 | 36259482 |
| RUNX1_EX3_1MPLXID715 | AAAATCATATACACATCTATGAAGGTGTG | ACTTGCATTACTTTTATTTTCCTACTTTCT | 21 | 36265156 | 36265300 |
| RUNX1_EX2_1MPLXID714 | AAGCTGCCATTTCATTACAGG | TCAGAAGAGGGTGCATTTTCA | 21 | 36421073 | 36421227 |
| CSF3R_EX14_1MPLXID413 | AATCAGCATCCTTTGGGTGG | ATCCACCTCATGGCTGC | 1 | 36933356 | 36933495 |
| CSF3R_EX14_2MPLXID414 | GACTGTACTGTTGGTGGCC | CATCCAGGCCTATGCTGAC | 1 | 36933448 | 36933598 |
| PIM1_EX1_1MPLXID1145 | GCACCGTCCCTGCGC | ATCCCCGGGCCGGAG | 6 | 37138318 | 37138465 |
| PIM1_EX2_1MPLXID1146 | GAGCTGGCGGCTCGC | GGATGCCTGAGTAGACCGA | 6 | 37138496 | 37138635 |
| PIM1_EX2_2MPLXID1147 | GCTACTGGGCAGCGG | TCTCCGTCAGGTTCCCTC | 6 | 37138592 | 37138742 |
| PIM1_EX3_1MPLXID1148 | cACGGGCGTGCTTTAGC | CACCCACTCATCCTGGG | 6 | 37138702 | 37138847 |
| PIM1_EX4_1MPLXID1149 | CCGCCCTAACGCGGC | TCCAGGATCAGGACGAAACT | 6 | 37138870 | 37139022 |
| PIM1_EX4_2MPLXID1150 | TCATTAGGCTCCTGGACTGG | ACCTGCCAGAAGAAGCTG | 6 | 37138968 | 37139112 |
| PIM1_EX4_3MPLXID1151 | CGACTTCATCACGGAAAGGG | CCGCGATTGAGGTCGATAAG | 6 | 37139050 | 37139199 |
| PIM1_EX4_4MPLXID1152 | GCGACATCAAGGACGAAAAC | GTCACCTGGGCAGCTC | 6 | 37139157 | 37139302 |
| PIM1_EX5_1MPLXID1153 | TGCTAAAAGTGTGTTTTCTCTTCTATTC | CCATATCATACAGCAGGATCCC | 6 | 37140731 | 37140870 |
| PIM1_EX5_2MPLXID1154 | CGGCGGCAGTCTGGT | CAATAACCCCATGGCCAGG | 6 | 37140829 | 37140983 |
| PIM1_EX6_1MPLXID1155 | AAACAAGTTGAGTCATTCATAACCTC | ACATCTTGCATCCATGGATGG | 6 | 37141664 | 37141803 |
| PIM1_EX6_2MPLXID1156 | ATAGGCCAACCTTCGAAGAAAT | CGGGCATCTGACAAGAGAG | 6 | 37141755 | 37141909 |
| CREBBP_EX30_1MPLXID410 | CCCAGGCCGGCTGTG | CTGGTGGAGCTGCACAC | 16 | 3781152 | 3781291 |
| CREBBP_EX30_2MPLXID411 | GGTGTAGACAAAGCGGTCC | CAGCTGTGACCTCATGGATG | 16 | 3781247 | 3781397 |
| CREBBP_EX30_3MPLXID412 | AGGAAGGCGTCGCGC | CTTGTGTGGGACTAAAGCCC | 16 | 3781362 | 3781508 |
| CREBBP_EX29_1MPLXID408 | CTGCGAGTCTTTCCCTCC | CAAGAAGAAGCCCAGCATG | 16 | 3781716 | 3781855 |
| CREBBP_EX29_2MPLXID409 | CAGGTCATTGGACACGTTGG | CTGGTCTCACAGCCTTGC | 16 | 3781816 | 3781968 |
| CREBBP_EX28_1MPLXID406 | GGACACGTGGGCAATGG | GATTTCTGGCCCAATGTGTTAG | 16 | 3785999 | 3786138 |
| CREBBP_EX28_2MPLXID407 | TCTTGTTCTAGTTCCTTAATGCTCTC | GCATGGCCCTCATCTCAC | 16 | 3786089 | 3786243 |
| CREBBP_EX27_1MPLXID404 | TCCCCTCAGTTGTGACAAAAG | GCCACCCACCTGATCAAA | 16 | 3786603 | 3786754 |
| CREBBP_EX27_2MPLXID405 | ACTCCTGCAGTCGTTTTGG | CACGTTCATCTGACGTGTGT | 16 | 3786707 | 3786851 |
| CREBBP_EX26_1MPLXID402 | AATACCCATTATTTCACGGAATAAACATAC | GCACAGCCGTTTACCATGA | 16 | 3788478 | 3788617 |
| CREBBP_EX26_2MPLXID403 | TTCTTCACATACTCTAAATATCCAATAAGGA | GGATACCCTGAGTTAAACATGTGC | 16 | 3788566 | 3788710 |
| CREBBP_EX25_1MPLXID400 | CTCCTCTGGGACACTTAAGAG | GGCGTGGATGTCTGCTT | 16 | 3789497 | 3789649 |
| CREBBP_EX25_2MPLXID401 | CGTATTCTTGGACGTGCATTC | TTGTAAATGTCCGGCTCTGG | 16 | 3789607 | 3789757 |
| CREBBP_EX24_1MPLXID398 | GCAGAGCACTGTAGAGAGC | TGAACAAATTTTTGCGGCGC | 16 | 3790369 | 3790508 |
| CREBBP_EX24_2MPLXID399 | CGGCTTCAGGGTGATTCTG | GTCTCAGTCACTGTCTCAGC | 16 | 3790470 | 3790614 |
| MYD88_EX1_1MPLXID610 | TTCTCGGAAAGCGAAAGCC | GAGGAGACCGGGGCC | 3 | 38180085 | 38180238 |
| MYD88_EX1_2MPLXID611 | GCAGGAGGTCCCGGC | ATCTCCTCCGCCAGCG | 3 | 38180198 | 38180352 |
| MYD88_EX1_3MPLXID612 | CGGCCGACTGGACCG | GTAAGCAGCTCGAGCAGTC | 3 | 38180322 | 38180472 |
| MYD88_EX1_4MPLXID613 | CTGGCGCCTCTGTAGG | CTGAGGCCGCGGAAAG | 3 | 38180436 | 38180584 |
| MYD88_EX2_1MPLXID614 | TAAAGAGGTAGGCACTCCCA | GGACACTGCTGTCTACAGC | 3 | 38181302 | 38181441 |
| MYD88_EX2_2MPLXID615 | GGAGGCTGAGAAGCCTTTAC | CCACAATGTCCTATTCCACCT | 3 | 38181395 | 38181539 |
| MYD88_EX3_1MPLXID616 | ACCACCACCCTTGTGC | ACACAACTTCAGTCGATAGTTTGT | 3 | 38181852 | 38181991 |
| MYD88_EX3_2MPLXID617 | GATGATCCGGCAACTGGAA | TTCATGCATCCACGCACC | 3 | 38181946 | 38182100 |
| MYD88_EX4_1MPLXID618 | TAAGTTGCCACAGGACCTG | CTCTTGCCAGAGCAGGG | 3 | 38182207 | 38182366 |
| MYD88_EX5_1MPLXID619 | GCTTGTCCCACCATGGG | GACAGTGATGAACCTCAGGATG | 3 | 38182566 | 38182705 |
| MYD88_EX5_2MPLXID620 | GGCAATGAAGAAAGAGTTCCC | GACAGATACACACACACCCAG | 3 | 38182660 | 38182814 |
| IRF4_EX2_1MPLXID1106 | GCCTCGTGGCTGAAGG | CACCGCGCTCATGCC | 6 | 393063 | 393203 |
| IRF4_EX2_2MPLXID1107 | CCGAGGCGGAGAGTTC | CGCGTGCTTCCAGGG | 6 | 393173 | 393323 |
| IRF4_EX2_3MPLXID1108 | AGAACGAGGAGAAGAGCATCT | CCGGGCTCTGTCTCTG | 6 | 393280 | 393427 |
| IRF4_EX3_1MPLXID1109 | cagagcaggactctgattcc | GAGGGTCCGGCTTGTC | 6 | 394736 | 394875 |
| IRF4_EX3_2MPLXID1110 | CACTGTTTAAAGGAAAGTTCCGAG | TTGTACGGGTCTGAGATGTC | 6 | 394828 | 394972 |
| IRF4_EX3_3MPLXID1111 | ACTTTGAGGAACTGGTTGAGC | TAAGGTGCCTCAAGGATCTG | 6 | 394921 | 395068 |
| IRF4_EX4_1MPLXID1112 | GTTGTGCCATTTCCCTTTTCC | CGCCCTCAGGGAGCC | 6 | 395814 | 395968 |
| IRF4_EX5_1MPLXID1113 | CAATGCCAGTGCTTCTTATCTC | CATGGGACATTGGTACGGG | 6 | 397064 | 397203 |
| IRF4_EX5_2MPLXID1114 | ACTACGTCCCGGATCAGC | CCCATGGCCACATTAGCA | 6 | 397154 | 397308 |
| IRF4_EX6_1MPLXID1115 | CTTCCCAGGCTTCACACA | CCTGGGACTCAGGTGGG | 6 | 398737 | 398878 |
| IRF4_EX6_2MPLXID1116 | AGGAACCTTTTATGCTTGTGC | CAGAGCTGGCAGCAGAG | 6 | 398841 | 398995 |
| IRF4_EX7_1MPLXID1117 | ACTGACTCCGGAGCTCT | GTTGCTGGCGTCATACGT | 6 | 401397 | 401536 |
| IRF4_EX7_2MPLXID1118 | AGGGCTGCCGGATCT | GCCATCCAGAGGACCAC | 6 | 401493 | 401637 |
| IRF4_EX7_3MPLXID1119 | AACATTGAGAAGCTGCTGAGC | CTCTCTCCAGTTTGTTGGGC | 6 | 401585 | 401735 |
| IRF4_EX7_4MPLXID1120 | CGCTGTGCAACGACCG | GTGGTTTCTGACCCCTGTG | 6 | 401700 | 401846 |
| STAT5B_EX16_1MPLXID835 | CAAGAGAGATAACACACGCAGG | ATACTCCAAATACTACACACCAGTTC | 17 | 40359480 | 40359619 |
| STAT5B_EX16_2MPLXID836 | CGAACATTGTTACCAGTAGCAGA | GAGACTTCTCCATTCGGTCC | 17 | 40359563 | 40359707 |
| STAT5B_EX16_3MPLXID837 | GGTAATTCAAGTCTCCCAAGCG | GGGTTTTAAGATTTCCTAATTCAGAAATCA | 17 | 40359657 | 40359811 |
| STAT5B_EX15_1MPLXID833 | AATAATTTTGTAACGAAAGCAGCTAACT | TTAACAAGCCAGATGGGACC | 17 | 40362133 | 40362272 |
| STAT5B_EX15_2MPLXID834 | CCAATTTCTGAGTCACTGAATCTCA | ATCGTAAGTCACACTCTGCTTC | 17 | 40362221 | 40362365 |
| STAT5B_EX14_1MPLXID832 | TGTCGGCGCCTTAAGAAATAA | TGGTGTTCTTATGTTCACTGTTGT | 17 | 40362390 | 40362551 |
| STAT3_EX21_1MPLXID829 | GGATCCCAAAATTTCCAACTTTTCC | CTGACATTCCCAAGGAGGAG | 17 | 40474238 | 40474377 |
| STAT3_EX21_2MPLXID830 | CTCTCTGGCCGACAATACTTT | AGCAGCTGAACAACATGTCA | 17 | 40474329 | 40474473 |
| STAT3_EX21_3MPLXID831 | CATGATCTTATAGCCCATGATGATTTC | GAGATGACCTAGCTGTAGGTTC | 17 | 40474421 | 40474565 |
| STAT3_EX20_1MPLXID827 | CAGTGATGAGGCCTCAGC | CTCCAGGCACCTTCCTG | 17 | 40474966 | 40475105 |
| STAT3_EX20_2MPLXID828 | CTTCTTTGCTGCTTTCACTGAAT | GCCCTGTTAGCAATAACAACATT | 17 | 40475061 | 40475205 |
| STAT3_EX19_1MPLXID826 | GGTTACATCTGTGCACACTCT | AGACTTGGCTTTCCCATTACTC | 17 | 40475246 | 40475404 |
| IRF4_EX8_1MPLXID1121 | CCTGGTGTGTTCGGTGAT | GTCTGGAAACTCCTCTCCAAAG | 6 | 404958 | 405097 |
| IRF4_EX8_2MPLXID1122 | CCAAGATTCCAGGTGACTCTATG | TAAATGAAACTCTGGCCTTTACAAAG | 6 | 405053 | 405197 |
| IRF4_EX9_1MPLXID1123 | GTGCTGTTTAATAGTGAGCCAGT | CGTAGCCCCTCAGGAAATG | 6 | 407388 | 407527 |
| IRF4_EX9_2MPLXID1124 | CCAGACAACTATATTATTTTGCTCAACAA | AAAGAAAACCACTCATCTTGACATTTT | 6 | 407471 | 407625 |
| IRF4_EX9_3MPLXID1125 | ATTTACCAGAACACATCAGCAATC | GACCCCGTATCCCCGT | 6 | 407528 | 407667 |
| FOXO1_EX2_1MPLXID524 | ATGGCAAGTTACTGTGTTCCT | AAAGCTTCCCACACAGTGT | 13 | 41133573 | 41133712 |
| FOXO1_EX2_2MPLXID525 | CCAGCTATGTGTCGTTGTCT | CATGTTCATTGAGCGCTTAGAC | 13 | 41133672 | 41133816 |
| FOXO1_EX2_3MPLXID526 | CGAATGATGGATTCCATGTCAC | ATGCAGATGAGTGCCCTG | 13 | 41133772 | 41133920 |
| FOXO1_EX2_4MPLXID527 | CTCACGGAGGAGTAGCCC | CATCTGCAGTTAACGGGCG | 13 | 41133883 | 41134036 |
| FOXO1_EX2_5MPLXID528 | ATGGTGCTTACCGTGTGG | CATGATGGGCCCTAATTCGG | 13 | 41133991 | 41134140 |
| FOXO1_EX2_6MPLXID529 | GTTATGAGATGCCTGGCTGC | GACTTCTGACTCTCCTCCCC | 13 | 41134086 | 41134230 |
| FOXO1_EX2_7MPLXID530 | TACCCCAGGATCAACTGGT | GATGCCTATACAAACACTTCAGGA | 13 | 41134176 | 41134320 |
| FOXO1_EX2_8MPLXID531 | TGACTCATACCTCCATAACTCGA | CTACTCGTTTGCGCCACC | 13 | 41134267 | 41134416 |
| FOXO1_EX2_9MPLXID532 | CATATGTATATTTTTGGTAGTTTGGGCT | AACCTTCTCTCATCACCAACATC | 13 | 41134349 | 41134493 |
| FOXO1_EX2_10MPLXID533 | GTGAGGACTGGGTCGAAAC | CATTCTATGGTGTACCCGCC | 13 | 41134445 | 41134598 |
| FOXO1_EX2_11MPLXID534 | GGTAAAGTAGAGGCCATCTTTGC | CATTTCGCCCTCGAACTAGC | 13 | 41134549 | 41134693 |
| FOXO1_EX2_12MPLXID535 | GTCTCCCACTAATAGTACTAGCATTTG | CTCCAGTCTGGCCAGGA | 13 | 41134646 | 41134799 |
| FOXO1_EX2_13MPLXID536 | GGAAAACTGTGATCCAGGGC | GAGCGGGAAATCTCCTAGGA | 13 | 41134749 | 41134893 |
| FOXO1_EX2_14MPLXID537 | TACTGTTGTTGTCCATGGATGC | CGTCATAATCTGTCCCTACACAG | 13 | 41134844 | 41134988 |
| FOXO1_EX2_15MPLXID538 | TTGCCACCCTCTGGATTG | GCAGAGAAGTATTGTACTTCTAATAAGATGT | 13 | 41134894 | 41135040 |
| FOXO1_EX1_1MPLXID517 | CAGGCCGAGCAAACCT | CAAGAGCGTGCCCTACTTC | 13 | 41239638 | 41239777 |
| FOXO1_EX1_2MPLXID518 | CTGTTGCTGTCACCCTTATCC | TCGTCCCGCCGCAAC | 13 | 41239736 | 41239890 |
| FOXO1_EX1_3MPLXID519 | GTCGGCGTAGGACAGGT | TGCACCCAGCGCCAC | 13 | 41239849 | 41239997 |
| FOXO1_EX1_4MPLXID520 | gctgcgacagcggcc | CTTCCCGCAGGCGCC | 13 | 41239950 | 41240104 |
| FOXO1_EX1_5MPLXID521 | CTGGAAGTCCCCGCAC | CTGCCGCTGTCAGCG | 13 | 41240017 | 41240162 |
| FOXO1_EX1_6MPLXID522 | AAGCTCAGGTTGCTCATGAA | CCAGGCCGGAGTTTAGC | 13 | 41240123 | 41240267 |
| FOXO1_EX1_7MPLXID523 | GGTGGCCGAGTTGGAC | TCTCCTGCGGCTGGG | 13 | 41240233 | 41240382 |
| EP300_EX24_1MPLXID1072 | ACAACAGTAAATTTGCACCTCAG | AGTGACCTCTCCTGACTCAG | 22 | 41564405 | 41564544 |
| EP300_EX24_2MPLXID1073 | TGAATGACTTTCTGAGGCGAC | ATCCACGAGGAGAAGAAAAGTG | 22 | 41564495 | 41564639 |
| EP300_EX25_1MPLXID1074 | TGGTGATTCCAGTCTGAATGAG | AACACCATCAATTTCTTCAAAGGC | 22 | 41564667 | 41564806 |
| EP300_EX25_2MPLXID1075 | TTTCCATACCGAACCAAAGCC | CACACAAATCCGGAGCTAGC | 22 | 41564756 | 41564910 |
| EP300_EX26_1MPLXID1076 | TAGGCACATGGAGTAAAGAACTC | CTGCAGTCCTCAAGCATTTAGG | 22 | 41565432 | 41565571 |
| EP300_EX26_2MPLXID1077 | TCTTACCTCGATAGTGTTCATTTCTTC | CCAGAATGCCATGCTAGTTAAAG | 22 | 41565520 | 41565672 |
| EP300_EX27_1MPLXID1078 | AGATTATCTCTTTTCCTTAATGTTCTTTCTC | TTGGGCTTGGGTATCTTCTG | 22 | 41566366 | 41566505 |
| EP300_EX27_2MPLXID1079 | TTCCATTGCCATCCTCCTG | CAGATCTATTGTCAGCACCTG | 22 | 41566465 | 41566616 |
| EP300_EX28_1MPLXID1080 | AGCTTTCATGTTTCTTGTCAGC | CCTTTGCACTTGTTAATCTATCTTCAG | 22 | 41568409 | 41568548 |
| EP300_EX28_2MPLXID1081 | TACATGCATGTTTTCACAGGATATTTT | CTCTTCTTCCTCCTGTTCCAG | 22 | 41568484 | 41568628 |
| EP300_EX28_3MPLXID1082 | CCCAATGTTCTGGAAGAAAGC | GTGCACTTCTGGATTAAATATTAGAATCAT | 22 | 41568578 | 41568731 |
| EP300_EX29_1MPLXID1083 | TGCTATTCCCAAATTACTTAACAAAAACC | CCTACTCAGGCTGCTCTTATTTT | 22 | 41569565 | 41569704 |
| EP300_EX29_2MPLXID1084 | GCTAAAAAGAAGAATAATAAGAAAACCAGC | ATCTTACCTCTTTATGCTTCTCCATG | 22 | 41569651 | 41569795 |
| EP300_EX29_3MPLXID1085 | GGCAACAAGAAGAAACCCG | CATGCCTGGCCAGAAATCT | 22 | 41569705 | 41569844 |
| EP300_EX30_1MPLXID1086 | aagatcacgccactgcatt | TCAACAATGGGAGGCAGG | 22 | 41572173 | 41572312 |
| EP300_EX30_2MPLXID1087 | TTTGTGATCCGCCTCATTGC | GTGAAGAGAACTCCAGGTGC | 22 | 41572257 | 41572401 |
| EP300_EX30_3MPLXID1088 | GTTTCTCACGCTGGCAAG | CACATGGTGCTTGCATTCATT | 22 | 41572358 | 41572502 |
| EP300_EX30_4MPLXID1089 | CCAGGACCGCTTTGTCTAC | TAAAATACGTGGCTGCATGG | 22 | 41572457 | 41572608 |
| CCND3_EX5_1MPLXID358 | TTAGTGGCCACTCCAGAGG | AGCYTCTCAGACCAGCT | 6 | 41903647 | 41903786 |
| CCND3_EX5_2MPLXID359 | GCTTTGGGCGCTGGG | CTGCTGCCAGATGCTATGG | 6 | 41903751 | 41903903 |
| CCND3_EX4_1MPLXID360 | CTCAGGGCATAGCACTACTTTAT | CAGTGCAAGGCCTGGG | 6 | 41904237 | 41904376 |
| CCND3_EX4_2MPLXID361 | CTCATCCCCGGACATGG | ATCCTTCAGGTTTCCTTCTCTC | 6 | 41904336 | 41904480 |
| CCND3_EX3_1MPLXID362 | TCTTCTGATTTTTCCAATTTGGCAA | CTGGCCTTCATTCTGCACC | 6 | 41904927 | 41905066 |
| CCND3_EX3_2MPLXID363 | CCTGTCGGTCACGGG | CTTCTCCAGCCACTGACC | 6 | 41905021 | 41905173 |
| CCND3_EX2_1MPLXID364 | GCTTTCCTTTTCCTGGAAAGTG | CTGCGCGAGACCACG | 6 | 41908043 | 41908185 |
| CCND3_EX2_2MPLXID365 | TCGGTGTAGATGCACAGTTTT | CCATGAACTACCTGGATCGC | 6 | 41908136 | 41908280 |
| CCND3_EX2_3MPLXID366 | AACTGCGCCTTTCGGG | CTGTGTTCCCCACGCTC | 6 | 41908226 | 41908373 |
| CCND3_EX1_1MPLXID367 | GAGCATCCTGCAGATTGCT | CACATGCGGAAGATGCTG | 6 | 41909086 | 41909225 |
| CCND3_EX1_2MPLXID368 | CGTCCGGGCGGTACC | GGACCAGCGTGTCCTG | 6 | 41909176 | 41909319 |
| CCND3_EX1_3MPLXID369 | GTACGTAGCGCTCCTCCA | CGCACTCCCGCCCTG | 6 | 41909270 | 41909420 |
| CD79A_EX4_1MPLXID370 | TCTGAAAGATATTCACCTCCCC | GCCCCTACCCCATCCC | 19 | 42384678 | 42384835 |
| CD79A_EX5_1MPLXID371 | GGGGTGTTCCCTCTGG | AGGTGCCCTGGAGGC | 19 | 42384852 | 42384994 |
| CD79A_EX5_2MPLXID372 | ATGTATGAGGACATCTCCCGG | AAGTGAGCTGAGACACTGGA | 19 | 42384958 | 42385112 |
| SETBP1_EX4_1MPLXID736 | ATGCTCATCTTTGTTtctctctct | TTTTAAGGTGTCACCGGTGAA | 18 | 42529787 | 42529926 |
| SETBP1_EX4_2MPLXID737 | ACTCTCCATTATGACACGGG | TGATGAAGCAATTCTGAGTGA | 18 | 42529873 | 42530017 |
| SETBP1_EX4_3MPLXID738 | TGGTCCACCAACTCTGACA | CACTGCCTTTCTTACCCTGG | 18 | 42529969 | 42530113 |
| SETBP1_EX4_4MPLXID739 | CAAGATCCCCGCTCTTGAG | TGAGCTGTGGCTGCTTG | 18 | 42530052 | 42530199 |
| SETBP1_EX4_5MPLXID740 | GAGTCAGTTGTCTAACAATAACAAAGATC | TGTACCTCCTCCATCTTGATCC | 18 | 42530127 | 42530271 |
| SETBP1_EX4_6MPLXID741 | CGGGCTTCAGCCCTTG | ATTCTTGACCCAATCCAGGTC | 18 | 42530232 | 42530376 |
| SETBP1_EX4_7MPLXID742 | TGGGCAGCAAGAAAAAGTC | GATGCCTCTTGGGCACTATC | 18 | 42530297 | 42530447 |
| SETBP1_EX4_8MPLXID743 | CATTTGACAATACAGAAGGGAAAAGG | CCAGAGAGGGTGCCTTG | 18 | 42530387 | 42530539 |
| SETBP1_EX4_9MPLXID744 | ACTCAAGTCATGTCCGGATTAC | CTCATGGTGATTCCAGAAGCC | 18 | 42530492 | 42530636 |
| SETBP1_EX4_10MPLXID745 | GTGGAAAAGATCATGCCAGAGA | GGGACTCATTCTCTATCATTTTACTCA | 18 | 42530587 | 42530731 |
| SETBP1_EX4_11MPLXID746 | GAATAAGAAGGATCCCCGTGTC | TTGGAGGTGTCATGACCATG | 18 | 42530676 | 42530827 |
| SETBP1_EX4_12MPLXID747 | GCTGAGAAAGTTATCCCAGGAG | AAGTCCACCTTCGTTTTGCA | 18 | 42530761 | 42530905 |
| SETBP1_EX4_13MPLXID748 | CAGAAAGCTGCCAGAAATCC | TGATGGGATATGCAGACGG | 18 | 42530850 | 42530998 |
| SETBP1_EX4_14MPLXID749 | ACCTCTGATAAACTGATGCTGG | GTCTCGACTGTGAGCAAAGG | 18 | 42530953 | 42531104 |
| SETBP1_EX4_15MPLXID750 | GTGATCACTCCAGTCAAAAAGAAG | TCGTCGCTTTCTTTTCTTAGTGC | 18 | 42531040 | 42531183 |
| SETBP1_EX4_16MPLXID751 | TCAGCCGAGAGTTTCCTG | TTCTTATCCAACACGCCGAG | 18 | 42531143 | 42531287 |
| SETBP1_EX4_17MPLXID752 | ATGAAATTTCACAAGAAAGTTGGAAAG | CTTTTGCCTTCAGAGCAACG | 18 | 42531241 | 42531385 |
| SETBP1_EX4_18MPLXID753 | ATCAGATCTTGTCCTGTTCCAG | CGCTTCTTGCCAATGTAGATG | 18 | 42531341 | 42531485 |
| SETBP1_EX4_19MPLXID754 | AACTGGGCAAGCAGATTAATGT | CATCCGGCTGGCTAGAAAC | 18 | 42531428 | 42531577 |
| SETBP1_EX4_20MPLXID755 | CAAAACAGCCATCAAGCACC | CTCAGGTTGCCATTGGACC | 18 | 42531531 | 42531677 |
| SETBP1_EX4_21MPLXID756 | TATGCACCCACTTTCAACACA | GTGTATTCCCTTTTGGGTTTTGG | 18 | 42531630 | 42531774 |
| SETBP1_EX4_22MPLXID757 | GAGTTGAAAACTATGCCAAATCTCC | CTTTAGGGAGCCAATCTCGC | 18 | 42531706 | 42531852 |
| SETBP1_EX4_23MPLXID758 | ACCTGGAAGCTGTCTCCA | TTGTTGTCTGTCCCAATGCC | 18 | 42531781 | 42531932 |
| SETBP1_EX4_24MPLXID759 | AGACGATCCCCAGCGA | TTCTTTGTGCTGGTGTCGG | 18 | 42531893 | 42532043 |
| SETBP1_EX4_25MPLXID760 | TTTCTGCTCCCTGGACAAC | TTTCCGTTTCCTCTTGTGCT | 18 | 42531990 | 42532134 |
| SETBP1_EX4_26MPLXID761 | TCATTGTGGACAACTTTCTGGC | GAGATTCTGAACACTTGGAACTTG | 18 | 42532070 | 42532214 |
| SETBP1_EX4_27MPLXID762 | GTTTCTGGCAGACCTGGAG | TACTGGATATATGGCACCGGG | 18 | 42532161 | 42532307 |
| SETBP1_EX4_28MPLXID763 | CCAGCATTTTTCGGATTAATTTTGATC | CTTTGTCATGGTGTCATTGGTTT | 18 | 42532253 | 42532398 |
| SETBP1_EX4_29MPLXID764 | CTTGAAGTCAAAGAAGAAGCGTG | CATAGGCATTGATGTGTAAGGC | 18 | 42532341 | 42532485 |
| SETBP1_EX4_30MPLXID765 | CCTATTCCCAGTGGAAGTTACTATG | CTGTTGGCCTCATGAATGGG | 18 | 42532426 | 42532573 |
| SETBP1_EX4_31MPLXID766 | CTATCGCACACGCTTGGA | CATGCTAACAGGTCCCTGC | 18 | 42532528 | 42532674 |
| SETBP1_EX4_32MPLXID767 | CTAAGCATAAAGCCAAGCATGG | TTGTGTTTGTGTTTCCTCTTATGG | 18 | 42532625 | 42532769 |
| SETBP1_EX4_33MPLXID768 | CAAGGTAGGCAGTGCCAG | GGAGAAGCCTGTGGCTTT | 18 | 42532710 | 42532851 |
| SETBP1_EX4_34MPLXID769 | AAGACCGGATCCTAGGGAC | TCCTTATGCTTGTTCTTCTCACTC | 18 | 42532784 | 42532928 |
| SETBP1_EX4_35MPLXID770 | GAGTAGCGCAGACAAAGAGC | TCGGAAAGTGACAGTGTAGAC | 18 | 42532875 | 42533018 |
| SETBP1_EX4_36MPLXID771 | AAGCTTCTAAGAACAACTTTGAGGT | GCCACTGCCAGAGTATCTTT | 18 | 42532964 | 42533106 |
| SETBP1_EX4_37MPLXID772 | GGAGACTTGAGCAGTGAGC | TTACTCCCACTCACGTCACT | 18 | 42533050 | 42533201 |
| SETBP1_EX4_38MPLXID773 | GTGAAATGAACCCTTCGAATGAC | GAGGAGTCATAAGAACTTCTCTCC | 18 | 42533150 | 42533294 |
| SETBP1_EX4_39MPLXID774 | TATGAAGGCTTTGGAACGTACA | CAATGAAATAACTTGATAATCCATTTCTGAAG | 18 | 42533212 | 42533351 |
| MPL_EX10_1MPLXID592 | GCCGAAGTCTGACCCTTTT | AGTCTCCTGCCTGGCG | 1 | 43814905 | 43815056 |
| MPL_EX10_1 | TTGGTGACCGCTCTGCATCTA | GGTCACAGAGCGAACCAAGAATG | 1 | 43814945 | 43815117 |
| U2AF1_EX6_1MPLXID973 | TGTGCTCAGTCACGTCAC | GTGATTGACTTGAATAACCGTTGG | 21 | 44514729 | 44514868 |
| U2AF1_EX6_2MPLXID974 | GTGGATCGGCTGTCCATTAA | ACTTGGATTGGTAATTGAGAAAGTCT | 21 | 44514824 | 44514968 |
| U2AF1_EX2_1MPLXID971 | ATGACTTGCTTAATATGTAGAAATTAACTGTC | ATGTCGTCATGGAGACAGGT | 21 | 44524340 | 44524479 |
| U2AF1_EX2_2MPLXID972 | CTAAACGTCGGTTTATTGTGCAA | GACATATTCCATGTGTTTGATATCTTCC | 21 | 44524429 | 44524574 |
| B2M_EX1_1MPLXID978 | CATTCCTGAAGCTGACAGCA | CACAGAGGGTGCAGAGC | 15 | 45003714 | 45003869 |
| B2M_EX2_1MPLXID979 | GACCAAATGTAAACACTTGGTGC | TGCTGGATGACGTGAGTAAAC | 15 | 45007519 | 45007658 |
| B2M_EX2_2MPLXID980 | CCGATATTCCTCAGGTACTCCA | AATTCTCTCTCCATTCTTCAGTAAGTC | 15 | 45007607 | 45007751 |
| B2M_EX2_3MPLXID981 | TTTCATCCATCCGACATTGAAGT | CGGCAGGCATACTCATCTT | 15 | 45007701 | 45007855 |
| B2M_EX2_4MPLXID982 | TCTTGTACTACACTGAATTCACCC | ACTACTCATACACAACTTTCAGCA | 15 | 45007804 | 45007948 |
| B2M_EX3_1MPLXID983 | AGACATTTGTTAGTACATGGTATTTTAAAAGT | GTCCTCAGGACAGTGAAACAA | 15 | 45008450 | 45008603 |
| RHOA_EX2_1MPLXID712 | ATTCTAACATGGAAAATGGCATCAG | CTCATAGTCTTCAGCAAGGACC | 3 | 49412820 | 49412959 |
| RHOA_EX2_2MPLXID713 | GCACATACACCTCTGGGAAC | TGTGCATTGCAGGTAATATCTGT | 3 | 49412917 | 49413061 |
| JAK2_EX12_1MPLXID569 | CCTCTTTGGAGCAATTCATACTTTC | AGGCCTCTGTAATGTTGGTG | 9 | 5069859 | 5069998 |
| JAK2_EX12_2MPLXID570 | GGTGTTTCTGATGTACCAACCT | GAAAACAGATGTTGTTTTAAAAGGACAAA | 9 | 5069957 | 5070111 |
| JAK2_EX14_1MPLXID571 | ACAGTCAAACAACAATTCTTTGTACTT | AGAAAGGCATTAGAAAGCCTGT | 9 | 5073657 | 5073819 |
| KIT_EX8_1MPLXID572 | CAGCCTCAGGAAGGTTGTAG | TGCCATTCACGAGCCTG | 4 | 55589652 | 55589791 |
| KIT_EX8_2MPLXID573 | GTAGCAAAACCAGAAATCCTGAC | CTGCATTATAAGCAGTGCCAAAA | 4 | 55589746 | 55589900 |
| KIT_EX9_1MPLXID574 | TTTTAAAAGTATGCCACATCCCAAG | GGCCCAGATGAGTTTAGTGT | 4 | 55591937 | 55592076 |
| KIT_EX9_2MPLXID575 | CTTCTGTACTGCCAGTGGATG | CAGAAGTCTTGCCCACATCG | 4 | 55592031 | 55592180 |
| KIT_EX9_3MPLXID576 | CAATGGCACGGTTGAATGTAAG | GACTGATATGGTAGACAGAGCC | 4 | 55592131 | 55592275 |
| KIT_EX10_1MPLXID577 | AGTGGCTGTGGTAGAGATCC | ATAATGCACATCATGCCAGCT | 4 | 55593320 | 55593459 |
| KIT_EX10_2MPLXID578 | CACTCCTTTGCTGATTGGTTTC | AGATCACCTTTTAATAATTATTGTCTCAGTC | 4 | 55593409 | 55593559 |
| KIT_EX11_1MPLXID579 | TGACTGAGACAATAATTATTAAAAGGTGATC | ATAAGGAAGTTGTGTTGGGTCTAT | 4 | 55593528 | 55593668 |
| KIT_EX11_2MPLXID580 | TTGAGGAGATAAATGGAAACAATTATGTTT | AGTCACTGTTATGTGTACCCAAAA | 4 | 55593613 | 55593765 |
| KIT_EX12_1MPLXID581 | AAATGGTCCTTCAATTCCACCA | GCCGCATCTGACTTAATTAAGC | 4 | 55593925 | 55594064 |
| KIT_EX12_2MPLXID582 | GTTGTTGAGGCAACTGCTTATG | AAATTGGAAACATGCATTTTAGCAAAA | 4 | 55594021 | 55594173 |
| KIT_EX13_1MPLXID583 | GATGCTCAAGCGTAAGTTCCT | TTGAGTTCAGACATGAGGGC | 4 | 55594083 | 55594222 |
| KIT_EX13_2MPLXID584 | GAGTGCCCATTTGACAGAAC | ACAGACAATAAAAGGCAGCTTG | 4 | 55594178 | 55594322 |
| KIT_EX17_1MPLXID585 | AAGTTAGTTTTCACTCTTTACAAGTTAAAATGA | AAATCACAAATCTTTGTGATCCGAC | 4 | 55599167 | 55599306 |
| KIT_EX17_2MPLXID586 | AGACTTGGCAGCCAGAAATAT | CTTTGCAGGACTGTCAAGCA | 4 | 55599247 | 55599394 |
| STAT6_EX14_1MPLXID846 | CAGAGACAGAGGACTGGCT | ACGTTACTAGCCTTCTTCTCAATG | 12 | 57493518 | 57493657 |
| STAT6_EX14_2MPLXID847 | AGGAGAAAGGTTCCGTCG | TAGTCTGAGCAGCCATACAC | 12 | 57493612 | 57493760 |
| STAT6_EX13_1MPLXID845 | GCTCAGACTACCCAGGGT | CTCGGCTCACACCTTTCC | 12 | 57493751 | 57493905 |
| STAT6_EX12_1MPLXID842 | CACTGTGGAGAATCCAGTGG | ACCGTTCTGTGTCCTGGT | 12 | 57495965 | 57496104 |
| STAT6_EX12_2MPLXID843 | AGGAGAATGACCTTGTTGAACT | CTGAAGTTCATGGCTGAGGT | 12 | 57496062 | 57496216 |
| STAT6_EX12_3MPLXID844 | CCCCGGTTGGTCCCC | ATAACAAGACGGTAGCCTGC | 12 | 57496182 | 57496328 |
| STAT6_EX11_1MPLXID841 | TTCCCTCCAACTCCAGGA | GAAAAGAAACCCCTGTCCTCA | 12 | 57496584 | 57496735 |
| STAT6_EX10_1MPLXID839 | GCATTGGAGGAGGACTCG | TGAGTCTGTCACAGAGGAGAA | 12 | 57498192 | 57498331 |
| STAT6_EX10_2MPLXID840 | GCAGAGAAGAGCACAGCG | ATGTCAGGAAGCCCAGGA | 12 | 57498290 | 57498442 |
| STAT6_EX9_1MPLXID838 | AGTCTCCTAGTGGTGCCC | GCTCGTTCACACTCTGACC | 12 | 57498464 | 57498626 |
| BCL2_EX3_1MPLXID285 | TGCTTTAGTGAACCTTTTGCATATTT | TTGATTTCTCCTGGCTGTCTC | 18 | 60795810 | 60795949 |
| BCL2_EX3_2MPLXID286 | CCAAACTGAGCAGAGTCTTCA | CATTCTCTGCTCTCTCTCTAATACTC | 18 | 60795908 | 60796052 |
| BCL2_EX2_1MPLXID278 | ATTTCGCCGGCTCCAC | CACACCTGGATCCAGGATAAC | 18 | 60985205 | 60985344 |
| BCL2_EX2_2MPLXID279 | CAAGTGCACCTACCCAGC | CCTTCTTTGAGTTCGGTGGG | 18 | 60985302 | 60985454 |
| BCL2_EX2_3MPLXID280 | GGTTGACGCTCTCCACAC | GAGATGTCCAGCCAGCTG | 18 | 60985410 | 60985560 |
| BCL2_EX2_4MPLXID281 | GTGGCAAAGCGTCCCC | CTGCGCTCAGCCCGG | 18 | 60985505 | 60985649 |
| BCL2_EX2_5MPLXID282 | GGGAGAAGTCGTCGCC | CATCCAGCCGCATCCC | 18 | 60985584 | 60985728 |
| BCL2_EX2_6MPLXID283 | AGGTCCTGGCGACCG | TTATAAGCTGTCGCAGAGGG | 18 | 60985692 | 60985840 |
| BCL2_EX2_7MPLXID284 | cggcgcccACATCTC | GAGGTGCCGTTGGCC | 18 | 60985788 | 60985938 |
| XPO1_EX15_1MPLXID975 | ATTCATTTATTTTGTCCTGGACTCC | TAGTTAACAAGCTGTTCGAATTCATG | 2 | 61719350 | 61719489 |
| XPO1_EX15_2MPLXID976 | GCAAAATATAGTAAAGAAAGAGATTTACCATG | GGCAAAGATAATAAAGCTATTATTGCATCA | 2 | 61719432 | 61719586 |
| XPO1_EX15_3MPLXID977 | CGTGGGTATTGACCTACTATGTAC | TGCTAAATAAGTATTATGTTGTTACAATAAATAAT | 2 | 61719525 | 61719672 |
| CD79B_EX6_1MPLXID374 | CCTGCACCCAGGTCATG | CCGGCCTGAGTTCCAC | 17 | 62006555 | 62006713 |
| CD79B_EX5_1MPLXID373 | CAACCACACCAGCAGATAGT | AACACTCTGATCTCCATCCCT | 17 | 62006724 | 62006868 |
| GNA13_EX4_1MPLXID1098 | AGATTGTTCTAATTCTGGTTGTAAACTG | TTCTGCATGACAACCTCAAGC | 17 | 63010273 | 63010412 |
| GNA13_EX4_2MPLXID1099 | ACAGCAAGTCTTTTGTACATCACT | CAGCAACAGAAGCCCTTATACC | 17 | 63010356 | 63010500 |
| GNA13_EX4_3MPLXID1100 | TTCTCCGTGTTGATAGCAGTG | CAAAGACTATTTCCTAGAATTTGAAGGG | 17 | 63010448 | 63010594 |
| GNA13_EX4_4MPLXID1101 | TTTTGGACGTCTCTTAAGCAGT | TCAATAACCGGGTTTTCAGCA | 17 | 63010538 | 63010682 |
| GNA13_EX4_5MPLXID1102 | AAGTCTGTCTTGTTTAAGAACAGAATTATG | GTTTCCTCAAGTGAATTTGACCAG | 17 | 63010625 | 63010770 |
| GNA13_EX4_6MPLXID1103 | CGATTGGTCAGTCGATCTTCC | TTCCTTTCAAAATGGTTGATGTAGG | 17 | 63010718 | 63010862 |
| GNA13_EX4_7MPLXID1104 | CAAACCAACGTTTCCTTTCTGAT | CCAGGATTATATTCCATCACAACAAG | 17 | 63010806 | 63010951 |
| GNA13_EX4_8MPLXID1105 | TTTTATTTCAAAGTCGTATTCATGGATGC | ATTAAAAATTCGCATTACTTCGGGATT | 17 | 63010867 | 63011006 |
| GNA13_EX3_1MPLXID1097 | CATCGATATCCTGACTTCTGCTT | TCTAAGCCCTAATAACTATTTGTTTTCTTC | 17 | 63014307 | 63014461 |
| GNA13_EX2_1MPLXID1094 | ACTAAACATCATTAAAATAAAACCACACGT | TTTCTTACAATATCTTCCTGCTATAAGAGC | 17 | 63049568 | 63049707 |
| GNA13_EX2_2MPLXID1095 | CATTCTGTATGCCGCTGTCT | AACTCAAACCAACAACATGGAGA | 17 | 63049649 | 63049793 |
| GNA13_EX2_3MPLXID1096 | CCGGGTATCAAACGACATCA | TGTAATGACTCCTCTCCATACATTTT | 17 | 63049746 | 63049893 |
| GNA13_EX1_1MPLXID1090 | TTCCTTCGCCAGCGAC | TTCCGCCCCACCATCT | 17 | 63052323 | 63052462 |
| GNA13_EX1_2MPLXID1091 | caccTTTGATCACGTTGCTG | CTGGTGAAGATCCTGCTGC | 17 | 63052426 | 63052570 |
| GNA13_EX1_3MPLXID1092 | CTTCAGGAAGGTGGACTTGC | CTGCCTGCTGACGAGTG | 17 | 63052514 | 63052661 |
| GNA13_EX1_4MPLXID1093 | GACTTGCGTTGCTGCTC | CCGCCGGAGGAGGAG | 17 | 63052620 | 63052774 |
| SRSF2_EX1_2 | GCCGCGGACCTTTGTGA | GCAAATGGCGCGCTACG | 17 | 74732803 | 74732982 |
| SRSF2_EX1_1MPLXID821 | GGACCTTTGTGAGGTcgc | ACACCACAGCCGCCG | 17 | 74732808 | 74732949 |
| SRSF2_EX1_2MPLXID822 | CCCCGTACCTGCGGG | CTTTCACGACAAGCGCGA | 17 | 74732909 | 74733060 |
| SRSF2_EX1_1 | CGTACCTGCGGGGTGG | CCTTCGTTCGCTTTCACGACAA | 17 | 74732912 | 74733070 |
| SRSF2_EX1_3 | GGCGGCTGTGGTGTGA | CCGCGGGACCGCTACA | 17 | 74732936 | 74733107 |
| SRSF2_EX1_3MPLXID823 | GTCCATGGCATCCATAGCG | CACGCTGAGGCGCGT | 17 | 74733015 | 74733159 |
| SRSF2_EX1_4 | CTTGTCGTGAAAGCGAACGAAG | CTCAAGGTGGACAACCTGACCTA | 17 | 74733048 | 74733197 |
| SRSF2_EX1_4MPLXID824 | CGCGGGATGTACACGTC | TCAGAGCTATGAGCTACGGC | 17 | 74733103 | 74733250 |
| SRSF2_EX1_5MPLXID825 | CCGACGCGCCCGTAC | GTTCGCGGGGCTCCG | 17 | 74733121 | 74733274 |
| TP53_EX11_1MPLXID970 | CTGTCAGTGGGGAACAAGAA | ATGTCATCTCTCCTCCCTGC | 17 | 7572897 | 7573043 |
| TP53_EX10_1MPLXID968 | CTATGGCTTTCCAACCTAGGAA | CCGAGAGCTGAATGAGGC | 17 | 7573865 | 7574004 |
| TP53_EX10_2MPLXID969 | GGGCATCCTTGAGTTCCAA | AAACAATTGTAACTTGAACCATCTTTTAAC | 17 | 7573967 | 7574111 |
| TP53_EX9_1MPLXID967 | GTTAGACTGGAAACTTTCCACTTG | CACTTTTATCACCTTTCCTTGCC | 17 | 7576805 | 7576959 |
| TP53_EX8_1MPLXID965 | GATAAAAGTGAATCTGAGGCATAACTG | CACAGAGGAAGAGAATCTCCG | 17 | 7576950 | 7577089 |
| TP53_EX8_2MPLXID966 | GTGAGGCTCCCCTTTCTTG | CCTTACTGCCTCTTGCTTCTC | 17 | 7577050 | 7577194 |
| TP53_EX7_1MPLXID964 | GTGCAGGGTGGCAAGT | CCTCATCTTGGGCCTGTG | 17 | 7577473 | 7577637 |
| TP53_EX6_1MPLXID962 | GGAGGGCCACTGACAAC | AGGAAATTTGCGTGTGGAGT | 17 | 7578116 | 7578255 |
| TP53_EX6_2MPLXID963 | ACACTATGTCGAAAAGTGTTTCTGT | GACAGGGCTGGTTGCC | 17 | 7578202 | 7578346 |
| TP53_EX5_1MPLXID959 | GCCCTGTCGTCTCTCCA | GGGTTGATTCCACACCCC | 17 | 7578339 | 7578493 |
| TP53_EX5_2MPLXID960 | CTGCTTGTAGATGGCCATGG | CTCTGTCTCCTTCCTCTTCCT | 17 | 7578435 | 7578579 |
| TP53_EX5_3MPLXID961 | ACAGCTGCACAGGGC | CTTTGCTGCCGTCTTCCA | 17 | 7578494 | 7578633 |
| TP53_EX4_1MPLXID956 | TGGAAGCCAGCCCCT | CCCCTCCTGGCCCCT | 17 | 7579284 | 7579423 |
| TP53_EX4_2MPLXID957 | TAGGTTTTCTGGGAAGGGACA | GTTCACTGAAGACCCAGGTC | 17 | 7579379 | 7579528 |
| TP53_EX4_3MPLXID958 | GGCATTCTGGGAGCTTCAT | GGCTGAGGACCTGGTC | 17 | 7579487 | 7579635 |
| TP53_EX3_1MPLXID955 | TGGGTGAAAAGAGCAGTCAG | ATGGGACTGACTTTCTGCTC | 17 | 7579598 | 7579752 |
| TP53_EX2_1MPLXID954 | GCCTGCCCTTCCAATGG | ACTTTTCCTCTTGCAGCAGC | 17 | 7579812 | 7579956 |
| PLCG2_EX17_1MPLXID670 | CAAGGCTTTCAGAAACCCCT | CTCCATGCAGTATTCCTGCA | 16 | 81941983 | 81942122 |
| PLCG2_EX17_2MPLXID671 | ACGAGTGCCGAGAAGTTG | AAGAAGGTAGGCGTTCCCT | 16 | 81942084 | 81942231 |
| PLCG2_EX18_1MPLXID672 | GTGTGGCCACATGTAATGTC | CCATGGTGGAGCGGATC | 16 | 81944029 | 81944168 |
| PLCG2_EX18_2MPLXID673 | ACTGATGGCGTCCTCTCC | TCTCGCGGTAGTGCTGG | 16 | 81944101 | 81944249 |
| PLCG2_EX18_3MPLXID674 | CTCACCTTCAGCAGCATCTAT | TGCTCCACCCTCAGACA | 16 | 81944204 | 81944347 |
| PLCG2_EX18_4MPLXID675 | CGCACCTGCGCTGCG | GGCCCGGCTGAACCA | 16 | 81944250 | 81944413 |
| PLCG2_EX19_1MPLXID676 | CTGGTGCCATTATCTTGTCCT | CCTCATCAGCATGTCCTCTG | 16 | 81946114 | 81946253 |
| PLCG2_EX19_2MPLXID677 | GTACTATGACAGCCTGAGCC | CTCTGTAGAGCATGTGCCTC | 16 | 81946205 | 81946357 |
| PLCG2_EX20_1MPLXID678 | GATCTTGGCATGTCAACCCT | TAACTGACGAGCTCCACCA | 16 | 81953051 | 81953190 |
| PLCG2_EX20_2MPLXID679 | GGACCTCCGCCTATTTTGAG | AAATCACAAGGGAGTCCACC | 16 | 81953148 | 81953295 |
| PLCG2_EX20_3MPLXID680 | CTACGAGAAGCATTCACTCTACC | CAGATGAGTTACAACCTCAGACAA | 16 | 81953191 | 81953330 |
| PLCG2_EX21_1MPLXID681 | TTCCAGGAGCATGGATTATTCC | CCAAAATCATGTGAATTGGAGGTT | 16 | 81954755 | 81954909 |
| PLCG2_EX22_1MPLXID682 | AAGAACCAAATGGTACCTGGG | CTCATCGCTTCGCTTGGC | 16 | 81957004 | 81957143 |
| PLCG2_EX22_2MPLXID683 | CGTGAAAGCTCTGTATGACTACA | AAGAAGGGACAAGTCAAGCC | 16 | 81957101 | 81957255 |
| PLCG2_EX23_1MPLXID684 | CATCAGAATTGAGCCAGCAG | ATGGGAAGTACTGCTGGATC | 16 | 81960591 | 81960730 |
| PLCG2_EX23_2MPLXID685 | TGGAAAGGAGACTATGGAACCA | GACCTGCAGATCCCTTCTTC | 16 | 81960688 | 81960832 |
| IDH2_EX4_1MPLXID9 | TCTGTGGCCTTGTACTGC | ACCAAGCCCATCACCATT | 15 | 90631721 | 90631860 |
| IDH2_EX4_2MPLXID10 | GGCCTACCTGGTCGCCAT | TGAAGAAGATGTGGAAAAGTCCCAA | 15 | 90631812 | 90631970 |
| IDH2_EX4_3MPLXID11 | GTTTTTGCAGATGATGGGCTCC | GGGGTTCAAATTCTGGTTGAAAGA | 15 | 90631885 | 90632044 |
