# Supplementary material for: A reduced panel of eight genes (ATM, SF3B1, NOTCH1, BIRC3, XPO1, MYD88, TNFAIP3, and TP53) as an estimator of the tumor mutational burden in chronic lymphocytic leukemia
Source: Int J Lab Hematol. 2020 Dec 16;43(4):683–92. doi: 10.1111/ijlh.13435 (PMC8451785; doi:10.1111/ijlh.13435)
Supplement: Supplementary file 19 — Supplementary Methods [file IJLH-43-683-s013.docx]

Supplementary Material and Methods

For series 1, the obtained FASTQ files were aligned using the Torrent Mapping Alignment Program (TMAP) software included into the Torrent Suite (Thermo Fisher Scientific, Waltham, Massachusetts, USA) on the GRCh37 (hg19) version of the human genome. Variant calling was performed with the Torrent Variant Caller (TVC) also included into the Torrent Suite. An in-house tool, based on ANNOVAR (Wang et al., ANNOVAR: functional annotation of genetic variants from high-throughput sequencing data, Nucleic Acids Research, Volume 38, Issue 16, 1 September 2010, Page e164, https://doi.org/10.1093/nar/gkq603) was then used for the annotation of the different variants by checking databases for Single Nucleotide polymorphisms (Human dbSNP Database at NCBI, <https://www.ncbi.nlm.nih.gov/snp/>, GnomAD, https://gnomad.broadinstitute.org/ or 1000Genome, https://www.internationalgenome.org/) and Cancer (COSMIC database, <https://cancer.sanger.ac.uk/cosmic>). We also integrated pathogenicity scores according to SIFT and CADD prediction tools.

For series 2, the FASTQ files were generated with Bcl2Fastq2 conversion software. Reads were filtered using an in-house Perl script and mapped to the Human genome build (hg38) by using the Burrows-Wheeler Aligner (BWA) tool. An in-house tool including samtools and Perls scripts was used for variant detection. Variant calling was performed by using Ensembl Variant Effect Predictor (VEP). The mutations detected in the two technical duplicates are interpreted as described below.

Variants were then classified according to the American College of Medical Genetics guidelines (ACMG). Briefly, variants were filtered according to sequencing depth (≥ 100X and supported by ≥5 mutated reads) and minimum variant allele frequency (VAF) was set to 2% to limit reporting of sequencing errors. All variants with a minor allele frequency (MAF) in the general population ≥0.01 were considered as polymorphisms. Highly recurrent hotspot mutations in CLL were identified based on bibliography and by systematic screening of databases for cancer mutations (COSMIC, ClinVarn, IARC TP53 databases). Validation of non-hotspot mutations was based on positive bioinformatics prediction of pathogenicity in order to control the false discovery rate of new variants. Variants with no available classification by SIFT or CADD scores were also evaluated but all tolerated variants were not considered. For other variants with an entry in dbSNP, GnomAD or 1000 genome databases but with MAF < 0.01 or with absence of available annotations were specifically reviewed by two of us (JC, DR) to strictly retain highly plausible mutations only. Finally, only pathogenic (class 5) and likely pathogenic (class 4) variants were retained.
